# Supplementary material for: Neural Inflammation in Thoracic Dorsal Root Ganglia Mediates Cardiopulmonary Spinal Afferent Sensitization in Chronic Heart Failure
Source: bioRxiv. 2025 Oct 23:2025.10.22.683960. Preprint. [Version 1] doi: 10.1101/2025.10.22.683960 (PMC12633454; doi:10.1101/2025.10.22.683960)
Supplement: Supplement 1 [file media-1.pdf]

Supplemental Materials

Neural Inflammation in Thoracic Dorsal Root Ganglia Mediates Cardiopulmonary Spinal Afferent Sensitization in Chronic Heart Failure

Juan Hong[1]#, Samuel Gillman[1,2]#, Peter Pellegrino[1], Gang Zhao[4], Rongguo Ren[4], Steven J. Lisco[1], Irving H. Zucker[3], Dong Wang[4], Han-Jun Wang[1]

1 Department of Anesthesiology, University of Nebraska Medical Center, Omaha, NE 68198

2 Department of Genetics, Cell Biology, & Anatomy, University of Nebraska Medical Center, Omaha, NE 68198

3 Cellular and Integrative Physiology, University of Nebraska Medical Center, Omaha, NE 68198

4 Department of Pharmaceutical Sciences, University of Nebraska Medical Center, Omaha, NE 68198

# Both authors contribute equally to this work

Running title: Neural Inflammation and Cardiopulmonary Afferent Sensitization

*Correspondence to: Hanjun Wang, Department of Anesthesiology, University of Nebraska Medical Center, Omaha, NE, 68198, USA

Tel: 1-402-559-2493, Fax: 1-402-559-4438; Email: hanjunwang@unmc.edu

Disclosure: Drs. Hanjun Wang, Dong Wang, Irving H. Zucker, Steven J. Lisco, and Juan Hong are holding a US patent related to the utility of ProGel-Dex in treating chronic heart failure (US Patent issued No. 12,138,274 B2).

Figure S1

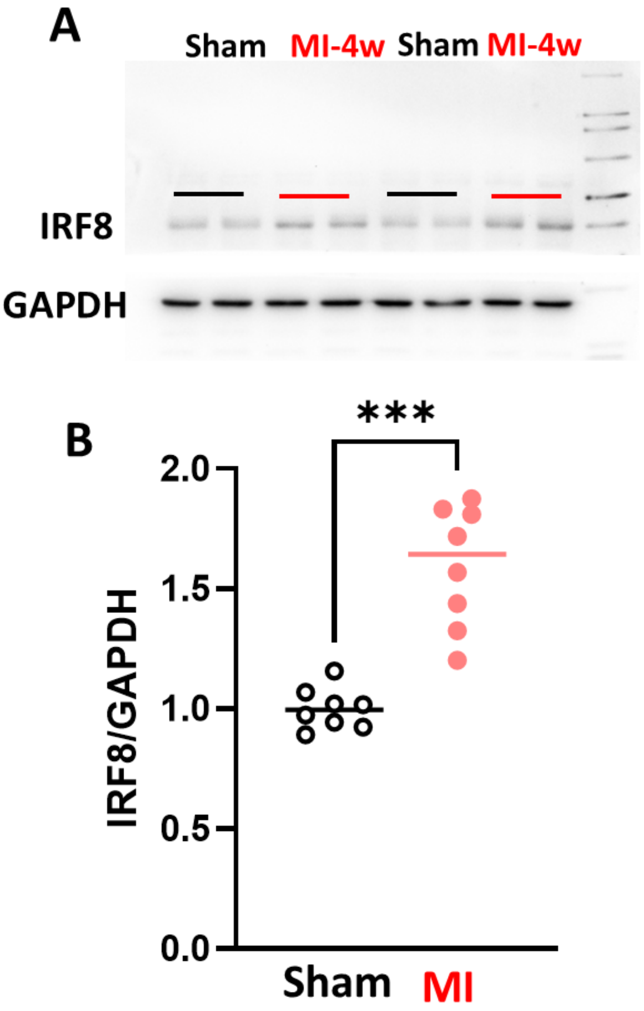

Figure S1. Western blot analysis of IRF8 protein in the T1-T4 DRG of female sham and MI rats at 4 weeks post sham and MI. Values are means ± SD. N=8 each group. *** P<0.001 vs. Sham.

Figure S2

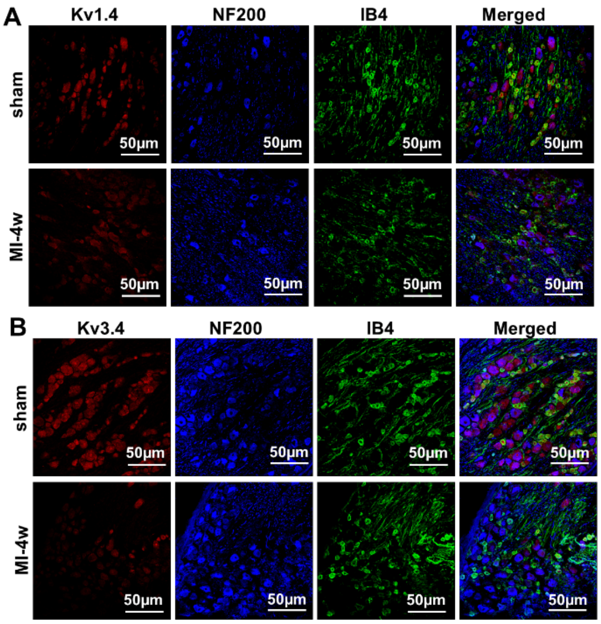

Figure S2. Immunofluorescence straining of Kv channels including Kv1.4, Kv3.4, Kv4.2 and Kv4.3 in T1-T4 DRG between sham and MI rats (**A-D**). Kv channels (red color); Isolectin B4 (IB4, green color), a C-fiber neuron marker; NF200 (blue color), an A-fiber neuronal marker. Scale bar = 50 μm.

Figure S3

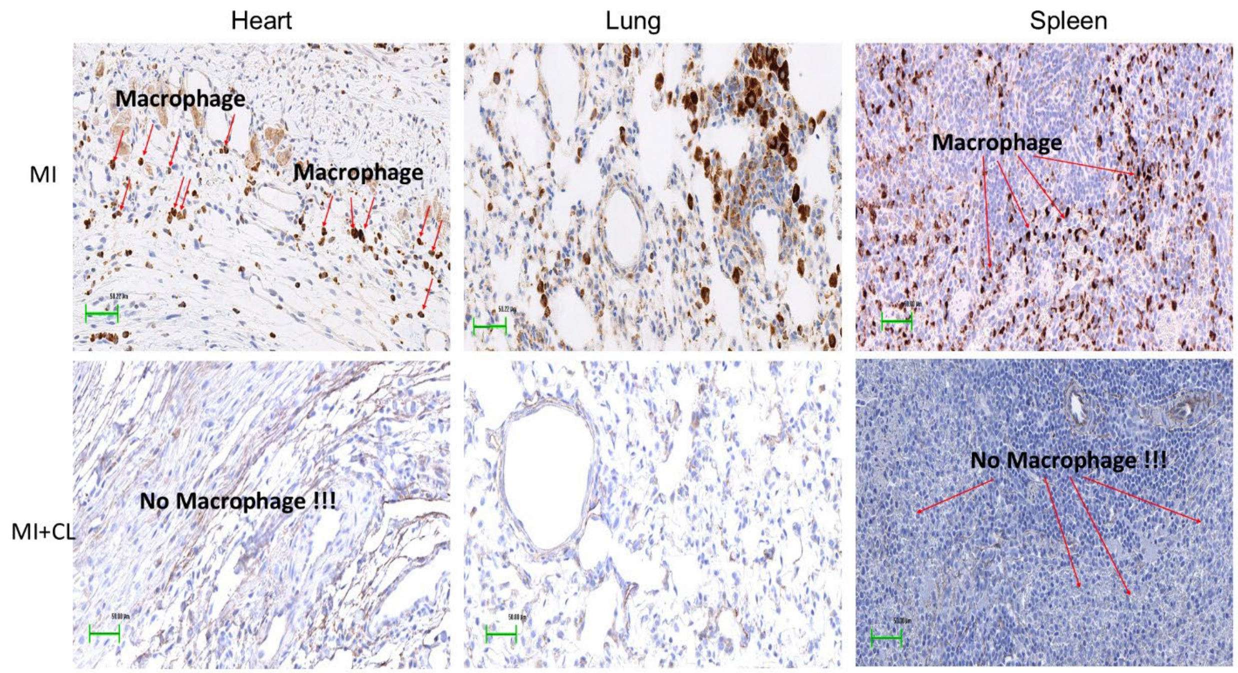

Figure S3. Immunohistological straining of CD68-positive cells in the heart, lungs and spleen in MI rats treated with and without clodronate liposomes (CL).

Figure S4

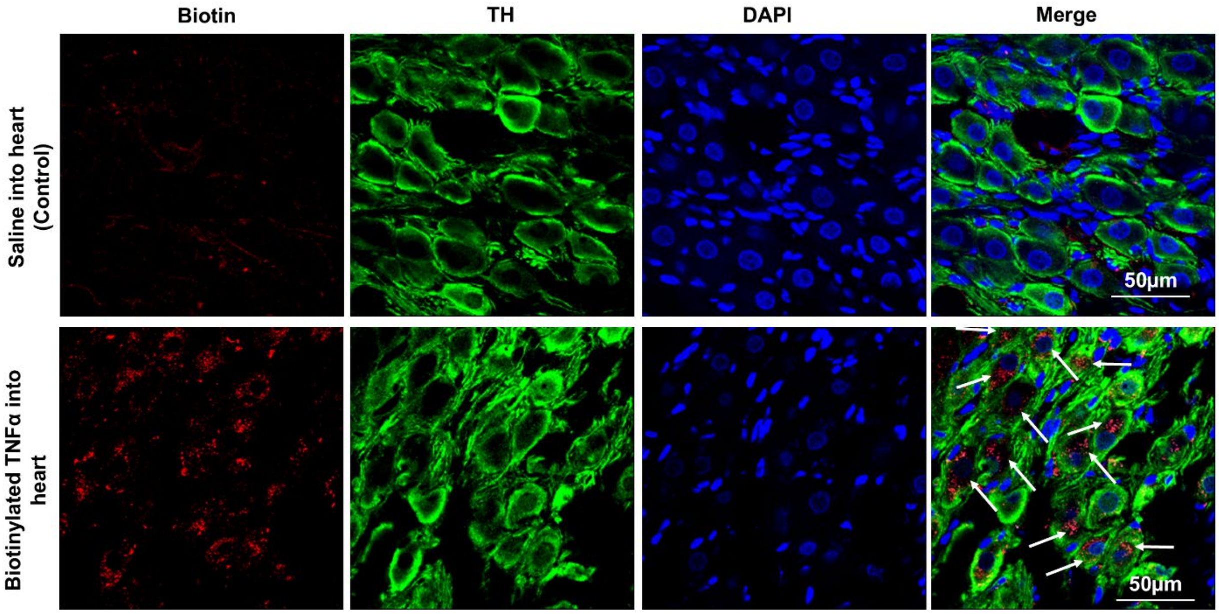

Figure S4. Immunofluorescence staining demonstrating that exogenous biotinylated TNFα in the sub epicardium of the left ventricle in normal rats can be taken up by Tyrosine hydroxylase (TH)-positive cardiac sympathetic efferents and transported back to the stellate ganglia soma.

Figure S5

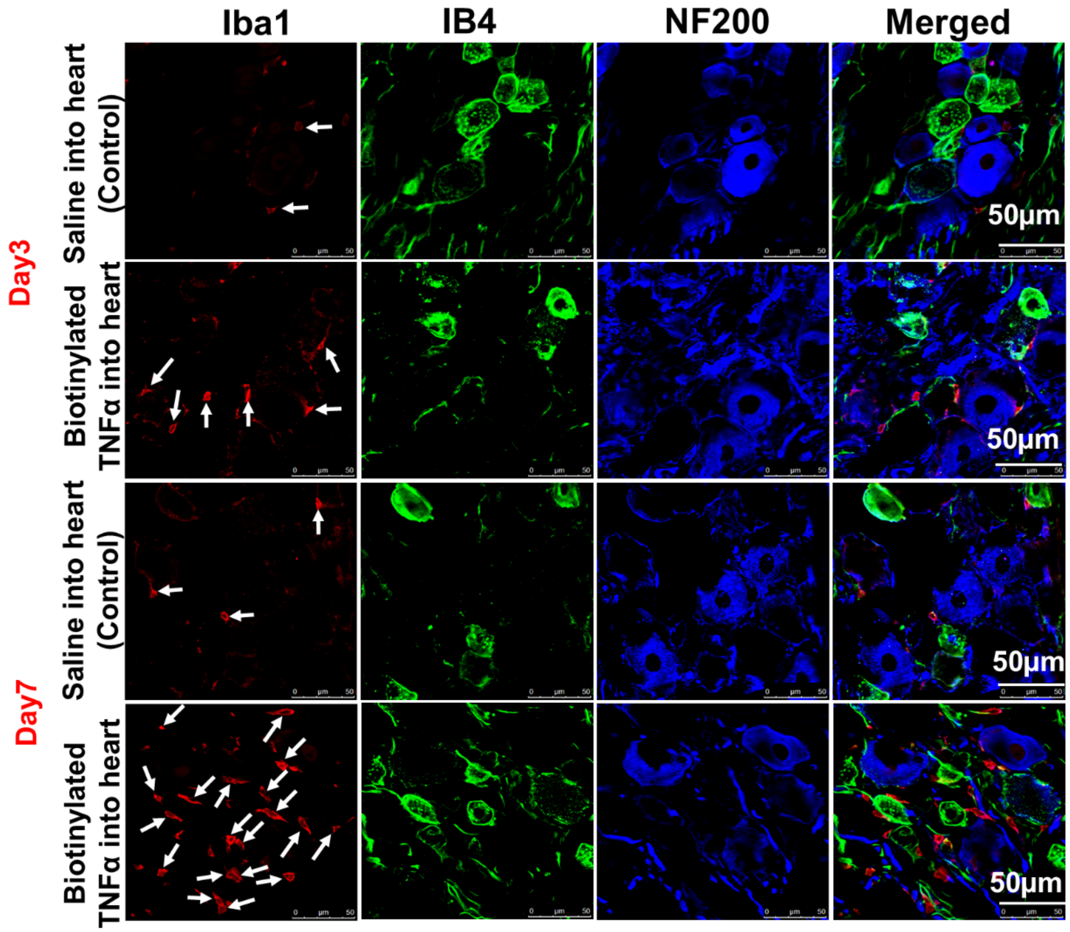

Figure S5. Immunofluorescence staining data demonstrate that injection of exogenous biotinylated TNFα into sub epicardium of the left ventricle caused gradually increased Iba1-positive cells in T1-T4 DRGs from day 3 to day 7 after injection in normal rats. IB4, a C-fiber neuron marker; NF200, an A-fiber neuron marker. White bar =50 μm.

Table S1. The percent of Kv1.4, Kv3.4, Kv4.2 and Kv4.3-positive DRG neurons to IB4- and NF200-positive neurons in T1-T4 DRGs in sham and MI rats

Table 1. The percent of potassium channels (Kv1.4, Kv3.4, Kv4.2 and Kv4.3)-positive DRG neurons to IB4-and NF200-neurons in intact ganglia in sham and CHF rats

	sham		CHF	
	Number	%	Number	%
Total IB4-positive	2534±251		2450±461	
Kv1.4 with IB4	1806±346	70.2±8.1	562±137	23.8±5.5**
Total NF200-positive	2520±331		2379±442	
Kv1.4 with NF200	2184±222	88.2±5.1	897±207	36.8±4.6**
Total IB4-positive	2622±420		2600±348	
Kv3.4 with IB4	2152±314	82.1±4.3	910±217	35.0±5.2**
Total NF200-positive	2428±479		2405±384	
Kv3.4 with NF200	1858±375	76.5±5.5	1332±425	55.4±8.3*
Total IB4-positive	2691±372		2604±474	
Kv4.2 with IB4	2076±240	77.1±5.1	984±207	37.7±4.9**
Total NF200-positive	2576±192		2430±528	
Kv4.2 with NF200	2240±141	86.9±3.3	1176±304	48.3±6.0**
Total IB4-positive	2556±163		2488±231	
Kv4.3 with IB4	2214±144	86.6±5.7	680±168	27.3±5.8**
Total NF200-positive	2362±453		2325±295	
Kv4.3 with NF200	1990±364	84.2±6.2	934±152	40.2±7.7**

Values are Mean±S.E.M. n=5 per group. *P<0.05 and **P<0.01 vs. sham.

Supplemental Video

Sham T1-T4 DRG

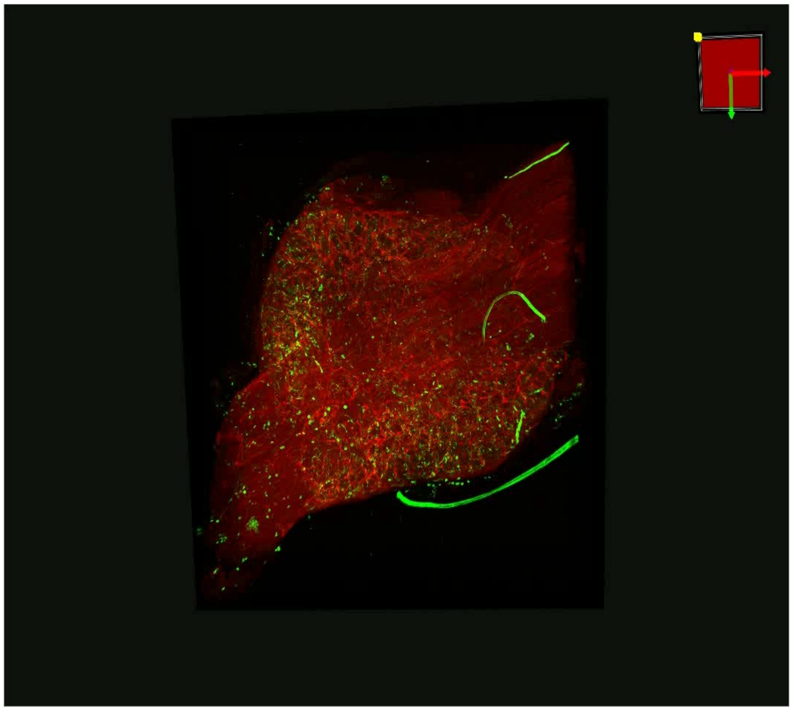

MI T1-T4 DRGs

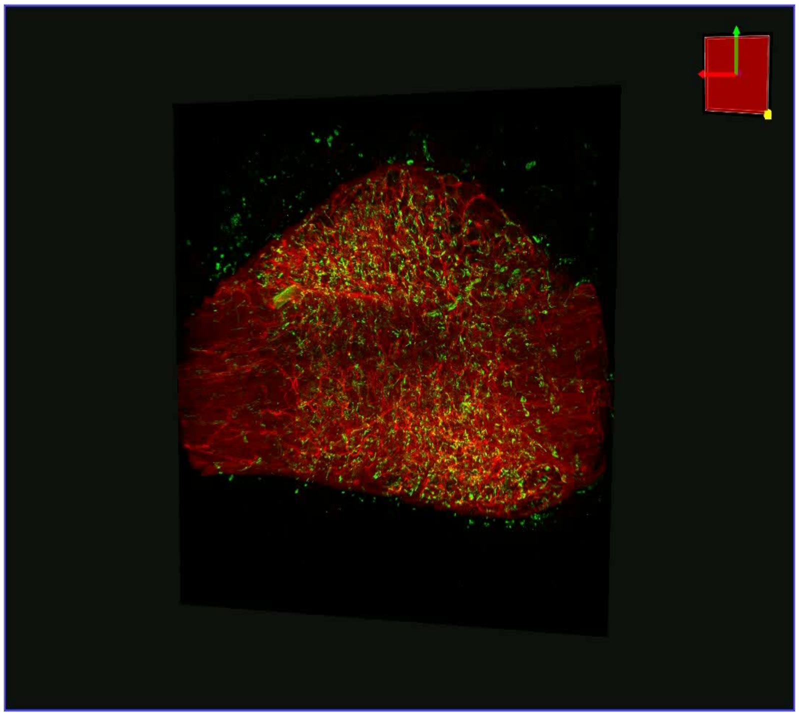

Table S2. Thoracic DRG MI vs Sham mRNA-Seq. All expressed genes.

Gene	FC	log2FC	PValue	FDR
Cst3	1.69874052	0.7644655	7.43E-28	4.39E-24
Necab1	1.45358639	0.539616817	3.03E-11	5.97E-08
Myh11	1.57150815	0.652149758	2.45E-14	9.66E-11
Gpx2	1.51593488	0.600207785	6.19E-12	1.83E-08
Thbs4	0.67676144	-0.563280727	2.31E-10	3.40E-07
Myl9	1.45277314	0.538809437	2.21E-07	1.38E-04
Igfbp3	1.52763667	0.61130146	7.04E-09	7.56E-06
Vgf	0.67322972	-0.570829218	4.67E-07	2.62E-04
Slc6a20	1.42959924	0.515610772	9.77E-06	0.0033923
Acta2	1.7542968	0.810892852	1.36E-11	3.21E-08
Slfn4	1.5848652	0.664360141	2.60E-08	2.36E-05
Myom2	1.39867978	0.484065698	4.73E-05	0.0136129
Anpep	1.44187988	0.527950986	1.28E-05	0.0039761
Elk1	0.57985511	-0.786235644	1.29E-10	2.18E-07
C4b	1.55707195	0.638835612	2.90E-06	0.0012243
Rtn4ip1	1.42078908	0.506692397	2.47E-04	0.0495239
Pcdha7	1.45465914	0.540681138	1.48E-04	0.0332059
Cbln4	1.72729259	0.788512481	1.48E-07	1.09E-04
Acta1	0.6864839	-0.542702212	2.65E-04	0.0504073
Irf7	1.5951439	0.673686577	1.03E-05	0.0034881
Fam111a	0.14177968	-2.818277272	2.12E-51	2.50E-47
Cfp	0.64197603	-0.639408661	1.22E-04	0.0293905
Pla2g2a	1.95536552	0.967438315	1.29E-07	1.09E-04
Pcdhga3	0.62875694	-0.669425673	1.69E-04	0.036983
Lgals5	1.61534417	0.691841585	1.87E-04	0.0401627
Aox3	1.607574	0.684885152	4.79E-04	0.0816015
Smoc1	1.57514624	0.655485775	9.16E-04	0.1424064
RGD1309362	1.84562647	0.884110604	1.11E-05	0.0036253
Prg2	1.9226729	0.943113342	1.17E-05	0.0037483
Cnn1	2.42399331	1.277385718	1.46E-07	1.09E-04
Actg2	2.42589906	1.278519524	1.71E-07	1.12E-04
RT1-CE11	1.83861905	0.878622597	3.42E-04	0.0621292
Epx	1.9679175	0.976669743	4.24E-04	0.0758517
RT1-T24-3	2.09250891	1.065233767	1.49E-04	0.0332059
Myh4	0.4584093	-1.125291797	1.25E-04	0.0295485
Ifit1	3.56932506	1.835651295	4.91E-07	2.64E-04
Fndc7	0.32380424	-1.626806232	5.16E-06	0.0020328
Col11a1	0.71853918	-0.476861273	1.06E-04	0.0271388

Gbp2	1.36978714	0.45395172	8.11E-06	0.0030712
Hspa1b	0.73080028	-0.452450916	4.44E-07	2.62E-04
Ass1	1.35817497	0.441669352	9.75E-06	0.0033923
Hba2	0.74022178	-0.433970513	1.15E-09	1.51E-06
C4a	1.34310512	0.425572222	5.38E-07	2.76E-04
Sfrp2	1.33639946	0.418351304	4.84E-04	0.0816015
Trpa1	0.75747432	-0.400731117	1.67E-07	1.12E-04
Aldh1a2	1.31289276	0.392749077	3.12E-04	0.0576039
Lypd1	1.30874472	0.388183722	5.91E-04	0.0968581
Ctgf	0.76647442	-0.383690456	8.04E-07	3.80E-04
Herpud1	0.7735873	-0.37036399	4.18E-06	0.0017028
Mylk	1.28806181	0.365201826	2.36E-04	0.0480532
Mx2	1.26772699	0.342244093	2.35E-04	0.0480532
Vcl	0.78899961	-0.341903506	5.63E-07	2.77E-04
Gpm6a	0.79231854	-0.335847533	6.55E-05	0.0179966
Ppp1r10	0.79482784	-0.331285688	4.35E-04	0.0766883
Tagln	1.23908272	0.309272507	2.54E-04	0.0499833
Dpysl4	0.81053845	-0.303047471	3.81E-05	0.0112357
Col1a1	0.81345256	-0.297869879	8.32E-06	0.0030712
Mfap4	0.81710776	-0.291401739	1.22E-04	0.0293905
Ddit4	0.82514829	-0.277274678	9.76E-05	0.0256189
Itm2a	0.82703744	-0.273975448	2.63E-04	0.0504073
Basp1	0.82783533	-0.27258428	6.47E-04	0.1046
Col1a2	0.82880391	-0.27089728	6.87E-05	0.0184507
Igfbp6	1.19174774	0.253078885	1.45E-04	0.0332059
Phgdh	0.83911262	-0.253063643	5.11E-04	0.0850186
Scd	0.84423457	-0.24428418	2.10E-04	0.0443635
Serpinh1	0.84672026	-0.240042693	2.73E-04	0.0512234
Clk1	0.85156426	-0.231812697	8.45E-04	0.1348625
Col15a1	0.85401401	-0.22766836	9.03E-04	0.1422239
Coch	2.57927595	1.366966129	4.72E-04	0.0816015
RT1-N2	3.97586685	1.991269442	2.42E-06	0.0010565
Car4	0.32402478	-1.625823955	6.52E-05	0.0179966
Ttr	4.15713445	2.055589408	1.55E-05	0.0047067
Alb	0.15618921	-2.678633334	8.46E-09	8.33E-06
Ear11	3.52355004	1.817029702	1.20E-04	0.0293905
Rnase1l2	0.19776215	-2.338161783	1.60E-06	7.27E-04
Myh6	0.04492928	-4.476200147	5.91E-09	6.98E-06
Per1	0.8167531	-0.292028072	0.00109793	0.1683831
Zdhhc21	0.81899391	-0.288075376	0.00116447	0.1762983

Lss	0.8396468	-0.252145507	0.00132294	0.1977551
Lect1	0.85170327	-0.231577214	0.00142595	0.2082519
Osmr	1.35236463	0.435484188	0.00142844	0.2082519
C1qtnf1	0.77031053	-0.376487945	0.00146154	0.2093046
Cartpt	0.41957286	-1.25300672	0.0014757	0.2093046
Pcp4l1	0.78827452	-0.343229948	0.00148883	0.2093046
Anxa6	1.16321798	0.21812147	0.00151285	0.2101795
Plau	1.53556437	0.618768987	0.00153541	0.210833
Kcnh6	0.77868715	-0.360884279	0.00163404	0.2217973
Nfkbia	0.82272074	-0.281525282	0.00166265	0.2231165
Kcnj8	2.11256476	1.07899557	0.00170151	0.2257653
Art2b	0.40026627	-1.320968032	0.00174452	0.2289
Sphk1	0.76974257	-0.377552065	0.00176591	0.2289798
Pcdhgb8	1.46162653	0.547574721	0.00178391	0.2289798
Sv2a	0.81386687	-0.297135267	0.00181874	0.2309405
Oas2	2.50135267	1.322708477	0.0018601	0.2336806
Chad	0.5523622	-0.856313511	0.00195322	0.2427954
Mmp13	0.51479704	-0.95792433	0.00208531	0.2565144
Zcchc9	0.81409335	-0.296733856	0.00214968	0.2617064
Usp18	1.71982673	0.782263226	0.00219027	0.2621344
Fos	0.76794025	-0.380934034	0.00219759	0.2621344
Cacna2d2	0.79807704	-0.325400068	0.00228933	0.2703464
Chrne	0.74538768	-0.423937122	0.00234556	0.2730769
Lyz2	1.14951564	0.201026099	0.00239606	0.2730769
Cyr61	1.24312094	0.313966661	0.00239655	0.2730769
Steap4	1.22186781	0.289088207	0.00240494	0.2730769
Npy1r	0.8309899	-0.267097157	0.00257043	0.2871258
Fabp4	1.74633794	0.804332771	0.0025773	0.2871258
C2	1.24514114	0.316309287	0.00265404	0.2929119
Fasn	0.87063559	-0.199859103	0.00268835	0.2939508
Il2rg	0.67379529	-0.569617746	0.00275959	0.2970162
Nqo1	1.19987203	0.262880552	0.00276668	0.2970162
Sostdc1	0.83405496	-0.261785648	0.00284243	0.3011822
RT1-A1	1.27047038	0.345362741	0.0028565	0.3011822
Cd163	1.28303602	0.359561675	0.00290886	0.3039883
Col3a1	0.86682717	-0.206183727	0.00301281	0.3120901
Xdh	1.23186604	0.300845377	0.00308556	0.3161321
Mri1	1.31658647	0.396802276	0.00311828	0.3161321
Mbp	0.86588188	-0.207757869	0.00317067	0.3161321
C3	1.39935969	0.484766842	0.00317654	0.3161321

Krt12	0.17782466	-2.491472727	0.00318568	0.3161321
Scn11a	0.87185769	-0.197835425	0.00326749	0.3215482
Tpbg	0.57244311	-0.804795778	0.00330162	0.3222218
Ccr5	1.50383792	0.58864909	0.00345202	0.3341386
Tmem87b	1.2225127	0.289849457	0.00349783	0.3358205
Crabp1	0.60955947	-0.714161103	0.00354042	0.3371682
Rn45s	1.54097248	0.623841096	0.0036257	0.3425274
Fkbp1a	0.87419178	-0.193978281	0.003659	0.3429294
Mrgprx1	1.59823717	0.676481517	0.0038173	0.3549486
Pygb	1.14880891	0.200138844	0.00389341	0.3564187
Top2a	0.64819287	-0.625504938	0.00389347	0.3564187
Mpp2	0.70637326	-0.501497359	0.00396739	0.360392
Larp6	1.28475694	0.361495443	0.00400572	0.3610954
Fkbp5	0.84695603	-0.23964102	0.0040382	0.361266
Txnip	1.14101711	0.190320425	0.00407634	0.3619364
Pi15	0.53797084	-0.894400125	0.00424938	0.3742142
Map3k11	0.81741972	-0.290851046	0.004278	0.3742142
P2rx4	1.2332484	0.302463414	0.00437146	0.379578
Lrrc4b	0.82331877	-0.280476979	0.00443783	0.3825277
Cd36	1.271248	0.346245505	0.00449859	0.3849551
Per2	0.82832849	-0.271725093	0.00454259	0.3859244
C1qb	1.222556	0.289900552	0.00479541	0.4044931
Slc4a1	1.30603181	0.385190032	0.00485914	0.4045826
Pmp2	1.14135213	0.190743958	0.00489223	0.4045826
Wee1	0.83877609	-0.253642357	0.00489926	0.4045826
Adamts1	1.19918551	0.262054853	0.00494296	0.4053574
Tenm3	0.85409935	-0.227524206	0.00498537	0.4060151
Slc16a11	1.17303671	0.230248167	0.00506673	0.4070793
Zer1	0.84801568	-0.237837162	0.0050777	0.4070793
Slc3a1	0.42923878	-1.220147655	0.00512818	0.4070793
Capn5	0.87827462	-0.187255984	0.00513632	0.4070793
Rspo1	1.91168498	0.934844803	0.00529174	0.4161276
Cxcl13	1.48996888	0.575282199	0.00532096	0.4161276
Tpm2	1.22881388	0.297266415	0.00538301	0.4182101
Net1	1.18169595	0.240858881	0.00561093	0.4330683
Prim1	0.81735688	-0.290961958	0.00565149	0.4333665
Inmt	1.4056888	0.491277235	0.00571283	0.434137
Slc13a4	1.27045206	0.345341934	0.00575652	0.434137
Abhd4	0.8788821	-0.186258454	0.00581926	0.434137
Spp1	1.14206816	0.191648755	0.005837	0.434137

Tnfrsf25	0.61362785	-0.704564137	0.00584535	0.434137
Dnm1	0.88106224	-0.182684156	0.00599589	0.4366669
Celsr2	0.84319696	-0.246058434	0.00600091	0.4366669
Ifi27l2b	1.14453118	0.194756772	0.00602148	0.4366669
Dmbt1	1.33103623	0.412549841	0.00602733	0.4366669
Mcmdc2	1.57414991	0.654572942	0.00612369	0.4387971
Nptxr	0.86802808	-0.204186382	0.00615223	0.4387971
Fbxl4	1.22284263	0.290238753	0.0061682	0.4387971
Oaf	0.84813075	-0.237641407	0.0062518	0.4420807
Ptgds	1.14079421	0.190038564	0.00631676	0.4440157
Myl1	0.62125515	-0.686742178	0.00643865	0.4499054
Cybb	1.34621144	0.428905021	0.00663354	0.460797
Mmd2	0.8707522	-0.199665889	0.00677964	0.4674423
Gstm2	1.20936142	0.274245466	0.00680837	0.4674423
Ramp3	0.78110903	-0.356404149	0.00696944	0.4742751
Mrgprc	0.6493126	-0.623014892	0.00698822	0.4742751
Serping1	1.13538471	0.183181218	0.00703116	0.474463
Plcb3	0.88065077	-0.183358075	0.00732742	0.4916449
C1r	1.14278101	0.192548974	0.00737142	0.491803
Ncam2	0.8121737	-0.300139787	0.00753502	0.4946294
Sfrp4	1.20879454	0.273569053	0.00755983	0.4946294
Ddah1	0.86200358	-0.214234232	0.00757769	0.4946294
Scara5	1.15921015	0.213142128	0.00758133	0.4946294
Pi16	1.14041887	0.18956382	0.00776276	0.5036835
Ankrd9	0.75015368	-0.414741915	0.00783128	0.5053529
Nov	1.21639515	0.282611965	0.00811747	0.5206653
Otof	0.75865187	-0.39849009	0.00818535	0.5206653
Frzb	1.39990888	0.485332922	0.00820084	0.5206653
Tril	0.84455296	-0.2437402	0.00841115	0.5294645
Acsl5	1.17851496	0.236970076	0.00842911	0.5294645
Cfd	1.41477146	0.500569017	0.00849066	0.5305089
RT1-DOb	2.49132793	1.316914934	0.00861647	0.5317813
Rab7b	0.75514174	-0.405180641	0.00862217	0.5317813
Eif2ak2	1.18755458	0.247993818	0.00864612	0.5317813
Cd247	0.48002326	-1.058823788	0.00878015	0.5372268
Mras	0.85260375	-0.230052694	0.00882769	0.5373517
Ubash3b	0.87780381	-0.188029556	0.00893506	0.5410979
Sdc3	0.88648293	-0.173835237	0.00908115	0.5448184
Mvd	0.83339361	-0.262930064	0.00908877	0.5448184
Pcolce	0.86315421	-0.212309763	0.00917296	0.5453825

Col9a2	0.74809677	-0.418703187	0.00919054	0.5453825
Ednrb	0.85634293	-0.223739437	0.00928942	0.5476468
Map3k7cl	1.99371734	0.995460882	0.00932145	0.5476468
Srd5a2	10.4835148	3.390050586	0.00945476	0.5527289
Elp6	1.23521078	0.304757252	0.00959036	0.5571745
Tp53i13	0.70112966	-0.512246819	0.00962517	0.5571745
Ghdc	0.76248027	-0.391228085	0.01005354	0.5791327
Col4a1	0.88793117	-0.171480254	0.01013926	0.5812355
Aatk	0.8894632	-0.168993179	0.01038675	0.5908462
Sult1a1	1.15852433	0.212288341	0.01040698	0.5908462
Prlr	2.07860145	1.055613162	0.0106687	0.5953581
Dlgap3	0.84591294	-0.241418902	0.01069395	0.5953581
Gpx1	1.13883651	0.187560651	0.01070439	0.5953581
Rin2	1.19051703	0.251588258	0.01070787	0.5953581
Tec	1.27481062	0.35028294	0.01082667	0.5953581
Fam221a	0.50835628	-0.976088148	0.01082708	0.5953581
Ankrd6	0.82059899	-0.285250714	0.01085036	0.5953581
Snx14	0.84895898	-0.236233251	0.01093419	0.5953581
Rit2	1.14630388	0.196989542	0.01094019	0.5953581
LOC360231	1.57603444	0.656299058	0.01106913	0.5976639
Slc27a4	0.85718862	-0.222315395	0.01112299	0.5976639
Sgk1	0.86072143	-0.216381698	0.01117309	0.5976639
Dnase1l3	2.02170702	1.015573941	0.01122969	0.5976639
RT1-S2	1.69907536	0.764749839	0.01123562	0.5976639
Siglec1	1.57031539	0.651054348	0.01133165	0.6000691
Ptms	0.89045984	-0.167377554	0.01146255	0.6039076
Cd53	1.2350187	0.304532891	0.01150641	0.6039076
Ackr2	1.60198154	0.67985752	0.01170793	0.6117654
Dagla	0.87295235	-0.196025182	0.0117607	0.6118156
Npr3	0.66254359	-0.59391273	0.01196399	0.6173367
Rtp4	1.47507388	0.560787214	0.01199845	0.6173367
Fbxo41	0.77306497	-0.371338431	0.01210453	0.6173367
Cd4	1.24304214	0.313875209	0.01212594	0.6173367
Hmgcr	0.89042978	-0.167426246	0.0121465	0.6173367
Ctnnd2	0.85327272	-0.228921168	0.01221696	0.6173367
Cnp	0.89132498	-0.165976551	0.01223277	0.6173367
Arsb	0.84468341	-0.24351738	0.01239548	0.6212965
Usp2	0.6911598	-0.532908789	0.01241646	0.6212965
Lrif1	1.20146786	0.264798053	0.01272368	0.6330313
Orai1	1.29265386	0.37033601	0.01280817	0.6330313

Agps	0.83325256	-0.263174247	0.01288006	0.6330313
Mpeg1	1.18960057	0.250477247	0.01290181	0.6330313
Slitrk2	0.81493609	-0.295241163	0.01291901	0.6330313
Nid2	0.87076115	-0.199651054	0.01297988	0.6333859
Cx3cr1	1.69990328	0.765452663	0.01310929	0.6370685
RGD1305938	0.87455644	-0.193376596	0.01327099	0.6422833
Madd	0.88838287	-0.170746518	0.0135817	0.6528714
Slfn2	1.26325813	0.33714947	0.01367086	0.6528714
Olfml3	0.84502887	-0.242927459	0.0136984	0.6528714
B3galt5	1.21898597	0.285681517	0.01372177	0.6528714
Scn10a	0.89158975	-0.165548062	0.01383771	0.6528714
Cd180	1.75029383	0.80759713	0.01386415	0.6528714
Slc4a4	0.8566972	-0.223142728	0.01387768	0.6528714
Mboat2	0.87214057	-0.197367412	0.01401061	0.6528714
Car1	1.81132034	0.857041716	0.01401508	0.6528714
Pclo	0.89064514	-0.167077366	0.01404262	0.6528714
Dnajb1	0.87662215	-0.189972957	0.01421491	0.6544204
LOC498675	0.53972434	-0.889705356	0.01422682	0.6544204
Fut7	0.58206405	-0.780750168	0.01424998	0.6544204
Rdm1	1.47868674	0.564316454	0.01429761	0.6544204
Tmem132a	0.83945986	-0.252466753	0.01448472	0.6563146
Tlr3	1.28443028	0.36112858	0.01454855	0.6563146
Fadd	2.07019444	1.049766275	0.01455577	0.6563146
F13a1	1.21581822	0.281927546	0.01460541	0.6563146
Myof	1.1835479	0.243118092	0.01461688	0.6563146
Bst2	1.24059643	0.311033883	0.01529473	0.6806416
Tanc1	0.88057707	-0.183478814	0.01532469	0.6806416
Ahsa2	0.82587788	-0.27599962	0.01535031	0.6806416
Tbx18	1.34553484	0.428179745	0.01538922	0.6806416
Pecam1	1.23016861	0.298856067	0.01547132	0.6817194
RT1-N3	1.49814928	0.583181386	0.01555188	0.6827219
Ly6e	1.1502402	0.201935162	0.01569516	0.6864597
Osr1	1.34477773	0.427367742	0.01595791	0.6953762
Tbkbp1	0.81162332	-0.301117775	0.0160697	0.6969059
Phf11b	2.27258372	1.184333443	0.01614299	0.6969059
Lnc215	1.17026145	0.226830879	0.01617006	0.6969059
Kcnc4	0.86591	-0.207711008	0.0163209	0.7008491
Peak1	0.87443881	-0.193570666	0.0165737	0.7066856
Ebf2	1.1990942	0.261945005	0.0165765	0.7066856
Adam19	0.89194842	-0.164967808	0.01682216	0.7145785

Birc3	1.75517221	0.811612586	0.0169494	0.7150672
Ptpdc1	0.85065889	-0.233347361	0.01695476	0.7150672
Cilp	1.25202464	0.324262951	0.01713653	0.7201609
Pex12	1.25191682	0.324138708	0.01731245	0.7249741
C1s	1.11835778	0.161381799	0.01739214	0.725046
St6galnac5	0.64311884	-0.636842751	0.01748474	0.725046
Ntrk2	0.8970279	-0.156775238	0.0175556	0.725046
Ism1	1.65193367	0.724155757	0.01763364	0.725046
Dapp1	1.52713009	0.610822961	0.0176865	0.725046
Fxyd1	1.13754514	0.185923792	0.017744	0.725046
Lrat	1.78600363	0.836735016	0.01780145	0.725046
Gpd1	0.85158077	-0.231784717	0.01781771	0.725046
Mrps35	1.17691639	0.235011837	0.01788161	0.725046
Crot	0.88783729	-0.171632784	0.01792814	0.725046
Sparcl1	1.11675193	0.159308752	0.01807683	0.7285641
Pnpla1	1.39846836	0.48384761	0.01849413	0.7412579
Tagap	0.66291519	-0.593103787	0.01852108	0.7412579
Ncs1	0.86621969	-0.207195131	0.0185866	0.7412579
Vtn	1.18108014	0.240106858	0.01868906	0.7412579
Smpx	1.27818151	0.354092728	0.01871705	0.7412579
Alpl	0.85424343	-0.227280847	0.01877752	0.7412579
Gstm7	1.1535901	0.206130684	0.01883118	0.7412579
Cep104	0.87292509	-0.196070234	0.01895927	0.7414275
Lrfn4	0.81064667	-0.302854866	0.01898398	0.7414275
Gucy1a3	1.31187815	0.391633729	0.01902398	0.7414275
Ankrd52	0.87856294	-0.186782448	0.01908662	0.7414275
Prex1	0.87206403	-0.197494035	0.01918804	0.7429231
Fbxl16	0.82835853	-0.271672771	0.01942244	0.7495412
Slc16a10	1.96484869	0.97441822	0.01960815	0.7520753
Acad10	1.36021621	0.443835992	0.01961548	0.7520753
Uba7	1.44109902	0.527169472	0.01988592	0.7599767
Tf	1.12496652	0.169882062	0.02005124	0.763823
Spib	0.65349915	-0.613742736	0.02012485	0.7641619
Nrp2	0.87627053	-0.190551749	0.02039013	0.7717532
Rgs8	0.69916648	-0.516292076	0.02049349	0.7731872
Gsk3a	0.88272317	-0.179967032	0.02065198	0.7766854
Col11a2	0.75229033	-0.410638542	0.02075149	0.7779503
Cdo1	1.16894956	0.225212674	0.02088049	0.7803093
Gsn	1.11809272	0.161039833	0.02103274	0.7833953
Mmp2	0.89535357	-0.159470592	0.02109575	0.7833953

Kcna6	0.88660819	-0.173631406	0.02153882	0.7973415
Akr1b8	1.7114602	0.775227747	0.02168554	0.7989878
Nox4	1.69711314	0.763082747	0.02171861	0.7989878
Taf1a	0.69932991	-0.51595489	0.02187284	0.8008687
Itgam	1.40968223	0.495369983	0.02190538	0.8008687
Phka2	0.65388249	-0.612896711	0.02218621	0.802256
Zbtb3	1.58458004	0.664100535	0.02231921	0.802256
Sf1	0.88506633	-0.176142516	0.0223432	0.802256
St6galnac3	0.72547076	-0.46301062	0.02246629	0.802256
Samsn1	1.83155106	0.873065923	0.0225754	0.802256
Map3k2	0.71638207	-0.481198872	0.02262346	0.802256
Fads2	0.88878064	-0.170100704	0.0226433	0.802256
Ces1d	1.5017142	0.586610267	0.02265096	0.802256
Arhgef7	0.87998866	-0.184443166	0.02265156	0.802256
Cacng5	0.69474808	-0.525438163	0.02267607	0.802256
Kctd7	0.76448641	-0.38743724	0.02274137	0.802256
Nrap	0.42288961	-1.241646995	0.022799	0.802256
Rab36	0.75858907	-0.398609503	0.0228628	0.802256
Usp33	1.1116205	0.152664346	0.02289443	0.802256
Cntn2	0.90097015	-0.150448788	0.02340113	0.8083752
Zfp746	0.79784957	-0.325811332	0.02342121	0.8083752
Prr7	0.64197328	-0.639414852	0.02348631	0.8083752
Sh3bp1	1.50733569	0.592000749	0.02354222	0.8083752
Csrnp2	1.20454077	0.268483221	0.02357813	0.8083752
Itga8	1.28847189	0.365661063	0.02361111	0.8083752
Mib1	0.87026671	-0.200470492	0.02362012	0.8083752
Il17rc	1.40775251	0.493393719	0.02365172	0.8083752
Hmg1l1	1.18949961	0.250354797	0.02372577	0.8083752
Map4k3	0.86953923	-0.201676979	0.02375359	0.8083752
RT1-CE10	1.17746426	0.235683269	0.02386504	0.8098341
Dcdc2	1.41672165	0.502556338	0.02410394	0.815597
Pank3	0.90189944	-0.148961505	0.02436931	0.8222207
Phlda3	0.88749621	-0.172187139	0.02450441	0.8244233
C6	1.45948755	0.545461901	0.02476968	0.8300439
Exosc7	1.3738929	0.458269547	0.02481205	0.8300439
Cfb	1.2507994	0.322850429	0.02497037	0.8329806
Cd3e	0.56962227	-0.81192255	0.02508127	0.8343233
Hemgn	1.41455863	0.500351972	0.02523834	0.8353303
Srsf5	1.1115635	0.152590371	0.02525302	0.8353303
Prg3	1.8426948	0.881817144	0.02541393	0.8383048

Mmgt2	1.36340416	0.447213291	0.02556851	0.8403299
Ehd4	1.13549113	0.183316442	0.02561765	0.8403299
Acss1	1.20818992	0.27284725	0.02585633	0.8437987
Atp13a2	0.90050024	-0.15120144	0.02592163	0.8437987
Cpxm1	0.77787767	-0.362384802	0.02593775	0.8437987
Tmem184b	0.89658492	-0.157487858	0.02607917	0.8458398
Disp2	0.90082609	-0.150679487	0.02614375	0.8458398
Wipf3	0.88328934	-0.179041997	0.02647034	0.8537316
Rarres2	0.87611879	-0.190801599	0.02653226	0.8537316
Pfkl	0.87289231	-0.196124424	0.02667877	0.856113
Set	0.88895716	-0.169814197	0.02684568	0.8566906
Elmo3	1.5245882	0.608419616	0.02691287	0.8566906
Spn	1.39751725	0.482866086	0.02691441	0.8566906
Taf6l	1.41477528	0.500572916	0.02699832	0.8567516
Ly6g6e	0.62246991	-0.683923992	0.02715502	0.8567516
Oprm1	0.82186455	-0.28302745	0.02731905	0.8567516
Ltbp3	0.89650781	-0.157611943	0.02733098	0.8567516
Arap2	1.13131306	0.177998216	0.02744572	0.8567516
Ndst1	0.85591092	-0.224467437	0.0274972	0.8567516
Nacc1	0.86039377	-0.216931016	0.02750402	0.8567516
Hspa1a	0.75484815	-0.405741645	0.02756053	0.8567516
Dhrs3	1.21366032	0.279364698	0.02769059	0.8567516
Fam150b	0.52379204	-0.932933959	0.02769425	0.8567516
Eng	1.15166246	0.20371794	0.02782625	0.8567516
Ugt8	0.9038681	-0.14581584	0.02783988	0.8567516
Cpxm2	0.90090436	-0.15055413	0.02804589	0.8567516
Cyp2s1	0.84284848	-0.246654803	0.0281579	0.8567516
Fggy	0.77222249	-0.372911527	0.02817134	0.8567516
Galr2	0.7799375	-0.358569585	0.028272	0.8567516
Prss35	1.36986534	0.454034084	0.02831223	0.8567516
Bin2	1.489777	0.575096392	0.02839896	0.8567516
Fam89a	0.83136322	-0.266449164	0.02840972	0.8567516
RGD1565059	1.38237576	0.467149827	0.02841491	0.8567516
Rag1	0.57810843	-0.790587989	0.02843989	0.8567516
Ptprz1	0.89980856	-0.152310002	0.02851891	0.8569459
Fndc5	0.86115801	-0.215650124	0.02875908	0.859499
Vipr2	0.78224517	-0.354307247	0.02888524	0.859499
Mab21l1	0.75945295	-0.396967508	0.02890566	0.859499
Slc2a12	1.36281186	0.446586412	0.02892749	0.859499
Tfpi2	1.85105607	0.888348597	0.02896779	0.859499

Nupr2	1.54887508	0.631220789	0.02931619	0.8676565
Map7d3	1.23069734	0.299476013	0.02965907	0.87561
Sema5a	0.90321801	-0.146853844	0.0299188	0.881075
Tph1	0.74372757	-0.427153834	0.03021924	0.8814563
Slc9a3r1	0.88781066	-0.17167606	0.0302228	0.8814563
Mdfi	0.53735025	-0.896065349	0.03022688	0.8814563
LOC24906	1.65135074	0.723646579	0.03036701	0.8814563
Abca2	0.90588456	-0.142600884	0.03039343	0.8814563
Spta1	1.46140334	0.547354406	0.03042764	0.8814563
Zfyve28	0.88657492	-0.173685551	0.03049061	0.8814563
Cd47	0.88337957	-0.178894619	0.03052889	0.8814563
Fgfr4	0.65269701	-0.615514667	0.0306574	0.883008
Adipoq	1.65655205	0.728183532	0.03078639	0.8830421
Pcdhgc3	0.88513309	-0.176033691	0.03088243	0.8830421
Nrgn	1.77889479	0.830981183	0.03088292	0.8830421
RGD1359290	1.14770507	0.198751956	0.03105314	0.8840758
Jph4	0.86140778	-0.215231736	0.0310688	0.8840758
Drgx	0.88779301	-0.171704752	0.03116982	0.884525
Fcgr2a	1.48163459	0.567189689	0.03125975	0.884525
Pou2af1	0.63820809	-0.647901209	0.03130929	0.884525
Pla2g7	1.11792836	0.160827743	0.03159524	0.8904731
Igfbp2	0.86835162	-0.203648742	0.03192236	0.8931451
Ckm	0.72235375	-0.469222571	0.03201687	0.8931451
Cd302	1.16550936	0.220960597	0.03203089	0.8931451
Ppp2r5b	0.90239353	-0.148171371	0.03203644	0.8931451
Atp13a5	1.60722573	0.684572561	0.032174	0.8931451
Rufy1	0.84386505	-0.244915785	0.03219795	0.8931451
Kbtbd11	0.83206912	-0.265224721	0.03221948	0.8931451
Ciart	0.76068903	-0.394621293	0.0323843	0.8956116
Mis18bp1	1.30281853	0.38163615	0.03252479	0.8966152
Ece1	0.88528425	-0.175787341	0.03257244	0.8966152
Mecom	1.36242955	0.446181631	0.03271913	0.8985587
Ifit3	1.42012116	0.506014023	0.03283406	0.8988568
Zc3h12d	1.61980342	0.695818734	0.03299436	0.8988568
Rad54l2	0.84281855	-0.246706022	0.03308736	0.8988568
Parvg	1.4938581	0.579043117	0.03313585	0.8988568
Sat1	1.11722586	0.159920867	0.03318283	0.8988568
Zfp498	0.72372018	-0.466496101	0.03318669	0.8988568
Ccnb2	0.64308076	-0.636928177	0.0333308	0.9004595
Tlr2	1.38532213	0.470221485	0.03339836	0.9004595

Nckap5l	0.85852914	-0.220060985	0.03356753	0.902959
Sell	0.71537814	-0.483222067	0.03370857	0.9046921
Crk	0.88018566	-0.184120227	0.03396385	0.9094763
Ifi47	1.30086904	0.379475735	0.03406528	0.9101287
Zfp870	1.47045709	0.55626468	0.03421271	0.9120044
Lamp5	1.18414307	0.243843401	0.03429226	0.9120659
Cp	1.12087875	0.164630227	0.03446221	0.9126916
Postn	0.82905286	-0.270464001	0.03461553	0.9126916
Ung	1.49508732	0.58022975	0.03462231	0.9126916
Kcnj13	1.31508157	0.395152288	0.03464361	0.9126916
Gfra2	0.89203011	-0.16483569	0.03470222	0.9126916
Nup210	0.74393734	-0.426746983	0.03493776	0.9164648
Pde11a	0.75133208	-0.41247739	0.03506619	0.9164648
Eif3k	1.16310084	0.217976183	0.03507851	0.9164648
Pnpla7	1.19755744	0.260094858	0.03519494	0.9174769
Dmgdh	0.76027333	-0.395409913	0.03540843	0.921009
Igsf3	0.90042762	-0.151317786	0.03556089	0.9229419
Wsb2	0.9046386	-0.144586537	0.03593198	0.927265
Prdm9	0.62895163	-0.668979034	0.03594626	0.927265
Tlr6	1.49372741	0.578916897	0.03596302	0.927265
Clec14a	1.23444206	0.303859127	0.03620668	0.9281395
MGC116121	1.27932703	0.3553851	0.03626363	0.9281395
Rps25	0.90474438	-0.14441785	0.036289	0.9281395
Cacna1b	0.90317986	-0.146914777	0.03631133	0.9281395
Synpo2	1.27544102	0.350996189	0.03645168	0.9287141
Coq3	1.17115313	0.227929719	0.0364911	0.9287141
Pamr1	1.48184504	0.567394591	0.03682027	0.9350765
Aaed1	1.50433461	0.5891255	0.03695941	0.9365958
Itgad	2.63607185	1.398389696	0.03712052	0.9382806
Unc13b	0.79418255	-0.332457433	0.03725493	0.9382806
Hgs	0.90120706	-0.150069472	0.03730196	0.9382806
Rhob	0.90551043	-0.14319683	0.03736799	0.9382806
Ina	0.89627149	-0.157992288	0.03743449	0.9382806
Zfp763	1.35476619	0.438043888	0.03750262	0.9382806
Il20rb	1.64684399	0.719703887	0.03778385	0.9433181
Dyrk1b	0.82639764	-0.275091962	0.03809583	0.9447488
Bloc1s6	1.18920341	0.24999551	0.0381116	0.9447488
Cacnb3	0.9092166	-0.137304068	0.03812717	0.9447488
Rbfox3	0.86301571	-0.212541274	0.03816116	0.9447488
Gpr146	0.80853399	-0.306619666	0.03835365	0.9464532

Cyth4	1.264827	0.338940071	0.0383903	0.9464532
Bcl10	0.85744317	-0.221887048	0.03849214	0.9469868
Lat	0.60716128	-0.71984831	0.03890935	0.953255
Hhex	1.66668398	0.736980581	0.03897196	0.953255
Cdnf	1.55178511	0.633928792	0.03898909	0.953255
Alkbh3	1.11462003	0.156551983	0.03915137	0.9537669
Scrt2	0.77767788	-0.362755393	0.03917156	0.9537669
Plod1	0.89386389	-0.161872924	0.03927453	0.9543063
Adgrg1	0.90862521	-0.138242756	0.0394713	0.9571183
Med25	0.86348138	-0.211763028	0.03957306	0.9576193
Bnip3	1.09967753	0.137080525	0.03971935	0.9578221
C3ar1	1.64019504	0.713867377	0.03974366	0.9578221
Mrps28	0.64497617	-0.632682229	0.04004146	0.9630338
Ifitm3	1.13282388	0.179923588	0.04027686	0.9634059
Rbl2	1.13517474	0.182914392	0.04046228	0.9634059
Aif1	1.23049904	0.299243529	0.04060616	0.9634059
Igtp	1.28685432	0.363848744	0.04065944	0.9634059
Ckap4	0.87181779	-0.19790145	0.0407195	0.9634059
Mpz	0.90266432	-0.147738507	0.04079265	0.9634059
Was	1.56172807	0.643143275	0.04100593	0.9634059
Mcpt8	1.48066288	0.566243201	0.04101668	0.9634059
Car14	0.61769514	-0.695033126	0.04106625	0.9634059
Pcdhac2	0.90813046	-0.139028527	0.04116138	0.9634059
Map1s	0.86470735	-0.209716135	0.04120193	0.9634059
Natd1	0.83694917	-0.256788091	0.04120694	0.9634059
Irgm	1.35605073	0.439411153	0.0412292	0.9634059
Fam213a	0.84249078	-0.247267192	0.04124179	0.9634059
Hebp2	1.10133318	0.13925099	0.0413158	0.9634059
Prkca	0.90445017	-0.144887077	0.04142139	0.9634059
Ctps2	1.1258208	0.170977213	0.04146633	0.9634059
Fhl1	1.10195124	0.140060392	0.04159083	0.9634059
Pcdhga2	1.20413895	0.268001883	0.041607	0.9634059
Acer2	1.20178651	0.265180628	0.04192571	0.9637836
Tsen34l1	1.30354239	0.382437497	0.04201851	0.9637836
Mgat4d	1.64057798	0.714204174	0.04201928	0.9637836
Ogdh	0.9111568	-0.134228752	0.04202528	0.9637836
Tead2	0.65880942	-0.602066921	0.04203138	0.9637836
Rce1	1.25469226	0.327333553	0.04238033	0.9690959
Pno1	1.1675912	0.223535238	0.04251274	0.9690959
Ccbl2	1.23033712	0.29905368	0.04253239	0.9690959

Plk3	1.22687157	0.294984239	0.04261931	0.9690959
Myo1a	0.85994772	-0.217679141	0.04275246	0.9690959
Dab2ip	0.90186011	-0.149024418	0.04275544	0.9690959
Bscl2	0.90667864	-0.141336801	0.0431044	0.9743351
Mrpl38	0.84358395	-0.245396444	0.0431516	0.9743351
Rcsd1	1.18404635	0.243725553	0.04327783	0.9753203
Efl1	1.17761223	0.235864562	0.04343307	0.9754553
Orai2	0.87386277	-0.194521357	0.04352374	0.9754553
H1f0	1.11657542	0.159080696	0.04358583	0.9754553
Por	0.89423853	-0.16126838	0.0436524	0.9754553
Pde7a	1.24565916	0.316909368	0.04378668	0.9754553
Rab3b	1.28409703	0.36075422	0.0439911	0.9754553
Rps4y2	1.40562636	0.491213151	0.04406986	0.9754553
Slc12a2	1.09670264	0.133172404	0.04413949	0.9754553
Itpr3	0.90696501	-0.140881198	0.04414471	0.9754553
Aamdc	1.44465762	0.530727616	0.04417399	0.9754553
Kif18a	1.63863563	0.71249509	0.04420379	0.9754553
Ccne1	0.78534665	-0.348598494	0.04429229	0.9754553
Scn8a	0.91088456	-0.134659873	0.04440182	0.9754553
Pitrm1	1.10584581	0.145150243	0.04444025	0.9754553
Atp6ap1l	0.74943759	-0.416119754	0.04459651	0.9765076
Slc9a5	1.34859611	0.431458336	0.04465358	0.9765076
Dkk2	1.37311333	0.457450699	0.04481707	0.9778804
Pmp22	0.90627439	-0.141980176	0.04492806	0.9778804
Ldlr	0.90877606	-0.138003271	0.04496478	0.9778804
Map3k1	1.19093123	0.252090105	0.04561838	0.9898361
Syt1	0.90354622	-0.146329698	0.04568216	0.9898361
Aldh1a1	0.88113305	-0.182568209	0.04580244	0.9899427
Gpx7	0.81308503	-0.298521859	0.04591018	0.9899427
Aspm	0.73625804	-0.441716613	0.04610431	0.9899427
Tmem202	0.61454015	-0.702420825	0.04614608	0.9899427
Pyroxd1	1.19680341	0.259186192	0.04621178	0.9899427
Bub1	0.7613031	-0.393457133	0.04626451	0.9899427
Omg	1.14159044	0.191045153	0.04627389	0.9899427
RGD1311517	1.2859508	0.362835445	0.04641546	0.9911756
Dio2	0.6363974	-0.652000149	0.04669336	0.9928878
Tmeff1	0.85080056	-0.233107116	0.04672961	0.9928878
Gas8	1.31205606	0.391829358	0.04687052	0.9928878
Jund	0.90432813	-0.145081756	0.04691036	0.9928878
Angptl2	0.87668345	-0.189872078	0.04691603	0.9928878

Cars2	1.20692892	0.271340713	0.04718915	0.9968813
Ptgdr	0.50959851	-0.972567024	0.04733331	0.9976525
Kcnb1	0.88429446	-0.177401251	0.04739462	0.9976525
Aacs	0.90397192	-0.145650141	0.04761932	1
Etfb	1.13955372	0.18846894	0.04785972	1
Sepp1	1.09781369	0.13463323	0.04793427	1
Nr2c1	1.18203509	0.241272862	0.04796792	1
Id4	1.15224146	0.204443074	0.04817286	1
Crtac1	1.17679777	0.234866418	0.04819359	1
Slc27a1	0.89798932	-0.155229802	0.04821264	1
Hp	1.48074781	0.566325956	0.04838335	1
Abcd2	1.12555773	0.170640051	0.04850573	1
Itgal	1.31152985	0.39125064	0.04913364	1
Pak6	0.6029265	-0.729945954	0.04916366	1
Prep	0.89785236	-0.155449856	0.0492923	1
Kcng1	0.81928192	-0.287568121	0.0493086	1
Setd7	1.11851527	0.161584953	0.0495152	1
Inpp5j	0.87977677	-0.184790583	0.04954127	1
Id1	0.84760406	-0.238537605	0.04991226	1
Gpnmb	1.19296688	0.25455399	0.04997366	1
Papss2	1.27129565	0.346299582	0.05001308	1
Scn3b	0.90046685	-0.151254933	0.05003352	1
RT1-A3	0.70185261	-0.510760005	0.05013349	1
P2ry1	0.88219036	-0.180838095	0.05018891	1
Ccl24	1.64266593	0.716039104	0.05026328	1
RGD1563365	0.73745992	-0.439363448	0.05039904	1
Dbp	0.89943105	-0.152915411	0.0505586	1
Rfx2	1.50407353	0.588875096	0.05098318	1
Med15	0.8797946	-0.184761341	0.05101415	1
Nnat	0.87019766	-0.200584955	0.05106207	1
Lmod1	1.31537888	0.395478412	0.05119205	1
Mblac2	0.8530399	-0.229314869	0.05126568	1
Cd55	0.91433994	-0.129197462	0.05127592	1
Agt	0.75617627	-0.40320551	0.05146105	1
RatNP-3b	1.39195349	0.47711101	0.05156504	1
Aoah	1.90778201	0.931896336	0.05170973	1
Bmp6	1.21723291	0.283605242	0.0517229	1
P2ry14	1.36273371	0.446503678	0.05195926	1
Usp34	0.91456803	-0.128837608	0.05197921	1
Selp	1.52863328	0.612242341	0.05222361	1

Csf1r	1.12286595	0.167185701	0.05242563	1
Cbx6	0.91089305	-0.134646421	0.05246215	1
Bcl6	0.87794188	-0.187802656	0.052656	1
Csrp1	1.09298892	0.128278781	0.05270195	1
Myot	0.43457566	-1.202320738	0.05276249	1
Dusp10	1.3426427	0.425075429	0.0528663	1
Mcpt9	2.25979103	1.176189371	0.05294813	1
Emc2	1.09715402	0.133766064	0.05299603	1
RT1-CE16	0.85791035	-0.221101194	0.05305842	1
Dtymk	1.15125373	0.203205834	0.05307246	1
Syt15	1.30010043	0.378623074	0.05320353	1
Acbd4	1.1705348	0.227167823	0.05333941	1
RT1-Db1	1.13935211	0.18821367	0.05340453	1
Sox10	0.90033126	-0.151472179	0.05342186	1
Haus8	1.30113558	0.379771304	0.05345223	1
Col14a1	0.90878301	-0.137992227	0.05355622	1
Adra2a	0.72870945	-0.456584393	0.05365439	1
Plppr3	0.77815869	-0.361863696	0.05371663	1
Ubqln1	0.91256769	-0.13199652	0.05378342	1
Skap1	0.6166591	-0.69745494	0.05384595	1
Cdc26	0.86417369	-0.21060678	0.05394582	1
Sorcs1	0.88133133	-0.182243597	0.05423509	1
Inhba	1.61343856	0.690138643	0.05432041	1
Hid1	0.90993828	-0.136159409	0.05443653	1
Rpap2	1.16788167	0.223894109	0.05458116	1
Ap4e1	1.18535697	0.245321589	0.0547245	1
Ankzf1	1.15154872	0.203575455	0.05487749	1
Tef	0.913355	-0.130752387	0.05494993	1
Mtmr1	0.85777307	-0.221332069	0.05503108	1
Lrrn3	0.89106639	-0.166395173	0.05504733	1
Tspan14	0.86993977	-0.201012582	0.05517924	1
Bmper	0.82497782	-0.277572755	0.0553047	1
Srcin1	0.84285336	-0.24664645	0.05544399	1
Rasgrp1	0.86518782	-0.208914733	0.05546277	1
Foxk2	0.88283901	-0.179777712	0.05551793	1
Hs6st3	0.77918059	-0.359970347	0.05579305	1
Clstn3	0.91348569	-0.130545969	0.05589939	1
Cog7	1.13798966	0.186487447	0.05600389	1
Cox6b2	1.68454849	0.752361958	0.05601838	1
Ubr4	0.91563277	-0.127158997	0.0560355	1

Lgals9	1.19342111	0.255103196	0.05616243	1
Ephb6	0.87509083	-0.192495317	0.05619146	1
Mrpl41	1.12179571	0.165809973	0.05619845	1
Apc2	0.91386352	-0.129949378	0.05629881	1
Caskin1	0.91358276	-0.130392676	0.05642243	1
Nxph1	1.26622881	0.340538127	0.05643116	1
Hltf	1.13593174	0.183876139	0.05645724	1
Slc14a1	0.67000716	-0.577751592	0.05670386	1
Ccdc125	1.49049401	0.575790581	0.05690162	1
Tnn	0.45369831	-1.140194806	0.05701054	1
Mid1	1.30521541	0.38428793	0.05710593	1
Cacna1h	0.89021813	-0.167769207	0.05714013	1
Itgb7	0.68834819	-0.538789572	0.05726345	1
Slit2	0.90364653	-0.146169542	0.05747198	1
Tmem72	0.85798481	-0.220975982	0.05751133	1
Casc3	0.88573907	-0.175046341	0.0575609	1
Dirc2	0.89184303	-0.165138293	0.05765335	1
Eps15l1	1.11896329	0.162162708	0.05777075	1
Adgrl1	0.90661886	-0.141431916	0.05779092	1
Lpcat1	0.87868611	-0.186580203	0.05780807	1
Gnao1	0.91695881	-0.125071158	0.05783041	1
Tceanc2	1.19360413	0.25532443	0.05784853	1
Csrp2	0.83076855	-0.2674815	0.05788316	1
Scd2	0.91006185	-0.135963501	0.05798384	1
Gins1	0.70961548	-0.494890611	0.05803577	1
Lyn	1.23208834	0.301105703	0.05807753	1
Ncan	0.83483467	-0.260437572	0.05808203	1
Myzap	1.42724962	0.513237679	0.05816983	1
Oas1a	1.37083992	0.455060111	0.05853511	1
Gfra3	0.86801474	-0.204208551	0.05859096	1
Polr2g	1.12857481	0.174502054	0.05876551	1
Csad	0.84973878	-0.23490868	0.05889476	1
Kel	1.59982834	0.677917115	0.05929158	1
Jup	0.91484207	-0.128405385	0.05956908	1
Cd93	1.19927823	0.262166404	0.05969759	1
Dtd1	1.12704869	0.172549847	0.05969905	1
Ntng2	0.89293802	-0.16336806	0.05978045	1
Pnmal2	0.88070403	-0.183270832	0.05986577	1
Clcn4	1.0916889	0.126561789	0.05986757	1
Lgr5	0.88123042	-0.182408795	0.0599257	1

Abcb4	1.454436	0.540459818	0.06038201	1
Fa2h	0.88751805	-0.17215164	0.06038943	1
Mppe1	1.18678186	0.247054779	0.06039613	1
Bre	1.11991758	0.163392563	0.06046233	1
Taf9b	1.14625601	0.196929298	0.06054996	1
Mul1	1.17090663	0.227626037	0.06065275	1
Lsm10	1.35559152	0.438922516	0.06065778	1
Pcdhga11	0.82135627	-0.283919949	0.06097725	1
Bmp5	1.23606664	0.305756521	0.06101521	1
Ripk4	0.70995612	-0.494198229	0.06112218	1
Cadm1	0.91429512	-0.129268178	0.06120788	1
Fat1	0.90926043	-0.13723453	0.06152503	1
Atp8b2	0.91384392	-0.129980309	0.06169365	1
Sparc	0.91250588	-0.132094234	0.06179292	1
Tex15	0.80734471	-0.308743303	0.06191636	1
Ino80e	0.86560264	-0.208223192	0.06226263	1
Tp53inp2	0.90770936	-0.139697659	0.06288247	1
Slc25a14	1.16566895	0.221158124	0.06294786	1
Tsc2	0.89902979	-0.153559177	0.06294822	1
Tsc22d3	0.91732039	-0.124502386	0.0629812	1
Errfi1	0.89077265	-0.166870833	0.06311928	1
P2rx6	1.14026657	0.18937114	0.06316738	1
Myc	0.78640722	-0.346651519	0.06336859	1
Gemin7	0.84292784	-0.246518963	0.06339091	1
Tm4sf1	1.14174793	0.191244177	0.06343928	1
Tnk2	0.91239168	-0.132274809	0.06345317	1
Frem2	1.50637632	0.591082224	0.06348716	1
Fcrl2	0.76105389	-0.393929483	0.06357356	1
Tmem86a	1.31195287	0.391715896	0.06358327	1
Lgals3bp	1.10377068	0.14244047	0.06372048	1
Inpp1	1.16659999	0.222309968	0.0637481	1
Tmem33	0.90588511	-0.142600005	0.06385429	1
Plat	1.09142164	0.126208556	0.06387252	1
Dgki	0.90033517	-0.151465914	0.06403342	1
Eif2s3y	1.09247451	0.127599616	0.0642788	1
Loxl1	0.87217133	-0.197316536	0.06429266	1
Xk	0.81323871	-0.2982492	0.06467703	1
Cpsf1	0.88614365	-0.174387511	0.06472456	1
Klf1	1.55887509	0.64050533	0.06472467	1
Tmem176a	0.91853679	-0.122590589	0.0647665	1

Aph1b	0.86383037	-0.211180061	0.06480933	1
Pum2	0.91699269	-0.125017863	0.06481596	1
Tnnt3	0.78990659	-0.340246041	0.06506778	1
Isl2	0.88616608	-0.174350997	0.06515761	1
Rpl34	1.1041731	0.142966361	0.06519692	1
Hspa5	0.91648064	-0.125823684	0.06523445	1
Ncf1	1.22962245	0.298215413	0.06538544	1
Pdlim5	0.84989186	-0.234648812	0.06546792	1
Fcgbp	1.42914912	0.515156453	0.06551437	1
Dab2	1.12699595	0.172482329	0.06553885	1
Dpf1	1.3868174	0.471777839	0.06554513	1
Sec31a	0.90737488	-0.14022938	0.06556428	1
Tmem164	0.90480368	-0.144323304	0.06568806	1
Nlgn3	0.86556512	-0.208285738	0.06590922	1
Ndufc2	1.10461807	0.143547638	0.06606232	1
Tmem209	0.83770522	-0.255485425	0.06623207	1
Zfp39	1.39935013	0.484756983	0.06629898	1
Rnase4	1.12628653	0.171573897	0.06636553	1
Tmc8	0.65761885	-0.604676439	0.06654094	1
Glrb	1.09019593	0.124587433	0.06654282	1
Commd9	1.1403065	0.18942166	0.0666349	1
Commd10	1.14325048	0.193141519	0.06672464	1
Hivep1	0.90205106	-0.148718993	0.06676373	1
Jmjd4	1.43873348	0.52479936	0.06681583	1
Hecw2	0.82568955	-0.276328652	0.06681804	1
Vamp2	0.91850726	-0.122636976	0.06721978	1
Rbm33	0.86627604	-0.207101275	0.06735083	1
Isyna1	1.12408255	0.168747982	0.06737323	1
Ttc38	0.75839646	-0.398975868	0.06737903	1
Laptm4b	1.08914836	0.12320048	0.06762633	1
Ahsp	1.29502714	0.372982329	0.06806775	1
Psmb8	1.21857546	0.285195598	0.06816422	1
Padi2	0.88561063	-0.175255559	0.06843465	1
Ddx56	0.83789936	-0.25515112	0.06849748	1
Zfp956	1.32796608	0.409218296	0.06852454	1
Mrpl15	1.1645245	0.219740992	0.06874543	1
Lmna	0.91701724	-0.124979239	0.06879523	1
Mcm7	1.20115986	0.264428166	0.06894546	1
Mphosph8	1.0932025	0.128560662	0.06897797	1
Faah	0.79542863	-0.330195606	0.06899853	1

Donson	0.81977516	-0.286699811	0.06910767	1
Adcyap1	0.87127545	-0.198799204	0.06929968	1
Ppa1	1.09016239	0.124543053	0.06934983	1
Vof16	1.230551	0.29930445	0.0694388	1
RGD1564379	0.89175291	-0.165284069	0.06956871	1
Wdr92	1.17282547	0.229988344	0.06957542	1
Tcerg1l	1.31014417	0.389725577	0.06968435	1
Golga5	1.12906841	0.175132899	0.06970846	1
Smoc2	0.9023649	-0.148217148	0.0697521	1
Hnrnpl	0.91434945	-0.129182448	0.06978337	1
Ace2	1.24229013	0.313002143	0.06982706	1
Slc36a4	0.83769789	-0.255498049	0.06990629	1
Tap2	1.17656772	0.23458436	0.07012743	1
Sgpp2	0.88227792	-0.180694913	0.07019283	1
Exoc8	0.88279043	-0.179857101	0.07027017	1
Pltp	1.1383803	0.186982595	0.0705272	1
Cyb561a3	1.18006143	0.238861965	0.07057426	1
LOC100365921	1.24464787	0.315737636	0.07059712	1
Rn5-8s	1.44762222	0.533685155	0.07076389	1
Atrn	0.92054266	-0.119443514	0.07077557	1
Ache	0.92022608	-0.119939749	0.07078212	1
Zfp213	1.2645947	0.338675078	0.07080577	1
Cd22	1.45522126	0.541238525	0.07094606	1
Ubap2	0.91513667	-0.127940876	0.07126655	1
Armc7	0.79082389	-0.338571634	0.07148309	1
Pih1d1	1.22295491	0.290371213	0.07159741	1
Mag	0.85669379	-0.223148464	0.07161617	1
Plac8	1.19182325	0.253170293	0.07161911	1
Abcg1	0.9113832	-0.133870315	0.07170844	1
Klf15	0.87081286	-0.199565388	0.07184941	1
Tor1a	0.86941834	-0.201877563	0.07186478	1
Plk2	0.883239	-0.179124224	0.07214671	1
Bex3	1.13305533	0.180218311	0.07220728	1
Ptprn	0.91941109	-0.121218022	0.07223877	1
Gpm6b	0.9208454	-0.118969125	0.07224409	1
Sema3g	0.89384312	-0.161906453	0.07237408	1
Rftn1	1.29824315	0.376560611	0.07238257	1
Msr1	1.38524735	0.470143608	0.07282717	1
Dgkz	0.92089714	-0.118888077	0.07306501	1
Nxpe3	1.09954054	0.136900791	0.07325015	1

Ank1	0.92144678	-0.118027256	0.07334221	1
Tcaim	1.15184577	0.203947551	0.07356848	1
Nfatc3	0.89205272	-0.164799119	0.07366537	1
Ccsap	1.50227857	0.587152362	0.07367243	1
Traf3	0.90693451	-0.140929713	0.0738544	1
Adamts7	0.81755499	-0.290612329	0.07443648	1
Kctd1	0.8600766	-0.217462942	0.07443763	1
Snta1	0.87653944	-0.19010909	0.07448064	1
Ptch1	1.11681413	0.159389097	0.07454212	1
Aox4	1.21644354	0.282669357	0.07457588	1
Rdh16	1.41965485	0.50554022	0.07469452	1
Cbfa2t3	0.68904532	-0.537329217	0.0747296	1
Napb	1.09694352	0.133489247	0.07475693	1
Sepsecs	1.14968436	0.201237828	0.07482899	1
Irgq	0.91846993	-0.122695601	0.07489649	1
Ube2z	0.91834514	-0.122891628	0.07501732	1
Prorsd1	1.48207931	0.567622651	0.07525267	1
Chpt1	1.20639923	0.270707415	0.07525809	1
Tspan18	0.88659912	-0.173646163	0.07527373	1
Clic1	1.0977284	0.134521144	0.07527592	1
Spg7	1.10969114	0.150158189	0.07530083	1
Crtc2	0.86503094	-0.209176358	0.07539629	1
Clip3	0.92154176	-0.117878554	0.07549159	1
Senp8	1.26162712	0.33528558	0.07568649	1
Plekhg2	0.89817074	-0.154938374	0.07595947	1
Purb	0.88013111	-0.184209643	0.07606592	1
Mpzl2	1.1813247	0.240405556	0.0762319	1
Pxmp2	1.29332015	0.371079446	0.07633958	1
Aph1a	0.8878012	-0.171691437	0.07634758	1
C1qa	1.12725446	0.172813223	0.07635219	1
Mterf3	0.86723098	-0.205511802	0.07637978	1
Ptprcap	0.62636679	-0.674920372	0.07647352	1
Slc9a2	1.20553709	0.269676037	0.0768286	1
Efhc2	1.28188422	0.358265968	0.07690647	1
Speg	0.87110808	-0.199076367	0.07723186	1
Kcnd3	0.91890605	-0.122010728	0.07723525	1
Pla2g2d	0.63511745	-0.654904683	0.07731746	1
Engase	0.84614827	-0.241017612	0.0773404	1
Tsen15	0.88304562	-0.17944013	0.07747598	1
Gpr35	0.76340538	-0.389478746	0.07749828	1

Cdh1	0.92249234	-0.116391157	0.07749934	1
Tigd5	1.51341088	0.597803724	0.07762914	1
Cebpz	1.10814318	0.148144301	0.07772045	1
Map7d2	1.08605558	0.11909794	0.07781725	1
Smim3	1.17045825	0.227073479	0.07790222	1
Sec14l1	0.91982882	-0.120562695	0.07793553	1
Scyl1	0.91196345	-0.132952086	0.07795667	1
Usp31	0.91195323	-0.132968252	0.07830931	1
Gpc3	1.0959092	0.132128265	0.07832639	1
Crmp1	0.90960744	-0.136684036	0.07852343	1
Dusp5	0.84562228	-0.241914702	0.07862397	1
Zfp423	1.15125857	0.203211897	0.07871073	1
Trim34	1.19511532	0.257149837	0.0788106	1
Slc11a2	1.13512611	0.182852585	0.07889506	1
Chka	0.89051152	-0.167293822	0.07912855	1
Mmd	0.89361331	-0.162277425	0.07941897	1
Lap3	1.09961216	0.136994768	0.07950006	1
Reg3a	0.31540385	-1.664727843	0.07963001	1
Ckmt1b	1.08452949	0.117069277	0.07971634	1
Irak3	1.17031397	0.226895626	0.07972469	1
Hacd4	1.24564112	0.316888476	0.07983694	1
Tmem231	1.13602055	0.183988929	0.07985798	1
Rcn1	0.92023528	-0.119925321	0.07996622	1
Vsir	1.15517268	0.208108532	0.08007546	1
Lum	0.92223356	-0.116795928	0.08010735	1
Clcn6	0.91458859	-0.128805166	0.08019291	1
Ly49s7	0.59540726	-0.748051279	0.08025544	1
Csdc2	1.10440249	0.143266045	0.0807478	1
Apbb1ip	1.25743528	0.330484146	0.08083353	1
Cdc42bpb	0.91662479	-0.125596786	0.080948	1
S100a9	1.12765578	0.173326745	0.08106759	1
Casp12	1.29351817	0.37130032	0.08118643	1
Fut10	0.7159282	-0.482113182	0.08126837	1
Ddrgk1	1.11217348	0.153381845	0.08137317	1
Foxp2	1.21816787	0.284712963	0.08138287	1
Dpp9	0.91070359	-0.134946527	0.08150858	1
Sv2c	0.92337563	-0.115010433	0.08179043	1
Abat	1.14524646	0.195658098	0.08188785	1
Coq2	1.15476134	0.207594719	0.08190385	1
Slc17a8	0.73628388	-0.441665976	0.08190849	1

Nsd1	0.92027772	-0.119858793	0.08191367	1
Smarca4	0.91958299	-0.120948312	0.08195917	1
Syngap1	0.86026326	-0.217149873	0.08201935	1
Socs2	0.79833395	-0.324935723	0.08202646	1
Fcna	1.28276962	0.359262094	0.08202821	1
Slit1	0.9130263	-0.131271672	0.08245261	1
Il17re	1.37144578	0.455697588	0.08246015	1
Vars	0.90234214	-0.148253538	0.08251494	1
Scn1a	1.08351641	0.115721005	0.08253124	1
Etfbkmt	1.28168704	0.358044029	0.0825645	1
Xpr1	0.87644627	-0.190262452	0.08284589	1
Mtor	0.91528639	-0.127704859	0.08287034	1
Gtse1	1.40698447	0.492606406	0.08290836	1
Cyp4f4	1.53099141	0.614466192	0.08306984	1
Nelfb	0.91950967	-0.121063349	0.08327476	1
Zc3h7b	0.92175923	-0.117538138	0.08334463	1
RGD1310587	1.12602679	0.171241146	0.08339118	1
Zbtb6	1.18323934	0.242741919	0.08344174	1
Mrpl14	1.16602511	0.221598862	0.08360223	1
Dmap1	1.15239438	0.204634531	0.08374231	1
Palm	0.91741762	-0.124349472	0.08377117	1
Gadd45b	1.19760994	0.260158103	0.08396763	1
Tpx2	0.78093231	-0.356730593	0.08420742	1
Traf1	1.44713536	0.53319987	0.08446244	1
Arg1	0.65549768	-0.60933742	0.08448218	1
Zswim5	0.87486976	-0.192859834	0.08474218	1
Gga1	0.89617073	-0.158154489	0.08492061	1
Actr8	1.11760503	0.160410414	0.08492366	1
Epas1	0.91516508	-0.127896094	0.08496392	1
Adgrf5	1.12061941	0.164296385	0.08499605	1
Smug1	0.76160685	-0.39288164	0.08503489	1
Ddhd1	0.89960805	-0.15263152	0.08506397	1
Erf	0.86385236	-0.211143325	0.08509158	1
Plcl1	1.13929768	0.188144749	0.08510661	1
Ddx51	1.19846881	0.261192365	0.08525135	1
Ch25h	1.33895696	0.421109592	0.08528075	1
Crbn	1.08262609	0.114535067	0.0853834	1
Lyrm1	1.15175074	0.203828529	0.08556598	1
Arid3b	0.7571228	-0.401400772	0.08577186	1
Ubald1	0.87905021	-0.185982531	0.08579365	1

Fam46c	1.29348309	0.371261193	0.08579487	1
Prkx	0.90331162	-0.146704323	0.08586303	1
Spg21	0.8940156	-0.161628096	0.08598322	1
Nptx1	0.92397867	-0.11406854	0.08608438	1
Perp	1.1610893	0.215478939	0.08615426	1
Abcc8	0.8460274	-0.241223711	0.08660831	1
Myh9	0.91675075	-0.125398547	0.08682542	1
ST7	1.19392235	0.255709015	0.08699352	1
Tmem229a	0.92100544	-0.118718418	0.08715738	1
Mybpc1	0.7379665	-0.438372771	0.08716926	1
RT1-S3	1.13238625	0.179366141	0.08735632	1
Atp1a3	0.92378153	-0.114376397	0.08745807	1
Haus1	1.17641804	0.234400814	0.08759339	1
Kctd8	0.90761553	-0.139846797	0.08762473	1
Pcdha11	0.78163451	-0.355433923	0.08775414	1
Ret	0.92279531	-0.115917425	0.08800938	1
Hoxc4	1.25097383	0.323051608	0.08818718	1
Pef1	1.09900334	0.136195777	0.0883963	1
Pcdhac1	0.90352568	-0.146362492	0.08841364	1
Il1rl1	1.54705451	0.629524033	0.08844196	1
Slc46a2	0.73089076	-0.4522723	0.08844276	1
Gmip	1.18067084	0.239606806	0.08850322	1
Sesn3	0.90601468	-0.142393672	0.08867573	1
Trmt61a	0.86340256	-0.211894723	0.08871005	1
Cfh	1.08576207	0.118707985	0.08881245	1
Aste1	0.73458992	-0.444988986	0.08883515	1
Grik3	0.764383	-0.387632398	0.0888902	1
Rbks	1.30816518	0.38754472	0.0889383	1
Fcnb	1.33101937	0.412531571	0.08908332	1
Ccdc89	1.38319145	0.468000853	0.0892026	1
Sptbn4	0.9079762	-0.139273621	0.08930098	1
Pcdh9	1.0864303	0.119595622	0.08938404	1
Magi2	0.85567582	-0.224863774	0.08954919	1
Htr3b	1.09022048	0.124619931	0.08958121	1
Farp1	0.88331828	-0.178994728	0.08965133	1
Snx29	0.82728921	-0.27353633	0.08971897	1
Rngtt	0.89080574	-0.166817234	0.08976374	1
Ddb2	1.40271679	0.488223756	0.08987794	1
Cd3eap	0.77458911	-0.368496876	0.08989805	1
Plscr3	0.88966228	-0.168670305	0.08991156	1

Atp5o	1.08107174	0.112462269	0.09067422	1
Gem	0.74502848	-0.424632519	0.09070066	1
Pgls	0.85167859	-0.231619007	0.09102541	1
Akt1	0.91895453	-0.121934624	0.09102795	1
Shc3	0.71692429	-0.480107325	0.09156925	1
Plekha3	0.8788539	-0.186304746	0.09168398	1
Rbm4b	0.83726565	-0.256242652	0.09178117	1
Slfn13	1.14708987	0.19797843	0.09186355	1
Htra1	1.08161114	0.113181916	0.09188117	1
Lnx2	0.85080436	-0.233100676	0.0919375	1
Zfp35	1.32190518	0.402618698	0.0919503	1
E2f4	1.1609685	0.215328826	0.09206893	1
Epb41l4a	0.84329149	-0.245896695	0.09229256	1
Prr5l	1.57189047	0.652500697	0.09233004	1
Slc6a9	1.32688705	0.408045565	0.09236139	1
Fam151a	0.76557217	-0.385389712	0.09246059	1
Rcbtb2	1.1668225	0.222585116	0.09252064	1
Zbtb7c	1.23905463	0.309239795	0.09257434	1
Isg15	1.34346699	0.425960869	0.09258933	1
Slc2a6	0.89361478	-0.162275052	0.09260495	1
RGD1311739	0.89541547	-0.159370851	0.09263194	1
Chrna4	0.65507923	-0.610258689	0.09268429	1
Nedd4l	0.92290436	-0.115746943	0.09304291	1
Adck3	1.15785943	0.211460117	0.09314599	1
Efnb1	1.12086291	0.164609839	0.09336883	1
G0s2	0.79579725	-0.329527188	0.09344159	1
Galt	0.85532197	-0.225460492	0.09374817	1
Snph	1.08999381	0.124319936	0.09387891	1
Snapin	1.0927643	0.127982259	0.09396736	1
Arhgef12	0.91208566	-0.132758763	0.0940266	1
Olfm4	1.93038882	0.948891466	0.09405311	1
Bves	0.78778288	-0.344130036	0.09409396	1
Ddx10	1.111144	0.152045801	0.09413906	1
Mfge8	1.10230395	0.140522092	0.09414812	1
Iqsec3	0.89674091	-0.157236873	0.09426874	1
Mks1	1.23508906	0.30461507	0.09452082	1
Dot1l	0.87990663	-0.184577658	0.09457757	1
Xrn1	1.15261181	0.204906705	0.09457774	1
Pou3f1	0.7782645	-0.361667538	0.09460674	1
Hdac4	0.85948487	-0.218455859	0.0947964	1

Pcdha4	0.84976045	-0.234871898	0.09494639	1
Erg	1.33579798	0.417701835	0.09529864	1
Bcam	0.92615663	-0.110671896	0.09575208	1
Hoxb8	1.320907	0.401528895	0.09581722	1
Slc5a5	0.62318535	-0.682266781	0.09585194	1
Tesk1	0.88345654	-0.178768937	0.09594441	1
Tie1	1.13849305	0.187125481	0.09628102	1
Kcna2	0.92368922	-0.114520567	0.09630616	1
Il7r	0.86700501	-0.20588776	0.09641562	1
Lppos	0.71375264	-0.486503912	0.09675296	1
Anp32e	1.10859962	0.148738416	0.09683215	1
Rps7	1.08023443	0.111344439	0.0968647	1
Syt5	0.87914326	-0.185829824	0.09692346	1
Ndst4	1.10728769	0.147030106	0.0969604	1
Cmtm4	0.82765972	-0.27289034	0.09712343	1
Bcl2l12	1.38280047	0.467592998	0.09723214	1
Cdk2ap2	1.14576747	0.196314284	0.09739617	1
Sec11c	1.1108628	0.151680641	0.09772245	1
Rc3h2	1.13458186	0.182160699	0.09776091	1
Slc52a2	0.82654164	-0.274840591	0.09801066	1
Kcnc2	1.15431783	0.207040514	0.09822061	1
Gal	0.80887888	-0.306004406	0.09823357	1
Ntrk1	0.92515813	-0.11222812	0.09838496	1
Ptpn23	0.89657005	-0.157511784	0.09844214	1
Scand1	0.88407424	-0.177760572	0.09849949	1
Epha5	0.86744324	-0.205158734	0.09886344	1
Msmo1	0.92717045	-0.10909351	0.09894539	1
Tbc1d24	0.85229157	-0.230581025	0.09905472	1
Dynlt3	1.080485	0.111679046	0.09927638	1
RGD1565355	1.54993062	0.632203636	0.09933439	1
Polr3c	1.12547913	0.170539307	0.09952809	1
Ubxn1	1.09016772	0.124550107	0.09956728	1
Ncln	0.90449438	-0.144816564	0.09973383	1
Klrk1	0.68046941	-0.555397794	0.09999834	1
Lyl1	1.36974344	0.453905691	0.09999911	1
Slc16a6	0.87271455	-0.196418252	0.10002872	1
Lrrc8b	0.90038566	-0.151385019	0.1001668	1
Marcks	0.91513256	-0.127947355	0.10039802	1
Fam45a	1.09408531	0.12972524	0.10045546	1
Slc44a1	0.92764631	-0.108353244	0.10061666	1

Hirip3	1.1490712	0.200468198	0.10061875	1
Scrg1	0.74262167	-0.429300676	0.10062957	1
Slc25a22	0.91468587	-0.128651737	0.10063383	1
Adm	1.26555699	0.339772478	0.10064246	1
Dclk1	0.92667016	-0.109872186	0.10088211	1
Ms4a6bl	1.17785898	0.236166824	0.10111539	1
Pde1c	0.91271491	-0.131763789	0.1011901	1
Adam11	0.92351947	-0.114785722	0.10131799	1
Ak3	0.90600831	-0.142403813	0.10146261	1
Pdilt	1.49683197	0.58191228	0.10149897	1
Dnajc19	0.84010077	-0.251365697	0.1019122	1
Elovl6	0.91749698	-0.124224682	0.10202924	1
Ptprs	0.92743557	-0.108681035	0.10223082	1
Zic1	1.24068182	0.31113317	0.1023251	1
Mfhas1	0.89191627	-0.165019821	0.10256484	1
Gpsm1	0.88978161	-0.168476814	0.10272472	1
Bri3	0.8803609	-0.18383302	0.10289618	1
Efcab1	1.30807075	0.38744058	0.10290497	1
Insig1	0.92733429	-0.108838594	0.10293265	1
P2rx3	0.92699604	-0.109364924	0.10294873	1
Tmem55a	0.92095741	-0.118793657	0.10298938	1
Mup5	0.70077704	-0.512972588	0.10301654	1
Sez6l2	0.9253773	-0.111886382	0.10313974	1
Pggt1b	1.15890622	0.212763822	0.10316572	1
Cntln	1.17928963	0.237918087	0.10327323	1
Ehd1	0.92069511	-0.119204609	0.10330048	1
Camk2b	0.90828582	-0.138781735	0.10350519	1
Serinc5	0.92076552	-0.119094291	0.10366873	1
Arhgef6	0.88778245	-0.17172191	0.10378049	1
Tbcb	1.0871482	0.120548618	0.10393939	1
Erich1	1.20910783	0.273942912	0.10395725	1
Slc5a7	0.80625241	-0.310696535	0.10402278	1
Hoxd9	1.28886078	0.366096434	0.10403054	1
Alg10	0.86960413	-0.201569305	0.10404618	1
Dctn1	0.92670286	-0.109821265	0.10420456	1
Hnrnpul2	0.92225398	-0.116763986	0.10423213	1
Piwil2	1.1574136	0.210904497	0.10423649	1
Angpt2	1.17772222	0.235999308	0.10451513	1
Fcmr	1.23723602	0.307120738	0.10470763	1
Ostc	1.09445321	0.130210279	0.10490475	1

Cacna2d1	0.91321533	-0.130973013	0.10527188	1
Hmmr	1.20969606	0.274644616	0.10539902	1
Kcnq3	0.88620083	-0.174294409	0.10544615	1
Inafm1	1.20431207	0.268209286	0.1054531	1
Gpr19	1.28606	0.362957956	0.10549182	1
Camk2n2	0.81736535	-0.290947016	0.10554364	1
Cnr2	1.49573893	0.580858385	0.10582617	1
Guk1	1.08388595	0.116212961	0.1059025	1
Cdc25c	1.23984601	0.310160944	0.10590887	1
Slc16a13	1.2926323	0.37031195	0.10604955	1
Gjb2	1.18932208	0.250139461	0.10615523	1
Pvrl1	0.88388705	-0.17806608	0.10629775	1
Ugt1a6	1.19487231	0.256856457	0.10640875	1
Nkd1	0.8356138	-0.259091776	0.10643227	1
Plcg2	1.289131	0.366398871	0.10648631	1
Sema3b	1.07680004	0.106750371	0.1065201	1
Asrgl1	0.90405785	-0.145513008	0.10664702	1
Hip1	0.92006114	-0.120198364	0.10665171	1
Btg3	1.16235632	0.217052396	0.10669059	1
Kcnj3	1.12482489	0.169700423	0.10672905	1
Gtf3c6	1.20033962	0.263442649	0.10675649	1
Mtss1l	0.90425899	-0.145192061	0.10691736	1
Hcfc1r1	1.08949813	0.123663715	0.10726572	1
Syn1	1.07920953	0.109974991	0.10734308	1
Abtb2	0.90016205	-0.151743358	0.10736745	1
Sav1	0.8831175	-0.179322689	0.1074153	1
Rsl24d1	0.90468415	-0.144513897	0.10756368	1
Ankrd13c	0.91843744	-0.122746637	0.10768507	1
Thoc3	1.13016367	0.17653172	0.10787976	1
Tnfrsf11b	1.25426053	0.326837052	0.10803135	1
Cdpf1	0.77259316	-0.372219195	0.10811489	1
Ddt	1.09960707	0.136988093	0.1083064	1
Creld2	0.86214962	-0.213989826	0.10849472	1
Cnot3	1.14218336	0.191794275	0.10850148	1
Gjb6	1.18929746	0.250109595	0.1085348	1
Apobr	1.38150966	0.466245648	0.10883691	1
Icam1	1.19931598	0.262211815	0.10909373	1
Mnda	1.34900994	0.431900975	0.1091966	1
Cope	1.10658699	0.146116864	0.10926429	1
Higd2a	1.10383423	0.142523535	0.10932376	1

Klf16	0.84793694	-0.237971119	0.1094404	1
Rpl12	1.11278579	0.154175897	0.10998131	1
Kcnn2	1.19062613	0.25172046	0.11005238	1
Cyb5b	0.91553812	-0.127308138	0.11014662	1
Nipsnap1	1.08169223	0.113290072	0.11024818	1
Tatdn3	0.85064851	-0.233364968	0.11029269	1
Prkacb	1.09275077	0.127964401	0.11043104	1
Cdc42ep4	0.90521766	-0.143663361	0.11048017	1
Gcn1l1	0.91778972	-0.123764451	0.11049408	1
RGD1562136	1.08907656	0.12310538	0.11051709	1
Eln	1.13529872	0.183071947	0.1105845	1
Igsf11	0.91117676	-0.134197145	0.11065463	1
LOC361016	1.24977233	0.321665308	0.11074254	1
Acp5	0.78944943	-0.34108124	0.11079725	1
Vmp1	1.08228305	0.114077863	0.11089433	1
Rbm7	1.14158025	0.191032285	0.11097433	1
Wdfy3	0.92980619	-0.104998071	0.11117428	1
Arhgef1	0.91043321	-0.135374912	0.11118218	1
Zdhhc18	0.89159815	-0.165534478	0.11136912	1
Gas2l1	0.89320228	-0.162941153	0.11141822	1
Maf	1.13353557	0.180829659	0.11144152	1
Slc45a1	0.91415588	-0.129487903	0.11149298	1
Ednra	0.86290943	-0.212718955	0.11150621	1
Creb3l1	1.16360277	0.218598639	0.11155712	1
LOC361646	0.87609638	-0.190838509	0.11161485	1
Rasl11b	0.84673867	-0.240011323	0.11162336	1
Luc7l3	1.07867038	0.109254069	0.11163466	1
Gdpd1	1.12159952	0.165557642	0.11172662	1
Tmem51	1.23044703	0.299182557	0.1118458	1
Ppp1r9b	0.9231474	-0.115367065	0.1118664	1
Akap10	0.89636202	-0.157846575	0.11193854	1
Pde9a	0.78761974	-0.344428821	0.11203568	1
Tmem184c	1.0767269	0.106652366	0.11213932	1
Krt19	0.80338251	-0.315841037	0.11214554	1
Kptn	1.3539156	0.437137808	0.11221854	1
Spr	0.85852762	-0.220063553	0.11227229	1
Tlr7	1.21197374	0.277358446	0.11233168	1
Cacnb1	0.88764713	-0.171941817	0.11239772	1
Pten	0.91281285	-0.131608989	0.11242815	1
RGD1564053	1.13534067	0.183125253	0.11248691	1

Tsta3	1.12716521	0.172698991	0.11256115	1
Gbp5	1.20185077	0.265257767	0.11286443	1
Rabl3	1.14370214	0.193711373	0.11293121	1
Adgre4	1.82970604	0.871611887	0.11299481	1
Rnf139	1.12107815	0.164886851	0.11319253	1
Zbtb16	0.82313303	-0.280802484	0.11325697	1
Marcksl1	0.91802257	-0.123398479	0.11333608	1
Cd5	0.63802871	-0.648306752	0.11337123	1
Qk	0.90430089	-0.145125217	0.11377233	1
Ppic	0.87994791	-0.184509974	0.11382491	1
Xiap	0.89702566	-0.156778839	0.11384347	1
Rhobtb2	0.89822899	-0.154844817	0.11385889	1
Kcnj6	0.8737239	-0.194750633	0.11396572	1
Htr1b	0.81910351	-0.287882324	0.11422414	1
S1pr1	0.86869689	-0.20307522	0.11422944	1
Irf3	1.09872624	0.135831964	0.11425064	1
Des	1.23449172	0.303917159	0.11431777	1
Stt3b	0.91428081	-0.129290757	0.11434759	1
Tmem200a	0.85459309	-0.226690441	0.1146424	1
Mbtps2	1.217746	0.284213243	0.11473339	1
Mgrn1	0.91533862	-0.127622544	0.11482531	1
Cacna1e	0.74662478	-0.421544694	0.11490748	1
Tpm1	1.07521519	0.104625422	0.11492516	1
Prdx2	1.07591619	0.105565701	0.1150502	1
Fam219a	0.91493708	-0.128255562	0.11531804	1
Fam129b	0.91559582	-0.127217212	0.1153744	1
Gstm4	1.11425052	0.156073628	0.11538281	1
Fras1	0.87973285	-0.184862612	0.11547991	1
Nectin4	1.35009718	0.433063252	0.11569261	1
Cab39l	1.10128114	0.139182819	0.11571722	1
Gstm6	1.28219374	0.358614268	0.11577559	1
Enpp3	0.88712048	-0.17279805	0.11588703	1
Usp11	1.09794576	0.13480678	0.11598681	1
Lpar6	1.19462975	0.256563553	0.11601623	1
Notch1	0.9175648	-0.124118051	0.11607116	1
Galnt2	0.91349451	-0.130532038	0.1162229	1
B4galt5	0.89376891	-0.162026233	0.11656082	1
Ing1	1.18121494	0.240271504	0.11681269	1
Slc37a1	0.88833504	-0.170824202	0.11686005	1
Tln1	0.92998057	-0.104727516	0.11693695	1

Clic2	1.15904626	0.212938146	0.11699375	1
RGD1559896	0.91361854	-0.130336175	0.11703514	1
Grasp	1.30821185	0.387596189	0.11718908	1
Ranbp17	0.82335498	-0.280413523	0.11740616	1
Tox2	0.87070657	-0.199741479	0.11751595	1
Fam46a	1.34594189	0.428616126	0.11797784	1
Ap2a1	0.92790094	-0.107957292	0.11820888	1
Vwf	1.09536122	0.131406705	0.1183163	1
Hbp1	1.09369845	0.129215022	0.11837812	1
Ero1b	0.83532657	-0.259587767	0.11845414	1
Mustn1	1.20686852	0.271268508	0.11860236	1
Mak16	1.13157749	0.17833538	0.11868016	1
Cd274	1.20606779	0.270311006	0.11868497	1
Reln	0.92967881	-0.105195728	0.11905582	1
Igfbp5	1.07540166	0.104875599	0.11912601	1
Nap1l5	1.07508399	0.10444938	0.11927783	1
Tph2	0.87401561	-0.194269053	0.11939157	1
Neurl4	0.91222281	-0.132541852	0.11946845	1
Scg5	1.07457217	0.103762384	0.11959439	1
Mkks	1.09638775	0.132758117	0.11974098	1
Fam180a	1.15115868	0.203086718	0.11976916	1
Nhlrc2	0.87161653	-0.198234546	0.11981843	1
Nrep	0.84162886	-0.248743918	0.12003564	1
Gimap9	1.35436525	0.437616866	0.12004456	1
Glyr1	0.91067708	-0.134988513	0.12004544	1
Cd27	0.66614861	-0.586084038	0.12016343	1
Tagln3	1.0785905	0.109147237	0.12018393	1
Extl2	1.08665524	0.11989429	0.12030614	1
Cirbp	1.11570716	0.157958415	0.12032832	1
Dgat2	0.87283641	-0.196216813	0.1204072	1
Tbc1d19	1.09295332	0.128231786	0.12108005	1
Bet1l	1.13708392	0.185338731	0.12121028	1
Arl11	1.33141442	0.412959702	0.1212351	1
LOC365985	0.92342043	-0.114940439	0.12128405	1
Pappa	0.78904383	-0.341822661	0.1214355	1
Secisbp2l	0.93062408	-0.103729575	0.12160792	1
Anks1b	0.89200189	-0.164881322	0.12174652	1
Slc12a1	0.7446777	-0.425311938	0.12180535	1
Poc5	1.16838762	0.224518971	0.12185116	1
Ctsg	0.60634978	-0.721777815	0.12194708	1

Gas6	1.07363265	0.102500456	0.12199719	1
Ubqln2	0.92229335	-0.116702404	0.12219356	1
Pabpn1	0.92436609	-0.113463752	0.12222819	1
LOC100302372	0.80116108	-0.319835763	0.12223452	1
Apobec1	1.36105828	0.444728844	0.12227759	1
Cops3	1.08547572	0.118327455	0.12256093	1
Chgb	1.07351973	0.1023487	0.12266922	1
Npy5r	0.77897997	-0.360341855	0.12309148	1
Unc13a	0.9141393	-0.129514077	0.12309776	1
Rhbdl3	1.14422113	0.194365892	0.12311776	1
Naalad2	1.5172225	0.601432669	0.12311835	1
Cubn	1.20169506	0.265070843	0.12331423	1
Rab31	0.92035185	-0.11974258	0.12347354	1
Add2	0.92795206	-0.10787782	0.12348918	1
Shox2	1.13168515	0.178472638	0.12350724	1
Tmem220	1.29917303	0.377593592	0.12359621	1
Abcg3l3	1.13598299	0.18394123	0.12368119	1
Cog4	1.07935404	0.110168162	0.12369825	1
Mrps7	1.10354733	0.142148506	0.12377812	1
Prpf31	1.10718747	0.146899519	0.12385741	1
Sema4g	0.87607336	-0.190876411	0.12404691	1
Atp6v1f	1.07738612	0.107535386	0.12412463	1
Nadsyn1	1.16557578	0.221042807	0.12412677	1
Taf13	1.08397905	0.116336881	0.12420382	1
Sdf4	1.07553491	0.10505435	0.1242911	1
Fbxl12	1.28283201	0.359332256	0.1243077	1
Slco2b1	1.12659668	0.171971119	0.12444887	1
Polr2c	1.12169802	0.165684333	0.12454156	1
Adamts2	0.88966326	-0.168668724	0.1245469	1
Lrrc51	0.74762681	-0.419609788	0.12457599	1
Sdr39u1	1.13109515	0.1777203	0.12481322	1
Thbs1	0.90456099	-0.144710315	0.12481451	1
Dcaf12	0.86636687	-0.206950027	0.12484047	1
Tppp3	1.07628633	0.106061934	0.12502066	1
Arvcf	0.91408484	-0.129600022	0.12511504	1
Abcb1	1.10026021	0.137844763	0.12518289	1
Clic5	0.83947595	-0.252439095	0.12521135	1
Cdca3	0.63529901	-0.654492327	0.12550163	1
Wdr13	1.1237215	0.168284522	0.12556445	1
Cpt1b	0.76415466	-0.388063431	0.12580775	1

Lamc1	0.93223021	-0.101241829	0.12583104	1
Henmt1	1.31002749	0.389597085	0.12583395	1
Pde2a	0.91938566	-0.121257931	0.12588891	1
Nuf2	0.69398412	-0.527025454	0.12601597	1
Fgd4	1.12334114	0.167796122	0.12620858	1
Dusp15	0.92741111	-0.108719088	0.1262573	1
RGD1309779	1.27540397	0.350954274	0.12661823	1
Hpca	0.8415871	-0.248815504	0.12669298	1
Nat8f5	0.72751884	-0.458943492	0.12674608	1
Fez1	1.0734997	0.102321788	0.12689017	1
Usp20	0.9312624	-0.102740366	0.12742196	1
Kctd4	0.80820591	-0.307205187	0.12761561	1
Sncaip	0.81495744	-0.295203381	0.12765626	1
Rnf168	1.11416981	0.155969126	0.12774557	1
Azin1	0.93216104	-0.101348875	0.1277945	1
Zmat3	0.92224129	-0.116783838	0.12779816	1
Fes	1.2985865	0.37694212	0.12795675	1
B3gnt7	0.7092079	-0.495719496	0.12796954	1
Enkd1	1.35762219	0.441082053	0.12798712	1
Cpne2	0.88055919	-0.183508111	0.12807164	1
Amigo3	0.90399998	-0.145605353	0.1280967	1
Bloc1s5	1.09389656	0.129476321	0.12811613	1
Ppp1r3c	1.13986499	0.188862954	0.12814898	1
Mtmr4	0.93229803	-0.101136879	0.12839042	1
Rbm22	0.87381288	-0.194603717	0.12852987	1
Aldh16a1	1.14687814	0.197712106	0.12853214	1
Naa20	1.09255687	0.127708379	0.1286229	1
Rab27a	1.1097141	0.150188033	0.12866239	1
Nup107	1.11732581	0.160049935	0.12866879	1
Mylip	1.12396446	0.168596412	0.12871311	1
Hus1	1.11872478	0.161855154	0.12884605	1
Prr3	0.89041919	-0.167443405	0.1290895	1
Hormad2	1.47983433	0.565435668	0.12909997	1
Hspb7	1.4665264	0.55240304	0.12922988	1
Prrx2	0.77702218	-0.363972321	0.12930436	1
Selm	1.07601563	0.105699029	0.1293746	1
Car3	1.33698357	0.418981735	0.12941823	1
Dclre1c	1.22566347	0.293562909	0.12960649	1
Hn1l	0.88656031	-0.173709325	0.12966217	1
Lrfn1	0.87176826	-0.197983425	0.12992451	1

Hbegf	1.14680904	0.197625184	0.12995109	1
Rwdd3	0.80871442	-0.306297764	0.1300671	1
Tmem255b	1.30460104	0.383608687	0.13041747	1
Pcdhga1	1.25118525	0.323295413	0.13049732	1
Mgp	0.93279281	-0.100371427	0.13065025	1
LOC500959	1.08377067	0.116059507	0.13068222	1
Iscu	1.07168278	0.099877929	0.1307397	1
Cdh11	0.93102983	-0.1031007	0.13088661	1
Mab21l3	0.79023685	-0.339642973	0.13090632	1
S100a8	1.13528876	0.183059288	0.13091946	1
Aurkb	1.54100731	0.623873709	0.13094671	1
MGC108823	1.23794984	0.307952858	0.13102106	1
Dnaaf5	0.8391971	-0.252918395	0.13103446	1
Nosip	1.11361739	0.155253651	0.13122678	1
Utp4	0.89309758	-0.163110287	0.13139815	1
Flna	1.07157709	0.099735645	0.13144816	1
Dhx58	1.29224692	0.369881764	0.13150254	1
Ifit2	1.14559407	0.196095933	0.13159121	1
Cdk13	0.90629166	-0.141952692	0.13179616	1
Urb2	0.84425873	-0.24424291	0.13198214	1
Sema3e	0.87464332	-0.193233292	0.1320871	1
Hdc	1.48810674	0.573478014	0.13220431	1
Prnp	1.07473445	0.103980232	0.13220642	1
Ngly1	1.0840834	0.116475749	0.13226461	1
Dcakd	0.89740408	-0.156170344	0.13230794	1
Mettl13	0.88420124	-0.177553344	0.13241036	1
Pitpnm2	0.93356744	-0.099173851	0.13241113	1
Zfp202	1.218537	0.285150056	0.13257946	1
Ell3	0.54823558	-0.867132141	0.13260524	1
Ptprf	0.93181262	-0.101888218	0.13273608	1
Cyb561	0.87807267	-0.187587751	0.13281107	1
Rnd1	0.81693864	-0.291700373	0.13291241	1
Anapc7	0.89359523	-0.162306609	0.13299105	1
Tgif1	1.29265237	0.370334351	0.13302704	1
Tmem223	1.10404437	0.142798159	0.13328054	1
Qtrt1	1.29074693	0.368206163	0.13331069	1
Ier5l	0.79608343	-0.329008452	0.13341052	1
Erc1	0.89093909	-0.166601286	0.13352004	1
Add3	0.93378504	-0.098837614	0.13373399	1
Traf3ip2	0.85836996	-0.220328516	0.13378067	1

Casc1	1.15400036	0.206643678	0.13381417	1
Psmd13	1.07582259	0.105440182	0.13399344	1
Rgcc	0.87963614	-0.185021217	0.13402337	1
Hivep2	0.92426874	-0.113615708	0.13426229	1
RGD1359508	1.31260747	0.392435545	0.13445867	1
Chrnb2	0.92160467	-0.117780061	0.1345289	1
Slc15a4	0.89383679	-0.161916663	0.13453776	1
Maob	0.92343361	-0.11491985	0.13470596	1
Med23	1.10111278	0.138962241	0.13490801	1
Slc12a7	0.91140735	-0.133832092	0.13498143	1
Plxnb2	1.08704026	0.120405371	0.13524297	1
Phex	1.33679381	0.418776962	0.13535254	1
Syndig1	1.16974744	0.226197072	0.13538574	1
Ccdc32	0.91677591	-0.125358956	0.13541641	1
Trub2	0.88267744	-0.180041774	0.13542308	1
Zcchc2	0.88234096	-0.18059183	0.13561349	1
Slc25a42	0.88402844	-0.177835318	0.13587096	1
Gpr183	1.34069875	0.422985104	0.13587305	1
Tfap4	1.2807056	0.356938875	0.13593244	1
Fbxo27	0.83197523	-0.265387519	0.13609385	1
Lamtor2	1.10179836	0.139860218	0.1361036	1
Atg12	1.07908978	0.109814895	0.13614065	1
Nts	1.39154008	0.476682463	0.13628203	1
Dpt	1.082509	0.114379022	0.1362896	1
LOC500956	1.13361994	0.18093704	0.1365042	1
Lrrcc1	1.1514498	0.203451521	0.13652105	1
Pdgfrl	0.86042039	-0.216886373	0.13665292	1
Zgpat	1.1370296	0.185269812	0.13672186	1
Prrt3	0.90385671	-0.145834022	0.13682395	1
Ppp1r15b	0.91121452	-0.134137359	0.1369092	1
Rrp9	0.85312199	-0.229176038	0.13713438	1
F2rl1	0.73229851	-0.449496239	0.13719445	1
Agtr1b	1.48770279	0.573086336	0.13765203	1
Coro2a	0.89901779	-0.153578427	0.13770059	1
Nsmaf	1.12003224	0.16354026	0.13789606	1
Slc43a1	1.31948011	0.399969603	0.13792277	1
Sla	1.34118961	0.423513211	0.1380831	1
Trpv1	0.92787749	-0.107993761	0.13819838	1
Atox1	0.90460922	-0.144633389	0.13834775	1
Prx	0.93252575	-0.100784537	0.13844513	1

Mrps18a	1.09406629	0.129700159	0.13859248	1
Hopx	1.07472369	0.10396579	0.13865474	1
Ctbp2	0.87534525	-0.19207595	0.13880812	1
Chek1	0.80265286	-0.317151927	0.13883508	1
Foxo4	1.11991231	0.163385778	0.13891064	1
Bclaf1	1.0753616	0.104821855	0.13935123	1
Pdzd8	0.93475271	-0.097343343	0.13959243	1
Aldoc	1.07173359	0.099946328	0.13977238	1
Polr2m	0.93105743	-0.103057939	0.13994895	1
Acvr2a	0.88240897	-0.180480646	0.14004097	1
Gpr162	0.89377811	-0.162011389	0.14024902	1
Lrrc75b	0.84598642	-0.241293591	0.14025722	1
Cep85l	0.84943186	-0.235429865	0.14051451	1
Ndp	1.13792123	0.186400693	0.14051959	1
Hipk3	0.91679525	-0.125328533	0.14074727	1
Fam195a	1.34985678	0.432806347	0.14074892	1
Ankra2	0.88387125	-0.178091854	0.14079089	1
Reck	1.08186854	0.11352521	0.14085164	1
Cnksr3	1.20876289	0.273531276	0.1408672	1
Abl1	0.91740573	-0.124368178	0.14128372	1
Tmem151b	0.86215774	-0.213976249	0.14128881	1
Hyal2	0.88616514	-0.174352518	0.14157305	1
Frs3	1.16368816	0.218704505	0.14159897	1
Bin3	1.11757886	0.160376642	0.14169627	1
Tsr2	1.07780348	0.108094144	0.1417511	1
RGD735029	1.09012768	0.124497119	0.14176693	1
Tmem151a	0.92551983	-0.111664188	0.14182013	1
Mon1a	0.88961202	-0.168751818	0.14183111	1
Ttc9c	1.0964917	0.132894894	0.14192652	1
Pcdha10	1.20231794	0.265818453	0.14219621	1
Elk4	0.91381106	-0.130032198	0.14236889	1
Srd5a1	0.8822093	-0.180807127	0.14262949	1
Sdhaf3	0.76407237	-0.388218804	0.14288458	1
Trappc4	1.08648524	0.119668572	0.14292643	1
Alg14	1.10918312	0.149497567	0.14293074	1
Dnajc17	1.23908277	0.309272567	0.14312257	1
Mettl3	1.09792248	0.134776198	0.1432919	1
Tlk2	1.09288604	0.128142975	0.1433344	1
Pck2	0.89824893	-0.154812783	0.1433503	1
Ccpg1os	1.34211567	0.424509013	0.14350186	1

Olfm2	0.85340563	-0.228696463	0.14356639	1
Samd4b	0.90328118	-0.14675295	0.1435827	1
Plpp3	1.07220511	0.100580917	0.14367922	1
Phospho2	1.11543591	0.157607625	0.14373092	1
Gla	1.08650975	0.119701123	0.1437438	1
Igsf9	1.31395472	0.393915557	0.14400609	1
Fam57b	1.06960323	0.097075725	0.14413052	1
Kif20b	1.28024544	0.356420421	0.14463382	1
Snrpb2	1.1028157	0.14119171	0.14493838	1
Ddx31	1.17276821	0.229917902	0.14499413	1
Tcf4	1.14046623	0.189623723	0.14500373	1
Psmc5	1.06952995	0.096976887	0.14506161	1
Hk2	1.21929442	0.286046528	0.14513151	1
Arhgap12	1.08186129	0.113515539	0.14513568	1
Fzd6	1.24742306	0.318950836	0.14513984	1
Cyp1b1	1.18077482	0.239733861	0.14523175	1
Ybx1-ps3	0.93298675	-0.100071503	0.14525677	1
Trmt6	1.11968272	0.163089982	0.14532473	1
Parn	1.12680767	0.172241291	0.1453746	1
Tek	1.11130375	0.152253196	0.14539631	1
Fkbp3	1.0694184	0.096826409	0.14556069	1
Cbwd1	1.19089112	0.252041512	0.14564803	1
Chmp3	1.07401037	0.103007926	0.14567019	1
Gfra1	0.93437516	-0.097926176	0.1458783	1
Timp4	1.19030381	0.251329847	0.14596273	1
Mrpl40	1.12655043	0.171911902	0.14600978	1
Ybx3	1.07568003	0.105248999	0.1460325	1
Zfp703	0.8663385	-0.206997258	0.14629433	1
Mex3b	0.81016999	-0.30370344	0.1463777	1
Clptm1	0.9337626	-0.098872296	0.14639682	1
Aldh1a3	1.1741666	0.23163712	0.14642078	1
Mtf1	0.88327746	-0.179061396	0.14651396	1
Nmnat1	0.847192	-0.239239131	0.14657207	1
Tuba1c	0.92855477	-0.106941081	0.14659499	1
Kcnt1	0.91798495	-0.12345759	0.1466194	1
Grin3a	1.26991173	0.344728225	0.14674759	1
Ncor1	0.93085719	-0.103368238	0.14693713	1
Gm2a	1.08620432	0.119295511	0.14694062	1
Fkrp	0.89779895	-0.155535693	0.14702096	1
Hyou1	0.93318797	-0.09976038	0.14704113	1

Wdr45	1.12398987	0.168629036	0.14713287	1
Ctxn3	0.90821941	-0.138887232	0.14731593	1
Plcd3	1.15344128	0.205944564	0.14751108	1
Recql	1.09892117	0.136087896	0.14757944	1
Mcc	1.17993894	0.238712201	0.14768512	1
Il1r1	1.22568078	0.293583283	0.14785814	1
Actn4	0.93603731	-0.095362052	0.14790148	1
Ankrd34c	1.35054215	0.433538668	0.14790283	1
Stom	1.13053967	0.17701162	0.14806054	1
Anapc10	1.17654937	0.234561856	0.14827068	1
Ing3	1.13814334	0.186682266	0.14832235	1
Nectin3	0.87265825	-0.196511319	0.14833741	1
Plk5	0.71227363	-0.489496511	0.14839524	1
Crh	0.67931205	-0.557853659	0.14841328	1
Surf2	1.10690974	0.146537591	0.14850636	1
Nxnl2	1.39066018	0.475769923	0.14855854	1
Clu	1.07221099	0.100588828	0.14871144	1
Bud31	1.0972134	0.133844151	0.1487774	1
Fkbp4	0.93510991	-0.096792152	0.14894969	1
Zbtb7b	0.83492665	-0.260278635	0.14901858	1
Safb	0.92209201	-0.117017377	0.1490767	1
Lactb	1.14206822	0.191648836	0.14917612	1
Cyp46a1	0.78670532	-0.346104758	0.14930684	1
Ccdc90b	1.12294011	0.167280985	0.14936639	1
Car11	0.90438398	-0.144992653	0.14939539	1
Gnl3	1.10274841	0.141103678	0.14951299	1
Mfap3	0.84771718	-0.238345077	0.14954684	1
Gtf3c1	0.93447181	-0.097776951	0.1497419	1
Sbf1	0.93018398	-0.104412001	0.14979509	1
Rpl38	1.07149936	0.099630992	0.14982184	1
Dhh	0.92060323	-0.119348596	0.15003516	1
Ifi44	1.10352536	0.142119785	0.15006412	1
Myh7b	1.17816971	0.236547364	0.15009401	1
Cnnm1	0.90669127	-0.141316699	0.15010612	1
Hmgn5	1.24135156	0.311911756	0.15056643	1
Rgs18	1.30865773	0.388087822	0.15062976	1
Prpf4	0.88809385	-0.17121596	0.1506733	1
Skp1	1.07133244	0.099406226	0.15067989	1
Pxdc1	0.88451644	-0.177039133	0.15081136	1
Adora1	0.88783241	-0.171640717	0.15100073	1

Atp2b3	0.92627065	-0.110494294	0.15109929	1
Asb1	0.89611069	-0.158251153	0.1512226	1
Gjc1	0.75618733	-0.403184415	0.15126385	1
Lhfpl5	1.10688539	0.146505846	0.15148657	1
Trim9	0.88409984	-0.177718789	0.15150445	1
Polr1b	0.88554886	-0.175356193	0.15157307	1
Plaur	1.16869112	0.224893685	0.15181957	1
Adamts9	0.87373664	-0.194729599	0.15183116	1
Mmp15	0.93308911	-0.099913224	0.15189778	1
Sptlc2	0.92851024	-0.107010275	0.15190538	1
Ppm1d	1.15862254	0.212410631	0.15207273	1
Cdk2	0.79726661	-0.32686585	0.15213512	1
Tpm3	0.9327137	-0.100493788	0.15221794	1
Tnnt2	1.18776893	0.248254196	0.15239509	1
Slc38a1	1.09759606	0.134347202	0.15277081	1
Tacc2	0.92631602	-0.110423623	0.1528048	1
Rnf138	0.8697318	-0.201357504	0.15302475	1
Pcdhb6	1.44755028	0.533613461	0.15328097	1
Gsdmd	1.22607515	0.294047407	0.15394367	1
Fmo4	1.25880734	0.332057493	0.15455744	1
RT1-CE14	1.15573046	0.208804973	0.15456353	1
Plxna3	0.92192523	-0.117278339	0.15459105	1
Apol9a	1.25540644	0.328154513	0.15461553	1
Slamf9	1.31728732	0.397570049	0.15469508	1
Kdelr3	0.90117846	-0.150115269	0.15485316	1
St14	1.3197426	0.400256572	0.1550818	1
Kctd13	0.86547387	-0.208437826	0.15513229	1
Apba1	0.93624084	-0.095048398	0.15517077	1
Oxr1	1.06884799	0.096056695	0.15525549	1
Cenpk	1.33299757	0.414674154	0.15569815	1
Aida	1.07419695	0.103258532	0.15574538	1
Ahsa1	1.06777974	0.094614079	0.15581296	1
Naf1	1.22304122	0.290473023	0.15594039	1
Npm1	1.07176759	0.099992096	0.15604145	1
Ptpn6	1.16935526	0.225713299	0.1560874	1
Gchfr	1.24987375	0.321782377	0.1561688	1
Synj2	0.8611444	-0.215672919	0.15617288	1
Cdc7	0.80502401	-0.312896283	0.15624462	1
Wdr72	1.38574145	0.470658107	0.1562703	1
Mrpl49	1.10043332	0.138071733	0.15629508	1

Clec4a1	1.24993237	0.321850038	0.1563821	1
Diaph3	0.82191156	-0.282944937	0.15641931	1
Rnf216	0.90121451	-0.15005756	0.15642259	1
Ier2	0.86864429	-0.203162583	0.15644226	1
Cluap1	1.10675078	0.146330386	0.15649657	1
Myh7	0.68307938	-0.549874863	0.15653262	1
Cdh23	0.68813316	-0.539240319	0.15656454	1
Tab1	1.11306956	0.15454375	0.15660249	1
Pcdhga9	0.81920965	-0.287695377	0.15677451	1
Ephx2	1.24172817	0.312349383	0.15703597	1
Folr2	1.21604294	0.282194176	0.15707877	1
Taf1c	0.85576933	-0.224706122	0.15715817	1
S100b	1.07104816	0.099023357	0.15727146	1
Ipo13	1.09117162	0.125878023	0.157288	1
Taldo1	0.93478259	-0.097297228	0.15751919	1
Arhgap25	1.15822929	0.21192089	0.15771011	1
Myh14	0.93383302	-0.098763497	0.1577321	1
Sirt4	1.2934105	0.371180222	0.1578079	1
Asb7	0.80048935	-0.321045891	0.15787599	1
Tcf12	1.11522003	0.157328379	0.15788437	1
Wnt5a	1.15228367	0.20449592	0.15794521	1
Ska2	1.22126398	0.288375072	0.15799796	1
Rgs7	0.92181106	-0.11745701	0.15806069	1
Cited2	0.88341951	-0.178829407	0.15818567	1
Manea	0.8933898	-0.162638311	0.1582116	1
Klhl42	0.89291169	-0.163410599	0.15837594	1
Cmss1	1.17071985	0.227395887	0.15842117	1
Prickle2	1.09446486	0.13022564	0.15845181	1
Mapt	0.93707075	-0.093770115	0.15859402	1
Tubb2b	0.93655665	-0.094561838	0.15869815	1
Eci1	1.13865729	0.187333599	0.15876188	1
Bnip1	0.8699123	-0.201058138	0.1588234	1
Trpc1	0.91521808	-0.127812541	0.15887708	1
Cse1l	1.0725563	0.101053378	0.15898227	1
Atp2a2	0.93628632	-0.094978316	0.1590117	1
Rtn4rl1	0.93163105	-0.102169364	0.15920635	1
Thbd	1.08130483	0.112773285	0.15927591	1
Prrc1	1.18497481	0.244856387	0.15931828	1
Snrk	0.91538036	-0.127556759	0.15942436	1
Tulp4	0.91468866	-0.128647326	0.15951323	1

L1cam	0.93656062	-0.094555715	0.15957598	1
Mark3	0.92168124	-0.117660212	0.15959476	1
Mybl1	1.21124411	0.276489649	0.15960635	1
Copz2	0.85837873	-0.220313773	0.15965089	1
Reep2	0.93660831	-0.094482259	0.15966965	1
Arhgap31	0.90599488	-0.142425196	0.1596763	1
Stambp	0.91045197	-0.135345181	0.15968635	1
Htra2	0.9055328	-0.143161191	0.15972914	1
Trim10	1.66216095	0.733060087	0.15984676	1
Kti12	0.86056144	-0.216649894	0.15995981	1
Cln6	0.90336646	-0.14661675	0.16023022	1
Traf2	1.1393689	0.188234934	0.16026975	1
Naa50	0.90501703	-0.143983154	0.16032456	1
Tmem179b	1.22373282	0.291288604	0.16039641	1
Nrm	1.26682697	0.341219482	0.16060083	1
Msn	1.06669231	0.093144092	0.16071004	1
Slc35f6	0.9068565	-0.141053816	0.16071751	1
Limch1	0.92455979	-0.113161482	0.16088226	1
Rasl10b	0.90091981	-0.150529401	0.16095968	1
Slc35b4	1.07562886	0.105180368	0.16108379	1
Kctd16	0.85691802	-0.222770904	0.16113659	1
Atpif1	1.06744942	0.094167711	0.16114795	1
Ptges3l1	0.89666199	-0.157363859	0.16115818	1
Nop9	0.87268379	-0.196469092	0.16115896	1
Tmem88	0.80940761	-0.305061683	0.16117193	1
Hsd11b1	1.21236721	0.277826737	0.16139752	1
Aspn	1.08070767	0.111976326	0.16140021	1
Dnajc4	1.1927073	0.254240042	0.16155808	1
Lipt2	0.74927768	-0.416427615	0.16174365	1
Zbtb37	1.37696158	0.461488302	0.16186706	1
Trim23	1.07492198	0.104231951	0.16198854	1
Cul3	0.93668327	-0.0943668	0.16201487	1
Klhl4	1.26825055	0.342839785	0.16246935	1
St3gal1	1.21135215	0.276618333	0.16273992	1
Htr1d	1.1350624	0.182771606	0.1627921	1
Ube2r2	0.92035277	-0.11974115	0.16279653	1
Slamf6	0.65989025	-0.599701987	0.16285161	1
Hrc	0.65231922	-0.616349962	0.16285608	1
Ccnd2	0.8484432	-0.237110011	0.16289375	1
8-Sep	0.93653624	-0.094593274	0.16289552	1

Exd1	1.33463087	0.416440784	0.16298732	1
Zfp438	1.2297762	0.298395793	0.16308162	1
Psmb5	1.06993382	0.097521558	0.16319463	1
Rfwd2	0.91118938	-0.134177157	0.16325545	1
Gpr34	1.28610597	0.363009522	0.1635916	1
Itsn1	0.92018331	-0.120006811	0.16382606	1
Abcg3l1	1.14048198	0.189643646	0.16387532	1
Sgce	1.09731553	0.133978424	0.16394447	1
Gpbp1l1	1.09978966	0.137227631	0.16410827	1
Pou3f2	1.14644829	0.197171289	0.16420961	1
Crlf1	1.19532725	0.257405648	0.16422609	1
Cntn1	1.06742687	0.094137235	0.16424896	1
Napsa	1.27695933	0.352712572	0.16429449	1
Agmo	1.13867138	0.187351441	0.1643093	1
Tyrp1	1.06702837	0.09359853	0.16439623	1
Ptprv	0.71750381	-0.478941593	0.16440496	1
Smad1	0.90296252	-0.147261988	0.16445636	1
Rwdd2b	1.15527389	0.208234925	0.16458447	1
Dis3l	1.09914371	0.136380023	0.1645977	1
Elf4	1.28296441	0.359481153	0.16467791	1
Wdr73	0.85208799	-0.230925678	0.16470954	1
Atad2	0.88370131	-0.178369274	0.16477081	1
Glrx	1.06891567	0.096148034	0.16477524	1
Tesk2	1.17799801	0.2363371	0.1651562	1
LOC100910945	0.74995739	-0.415119471	0.16516998	1
Slc25a26	0.81681425	-0.291920065	0.16517523	1
Mlycd	0.89542224	-0.159359939	0.16530516	1
Fermt3	1.19853342	0.261270135	0.16533197	1
Gja1	0.91112703	-0.134275887	0.16559792	1
Rbx1	1.07275732	0.101323745	0.16560097	1
Pdia5	0.80751689	-0.308435662	0.16571436	1
Fam136a	1.15902076	0.212906413	0.16578253	1
Unc119b	0.91513805	-0.12793871	0.16588242	1
Gpr68	0.8948056	-0.160353811	0.1659042	1
Fastkd2	1.11918067	0.162442957	0.16591889	1
Mtmr6	1.06634405	0.092672993	0.16608732	1
Sox5	1.38271871	0.467507694	0.16621079	1
Rpa1	1.08943812	0.123584248	0.16630897	1
Vipas39	1.08365346	0.115903477	0.16640794	1
Ltb	1.30867204	0.388103594	0.16643628	1

Mmp23	1.24898441	0.320755467	0.16655643	1
Grcc10	1.07921136	0.109977446	0.16659489	1
Slc47a1	1.13291395	0.180038282	0.16664002	1
Kdr	0.90421563	-0.14526124	0.16690082	1
Maoa	1.07403696	0.103043643	0.16693318	1
Adam9	1.06810932	0.09505931	0.16698966	1
Fam219b	0.84716714	-0.239281459	0.1671426	1
Commd1	1.08294041	0.114953854	0.16726237	1
Slc35e1	0.90903931	-0.137585419	0.16727531	1
Mmp8	1.28134164	0.357655192	0.16733825	1
Smtn	0.9113133	-0.133980972	0.16745454	1
Phf10	1.13930382	0.18815253	0.16754357	1
Has2	1.31435257	0.394352327	0.16787946	1
Kif19	0.92455721	-0.113165504	0.16788588	1
Dnajc12	0.87629891	-0.190505029	0.16794013	1
Vps13a	0.93490192	-0.097113076	0.16807263	1
Sarm1	0.92782346	-0.108077775	0.16828627	1
Pdk2	1.08942169	0.123562492	0.16832336	1
Dhx16	0.8953728	-0.1594396	0.16836	1
Scaf1	0.93192949	-0.101707285	0.16845384	1
Ddx50	1.08367379	0.115930541	0.16855193	1
Rasgrf1	0.8862353	-0.174238297	0.16863486	1
Usp22	0.92573634	-0.111326735	0.16871397	1
Slc7a8	0.89809684	-0.155057081	0.16876252	1
Dynll1	0.93892621	-0.090916308	0.16883924	1
Syngr1	0.90557291	-0.143097293	0.16900142	1
Cpne6	1.06549452	0.091523169	0.16902215	1
Pip5k1c	0.93822396	-0.091995753	0.16917298	1
Cnot6	0.89851639	-0.154383278	0.16928619	1
B4galt2	0.83661955	-0.257356386	0.16939067	1
Atg13	1.0793372	0.110145649	0.16953765	1
Ano6	0.93082715	-0.103414805	0.1695603	1
RT1-T24-4	1.1160015	0.158338969	0.16985741	1
LOC100912041	1.180998	0.240006521	0.16991021	1
Pawr	1.20181251	0.265211842	0.17002541	1
Hpdl	0.83419584	-0.261541981	0.17008266	1
Cuedc2	0.87060442	-0.199910752	0.17008929	1
Npr1	0.84298196	-0.24642633	0.17016181	1
Ubtd1	0.88903358	-0.169690179	0.17056386	1
Abhd2	0.9346722	-0.097467614	0.17068946	1

B3gnt2	0.92789787	-0.107962078	0.17084286	1
Actr6	1.09523658	0.13124254	0.17093031	1
Ptprn2	0.9196308	-0.12087331	0.17101352	1
Gpr158	1.06564297	0.091724167	0.17111501	1
Zw10	1.12301878	0.167382055	0.17119795	1
Ctsw	0.66536549	-0.587781062	0.171245	1
Slc29a1	0.87495384	-0.192721196	0.171322	1
Bmp1	0.91887188	-0.122064373	0.17144545	1
Nmnat2	0.91222347	-0.132540798	0.17150314	1
Car5b	1.17193309	0.228890201	0.17152319	1
Pcbp3	0.93828468	-0.09190239	0.17153297	1
Mme	0.88916062	-0.169484043	0.17164774	1
Fbxo5	1.30212632	0.380869416	0.17165073	1
Cul9	0.91653054	-0.12574514	0.17183841	1
Atp9b	1.06745118	0.094170096	0.17189763	1
Ibsp	0.72642796	-0.461108361	0.1719536	1
Slc48a1	0.9230424	-0.11553118	0.17225948	1
Usp53	1.08275609	0.114708288	0.17227269	1
Tlr1	1.44982135	0.535875136	0.17230616	1
Syt6	0.8871011	-0.172829559	0.17234649	1
Tmem163	1.10418184	0.142977775	0.17235383	1
Fancl	0.82858837	-0.27127252	0.1727295	1
Lrp10	0.92856626	-0.106923231	0.17274774	1
Trim13	1.13996128	0.188984819	0.17284038	1
Ptk7	0.91167521	-0.133408142	0.17314273	1
Ttc7b	0.92438226	-0.113438519	0.17315721	1
Id2	1.14660677	0.197370698	0.17329159	1
Aifm1	1.09002351	0.124359255	0.17334812	1
Tsku	0.85881494	-0.219580809	0.17343123	1
Calm2	1.06803146	0.094954149	0.17357683	1
Kpnb1	0.93756867	-0.093003738	0.17357873	1
Rnmtl1	0.86232661	-0.213693698	0.17378696	1
Dag1	0.93875557	-0.091178526	0.17390828	1
Rab39a	0.88894318	-0.169836893	0.17396028	1
Rmnd1	0.89163443	-0.165475765	0.17405465	1
Srd5a3	1.11612049	0.158492781	0.1741147	1
Icmt	0.91268214	-0.131815592	0.17414752	1
Map4k4	0.93898457	-0.090826648	0.17423607	1
Samd11	0.84095648	-0.249896951	0.17424703	1
Pbld1	1.19444402	0.256339238	0.17439353	1

Tdg	0.91267891	-0.131820699	0.17462815	1
Ptbp3	1.09579008	0.131971445	0.17476066	1
Lin54	0.90157084	-0.149487234	0.1749255	1
Stbd1	1.08498425	0.117674094	0.1749659	1
Acot1	0.85270781	-0.229876631	0.17500199	1
Kdm4b	0.91935971	-0.121298656	0.17500267	1
Pigt	0.93290769	-0.100193761	0.1751727	1
Ghr	1.09455614	0.130345949	0.17521884	1
Cdkn2b	0.85946351	-0.218491705	0.17523108	1
Rnf14	0.93854278	-0.091505594	0.17525643	1
Ap1s3	0.82279279	-0.281398936	0.17528503	1
Slc51a	0.85366137	-0.228264196	0.17529852	1
Pak1	1.06879718	0.0959881	0.17543581	1
Tecr	1.06543983	0.091449115	0.17544032	1
Ncoa6	0.92522951	-0.11211682	0.17544166	1
Mrpl18	0.9153211	-0.127650162	0.17544588	1
Vps39	1.06741494	0.094121112	0.17552298	1
Poll	1.10967346	0.150135208	0.17559137	1
Cd24	0.93877099	-0.091154836	0.17559469	1
Tmed9	1.07367161	0.102552809	0.17582328	1
Flii	0.93933963	-0.090281213	0.17583602	1
LOC365238	1.18475344	0.244586849	0.17589792	1
Ankrd27	1.0753742	0.104838771	0.17590235	1
Dapk1	0.92546123	-0.111755548	0.17591247	1
Cep19	1.07239577	0.100837438	0.17612609	1
Zdhhc1	0.88925386	-0.169332762	0.17631409	1
Hs6st1	0.92692483	-0.109475749	0.17632105	1
Prkce	0.93003087	-0.104649496	0.17689832	1
Plekhh1	0.91886429	-0.122076298	0.17690938	1
Fbxo36	0.7696982	-0.377635226	0.17695075	1
Tlr4	1.17263846	0.229758276	0.17710661	1
Cytl1	1.15944311	0.213432031	0.17712946	1
Dolk	0.90684245	-0.141076164	0.17764207	1
Gab1	1.07176732	0.099991734	0.17776725	1
Ctbp1	0.93917073	-0.090540655	0.17780771	1
Slc25a10	0.89973749	-0.152423962	0.17785845	1
Olfml2b	0.90462618	-0.144606343	0.17797009	1
R3hdml	1.35828121	0.441782192	0.17798933	1
Cd14	1.24470961	0.315809202	0.1780739	1
Mtmr2	0.93916788	-0.09054502	0.17829845	1

Akap2	1.0661336	0.092388234	0.17836789	1
Klf12	0.81059879	-0.302940078	0.17844494	1
Alcam	0.93996392	-0.089322714	0.17845843	1
Celf2	0.88424569	-0.177480816	0.1785032	1
Fam69a	1.09188535	0.126821374	0.17850962	1
Pde7b	0.82761121	-0.272974916	0.17862838	1
Cdk5rap2	1.0973274	0.133994039	0.17865533	1
LOC100158225	0.79750489	-0.326434728	0.17887045	1
Marveld1	0.89896366	-0.153665296	0.17929672	1
Adgre1	1.13026845	0.176665467	0.17951662	1
Nap1l3	0.92913673	-0.106037186	0.17962904	1
Gsk3b	0.92573668	-0.111326204	0.17963074	1
Defa5	1.2701187	0.344963327	0.17981816	1
LOC100361083	0.88062674	-0.183397439	0.17983623	1
Plec	0.93920053	-0.09049488	0.18000406	1
Rpe	1.08882532	0.122772515	0.18015696	1
Fgf12	1.0721965	0.100569325	0.1801967	1
Kdm4c	1.10288705	0.141285054	0.18021959	1
Hs6st2	0.90848314	-0.138468348	0.18053107	1
Cacna1c	0.91077601	-0.134831803	0.18080983	1
Uqcr10	1.07269365	0.101238112	0.18088046	1
Cbarp	0.92176474	-0.117529512	0.18093695	1
Dhcr24	0.93846899	-0.091619022	0.18094864	1
Neto2	0.87395745	-0.194365048	0.18101171	1
Necab3	1.06708907	0.093680604	0.18106484	1
Apool	1.11873588	0.16186947	0.18111061	1
Sin3b	0.90809466	-0.139085404	0.18120413	1
Lnc134	1.272889	0.348106622	0.1812519	1
Dusp8	0.87672494	-0.189803805	0.18129975	1
Mapk1ip1l	0.89411485	-0.161467937	0.18195773	1
Alkbh2	1.2695836	0.344355393	0.18233821	1
Top1	0.90720966	-0.140492092	0.18239447	1
Map1lc3b	1.06505771	0.090931601	0.18281512	1
Hcfc1	0.93559161	-0.09604917	0.18289547	1
Fut11	0.84929024	-0.235670428	0.18294858	1
Dpysl5	0.93926725	-0.090392394	0.18318904	1
Fech	1.08621093	0.119304285	0.18323152	1
Sdhd	1.06754921	0.094302574	0.18334893	1
Chchd4	1.14563133	0.196142846	0.18346294	1
Nle1	0.84053473	-0.250620663	0.18353672	1

Pik3r2	0.92159761	-0.117791113	0.18363431	1
Clvs1	1.10991759	0.150452565	0.18364294	1
Vps52	0.92771112	-0.108252463	0.18373601	1
Ublcp1	1.06405913	0.089578318	0.18373717	1
Gss	1.10111881	0.138970148	0.18380403	1
Stk11ip	0.88910384	-0.169576168	0.18386038	1
Cyfip2	0.94054357	-0.088433317	0.18390272	1
Ino80d	0.82293966	-0.281141437	0.18400161	1
Atp6v1e1	1.06332121	0.088577472	0.18412974	1
Ptpn2	1.16945998	0.225842498	0.18420292	1
Tnip2	0.85007853	-0.234331964	0.1842289	1
Foxo3	0.89927034	-0.153173201	0.18425272	1
Cep44	1.17781453	0.236112375	0.18427642	1
Dvl3	0.89227812	-0.164434637	0.18449678	1
Mnat1	1.09198884	0.126958115	0.18456676	1
Phf8	0.92356435	-0.114715611	0.18465224	1
Kcnq2	0.93343489	-0.0993787	0.18482816	1
Sipa1l2	0.9229634	-0.115654649	0.18521762	1
Thsd1	0.85498633	-0.226026748	0.18524809	1
Soga3	0.94108623	-0.08760117	0.18525146	1
Gtf2ird2	1.27250933	0.347676234	0.18525392	1
Armc10	1.09509314	0.131053576	0.18530103	1
LOC684871	0.85656141	-0.223371407	0.18538967	1
Rbm11	1.07136915	0.099455662	0.18553812	1
Wdr46	0.88829235	-0.170893534	0.18558751	1
Map2k6	1.13734871	0.185674654	0.18564148	1
Celf5	0.8658836	-0.207754989	0.1857176	1
Dnajc28	1.18132758	0.240409079	0.18610542	1
Msh3	1.11598073	0.158312113	0.18611111	1
Mmp19	1.24178861	0.31241961	0.1864094	1
Phyh	1.07512948	0.104510411	0.18645479	1
Creb3l2	0.90177184	-0.149165633	0.18645627	1
Tmem140	1.23238997	0.301458844	0.18653129	1
Sec24b	0.92558132	-0.111568352	0.18657639	1
Sdsl	0.75535231	-0.40477839	0.18666908	1
Scmh1	0.8788228	-0.186355793	0.18668565	1
Gramd1a	0.93028782	-0.104250962	0.18673453	1
Ccdc61	1.18511822	0.245030986	0.18673791	1
Parva	1.08855073	0.122408643	0.18675553	1
Rhbdf1	0.91403529	-0.129678223	0.1867719	1

Mpi	1.08097808	0.112337264	0.18682149	1
Col27a1	0.88537297	-0.175642761	0.18703154	1
Apba3	0.87622618	-0.190624767	0.18729436	1
Pcdhga7	1.1169586	0.159575708	0.18731896	1
Spef1	0.87247615	-0.196812404	0.18733821	1
Dpp10	1.06345435	0.08875811	0.18748857	1
Mapk14	0.92519682	-0.11216778	0.18752969	1
Phf19	0.76719675	-0.382331485	0.18753039	1
Pdxk	0.81656406	-0.292362017	0.18757341	1
Map7d1	0.9395304	-0.089988248	0.18757866	1
Ngfr	0.93992586	-0.089381126	0.18764962	1
Dpy19l1	0.93000352	-0.104691925	0.18765773	1
Htr7	0.7927103	-0.335134379	0.18766792	1
Ccndbp1	1.08522822	0.117998473	0.18769379	1
Mlf1	1.1970272	0.259455932	0.18777542	1
Nfasc	0.94109627	-0.087585778	0.18779351	1
Atp6v1d	1.0630417	0.088198197	0.18780874	1
Acyp1	1.12045423	0.164083715	0.18783992	1
Nppa	1.27628479	0.351950291	0.18793693	1
Fam220a	0.90506494	-0.143906778	0.18804313	1
Cdh2	0.9393864	-0.09020938	0.18812142	1
Zfp14	0.8611712	-0.215628019	0.18813468	1
LOC106631776	0.84649866	-0.240420319	0.18829554	1
Ubr2	0.93657538	-0.094532984	0.18834221	1
Kank4	0.92854896	-0.106950112	0.18837967	1
Ccdc80	0.92546901	-0.11174342	0.18838884	1
Mapk4	0.9028364	-0.147463513	0.1885312	1
Ston2	1.20682773	0.271219754	0.1885435	1
Diras1	0.9285998	-0.106871117	0.18857771	1
Cklf	1.36799194	0.452059727	0.18860058	1
Hoxc9	2.01499362	1.010775268	0.18871364	1
Epc1	1.09456057	0.130351793	0.18876727	1
Thoc1	1.11907707	0.162309392	0.18877605	1
Megf6	1.18890695	0.249635801	0.18889046	1
Prrx1	1.19824533	0.26092332	0.18889653	1
Myo5b	0.85347385	-0.228581143	0.18892075	1
Ndufa11	1.08198692	0.113683061	0.18892409	1
Dixdc1	0.88782407	-0.171654277	0.18893395	1
Mrps16	0.89957855	-0.152678835	0.18896087	1
Idua	1.09505024	0.130997055	0.18897716	1

Megf9	0.93933011	-0.090295844	0.18911251	1
Rer1	0.92816178	-0.107551804	0.18919967	1
Hprt1	1.06824096	0.095237104	0.1892432	1
Panx1	0.88792097	-0.171496819	0.18926359	1
Ggt7	0.93908199	-0.090676966	0.18944273	1
LOC499770	0.88420315	-0.177550221	0.18947273	1
RGD1563941	1.31063236	0.390263058	0.18953031	1
Appbp2	0.9332122	-0.099722922	0.18954133	1
Stk40	0.9178535	-0.1236642	0.1895704	1
Slf1	1.13755912	0.185941526	0.18966139	1
Nmral1	0.83712685	-0.256481843	0.18987851	1
Rad52	1.17968805	0.238405407	0.18989446	1
Spryd4	1.15009646	0.201754871	0.18997238	1
RT1-Db2	1.29395442	0.371786796	0.1900724	1
Snrpa1	1.13189668	0.178742272	0.19014119	1
Arhgap29	1.09622373	0.132542269	0.19014946	1
Lyrm7	1.11263109	0.153975325	0.19018818	1
Gria1	0.77215622	-0.37303534	0.19026926	1
Cc2d1a	0.90682586	-0.141102566	0.19043561	1
Gng2	0.94162547	-0.086774746	0.19052391	1
Ppp2r3c	1.09258249	0.127742211	0.19074341	1
Brpf3	0.82122436	-0.284151668	0.19086672	1
Serpine1	0.8660944	-0.207403814	0.19092602	1
Galnt16	1.17049839	0.227122944	0.19106652	1
Pla1a	1.24879554	0.320537284	0.19108382	1
Rasd1	0.81373663	-0.297366158	0.19117267	1
Cacng4	0.80074351	-0.320587896	0.19128922	1
Megf8	0.93997163	-0.089310883	0.19143819	1
Arl10	1.28933825	0.366630796	0.19146705	1
Slc2a4	1.29245678	0.37011604	0.19148172	1
Tubgcp5	1.08375583	0.116039748	0.19152754	1
Minpp1	1.08768086	0.121255316	0.19158016	1
Pcdh17	0.906884	-0.141010068	0.19164725	1
Dram2	0.90168753	-0.149300529	0.19173182	1
Dhx38	1.09818251	0.135117846	0.19173932	1
Borcs6	0.90274766	-0.147605326	0.19181461	1
Shroom4	1.1915875	0.252884893	0.19204109	1
Wbscr16	1.16076282	0.215073218	0.19211658	1
RGD1562987	1.06487887	0.090689328	0.19224424	1
RGD1560108	0.90810346	-0.139071416	0.19234212	1

Kcnk4	0.85995056	-0.217674368	0.19253948	1
Crls1	0.89488613	-0.160223984	0.19261281	1
Pced1b	1.19546347	0.25757004	0.19266094	1
Decr1	1.10796267	0.14790927	0.19270979	1
Cpq	1.07396042	0.102940828	0.19274024	1
B3gnt5	0.87197606	-0.197639562	0.19276355	1
Ankrd34b	1.19850301	0.261233538	0.19289048	1
Ankrd54	0.84274649	-0.246829383	0.19291504	1
Gtf2ird1	0.88939407	-0.169105304	0.19300708	1
Zfp68	1.07968684	0.110612924	0.19304223	1
Rbms1	0.9186724	-0.12237761	0.19309102	1
Slc7a6os	1.12547176	0.170529855	0.19312414	1
Med11	1.19481291	0.256784734	0.19316935	1
Nfyb	1.1066169	0.146155864	0.1932165	1
P2ry10	1.31530741	0.395400026	0.19323999	1
Fgfr2	1.14677757	0.197585597	0.19325143	1
Fkbp7	0.87548117	-0.19185195	0.19337952	1
Tmcc2	1.08456134	0.117111645	0.19346611	1
Cdh8	1.20704183	0.27147567	0.19361714	1
Atg9a	0.93599072	-0.095433868	0.19365952	1
Igbp1	1.08865509	0.122546947	0.19367711	1
Cep83	1.0779687	0.108315293	0.19368033	1
Ppdpf	0.92035701	-0.119734493	0.19372208	1
Crym	0.88364585	-0.178459816	0.19373853	1
Tk2	1.11025398	0.150889742	0.19398903	1
Prss12	1.07839311	0.108883186	0.19405	1
Astn2	0.89846986	-0.154457979	0.19407582	1
Akr1b10	0.87213746	-0.19737256	0.19411324	1
Myt1	0.91430755	-0.129248567	0.19428326	1
Numbl	0.90820075	-0.138916873	0.19432789	1
Tcf7l1	0.86333399	-0.212009312	0.19436725	1
Slitrk4	0.91674992	-0.125399855	0.19452266	1
Ddx18	1.11407293	0.155843683	0.19472124	1
Gpr4	0.80606499	-0.311031928	0.19474017	1
Repin1	1.14575641	0.19630036	0.1951552	1
Eif3e	1.07089179	0.098812705	0.19524055	1
Papola	0.93905273	-0.090721928	0.19539712	1
Rtn4rl2	0.88075596	-0.183185767	0.19559674	1
Gabbr1	0.94142823	-0.087076983	0.19561961	1
Umps	1.09830058	0.135272936	0.19567533	1

Pcdha2	0.87049659	-0.200089452	0.19570065	1
Ccnd3	0.92104995	-0.11864869	0.1958005	1
Ice2	1.14807165	0.199212679	0.19604025	1
Wdr89	0.82893603	-0.270667328	0.19609811	1
Map1a	0.9388134	-0.091089655	0.19616739	1
Prelid3b	1.08513227	0.117870906	0.19620765	1
Syt7	0.90971173	-0.136518646	0.19633908	1
Tlr8	1.20149719	0.264833271	0.19635156	1
Sec16b	1.16086838	0.215204407	0.19640001	1
Map4	0.94182225	-0.086473286	0.19643872	1
Tm2d1	1.08750091	0.121016609	0.19664644	1
Dcc	0.6437629	-0.635398657	0.19669244	1
Pim3	0.91902295	-0.121827212	0.19705506	1
Isoc2b	1.21509519	0.281069339	0.19707601	1
Psmb4	1.06217374	0.087019773	0.19709387	1
Trak1	0.93930661	-0.090331935	0.19712004	1
Foxp4	0.86019502	-0.217264323	0.1972481	1
Cluh	0.94085086	-0.087962041	0.19749881	1
Tcirg1	1.10524315	0.144363793	0.19749969	1
Stx4	1.10272537	0.141073536	0.19751598	1
Islr	1.06561749	0.091689669	0.19753585	1
Mat2a	0.92952053	-0.105441363	0.19759642	1
Atp2a1	0.80449176	-0.313850446	0.19772583	1
Slc22a3	1.27431233	0.349718921	0.19782733	1
Ttbk1	0.91167093	-0.133414917	0.19783653	1
Fance	0.87541361	-0.19196328	0.19783926	1
B4galt6	1.08114633	0.112561804	0.19789816	1
Katnal1	0.88862157	-0.17035894	0.19793056	1
Klf13	0.79286021	-0.334861566	0.19810648	1
Eif3i	1.08915969	0.12321549	0.19826464	1
Pdlim2	1.1778159	0.236114054	0.19850914	1
Pde6b	1.32514029	0.4061451	0.19863823	1
Mboat4	0.74120579	-0.432053946	0.19923658	1
Pfdn2	1.07849343	0.109017388	0.19923721	1
Trpm7	0.94031419	-0.08878521	0.199273	1
Rtel1	0.92137667	-0.118137024	0.1994256	1
Hmgcs1	0.94017792	-0.088994293	0.19947926	1
Ptger3	0.79137327	-0.337569749	0.19954357	1
Rprm	1.08218826	0.113951497	0.19970147	1
Adcy1	0.88353214	-0.178645481	0.1998542	1

Isca1	1.06230497	0.087197994	0.19989685	1
Epn1	0.93433213	-0.097992613	0.19989914	1
Flrt1	1.20882238	0.273602279	0.19991607	1
Pnp	1.08032033	0.111459156	0.20006809	1
Fgl2	0.94249592	-0.085441728	0.20050127	1
Aox1	1.0832193	0.115325346	0.20057097	1
Mknk2	0.92850669	-0.107015791	0.20062937	1
Cnppd1	0.92379317	-0.114358211	0.20091286	1
Nkain3	1.37678229	0.461300441	0.20098988	1
Kremen1	1.18714064	0.247490859	0.20106253	1
Mrpl48	1.07704549	0.107079191	0.20112803	1
Ccdc137	1.12255889	0.166791128	0.20117896	1
Kitlg	1.0618675	0.086603762	0.20118272	1
Th	1.36412093	0.447971551	0.20134007	1
Hoxd1	0.90811969	-0.139045643	0.20137063	1
Pbdc1	1.10858901	0.148724615	0.2015016	1
Spry2	0.87593818	-0.191099041	0.20160601	1
Acadm	1.06673101	0.093196429	0.20172396	1
Skp2	0.81825027	-0.289385927	0.20186769	1
Irf8	1.28591657	0.362797047	0.20190419	1
Grpel1	1.07930939	0.110108475	0.20223662	1
Cr2	1.59069112	0.669653724	0.20227796	1
Ddx19a	1.09035938	0.124803724	0.20229841	1
Slc29a4	0.85461475	-0.226653875	0.20233208	1
Rnf112	0.93278559	-0.100382586	0.20245126	1
Oplah	0.89064856	-0.167071829	0.20246098	1
Zcchc12	1.1218785	0.165916439	0.20276724	1
Strn	0.91927761	-0.121427496	0.20278898	1
Tbc1d20	0.89499891	-0.160042173	0.20286423	1
Usp39	1.10813317	0.148131263	0.20306282	1
Gabbr2	0.94289164	-0.084836119	0.20331688	1
Fry	0.94241575	-0.085564444	0.20335742	1
Uqcrh	1.06174099	0.086431868	0.20340296	1
Srgap3	0.93455892	-0.097642472	0.20341389	1
Srgap2	1.09307521	0.128392671	0.20356877	1
Tm7sf2	0.90944259	-0.136945525	0.20363203	1
Sgsm1	0.93036484	-0.104131518	0.20363991	1
Psmd4	1.06326018	0.088494667	0.20367328	1
Grm7	0.90977433	-0.136419365	0.20367411	1
Eif1	0.94339809	-0.08406142	0.20370871	1

Rcn3	0.92145328	-0.118017074	0.20371441	1
Serp1	0.9407328	-0.088143095	0.20379303	1
S100a13	1.10293675	0.14135006	0.20384033	1
RGD1311805	1.06939148	0.096790088	0.20390382	1
Ndufa10	1.0624129	0.087344563	0.20392863	1
Lace1	0.83567079	-0.258993394	0.2039705	1
Ahcyl2	1.06176937	0.086470425	0.20398212	1
Jakmip2	1.07891635	0.109583014	0.20401264	1
Mapk8ip3	0.94319428	-0.084373123	0.20406175	1
Slc25a27	1.09009225	0.124450235	0.20415817	1
Lman2	1.08699436	0.120344461	0.20424845	1
Twf1	0.93534419	-0.096430751	0.2042583	1
B4galt1	0.89575646	-0.158821559	0.20458831	1
Lpl	1.13353677	0.18083119	0.20473388	1
Tpst1	1.11030943	0.150961788	0.20479717	1
Tmem259	0.92005675	-0.120205251	0.20492771	1
Afap1l2	1.0708291	0.098728256	0.20500935	1
Zfp608	1.1154587	0.157637098	0.20504335	1
Pex19	1.06912374	0.096428841	0.20511364	1
Ncoa4	1.05994516	0.083989627	0.20524472	1
Slc16a4	1.24215031	0.312839758	0.20534224	1
Nr4a1	0.92265583	-0.116135508	0.20538327	1
Acat1	1.0772542	0.107358727	0.20548737	1
Gk	1.0811066	0.112508781	0.20573909	1
Ccdc117	0.88202408	-0.181110056	0.20589972	1
Nkap	1.13148917	0.17822278	0.20597385	1
Lsamp	0.9051736	-0.143733581	0.2060195	1
Peli2	0.9023911	-0.148175254	0.20603695	1
RGD1566265	0.89207591	-0.164761618	0.20612267	1
Ncdn	0.94271878	-0.085100627	0.20626528	1
Grn	0.93332326	-0.099551242	0.20682101	1
Ralgds	0.9285251	-0.106987192	0.20683337	1
Gtf2b	1.09087438	0.125484982	0.20685462	1
Aff1	0.88937537	-0.169135639	0.20685742	1
Sema5b	0.81150532	-0.301327536	0.20709252	1
Pth1r	1.13052321	0.176990613	0.2074286	1
Avpr1a	0.76996717	-0.37713117	0.2074592	1
Susd3	1.32321463	0.404047096	0.20745934	1
Acap2	0.92222971	-0.116801955	0.20751577	1
Zcchc4	1.20207985	0.26553273	0.20752789	1

Slc22a15	1.15529106	0.208256364	0.20765965	1
Dlat	0.93885085	-0.091032113	0.20766202	1
Tuft1	0.85986426	-0.217819167	0.20791764	1
Car6	1.37862168	0.463226605	0.20815397	1
Aen	0.86481593	-0.209534998	0.20822638	1
Colq	0.804349	-0.314106479	0.2084019	1
Kcna1	0.94193712	-0.086297338	0.20843151	1
MGC95210	0.8462127	-0.240907764	0.20848873	1
Nxt1	0.83416892	-0.261588538	0.20850558	1
Ikbke	1.1492015	0.200631779	0.20852449	1
Pspc1	0.91398019	-0.129765201	0.20853773	1
Cndp2	1.06211775	0.086943713	0.20860777	1
Cox20	0.89572904	-0.158865718	0.2086095	1
Anp32a	1.0785715	0.109121823	0.20873163	1
Pax9	1.22877332	0.297218802	0.20882754	1
Ppp1r3d	0.91241805	-0.132233103	0.20883607	1
Adcy7	0.90322308	-0.146845741	0.20910297	1
RGD1304884	0.94405283	-0.0830605	0.2091571	1
Sphkap	1.07618911	0.105931614	0.20918823	1
Epn2	0.94207578	-0.086084988	0.20923384	1
RGD1308117	0.85678834	-0.222989244	0.2092437	1
Fdxacb1	0.82511388	-0.277334849	0.20925646	1
Arhgef15	0.81209801	-0.300274249	0.20970665	1
Cabp7	1.18549458	0.24548907	0.20973183	1
Fam189b	0.94385485	-0.083363086	0.20994192	1
Tgoln2	1.05936519	0.083200003	0.21018073	1
Arhgef37	0.89118833	-0.166197759	0.21048034	1
Emp2	0.92530105	-0.112005273	0.21050567	1
Mbd3	0.9195759	-0.120959441	0.21067955	1
Slc36a2	1.1069893	0.146641274	0.21097007	1
Zdhhc3	1.07774621	0.108017491	0.21097401	1
Hddc2	1.13092133	0.177498575	0.21122757	1
A3galt2	0.94264479	-0.085213864	0.21134243	1
Me3	0.88698121	-0.173024548	0.21139296	1
Tmc7	0.90472455	-0.144449468	0.21149889	1
Dennd5a	0.94447816	-0.082410656	0.21167599	1
Cacng7	0.91221159	-0.132559598	0.21180239	1
Tprn	0.82654356	-0.27483724	0.21190194	1
Man2a1	0.92007148	-0.120182144	0.21190421	1
Glod4	1.06690887	0.093436958	0.21192642	1

Mrc1	1.08250865	0.114378547	0.21193117	1
Slc2a10	0.79670415	-0.327884006	0.21194985	1
Nr2f1	0.91473883	-0.128568195	0.21197306	1
Abhd8	0.93491811	-0.097088087	0.21197929	1
Mrps17	1.10770551	0.147574384	0.21202705	1
Slco1c1	0.86744491	-0.205155956	0.21208101	1
Plekha5	0.93487843	-0.09714933	0.21229093	1
Rnf113a2	0.86512103	-0.209026112	0.21255217	1
Ccng2	1.08728814	0.120734315	0.2127813	1
Mxd4	0.90380499	-0.145916578	0.21303133	1
Meis2	1.11342846	0.155008867	0.21309116	1
Bckdk	1.08684475	0.120145875	0.21353408	1
Tmem150c	0.93930126	-0.090340156	0.21356204	1
Supt4h1	1.09058296	0.125099517	0.21385376	1
Stc1	0.81167777	-0.301020997	0.21392019	1
Kcnip1	0.93353288	-0.099227255	0.21392211	1
Lrrc9	1.23317312	0.30237535	0.21396559	1
Ubxn11	0.77655803	-0.364834353	0.21428122	1
Cradd	1.2969011	0.375068463	0.21431392	1
Uqcc1	1.08231808	0.11412455	0.21455289	1
Ebpl	1.14327099	0.193167404	0.21469714	1
Sufu	0.8242666	-0.278817054	0.21481126	1
Ciapin1	1.07411551	0.103149148	0.21482544	1
Stat4	0.73479333	-0.444589568	0.21484541	1
Mccc1	1.11581027	0.15809173	0.21497935	1
Mal	0.94493732	-0.081709465	0.21506353	1
Clec4a3	1.19897266	0.261798759	0.21510725	1
Tbck	0.90726898	-0.140397761	0.21516292	1
RGD1305733	1.05991567	0.083949478	0.21522894	1
Rnf208	0.9014793	-0.149633733	0.21529841	1
Ace	1.14442793	0.194626615	0.21531331	1
Glg1	0.94453702	-0.082320757	0.21541594	1
Arhgap26	0.83408652	-0.261731045	0.21548792	1
Ddx54	0.91760402	-0.124056377	0.21550428	1
Shc4	0.91476863	-0.128521197	0.21570863	1
Arhgef28	0.92464229	-0.113032749	0.21577836	1
LOC500077	1.21680356	0.283096278	0.21579109	1
Tnfaip2	1.16734067	0.223225653	0.21595667	1
Stx18	1.08672626	0.119988576	0.2160426	1
Hs3st1	1.18948195	0.250333378	0.2164706	1

Oprl1	1.08091182	0.112248831	0.21674384	1
Map7	0.93828964	-0.091894761	0.2170744	1
Nkr-p1c	1.53115982	0.614624881	0.21727953	1
Lamtor3	1.08649893	0.119686753	0.21746725	1
Ptpn12	0.93134203	-0.102617003	0.2175688	1
Ndufs1	1.05832257	0.081779417	0.21760038	1
Fam227b	1.32481727	0.405793383	0.21769734	1
Neu3	1.26720469	0.341649585	0.21772742	1
Dnmt3b	0.76423261	-0.387916269	0.21773259	1
Tmem205	1.09987644	0.13734146	0.21776562	1
Rab38	1.21851933	0.285129133	0.21800395	1
Dolpp1	0.91515351	-0.127914331	0.21817049	1
Arl3	1.07111177	0.099109034	0.21817269	1
Cep95	1.11547203	0.157654341	0.21833468	1
Pdzd7	1.1534701	0.205980613	0.21843716	1
Trappc1	1.06502402	0.090885964	0.21851575	1
Trpm2	0.89217727	-0.164597699	0.2188761	1
Akap7	0.80894542	-0.305885727	0.21900833	1
Ccnb1	0.84636025	-0.240656221	0.21982073	1
Slc22a17	0.94454137	-0.082314113	0.21990856	1
Plekhd1	0.93961634	-0.089856298	0.21991667	1
Eif4a2	1.06055358	0.084817505	0.22005055	1
Cyb5r2	1.12403968	0.168692961	0.22008178	1
Mefv	1.25823714	0.331403847	0.22022324	1
Tgm2	1.07752756	0.107724765	0.2203348	1
Slc27a6	1.20401893	0.267858072	0.22035835	1
Cdadc1	1.08559854	0.118490682	0.22044324	1
Adck5	0.8813226	-0.182257888	0.22055764	1
Snrpn	1.0785832	0.109137469	0.22097118	1
E2f3	1.18009954	0.238908556	0.22119103	1
Mal2	0.86348485	-0.211757227	0.22134412	1
Gimap7	0.79433119	-0.332187442	0.22145341	1
Cdk5r1	0.87037304	-0.200294233	0.2215575	1
Kif16b	1.06817872	0.095153045	0.22161701	1
Ndufaf6	1.1656966	0.221192346	0.22163569	1
Taf2	0.92639849	-0.110295198	0.22167031	1
Lig3	1.0936214	0.129113379	0.22174727	1
Rnf181	1.06764014	0.094425447	0.22180985	1
Ppp4r1	0.92371716	-0.114476929	0.22186564	1
Arnt2	0.92356095	-0.114720917	0.22199279	1

Zwint	1.06071852	0.085041859	0.22210071	1
Hars	1.05958109	0.083493997	0.2222614	1
Zbed3	1.20781329	0.272397455	0.22227974	1
Hhip	1.33561831	0.417507779	0.22247279	1
Ccl21	0.89254844	-0.163997619	0.22264498	1
LOC308990	1.30923554	0.388724668	0.22266429	1
Vdac1	1.05796089	0.081286292	0.22282452	1
Pqlc3	1.06677972	0.093262302	0.22286078	1
Hmx1	1.36560242	0.449537519	0.22324071	1
Cd59	1.05840156	0.08188709	0.22340622	1
Gucd1	0.83384268	-0.262152883	0.22373316	1
Rgs1	1.20321814	0.26689822	0.22389876	1
Chml	0.83446485	-0.261076815	0.22395793	1
Dqx1	0.83922776	-0.252865692	0.22436525	1
Vkorc1	0.90083467	-0.15066575	0.22462995	1
Mfap5	0.9318552	-0.101822296	0.22495028	1
Slc35a3	0.90214582	-0.148567443	0.22496336	1
Vwa5a	1.05899756	0.082699268	0.22501666	1
Baz1b	0.93971479	-0.089705146	0.22505841	1
Pwwp2a	1.11859672	0.161690009	0.22508138	1
Cables1	0.89549276	-0.159246333	0.22519935	1
Fndc1	0.89620455	-0.158100046	0.22520445	1
RGD1560436	0.75690216	-0.401821264	0.22529928	1
Numb	0.91125033	-0.134080657	0.22551868	1
Oprk1	0.87811075	-0.187525181	0.22552015	1
Slc45a3	0.81256823	-0.299439144	0.22566431	1
Prdx1	1.05975345	0.083728667	0.22567708	1
Mthfs	0.86169164	-0.214756412	0.22571312	1
Ufsp2	1.07512586	0.10450556	0.22616605	1
Uggt1	0.93007159	-0.104586322	0.22628245	1
Cerk	0.94207485	-0.086086412	0.2263063	1
Snx13	1.06904148	0.096317838	0.22640098	1
Zbtb26	1.18898672	0.249732604	0.22647445	1
Esm1	1.16373887	0.218767371	0.22653999	1
Tvp23a	0.89287645	-0.163467536	0.22657054	1
Casp4	1.1309485	0.177533236	0.22662335	1
F2rl2	0.89578482	-0.158775874	0.22665645	1
Shtn1	0.91517202	-0.127885151	0.22668277	1
Slc35c1	0.90830936	-0.138744349	0.22668516	1
Blvrb	1.11245488	0.153746822	0.22676983	1

Exosc8	1.19572213	0.25788217	0.22683635	1
Spef2	1.42080014	0.506703632	0.22693779	1
Rpl17	0.94344685	-0.083986847	0.22708193	1
Traf4	0.82379742	-0.279638491	0.22716282	1
Mfsd12	0.90972245	-0.136501646	0.22717274	1
Pan2	0.89598352	-0.158455896	0.22717364	1
St6galnac6	0.92723443	-0.108993953	0.22718472	1
Dus4l	0.77229017	-0.372785081	0.22719994	1
Xpnpep1	0.93763392	-0.092903331	0.2272311	1
Rhoj	1.0873701	0.120843067	0.22727414	1
Mfng	1.28819123	0.365346777	0.22735057	1
Pla2g5	1.14807743	0.199219944	0.22736747	1
Agl	1.05758035	0.08076728	0.22756376	1
Pold2	0.86552519	-0.208352282	0.22773448	1
Tlcd1	0.87103411	-0.199198886	0.22800927	1
PCOLCE2	1.09176553	0.126663058	0.22821064	1
Tecpr1	0.93163214	-0.102167691	0.22822932	1
Tmem130	0.94635372	-0.079548575	0.22835341	1
Snx33	1.11029302	0.150940466	0.22840933	1
Pon3	0.79095892	-0.338325335	0.22844206	1
Cdk5r2	0.93683067	-0.094139788	0.22846228	1
Csnk1g2	0.94094434	-0.087818707	0.22850083	1
RT1-A2	1.06757077	0.094331709	0.22858407	1
Setd2	0.93696699	-0.093929877	0.22859275	1
Shank3	0.90627598	-0.141977645	0.22859643	1
Bcap31	1.05695827	0.079918423	0.22880995	1
Ptgfrn	0.93226984	-0.101180506	0.22882524	1
Wdr5	0.91250962	-0.13208833	0.22891164	1
Gna12	0.94321596	-0.084339964	0.22905975	1
Cog3	0.91682936	-0.125274845	0.22908772	1
Fyn	0.9338093	-0.098800143	0.22911156	1
Gaa	0.94465953	-0.082133643	0.22916651	1
Thg1l	1.20189699	0.265313252	0.22934693	1
Alox5ap	1.1768757	0.234961956	0.22936219	1
Rnf38	0.88007627	-0.18429953	0.22936451	1
Tmem9	0.92189789	-0.117321136	0.22948523	1
Cyp51	0.94587845	-0.080273289	0.22955211	1
Cep120	1.07251082	0.100992204	0.22960623	1
Cox7b	1.05869995	0.08229377	0.2297285	1
Shc2	0.93519176	-0.096665884	0.22972892	1

Cpa2	0.73282391	-0.448461517	0.22994607	1
Alad	1.11304744	0.154515089	0.23000544	1
Xrcc2	1.20941275	0.27430669	0.23004883	1
Fcgr2b	1.23638514	0.306128224	0.23005801	1
Psen1	1.08968728	0.123914172	0.23008823	1
Tnc	0.92843549	-0.107126423	0.23009419	1
Noc4l	1.12785798	0.173585412	0.23030426	1
Upp1	0.77721079	-0.363622161	0.23040882	1
Rbbp9	1.16633291	0.221979638	0.23049902	1
Lztr1	0.93788735	-0.092513437	0.23054828	1
Tjp1	1.05939922	0.083246356	0.23090475	1
Ptpn14	0.85876456	-0.219665442	0.23098451	1
G3bp1	0.94363044	-0.083706141	0.23116136	1
Cnnm4	0.92420967	-0.113707913	0.23132524	1
Cops5	1.0582653	0.081701353	0.23145518	1
RGD1305645	1.14334916	0.193266041	0.23154187	1
Ankib1	0.93792545	-0.092454843	0.23156733	1
Rec114	1.36702905	0.451043897	0.23161121	1
Pdgfc	1.30677153	0.386006932	0.23178825	1
Pcdhga10	0.90066972	-0.150929936	0.23183635	1
Frmd4a	0.91240394	-0.132255423	0.23193378	1
Tank	1.0977323	0.134526273	0.23202504	1
Mroh1	0.88317977	-0.179220977	0.2320832	1
Smim8	1.1164931	0.158974338	0.23223752	1
Rgl3	1.23225243	0.30129782	0.23230083	1
Nmb	1.08236428	0.114186136	0.23243064	1
Serpini1	1.06628906	0.092598593	0.23245788	1
Slc25a18	0.76761721	-0.381541044	0.23249908	1
Spata7	1.11334839	0.154905112	0.23262292	1
Dpy19l4	0.91790532	-0.12358274	0.23269045	1
Dcp1a	1.20438176	0.268292758	0.23273506	1
Dact2	1.12619781	0.171460248	0.23274623	1
Ylpm1	0.93222038	-0.101257037	0.23293496	1
Ntm	1.11796379	0.160873456	0.2330403	1
Agap1	0.92902722	-0.106207226	0.23330773	1
Doc2a	1.12920686	0.175309794	0.23332212	1
Rptor	0.93596327	-0.095476182	0.23337197	1
C1galt1	0.8782628	-0.187275405	0.23342562	1
Sez6	0.90803727	-0.139176581	0.23352781	1
Ahr	0.91667026	-0.125525234	0.23357125	1

Fah	1.15739042	0.210875604	0.23365539	1
Enah	0.93426494	-0.098096366	0.23367844	1
Bcor	1.11106023	0.151937024	0.23368193	1
Lims1	1.09098265	0.125628152	0.23369447	1
Gab2	0.9072853	-0.140371813	0.23369845	1
Dcun1d3	0.88100596	-0.182776313	0.23373201	1
Nell1	0.94066924	-0.088240559	0.23378156	1
Gba2	1.07942423	0.110261977	0.23402222	1
Abhd14a	1.13787437	0.186341276	0.23407577	1
Fbxo22	1.08281454	0.114786166	0.23410617	1
Rnf10	0.94647449	-0.079364479	0.23433171	1
Mrps22	1.09137354	0.126144965	0.23438709	1
Actn3	0.77538478	-0.36701568	0.23438926	1
Ccs	0.904088	-0.145464889	0.23443348	1
Ifitm1	1.12263061	0.166883307	0.2344462	1
Sgtb	1.06856947	0.095680698	0.23455457	1
Mt2A	1.19019157	0.251193804	0.23464756	1
Csf1	1.09431648	0.130030031	0.2346868	1
Klf5	1.07914515	0.109888923	0.23471195	1
Tgds	1.1238159	0.168405723	0.23472125	1
B4galt4	0.91157428	-0.133567875	0.23488549	1
Txlng	1.18892144	0.24965339	0.23492427	1
Dhcr7	0.92636873	-0.110341537	0.23492905	1
Top1mt	0.88140587	-0.182121593	0.23506678	1
Rars2	0.85871356	-0.219751127	0.23566854	1
Toe1	1.20842658	0.273129821	0.23566934	1
Man1c1	1.10784952	0.147761931	0.23585547	1
Lingo1	0.93209756	-0.101447126	0.23588738	1
Uhrf1	0.7521127	-0.410979237	0.23591155	1
Ndufaf7	1.08091729	0.112256134	0.23603189	1
Cenpj	1.09711357	0.133712882	0.2360413	1
Cdh6	0.91630732	-0.126096555	0.23606934	1
Scrn2	1.19104979	0.252233726	0.23608391	1
Tbxas1	1.2301069	0.298783701	0.23632188	1
Sfxn1	0.94085516	-0.087955458	0.23632966	1
Tnfrsf11a	1.19990458	0.262919688	0.23638159	1
Slco1a2	1.07265935	0.10119198	0.23650646	1
Nmd3	1.09867652	0.135766687	0.2369726	1
Tfg	0.94576991	-0.080438845	0.23707031	1
Nek6	0.92750557	-0.108572147	0.23711171	1

Phtf1	1.06072273	0.085047587	0.2371969	1
Limd1	1.09616866	0.132469793	0.23751838	1
Itgb3	0.8407581	-0.250237328	0.23763321	1
Nod1	1.09741545	0.134109794	0.23809398	1
Atp5h	1.05751246	0.080674662	0.23821386	1
Als2cr12	1.33675804	0.418738359	0.23821793	1
Hexim2	1.15874793	0.212566762	0.23842472	1
Smc1a	1.05686159	0.079786442	0.23851591	1
Pde4c	1.17072703	0.227404734	0.23855082	1
Smox	1.0966534	0.133107628	0.23866686	1
Sbno2	0.90824813	-0.13884161	0.23867727	1
Gda	1.06304368	0.08820088	0.23871124	1
Stxbp6	1.19207877	0.253479567	0.23885822	1
Rom1	1.14384104	0.193886579	0.23891467	1
Pparg	1.2192594	0.286005089	0.23902822	1
Tmem170a	1.17801197	0.236354205	0.23904061	1
Cox4i1	1.05631827	0.079044588	0.23919396	1
Zbtb11	0.93467355	-0.097465526	0.23920178	1
Flrt3	1.09772812	0.134520776	0.23928704	1
Cyp4b1	1.33288161	0.414548638	0.23931083	1
Spc25	1.32900526	0.410346811	0.2393848	1
Pld1	1.08533235	0.118136888	0.23956122	1
Shd	1.10863212	0.148780708	0.23957108	1
Atp5j	1.0575461	0.080720559	0.239606	1
Peli1	0.90056263	-0.151101486	0.23968733	1
Rab22a	0.9282468	-0.107419659	0.2398072	1
Med13	0.92349461	-0.114824553	0.23982054	1
Pcsk6	0.920926	-0.118842866	0.23989588	1
Scn4a	0.87745835	-0.188597448	0.2399245	1
RGD1308428	0.93366566	-0.099022072	0.2400883	1
Tmem208	1.10361586	0.142238099	0.24008894	1
Sumf2	0.84943491	-0.235424701	0.24012107	1
Txlna	0.92807552	-0.107685891	0.24015271	1
Syk	1.16102968	0.21540485	0.2402279	1
Icam2	1.17588147	0.233742649	0.24027295	1
Itpa	1.0721805	0.1005478	0.24028002	1
Lsg1	1.09418263	0.129853554	0.24036104	1
Gldn	0.92267645	-0.116103262	0.24038427	1
Akr1a1	1.05637445	0.079121309	0.24040286	1
Tmem183a	1.06735248	0.094036693	0.24042123	1

Scrib	0.92201545	-0.117137162	0.24051146	1
Snhg11	1.05899838	0.082700384	0.24059817	1
Rabep1	1.05749852	0.080655646	0.24082083	1
Tmem237	1.08814646	0.121872751	0.24100632	1
Eno3	0.85776208	-0.221350559	0.24103593	1
Klc4	1.08925447	0.123341033	0.24122641	1
Abhd10	1.09703799	0.133613483	0.24125921	1
Itih3	1.31785247	0.398188873	0.24139854	1
Pald1	1.1003269	0.137932199	0.24146321	1
Myo9b	0.94165656	-0.086727119	0.24170636	1
Bcas3	0.9344044	-0.097881025	0.24197164	1
Ikbkap	0.93584991	-0.095650928	0.24201071	1
Scyl3	1.10876702	0.148956245	0.24218723	1
Elmod3	0.80741424	-0.308619058	0.24265976	1
Psma5	1.06181316	0.086529929	0.24269516	1
Fmo5	0.80667846	-0.30993436	0.24275889	1
Rpl6	1.05524936	0.077583956	0.24284824	1
Slc7a4	1.14337961	0.193304463	0.24285987	1
Fnbp4	1.07770128	0.107957348	0.24302383	1
Arpp21	1.08853108	0.122382595	0.24305415	1
Rmdn2	1.14088536	0.190153835	0.24306213	1
Dars2	1.11746669	0.160231824	0.24311412	1
Cmah	1.37835671	0.462949293	0.24336019	1
Flt1	0.92981775	-0.104980125	0.24368332	1
Abcg3l4	0.90036678	-0.151415263	0.24371373	1
Taf5	0.85313001	-0.229162477	0.2439268	1
Lgr4	0.93712079	-0.093693082	0.2440084	1
Ctla2a	1.23427434	0.3036631	0.24415736	1
Pafah2	0.79119572	-0.33789347	0.24422344	1
Wif1	0.83779298	-0.255334304	0.24424372	1
Sh3bgrl2	1.13816226	0.186706249	0.2443943	1
Kcnk6	1.22203363	0.289283984	0.24439941	1
Ccl2	0.88708597	-0.172854172	0.24443114	1
Myh2	0.71615158	-0.481663106	0.24445688	1
Pnoc	1.34323835	0.425715323	0.24461265	1
Prelp	1.07120117	0.099229436	0.24475896	1
Yif1a	1.09513009	0.131102255	0.2451917	1
Ndufs4	1.06121866	0.085721942	0.24520825	1
Abhd6	1.07678148	0.106725499	0.24532638	1
Pld3	1.05643832	0.079208542	0.24535361	1

Dhx9	0.94543764	-0.080945787	0.24536693	1
Mgat5b	0.8535429	-0.228464424	0.24540599	1
Brf2	1.12465835	0.16948681	0.24542981	1
Fam222a	0.80133692	-0.31951914	0.24545406	1
Ppif	1.13432703	0.181836638	0.24547122	1
Tdrd1	0.74679244	-0.421220765	0.24556431	1
Alox5	1.22800722	0.296319042	0.24569548	1
Cmtm5	0.92513005	-0.112271905	0.24579698	1
Pfn2	1.05579744	0.078333079	0.24583885	1
Slc35f5	0.92547551	-0.111733282	0.24608538	1
Adhfe1	1.26662343	0.340987674	0.24610532	1
Dnajc22	1.21716077	0.283519741	0.24617505	1
Cript	1.06303684	0.088191595	0.24617877	1
RGD1559747	0.88092796	-0.182904046	0.24628951	1
Setdb1	1.0765904	0.10646947	0.24656797	1
Popdc3	0.90099479	-0.150409338	0.24669502	1
Chrdl1	1.10896347	0.149211844	0.24673743	1
Adra2c	0.88057668	-0.18347945	0.24674509	1
Prdx4	0.92114272	-0.1185034	0.24681368	1
Grap2	1.30387749	0.382808325	0.2468827	1
Amn1	1.10090987	0.138696357	0.24697861	1
Slc1a3	1.08927481	0.123367969	0.24701987	1
Ctdspl	0.91494954	-0.128235914	0.24704474	1
Ddx25	1.07610551	0.105819544	0.24706636	1
Gnb4	0.92095706	-0.1187942	0.24711576	1
Abl2	1.13763897	0.186042795	0.24729465	1
Bub3	1.08567827	0.118596643	0.2474648	1
Cep162	0.88888199	-0.169936195	0.2476235	1
Ltbp1	0.92039723	-0.119671451	0.24774034	1
11-Mar	0.87667063	-0.189893183	0.24800656	1
Ruvbl2	1.08890745	0.122881346	0.24830972	1
Tnfsf10	1.26160785	0.335263539	0.24834367	1
Ngdn	0.90788636	-0.139416371	0.24839087	1
Resp18	1.06026423	0.084423845	0.24846811	1
Cst7	1.49357947	0.578773999	0.24852044	1
Uaca	1.06463414	0.090357733	0.24860186	1
Pidd1	0.82378704	-0.279656662	0.24867648	1
Tnip1	1.07272922	0.101285951	0.24886921	1
B3galt2	1.12575329	0.17089069	0.2490069	1
RT1-DMa	1.1357121	0.183597163	0.24915519	1

Hist3h2ba	1.11004173	0.150613918	0.24916045	1
Rnf150	0.87264732	-0.196529384	0.24922667	1
Mb21d2	1.14406708	0.194171646	0.24924239	1
Abca7	0.94693533	-0.078662189	0.24937469	1
Acadvl	1.06205789	0.086862405	0.2494081	1
Phc2	0.93892455	-0.090918861	0.24943791	1
Ndufs5	1.06107892	0.08553197	0.24968506	1
Sh3bp5	1.08498584	0.117676221	0.24985994	1
Plekhf2	0.90190376	-0.1489546	0.24994582	1
Ccna2	0.77184451	-0.373617844	0.24999214	1
Wdr3	1.09998492	0.13748375	0.25000093	1
Kif26b	0.89629664	-0.157951804	0.25012261	1
Galnt9	0.91493903	-0.12825249	0.25013128	1
Smarcd1	0.92856377	-0.106927108	0.25016742	1
Zfp292	1.07534972	0.104805926	0.25016905	1
Tfcp2	0.90858259	-0.138310438	0.25017854	1
Clta	1.05443275	0.076467084	0.2503734	1
Wdr47	1.05528977	0.077639197	0.25044458	1
Pkm	1.05632644	0.079055745	0.25045299	1
Cox7c	1.05425227	0.076220133	0.25050671	1
Lrrc16b	0.93176792	-0.101957428	0.25059936	1
Msc	0.76985497	-0.377341409	0.2506912	1
Bola1	0.86877052	-0.20295295	0.25096556	1
Rims1	0.92742286	-0.108700803	0.25107533	1
Mfn1	1.07246905	0.100936011	0.25110602	1
Mettl7a	1.09008739	0.124443801	0.25119541	1
Gpr81	1.2704397	0.345327901	0.25123372	1
Zfp133	1.11594293	0.158263251	0.25132405	1
Gpd1l	1.10624755	0.145674266	0.25152555	1
Sos1	0.94635876	-0.079540896	0.25170962	1
Wnk3	1.24419809	0.315216196	0.25176446	1
Cpd	0.94421525	-0.082812304	0.25227894	1
Rmdn1	1.11596291	0.158289085	0.25231453	1
Fcrla	0.77450528	-0.368653024	0.25262367	1
Rhbdd1	0.81797607	-0.289869462	0.25274315	1
Ganc	1.09676283	0.13325158	0.2527622	1
Pragmin	0.85690196	-0.222797946	0.25288825	1
Srm	0.90843921	-0.138538124	0.25291817	1
Stxbp1	0.94719514	-0.078266422	0.25294343	1
RGD1309808	1.34084009	0.423137194	0.25299522	1

Timm23	1.06641338	0.092766784	0.25330806	1
Parvb	0.9422726	-0.085783596	0.25330913	1
Adrbk1	0.94061273	-0.088327237	0.2533435	1
Sap30bp	1.08042478	0.111598637	0.25337978	1
Acvr1b	0.91839606	-0.122811639	0.2533875	1
Colec12	1.08349103	0.115687215	0.25343664	1
Pja1	0.94906497	-0.075421236	0.25372395	1
Myh8	1.20588515	0.270092515	0.25378396	1
Atp1a1	0.94545231	-0.080923401	0.25378605	1
Atp6v1c2	1.30906945	0.388541644	0.25399085	1
Rnf13	1.05382661	0.075637513	0.25407954	1
Eif3j	1.05651288	0.079310359	0.25418711	1
Scamp5	0.92566691	-0.111434938	0.25459075	1
Col6a2	1.05632972	0.079060227	0.25470439	1
Nit2	1.12247979	0.166689475	0.25476298	1
Cd34	1.08715305	0.120555059	0.25477817	1
B9d2	1.1552755	0.208236931	0.25480392	1
Art3	0.94698789	-0.078582113	0.25497417	1
B4galt3	1.10029079	0.137884852	0.2550232	1
Abcb1b	1.29314496	0.370884011	0.25522611	1
Pdk3	1.07357875	0.102428016	0.25528727	1
Zfp61	1.10651771	0.146026544	0.25532183	1
Lca5	0.84933548	-0.235593577	0.25539414	1
Zcchc24	0.94337874	-0.084091012	0.25540112	1
Chfr	1.07233868	0.100760635	0.25567356	1
Lrfn3	0.84197697	-0.248147316	0.25588968	1
Tfip11	1.0754863	0.104989146	0.25605324	1
RGD1306746	1.09797629	0.134846898	0.25615947	1
Cald1	1.05527772	0.077622723	0.25631902	1
Upf3b	1.0820086	0.113711964	0.25647448	1
Kxd1	0.92918355	-0.105964488	0.25651238	1
Sorcs3	1.08287298	0.114864031	0.2566018	1
Sclt1	1.12923485	0.175345556	0.25664574	1
Ss18	0.93185539	-0.101822012	0.25664733	1
Wbscr27	0.80483299	-0.313238649	0.25673538	1
Mark4	0.93278955	-0.100376461	0.25685576	1
RGD1559482	1.18459095	0.244388967	0.25691206	1
LOC100125367	1.15140104	0.203390418	0.25692287	1
Hoxc8	1.2918457	0.369433761	0.25698216	1
Cntf	1.06130117	0.085834109	0.25701719	1

Dhrsx	0.86462598	-0.209851913	0.25724919	1
Adgre5	0.90446278	-0.144866962	0.25724938	1
Tnks2	0.94569719	-0.080549791	0.25729143	1
Uxt	0.88314001	-0.179285926	0.25735832	1
D2hgdh	0.88944126	-0.169028769	0.25747008	1
Gnal	0.94903252	-0.07547057	0.2575348	1
Ttc39c	1.07815178	0.108560297	0.25757201	1
Kdelc2	1.06683242	0.093333576	0.25782614	1
Pigv	0.86771268	-0.20471069	0.25785383	1
Creb5	1.16351181	0.218485851	0.25790972	1
Dnajc11	1.066831	0.093331658	0.25799612	1
Cercam	0.87096616	-0.199311435	0.25803817	1
Rasip1	0.88880731	-0.170057419	0.25822445	1
Tmem134	1.10319278	0.141684919	0.25865383	1
Rps6ka1	0.909595	-0.136703768	0.25868986	1
Fbxo42	0.89519202	-0.159730922	0.25870228	1
Psmb3	1.05817959	0.081584503	0.25889577	1
Nkiras1	1.05916542	0.08292793	0.25921481	1
Ctss	1.07633311	0.106124639	0.25951029	1
Pja2	1.05558211	0.078038802	0.25954669	1
Mmp24	0.93121127	-0.102819578	0.25961441	1
Slc23a2	0.94602462	-0.080050363	0.25996011	1
Gpr155	0.93358331	-0.099149329	0.25997378	1
Hibadh	1.06897386	0.096226579	0.2600826	1
Api5	1.0544387	0.076475231	0.26009385	1
Zfp90	1.22071044	0.287721029	0.2602511	1
Fosb	0.81858935	-0.2887882	0.26029592	1
Sod1	1.05292368	0.074400871	0.26029841	1
Pld4	1.12853421	0.174450149	0.26032135	1
Emid1	0.86102204	-0.215877929	0.26047864	1
Abcb7	1.11212745	0.15332213	0.26048128	1
S100a16	0.91360859	-0.130351885	0.26051884	1
Trex1	0.87902396	-0.186025605	0.26053553	1
Ptpn5	0.8843769	-0.177266745	0.26059446	1
Cpne7	0.80871383	-0.306298814	0.26065866	1
Fcer1g	1.12104156	0.164839765	0.26071567	1
Flt3	1.07856359	0.10911124	0.2607909	1
Fam60a	1.15639554	0.209634953	0.26089488	1
Ptprg	0.91576799	-0.126945955	0.26103447	1
Tob1	1.09125736	0.125991386	0.26112893	1

Cox18	0.88964665	-0.168695653	0.26119436	1
Camta1	0.94935505	-0.074980356	0.26138592	1
Gzmm	1.21565441	0.281733158	0.26139917	1
Ago3	0.88936878	-0.169146341	0.2614863	1
Wbp1	0.92211421	-0.116982642	0.26149601	1
Slc29a2	1.15077604	0.202607089	0.26167829	1
Dbi	0.94967186	-0.074498996	0.26174109	1
Ttc27	1.0834875	0.115682516	0.26185595	1
Mstn	0.8161161	-0.293153684	0.2619374	1
Pcdh8	1.26439072	0.338442348	0.26206918	1
Pgm2	1.1015843	0.139579901	0.26220007	1
Gspt2	1.110239	0.150870272	0.26221572	1
Rps26	1.05554876	0.077993219	0.26233822	1
Nupr1	1.06633784	0.092664594	0.26250147	1
Tmem230	1.05982858	0.083830936	0.26265721	1
Exosc2	0.87543908	-0.191921306	0.26271459	1
Nlgn1	1.10224298	0.14044229	0.26332795	1
Fastkd3	1.10951593	0.149930376	0.26334051	1
Galnt7	0.84952394	-0.235273486	0.26334491	1
Lurap1	1.17372205	0.231090802	0.26340173	1
Nalcn	0.9480448	-0.076972861	0.26350152	1
RGD1305713	1.12034234	0.163939642	0.26358164	1
Ubn1	0.92887248	-0.106447549	0.26369639	1
Snx12	1.10776337	0.147649737	0.26382427	1
Fbxl17	0.91061197	-0.135091668	0.26387422	1
Dnajb14	1.09008122	0.12443563	0.26392341	1
RT1-CE6	1.32519399	0.40620357	0.26416627	1
Abcc1	0.93637134	-0.09484731	0.26423045	1
Eif1a	1.06052698	0.084781323	0.26428829	1
Ap4s1	1.11641451	0.158872782	0.26440463	1
Adat3	0.8087335	-0.30626373	0.26474564	1
Rnf111	0.930456	-0.103990163	0.26490196	1
Eef1b2	1.05491817	0.077131093	0.26505348	1
Nlgn2	0.9359525	-0.095492786	0.26511917	1
Tnik	0.92417004	-0.113769781	0.2651262	1
Ptprj	1.06910717	0.096406481	0.26514492	1
Ercc8	0.91490245	-0.128310173	0.26515428	1
Rttn	0.8542429	-0.227281744	0.26515815	1
Ntrk3	0.91572301	-0.127016821	0.2652788	1
Fam129c	0.73528806	-0.443618532	0.26539426	1

Ociad1	1.05235988	0.07362815	0.26554986	1
Ahcyl1	0.94979099	-0.074318023	0.26564479	1
Wdr18	1.09325782	0.128633662	0.2657373	1
Gpr156	0.84962267	-0.235105841	0.26580083	1
Nin	1.09846221	0.135485238	0.26580558	1
Ptgr1	1.18578538	0.24584292	0.26605859	1
Ndufv2	1.05365617	0.075404167	0.26608963	1
Tomm20	1.05467669	0.076800815	0.26627546	1
Sh3gl1	0.92817768	-0.107527085	0.26651447	1
Htr4	1.18540772	0.245383361	0.26667428	1
Afmid	0.78255365	-0.353738433	0.26710103	1
Slc25a44	0.92629195	-0.110461122	0.26712607	1
Ulk2	0.94824478	-0.076668576	0.2677144	1
Nfe2	1.27679777	0.35253004	0.2678399	1
Mfsd5	1.08207441	0.113799705	0.2678646	1
Pid1	1.15775321	0.211327757	0.26795962	1
Ikzf3	1.39571146	0.481000718	0.26812223	1
Ccny	0.93209308	-0.101454057	0.26826284	1
Igf2	0.93823733	-0.091975186	0.26838242	1
Fto	1.07544902	0.104939132	0.26848796	1
Ocln	1.15314631	0.205575576	0.26852391	1
Sntb2	0.83017274	-0.268516539	0.26854371	1
Aff4	0.95012251	-0.073814552	0.26854883	1
Gnl1	0.93567448	-0.095921391	0.26865573	1
Pdk4	0.87215212	-0.197348306	0.26875217	1
Wwox	1.15683953	0.210188754	0.26879695	1
Pex14	0.93098123	-0.103176021	0.26882782	1
Prg4	1.08839248	0.122198888	0.26884388	1
Pkib	1.09423023	0.129916313	0.26900399	1
Egr3	0.7894103	-0.341152761	0.26919414	1
Pcsk1n	0.94985898	-0.074214756	0.26922307	1
Hsd17b10	1.07332183	0.102082732	0.26929569	1
Psma3l	1.07693907	0.106936632	0.26931716	1
Agpat4	1.06908136	0.096371644	0.26951063	1
Scfd2	1.09698413	0.133542656	0.2698737	1
Tmem159	0.90406555	-0.145500712	0.27002787	1
Slc22a18	1.21323768	0.278862207	0.27003252	1
Gpn3	1.09677562	0.133268401	0.2701604	1
Slc4a11	1.104332	0.14317396	0.27016884	1
Scaf4	1.08762876	0.121186206	0.2702862	1

Slc24a1	1.16835874	0.224483315	0.27040688	1
Chp2	0.91215345	-0.132651543	0.27060821	1
Strn4	0.93374746	-0.098895684	0.2707324	1
Crcp	1.09035129	0.124793017	0.27087473	1
Wdr25	1.16997599	0.226478925	0.27095146	1
Slc6a1	1.08608658	0.119139114	0.27104965	1
Zfp111	0.83604452	-0.258348319	0.27121015	1
Pxdn	0.94781	-0.07733021	0.27124432	1
Cdc42se1	0.93961476	-0.089858717	0.27137654	1
RGD1560455	1.41919675	0.505074613	0.27154088	1
Pla2g3	1.09336064	0.128769345	0.27162236	1
Rap1gap2	0.9427212	-0.085096918	0.27181513	1
Prrc2a	0.94985522	-0.074220468	0.27200813	1
LOC100912312	0.81381958	-0.297219099	0.2720425	1
Zkscan1	1.09699616	0.133558476	0.27206352	1
Plscr2	0.87072858	-0.199705014	0.27208041	1
Ninj1	0.91949087	-0.121092839	0.27211339	1
Hdgfrp2	0.93558321	-0.09606212	0.2721252	1
Ubox5	1.08658536	0.119801508	0.27220945	1
Lamp1	0.95021567	-0.073673093	0.27225963	1
Krr1	0.90309822	-0.147045197	0.27231487	1
Piezo1	0.90920461	-0.137323097	0.27242675	1
Nif3l1	0.88963167	-0.168719955	0.2726365	1
Kcnc1	0.92456809	-0.113148519	0.27266007	1
Dus3l	0.90890508	-0.137798459	0.27284906	1
Frmd6	1.06260581	0.087606505	0.27287693	1
Cnot2	1.08116876	0.112591728	0.27293096	1
Dbnl	1.05518274	0.077492867	0.27304186	1
Rfc1	1.05997297	0.084027477	0.27337375	1
Rad17	0.91711478	-0.124825795	0.27337636	1
Tdo2	1.23307647	0.302262273	0.27337725	1
Eif1ad	1.08617047	0.119250543	0.27338737	1
Dync1h1	0.94819951	-0.07673745	0.27339047	1
Inpp5e	0.91381824	-0.130020858	0.27341809	1
RGD1305110	1.05695438	0.079913106	0.27351266	1
Lsm1	1.10943123	0.149820245	0.27354739	1
Zfp36l1	0.93402282	-0.098470294	0.27368372	1
Mtx1	0.90375955	-0.145989101	0.27379843	1
Lonp2	1.06152611	0.086139863	0.27396277	1
Tamm41	1.11895341	0.162149963	0.2741464	1

Reep4	0.8562447	-0.223904939	0.27416797	1
Tmod4	1.24229784	0.313011103	0.27434076	1
Dnajc24	1.14104869	0.190360358	0.27442888	1
Cdc42ep2	1.0748913	0.104190778	0.27450159	1
Ftsj2	0.83564479	-0.259038272	0.27459691	1
Slc6a8	0.94360516	-0.083744787	0.27464244	1
Fam49a	1.14112486	0.19045666	0.27488488	1
Nt5e	1.10230301	0.140520857	0.27499867	1
Cep295	1.11308915	0.154569152	0.27531797	1
Sec61a1	0.94841222	-0.076413843	0.27538893	1
Csmd1	0.94022571	-0.088920969	0.27543684	1
Ccnc	1.07141694	0.099520015	0.27544905	1
Chrna3	0.94536686	-0.081053798	0.2755516	1
Cd72	0.85903493	-0.219211297	0.27559662	1
Pard6a	1.15393898	0.206566934	0.27575507	1
Nrip3	1.0514711	0.072409202	0.27579365	1
Nav2	0.93353667	-0.099221407	0.27589169	1
Arhgef25	1.06725112	0.093899671	0.27593965	1
Pccb	0.9244941	-0.113263984	0.27602506	1
Commd4	1.08516412	0.117913246	0.27603997	1
Syn2	0.94838658	-0.07645285	0.27607635	1
Mad1l1	0.91690604	-0.125154198	0.27618697	1
Mcts1	0.93239496	-0.100986893	0.27627298	1
Nat8b	0.81361561	-0.297580728	0.27657019	1
Lrrc47	0.92503686	-0.112417236	0.27661824	1
Akr1c14	1.1225653	0.166799372	0.27668918	1
Acaa1	0.91934929	-0.121315004	0.27672142	1
Slc11a1	1.28567395	0.362524822	0.27683694	1
Ccdc77	0.83760191	-0.255663356	0.27686525	1
Mreg	1.28347979	0.360060576	0.27699466	1
Pcp4	1.05180838	0.072871893	0.27724644	1
LOC500300	0.87584749	-0.191248411	0.27732354	1
Gpr75	1.17247972	0.229562975	0.27733293	1
Rhog	1.07803737	0.108407187	0.27734991	1
Rabl6	0.94814085	-0.076826702	0.27747296	1
Ermp1	0.94912983	-0.075322653	0.27762812	1
Slc25a1	0.93637797	-0.094837103	0.27767527	1
C1qtnf2	1.0990089	0.136203066	0.27791566	1
Ubxn8	1.09899596	0.136186077	0.27797329	1
Gfi1b	1.62644116	0.701718633	0.27801686	1

Pex5l	0.93783348	-0.092596319	0.27804935	1
Mblac1	1.14502669	0.195381225	0.27805898	1
Polr2b	1.05699363	0.079966687	0.27806757	1
Rassf4	0.94972553	-0.074417453	0.27812757	1
Slco4a1	1.31753128	0.397837209	0.27818901	1
Kcnk18	0.87188568	-0.197789118	0.27821199	1
Tmub2	0.94095721	-0.087798977	0.27827995	1
Stard4	0.92683524	-0.109615199	0.27843436	1
Zkscan3	0.90551876	-0.143183567	0.27867445	1
Clec4e	0.72269903	-0.468533139	0.27878583	1
Eri2	1.13221608	0.179149315	0.27885244	1
Spag8	1.27357048	0.348878807	0.27902423	1
Dph2	1.16572384	0.221226056	0.27916983	1
Fbxw4	0.91850568	-0.122639456	0.27932304	1
Rab14	0.94021447	-0.088938206	0.27937457	1
Dynlt1	0.91461058	-0.128770485	0.27941953	1
Dock1	0.9352335	-0.096601489	0.27957797	1
Smarcb1	0.93189307	-0.101763674	0.2796191	1
Trmt10c	1.08431609	0.116785383	0.27968791	1
Tbk1	1.06140001	0.085968468	0.27981326	1
Zfp161	0.90985104	-0.136297732	0.27989917	1
Ppie	0.87636185	-0.190401418	0.28000677	1
Pcyt1a	1.07165005	0.099833863	0.28002381	1
Ptprk	0.91973641	-0.120707645	0.28020403	1
Glyctk	1.15248119	0.2047432	0.28043766	1
Mb	0.78373518	-0.351561833	0.28047248	1
Arhgap27	1.1227586	0.167047767	0.28059047	1
LOC100911483	1.06570407	0.091806873	0.28059262	1
Zzz3	0.93101511	-0.103123516	0.28070442	1
Ntan1	1.05630373	0.079024724	0.28123271	1
Atp5i	1.05989071	0.083915513	0.28125465	1
Ndufb2	1.07120539	0.099235124	0.28147537	1
Qpct	0.87438646	-0.193657036	0.28150347	1
Actr1b	0.95194545	-0.071049197	0.28160566	1
Kcnj12	0.91830749	-0.122950778	0.28164407	1
Sema4d	0.93061706	-0.103740461	0.28174023	1
Prex2	1.07166003	0.099847308	0.28174694	1
Gins2	1.30778513	0.387125526	0.2818464	1
Fmnl1	0.92598068	-0.110946004	0.2819269	1
Minos1	0.9402485	-0.088886002	0.28203592	1

Dgkg	0.92173154	-0.117581471	0.28206954	1
Timm22	1.07350514	0.102329101	0.28212236	1
Tmem189	0.95088298	-0.072660283	0.2821991	1
Igf1	0.91141645	-0.133817692	0.28228724	1
Dapk3	0.93075008	-0.103534259	0.28235302	1
Mgmt	1.14137455	0.190772305	0.28243121	1
Fam184b	0.89130868	-0.166002946	0.28248796	1
Mical1	0.94541025	-0.080987584	0.28252139	1
Psma6	1.0518881	0.072981244	0.28253898	1
RGD1306941	1.05625675	0.078960567	0.28263425	1
Fpgs	0.89864315	-0.154179765	0.28289846	1
Sec22a	1.12302113	0.167385074	0.28291375	1
Evc	1.11782383	0.160692832	0.28296206	1
Dvl2	0.87994563	-0.184513708	0.28299156	1
Gimap8	0.88057635	-0.183479991	0.2830417	1
Hsd3b7	1.16057335	0.21483771	0.28316095	1
Mapk12	1.06427887	0.089876221	0.28335006	1
Nomo1	0.95016234	-0.073754073	0.28338995	1
Mitd1	1.11790595	0.160798822	0.28344436	1
Ttc4	1.11334958	0.154906654	0.28364389	1
Apoh	1.27715372	0.352932183	0.28369199	1
Igsf10	0.86121584	-0.215553238	0.28386974	1
Map9	1.07822111	0.108653063	0.28391107	1
Prkar1b	0.95143178	-0.071827887	0.28393248	1
Aff3	0.91747373	-0.124261238	0.28402881	1
Mcam	0.95195352	-0.071036956	0.28406161	1
Prr15	1.2103521	0.275426796	0.28415165	1
Snx19	0.95098591	-0.072504133	0.28439011	1
Cybrd1	1.1341892	0.18166132	0.28451501	1
Misp	0.81293542	-0.298787354	0.28455035	1
Top2b	0.94069925	-0.088194546	0.28456881	1
Gria2	0.93856362	-0.091473558	0.28460019	1
Tspan8	1.05074633	0.071414424	0.28461006	1
Sox2	0.9026004	-0.147840679	0.28469436	1
Rgma	1.1214548	0.16537147	0.28470674	1
Rbm48	0.87510755	-0.192467763	0.28473788	1
Lnpep	0.93333032	-0.099540338	0.28481816	1
Derl3	1.22260937	0.28996353	0.28493295	1
Csf3r	1.21345494	0.279120535	0.2850206	1
Bok	0.92366573	-0.114557253	0.2851979	1

Cyhr1	1.09950977	0.136860429	0.28524618	1
Ada	0.88041049	-0.183751764	0.28526668	1
Stx1a	0.9147162	-0.1286039	0.28530019	1
Coasy	1.07410193	0.103130906	0.28537402	1
Gng10	0.94068852	-0.088210996	0.28545067	1
Cbx5	1.09816511	0.135094975	0.28547368	1
Mapkapk3	1.0967539	0.133239843	0.28558517	1
Pex7	0.87880248	-0.186389154	0.28583217	1
Pgd	1.05424139	0.076205244	0.28603965	1
Igsf8	1.08774103	0.12133512	0.28608042	1
E2f1	1.11204343	0.153213126	0.28633775	1
R3hdm1	0.9340729	-0.098392951	0.28650631	1
Rrn3	0.94427489	-0.082721182	0.2865278	1
Aimp2	0.92430135	-0.113564803	0.28667082	1
Nat1	1.18085281	0.239829153	0.28676072	1
Pomp	1.05361865	0.075352784	0.28691598	1
Rsad2	1.10978847	0.150284717	0.28692272	1
Larp1b	0.93075014	-0.103534169	0.28696284	1
Mvb12b	0.91566147	-0.127113782	0.28700484	1
Fkbp14	1.09854483	0.135593739	0.28706059	1
Ugt1a7c	0.82589646	-0.275967167	0.28707036	1
Klhl7	1.05796851	0.081296687	0.28713158	1
Morc3	1.06648082	0.092858022	0.2872089	1
Hadhb	1.05012787	0.070565003	0.28725146	1
Ccdc25	1.07897833	0.109665886	0.28727515	1
Myo1b	0.94042821	-0.088610278	0.28732598	1
Ankrd42	0.91875314	-0.122250823	0.28749627	1
Tmem5	1.08798491	0.121658541	0.28772185	1
Pgm5	1.14936611	0.200838413	0.28776288	1
Ttpal	0.94066499	-0.088247079	0.28807772	1
Borcs7	1.06963007	0.097111923	0.28813056	1
Scrn1	0.94994339	-0.074086546	0.28832892	1
B2m	1.05203551	0.073183399	0.28844008	1
Lrrc63	0.81940142	-0.287357709	0.28844568	1
Nsun5	1.10548148	0.144674855	0.28847156	1
Aga	0.93113437	-0.102938725	0.2887187	1
Pitpna	0.95268796	-0.069924337	0.28887219	1
Tada2a	1.08935516	0.123474384	0.28896277	1
Evi5	0.95191232	-0.071099401	0.28897864	1
Zfp219	0.93135216	-0.102601314	0.28899348	1

Oaz1	0.95250783	-0.07019714	0.28908737	1
Tufm	1.05717531	0.08021463	0.28917717	1
Dcaf13	0.92562029	-0.11150761	0.28919042	1
Stk16	1.07754871	0.107753087	0.28925571	1
Sfxn5	0.88715172	-0.172747245	0.28930814	1
Micb	0.76700945	-0.382683738	0.28949933	1
Ccdc126	1.06474203	0.090503925	0.28963285	1
Nus1	0.95061542	-0.073066292	0.28967597	1
Vamp1	1.05267843	0.074064797	0.28973701	1
Ldhb	1.05074921	0.071418376	0.28974276	1
Edn3	1.34541374	0.428049901	0.28981466	1
Nsmf	0.94611518	-0.079912274	0.29010145	1
Ethe1	1.10546762	0.144656767	0.2901424	1
Lsm14a	0.94072108	-0.088161065	0.29029018	1
Serpine2	0.94979308	-0.074314854	0.29032362	1
Fads3	0.92436359	-0.113467663	0.29038884	1
Stk32c	0.92267619	-0.116103661	0.2904608	1
Dlgap5	0.86861067	-0.203218423	0.29056132	1
Sbds	1.05575323	0.078272665	0.29058195	1
Camk1g	0.86378566	-0.211254732	0.2908739	1
Hdhd2	0.91891492	-0.121996801	0.29091084	1
Chpf	0.94720522	-0.078251065	0.2910879	1
Scpep1	1.06490362	0.09072286	0.29112489	1
Mbip	1.11786319	0.160743639	0.29118323	1
Idi1	0.94939242	-0.074923566	0.29138443	1
Zic4	0.75499708	-0.405457027	0.29152378	1
Kcnh5	0.87889577	-0.186236017	0.29155017	1
Efcab7	1.11744872	0.160208622	0.29170224	1
Cisd3	1.07964143	0.110552247	0.29174008	1
Grik1	0.95105321	-0.072402032	0.29181248	1
Ncor2	0.95095811	-0.072546299	0.29193864	1
Ccnk	1.08571724	0.11864842	0.29205906	1
Lox	1.17775179	0.236035529	0.29210505	1
Fbxo33	0.91910128	-0.121704244	0.2925748	1
Pgk1	1.04977448	0.070079431	0.29268639	1
Vsnl1	1.04967803	0.06994688	0.29270746	1
Inpp5a	0.92086092	-0.118944808	0.29272051	1
Yod1	1.17023412	0.226797182	0.2927469	1
Dimt1	0.86026742	-0.217142902	0.29275175	1
Pank2	0.88886189	-0.169968821	0.29276014	1

Sertm1	1.07369972	0.102590579	0.29283964	1
Slc3a2	1.05169355	0.07271439	0.29288732	1
Ttll12	1.10793388	0.147871792	0.29312591	1
Nubp1	0.90667048	-0.141349784	0.29317497	1
Ndufb6	1.05867467	0.082259323	0.29320797	1
Rbm12	0.91675299	-0.125395024	0.29335286	1
Rasal3	0.89100593	-0.166493059	0.29352516	1
Cilp2	1.39696888	0.482299881	0.29356855	1
Timp2	0.95227847	-0.070544587	0.29371343	1
Gltscr1	0.90876318	-0.138023707	0.29382297	1
Clrn1	1.09080133	0.125388369	0.29393568	1
Rybp	0.93642814	-0.094759806	0.29394353	1
Gja5	0.79627663	-0.328658374	0.29397321	1
Arhgap20	1.11822394	0.161209137	0.29421659	1
Zfp641	0.87837945	-0.187083793	0.29437306	1
Mrpl17	1.05256119	0.073904111	0.29441659	1
Rufy2	0.93986376	-0.089476457	0.29446808	1
Rock2	0.9516602	-0.071481564	0.29458936	1
Ppp1r1a	0.94345134	-0.083979991	0.29459192	1
Nabp2	0.93322992	-0.099695533	0.29475859	1
Gclc	0.93847858	-0.091604282	0.2947854	1
Fbxl7	1.17390154	0.231311411	0.29479641	1
Klhdc10	0.9210596	-0.118633582	0.29480525	1
Rtfdc1	1.05594389	0.078533178	0.29498188	1
Tgfbr3	0.9430212	-0.084637887	0.29502206	1
Rprd1b	1.08386371	0.116183353	0.29505866	1
Map2	1.04973673	0.070027546	0.29510306	1
Ndufa4	1.04923487	0.069337658	0.29511932	1
Col5a2	0.95268817	-0.069924022	0.29513309	1
Etnk1	0.95317478	-0.069187318	0.29514145	1
Dgkb	1.07409222	0.10311786	0.29515921	1
RGD1310262	0.78145376	-0.355767585	0.29518861	1
Sema3c	1.05041056	0.070953328	0.29526511	1
Cpne4	1.05174783	0.072788837	0.29526877	1
Arhgdib	1.08404333	0.116422428	0.29527508	1
Nampt	0.93902434	-0.090765546	0.29528827	1
Ap3d1	0.95314439	-0.069233313	0.29558062	1
Pced1a	1.10752908	0.147344583	0.29561989	1
Camkv	0.8661687	-0.207280053	0.29571703	1
Kcnc3	0.94935913	-0.074974146	0.29574508	1

Snip1	1.09349949	0.128952553	0.29603624	1
Atf6b	0.93849297	-0.091582159	0.29611235	1
Dhtkd1	1.0941099	0.129757664	0.2961539	1
Pgap2	1.10688266	0.146502287	0.29623247	1
Hsp90aa1	1.05317667	0.074747473	0.29630598	1
Pde5a	1.11302234	0.154482551	0.29632179	1
Sowahc	0.93548268	-0.09621715	0.29680564	1
Celsr3	0.93870769	-0.091252119	0.29698068	1
Hrasls	1.21501727	0.280976822	0.29702169	1
Sema4c	0.93478396	-0.09729511	0.29713923	1
Rps17	1.05340168	0.075055659	0.29718185	1
Pigw	1.12701717	0.172509497	0.29727787	1
Nab1	0.94643494	-0.079424758	0.29762936	1
Lifr	0.95257114	-0.070101251	0.29776082	1
Zfp316	0.88533101	-0.17571114	0.29790155	1
Rgs7bp	0.94155447	-0.086883536	0.29794241	1
Impdh2	1.06076851	0.085109859	0.29796945	1
Bche	1.1696077	0.226024711	0.29797635	1
Qrsl1	1.12659837	0.171973293	0.29807672	1
Wars	1.04959261	0.069829471	0.29814488	1
Rbp1	1.11004373	0.150616508	0.29820481	1
Rnase2	1.46884456	0.554681733	0.29821128	1
Prph	0.95077133	-0.072829695	0.29832914	1
RGD1309534	1.08750044	0.121015981	0.29837013	1
Prelid1	0.94802611	-0.077001297	0.29838745	1
Rnf144a	0.90204099	-0.148735103	0.29841113	1
Rell2	0.94849001	-0.076295512	0.29846499	1
Als2	0.93226707	-0.101184787	0.29861452	1
Maz	0.92394747	-0.114117257	0.29867107	1
Nyap1	0.93554782	-0.096116698	0.29871057	1
Brpf1	1.09629106	0.132630881	0.29893195	1
Vps41	1.0496797	0.069949176	0.29910217	1
Ankrd24	1.1207475	0.164461286	0.29911763	1
Slc39a13	1.07108272	0.099069902	0.29932391	1
Mrpl3	0.9358012	-0.095726012	0.29933375	1
Plpp1	1.05381722	0.075624653	0.29933928	1
Hadha	1.04876885	0.068696745	0.29937642	1
Fads1	0.95367035	-0.068437429	0.29941119	1
Epyc	0.75201026	-0.411175754	0.29942758	1
Kcna3	0.81076285	-0.302648111	0.29944577	1

Slc2a3	1.0745257	0.10369999	0.29948181	1
Ttc23	1.11293715	0.154372125	0.29949927	1
Nup43	1.12376524	0.168340676	0.29958799	1
Lats2	0.94708448	-0.078434971	0.2996252	1
Heyl	0.91204031	-0.132830513	0.29974449	1
Nat14	0.86560427	-0.208220477	0.30037353	1
Nlrp3	1.20814958	0.27279909	0.30066156	1
Htatip2	1.06602173	0.092236849	0.30066204	1
Sf3a2	0.89805239	-0.155128491	0.30087318	1
Ccdc134	1.19583232	0.258015113	0.30088458	1
Btaf1	0.93727066	-0.093462368	0.30090936	1
Emc10	0.94920657	-0.075206014	0.3009716	1
rnf141	0.92180033	-0.117473806	0.30098008	1
Emc1	0.94718359	-0.078284001	0.30106879	1
Aadat	0.80458122	-0.31369004	0.30116994	1
Med27	1.0791919	0.109951423	0.30121886	1
Foxc2	1.12117407	0.165010283	0.30122174	1
Fam65b	1.05755877	0.080737845	0.30132297	1
Cutc	0.88867201	-0.170277048	0.30136431	1
Zc3h6	1.1334633	0.18073768	0.30145363	1
Egflam	0.9308752	-0.10334033	0.30152931	1
Fam127b	0.95001397	-0.073979362	0.30153095	1
Pls1	1.06068579	0.084997346	0.30153182	1
Eif5a2	1.04950255	0.069705673	0.3016856	1
Avpi1	0.9208802	-0.118914603	0.30186905	1
Zfp157	1.10059679	0.138286021	0.30192037	1
Dis3l2	0.90932345	-0.137134536	0.30192396	1
Hspb1	1.04848521	0.068306509	0.30196824	1
Apold1	0.88087704	-0.182987441	0.30208544	1
Srrm2	0.95256321	-0.07011327	0.3021148	1
Egln2	0.94776729	-0.077395227	0.30239064	1
Cish	0.90933697	-0.137113083	0.30240759	1
Rpl35a	0.94396615	-0.083192962	0.30249606	1
Ptgdrl	0.9067503	-0.141222783	0.30287056	1
Thrb	0.9085288	-0.13839585	0.30291178	1
Gusb	1.11444566	0.156326268	0.30312327	1
Cacybp	1.04832387	0.068084493	0.30319589	1
Sfpq	0.94657227	-0.079215433	0.30328828	1
Exoc3l1	1.18880234	0.249508862	0.30331092	1
Abhd5	0.91380314	-0.130044698	0.30332609	1

Smad9	1.08376168	0.116047543	0.30337345	1
Cc2d1b	0.94396151	-0.083200054	0.30347353	1
Tpst2	0.92479207	-0.112799074	0.30360832	1
Sorl1	0.95247021	-0.07025413	0.30398727	1
Irf5	1.1634192	0.218371017	0.3041233	1
Gmeb1	1.12851577	0.174426579	0.30418603	1
Timeless	1.21652706	0.282768412	0.30421716	1
Myrf	1.12343681	0.167918975	0.30422601	1
Dgcr8	0.92880614	-0.106550587	0.30423813	1
Enpp5	1.05096332	0.07171232	0.30426511	1
Ift172	1.06344971	0.088751816	0.30430767	1
Il1rap	0.89049816	-0.167315469	0.30439887	1
Anapc5	1.04825678	0.067992163	0.30443845	1
Cachd1	0.91223813	-0.132517627	0.30450792	1
Orc5	1.07148879	0.099616758	0.30451072	1
Chmp5	1.05006754	0.070482131	0.3045705	1
Tmem106c	1.09605591	0.132321396	0.30462532	1
Svil	1.05974779	0.083720958	0.30472857	1
B3galnt1	0.93291016	-0.100189938	0.30475554	1
Ttbk2	0.95016371	-0.073751991	0.30490341	1
Hlx	1.24616911	0.317499866	0.30499885	1
Pcdhb5	1.21162153	0.276939118	0.3050449	1
Ctsa	1.04813955	0.067830813	0.30513904	1
Pcdha9	1.16645611	0.222132029	0.30514802	1
E2f7	1.36334343	0.44714903	0.30549006	1
Cct2	1.04807692	0.067744607	0.30555736	1
Slc9a1	0.94479303	-0.081929776	0.30563004	1
Acp6	1.12524389	0.170237733	0.30581464	1
Ccna1	0.81122717	-0.301822115	0.30583372	1
Tmem165	1.07341005	0.102201304	0.30588385	1
Gpr108	1.06779387	0.094633178	0.30601069	1
B3gat3	1.05041207	0.070955396	0.30611095	1
Rabggta	0.91780319	-0.123743268	0.30641551	1
Sh2d3c	0.92017233	-0.120024024	0.30641985	1
Ctns	1.07894534	0.109621785	0.30654484	1
Map2k3	0.91040167	-0.135424894	0.30670948	1
Svop	0.92794703	-0.107885644	0.30679919	1
Brd1	0.93220986	-0.101273315	0.30690549	1
Zbtb8b	1.24500621	0.316152937	0.30691972	1
Fbxw17	1.11802403	0.160951193	0.30694904	1

Obfc1	0.91932102	-0.121359359	0.30702172	1
Cdkn2aip	1.0952537	0.131265091	0.30706758	1
Tinagl1	1.10862643	0.148773312	0.30712908	1
Scn7a	1.05074784	0.071416492	0.30713409	1
Heatr1	0.91733877	-0.124473477	0.30716341	1
Got1	0.95426682	-0.067535385	0.30718102	1
Whsc1l1	0.93123373	-0.102784786	0.30734767	1
Cryab	0.95329959	-0.068998424	0.30738407	1
Homer1	1.09082895	0.125424899	0.30739193	1
Ebf1	0.93787551	-0.092531658	0.30745994	1
Stx1b	1.05337886	0.075024407	0.30748922	1
Lhfp	1.0656556	0.091741267	0.30752129	1
Polg	0.93760876	-0.092942047	0.30756484	1
LOC500227	1.25647016	0.329376413	0.30764568	1
Il6r	0.9137688	-0.130098914	0.30772845	1
Synpr	0.9543141	-0.067463905	0.3077514	1
Mrap2	0.94105712	-0.087645798	0.30842137	1
Eif4e3	1.0901792	0.124565298	0.30842558	1
Nup214	0.93526783	-0.096548533	0.30857904	1
Selplg	0.82827073	-0.271825696	0.30885273	1
Lypd3	0.75389494	-0.407564597	0.30887655	1
RGD1305350	0.93850694	-0.091560676	0.30888976	1
Pcif1	1.11980649	0.163249446	0.30891482	1
Ns5atp9	1.40441376	0.489968032	0.30895343	1
Podxl2	0.93238815	-0.100997421	0.30897277	1
Ndufb9	1.05095085	0.071695201	0.30898204	1
Ppfibp1	1.06224684	0.087119051	0.30908672	1
Fhod3	1.05692148	0.079868207	0.30911349	1
Angpt1	1.14660138	0.197363926	0.30916879	1
Siva1	1.15203016	0.204178481	0.30918682	1
Plek	1.15759332	0.211128506	0.30930605	1
Rad51c	0.8455678	-0.242007657	0.30933151	1
LOC100125362	1.04765175	0.067159232	0.30948637	1
Ltbp2	1.0924859	0.127614656	0.30953438	1
Rasgrf2	1.19527019	0.257336771	0.30967088	1
Plcd4	0.95048444	-0.073265092	0.3098364	1
Nr2f2	1.07064474	0.098479847	0.30997222	1
Usp16	1.07595664	0.105619943	0.30998872	1
Kbtbd4	0.85338689	-0.228728154	0.31007089	1
Prkg2	1.11720183	0.159889842	0.31009029	1

Snap91	0.95458153	-0.067059672	0.31016178	1
Stxbp4	1.16282688	0.217636327	0.31020962	1
Pmfbp1	1.17112247	0.227891955	0.31025159	1
Pkd1	0.95355789	-0.068607566	0.31028273	1
Samhd1	1.13359248	0.180902097	0.31028425	1
H19	1.10211559	0.140275536	0.3103388	1
Cd276	1.08953983	0.123718937	0.31042957	1
Mtus2	1.17223882	0.229266524	0.31054476	1
LOC100125364	0.94183667	-0.086451193	0.31064106	1
Rwdd1	1.08348903	0.115684552	0.31102375	1
Bdkrb2	1.09111677	0.125805509	0.31114557	1
Bend5	1.17978206	0.238520376	0.31118263	1
Eci3	0.9137181	-0.130178959	0.31136594	1
Cntnap4	0.91863098	-0.122442657	0.31154393	1
Rbbp5	1.06274773	0.087799171	0.31178252	1
Wbp11	0.9356402	-0.09597425	0.31179622	1
Hexdc	0.9056244	-0.143015261	0.31180363	1
Ccp110	1.05538178	0.07776498	0.31196744	1
Zfp287	1.09250701	0.127642539	0.31214585	1
Osbpl2	0.94163437	-0.086761116	0.3122334	1
Fkbpl	0.91195281	-0.132968928	0.31236766	1
Tns1	0.95446294	-0.06723892	0.3124541	1
Desi1	0.93314584	-0.099825512	0.31248042	1
Cxcl12	1.0677416	0.094562548	0.31252292	1
Enpep	1.08119231	0.11262315	0.31252856	1
Gna15	1.2535594	0.326030355	0.31254353	1
Tbx15	1.21201893	0.277412231	0.31267676	1
Vcan	0.92763787	-0.108366372	0.31272992	1
Nkg7	1.38153192	0.466268894	0.31274243	1
Cxcr2	1.40050662	0.485948808	0.31289472	1
Naa25	0.9313771	-0.102562681	0.31291584	1
Lbh	0.94717401	-0.078298608	0.31296204	1
Cxcl14	1.30851549	0.387931003	0.31299126	1
Sema4f	0.94018655	-0.088981054	0.31313547	1
Fbxl8	0.82168785	-0.283337662	0.3131489	1
Cox4i2	1.25539984	0.328146927	0.31317377	1
Rpl27	1.05297845	0.074475913	0.31322068	1
Rapgef2	0.93794934	-0.092418094	0.31336727	1
Capn15	0.92060608	-0.119344123	0.31343996	1
Rab40b	0.85320626	-0.229033549	0.31346814	1

Brk1	1.049907	0.070261535	0.31361255	1
Dgcr6	1.06409755	0.089630409	0.31369724	1
Eno4	0.81136975	-0.301568583	0.31373785	1
Cmip	0.94999051	-0.074014999	0.3137735	1
Aspa	1.06912131	0.096425556	0.31379484	1
Cd19	1.2491952	0.320998929	0.31388852	1
Mrpl53	1.08271149	0.114648862	0.3139301	1
Kcnk16	1.30425493	0.383225886	0.31401841	1
Sirpa	1.07144838	0.099562341	0.31412208	1
Zbtb10	0.88369513	-0.178379365	0.31417793	1
Pcsk4	0.84719942	-0.239226491	0.31432683	1
Hnmt	1.28967718	0.367009994	0.31442534	1
Slco3a1	0.92440063	-0.113409852	0.31455088	1
Hspe1	1.06760865	0.094382893	0.31479451	1
Astn1	0.94790123	-0.077191355	0.31492378	1
Hint2	1.0870137	0.120370122	0.31505038	1
Pkp2	0.81494	-0.295234246	0.31506821	1
Bank1	1.33115575	0.412679384	0.31507207	1
Nme1	1.0527596	0.074176025	0.3151796	1
Tbc1d25	0.91251167	-0.13208509	0.3154117	1
Ndufa1	1.06609163	0.092331445	0.31555005	1
Slc25a38	1.07857389	0.109125021	0.31557169	1
Rpa3	0.8917964	-0.165213724	0.31571174	1
Atraid	0.91358607	-0.130387441	0.31572715	1
Ube2h	0.95372343	-0.068357138	0.3157495	1
Ppargc1b	0.83803544	-0.254916843	0.31575214	1
Tm2d3	1.14768263	0.198723751	0.31575725	1
Smarcd2	0.93734345	-0.093350336	0.31604361	1
Rnf182	0.85006303	-0.234358277	0.31655801	1
Mib2	0.93364192	-0.09905875	0.31658544	1
Sema6b	0.8916883	-0.165388604	0.31663044	1
P3h2	1.14953013	0.201044275	0.31691709	1
Shisa5	0.94377638	-0.083483032	0.31696462	1
Asb8	1.05360864	0.075339079	0.31711397	1
Urb1	0.93285009	-0.10028284	0.31714794	1
Ppp3cc	1.07255804	0.101055714	0.3171664	1
Smpd5	0.90331049	-0.146706138	0.31724854	1
Hnrnpa1	1.04811674	0.067799417	0.31729848	1
Lzts3	0.91103556	-0.134420725	0.31730119	1
Myadm	0.94929421	-0.075072814	0.31741847	1

Ampd2	0.93758502	-0.092978579	0.3176588	1
Imp4	0.92269389	-0.116075988	0.31778431	1
Gmeb2	1.17015087	0.226694558	0.31790844	1
Zc3h8	0.8956386	-0.159011385	0.31792126	1
Snn	0.95276749	-0.069803911	0.31799892	1
Elp2	1.04878191	0.068714711	0.31814269	1
RGD1305464	0.85485339	-0.226251074	0.31837972	1
Cttnbp2nl	0.9458547	-0.080309524	0.31839498	1
Zfp395	1.11566468	0.157903483	0.31841872	1
Stag1	0.93243403	-0.100926433	0.31842453	1
Mif	0.94935213	-0.074984784	0.31853765	1
Slc8a3	0.91018503	-0.135768232	0.31872257	1
Slc35f1	0.94760082	-0.077648648	0.31874747	1
Nkain4	0.88249679	-0.180337058	0.31887286	1
Ddit4l2	1.30899719	0.388462	0.31887481	1
Sdhc	1.05124841	0.072103613	0.3188955	1
Pqbp1	0.92566008	-0.111445597	0.31935814	1
Srebf1	0.9434176	-0.084031577	0.31952546	1
Fam192a	1.07176877	0.099993681	0.31963999	1
Plod3	0.93499175	-0.096974459	0.32000305	1
Trim8	0.9422638	-0.085797079	0.32000808	1
Fign	0.8814976	-0.181971451	0.3200448	1
Thrap3	1.05014799	0.070592652	0.32009186	1
Itfg2	1.13285148	0.179958729	0.32012458	1
RGD1561931	1.16142929	0.21590132	0.32023589	1
Zc3h10	0.90208652	-0.148662291	0.32043786	1
Mprip	0.95535391	-0.065892819	0.32046953	1
Ubr7	1.07022915	0.097919735	0.32064119	1
Ccdc62	0.8954336	-0.159341634	0.3206924	1
Adam15	0.94500008	-0.081613646	0.32074619	1
Coprs	0.91424544	-0.129346566	0.32074658	1
Map2k7	1.09375954	0.129295597	0.32076973	1
Eefsec	0.9096825	-0.136565	0.32078166	1
Idh3B	1.04688576	0.066104019	0.32085239	1
Espl1	1.25899163	0.332268692	0.32085913	1
Nrsn1	0.94584883	-0.080318472	0.32097883	1
Lin7c	0.8979769	-0.155249758	0.32098119	1
Man2b1	0.94279334	-0.084986521	0.32105493	1
Tspan11	0.92237069	-0.116581431	0.32106721	1
Xpo1	0.95110465	-0.072324004	0.32131939	1

Il16		1.0691677	0.096488156	0.32140042	1
Vcam1		1.0982888	0.135257464	0.32169924	1
Kcnd2		0.78247709	-0.353879591	0.32177298	1
Ppp2r1b		1.10238085	0.140622732	0.32220496	1
Zfp398		1.12087898	0.164630517	0.32233487	1
Atmin		0.9452086	-0.081295335	0.32234006	1
Tug1		0.94888664	-0.075692358	0.32235115	1
Ttc30a1		0.83237881	-0.264687853	0.3226046	1
Ndor1		0.90731215	-0.140329114	0.32260903	1
Hmgb3		0.93526802	-0.096548234	0.32285035	1
Pign		1.09117351	0.12588052	0.32297862	1
Ect2		0.80282992	-0.316833703	0.32300174	1
Hal		0.80561112	-0.311844492	0.32305511	1
Pou4f2		0.91598279	-0.126607599	0.32321364	1
Banp		0.92995886	-0.104761199	0.32330655	1
Pcbp4		0.9551376	-0.066219503	0.32333383	1
Glud1		0.9558242	-0.065182796	0.32334696	1
Pdzrn3		0.92769318	-0.108280358	0.32338713	1
Elavl1		0.9246673	-0.112993729	0.32348284	1
Runx1		0.90091676	-0.150534278	0.32358712	1
Tmem252		0.84675733	-0.239979532	0.32359641	1
Cadm2		0.89160755	-0.165519262	0.3237621	1
Pou4f3		1.07115113	0.099162048	0.32391232	1
Myh9l1		0.95579398	-0.065228412	0.32393093	1
Lypla2		0.94395263	-0.083213633	0.32397697	1
Ppp2r3b		1.13946018	0.188350512	0.3240466	1
Map4k1		1.19409459	0.255917123	0.32423104	1
Rbm14		0.91710151	-0.124846659	0.32431282	1
Maea		1.04752959	0.066990999	0.32457257	1
Plekha1		0.94989458	-0.074160689	0.32468791	1
Rad23a		1.07779001	0.108076116	0.32478791	1
Fbxo11		0.94954595	-0.074690274	0.32486775	1
Tmod3		0.9360759	-0.095302581	0.32501301	1
	7-Sep	1.04646486	0.06552387	0.32504351	1
Add1		0.95570223	-0.065366913	0.3251107	1
Slc35g2		1.08624161	0.119345036	0.3251415	1
Sympk		0.94677274	-0.078909933	0.32523259	1
Fundc1		1.05154145	0.072505715	0.32528339	1
Atp6v1h		1.04701779	0.066285962	0.32533979	1
Syt9		0.93742335	-0.093227366	0.32550366	1

Pacsin1	0.94661316	-0.079153116	0.32575199	1
Dclk2	1.05374681	0.075528259	0.32584335	1
Nfkbie	0.89622719	-0.158063598	0.3258982	1
Edn1	1.25594485	0.328773112	0.32598842	1
Sephs2	0.93315526	-0.099810957	0.32613572	1
Nedd8	1.04923	0.069330965	0.32615928	1
Ly96	1.28178078	0.358149539	0.32616473	1
Mdga1	0.91062924	-0.135064303	0.32618664	1
Chst5	0.87409526	-0.194137583	0.32622495	1
Med29	1.08698707	0.120334778	0.32626	1
Rpl9	1.04635559	0.065373214	0.32631495	1
Gtpbp10	1.14741296	0.198384724	0.32640543	1
Rab2a	1.05323358	0.074825419	0.32646098	1
Ctsc	0.93132288	-0.102646667	0.3264904	1
Smad3	1.10022477	0.137798292	0.32658947	1
Polr3d	1.06407193	0.089595676	0.32661577	1
Galnt6	0.86988028	-0.201111239	0.32700641	1
Tal1	1.19755616	0.260093319	0.32704627	1
Mn1	0.79557834	-0.329924101	0.32715952	1
Zfp518a	1.07751845	0.107712573	0.32722472	1
Rnf144b	1.15872727	0.212541037	0.32727325	1
Ppargc1a	1.09824738	0.135203054	0.32742098	1
Bag3	0.9537257	-0.068353707	0.32743628	1
St3gal2	0.94697392	-0.078603404	0.32747233	1
Chst11	0.90318457	-0.146907254	0.32748573	1
Npap60	0.93852445	-0.091533758	0.3275167	1
Nek11	0.84253048	-0.24719921	0.3277113	1
Pes1	0.94414486	-0.082919861	0.32776271	1
Phactr2	1.06698047	0.093533764	0.32778112	1
Ddx21	1.05823129	0.081654983	0.32778739	1
Mrps30	1.06296624	0.088095784	0.32784239	1
Mctp2	1.06032695	0.08450919	0.32794835	1
Gtpbp4	1.0859723	0.118987311	0.32821688	1
Kdm2a	0.95250358	-0.070203587	0.32822638	1
Tbp	1.10973261	0.150212096	0.32838773	1
Papd7	0.92815808	-0.107557559	0.32838987	1
Mrap	1.09513158	0.131104222	0.32854134	1
Ddx6	0.94421422	-0.082813891	0.32860985	1
Hipk1	0.95482494	-0.066691844	0.32874056	1
Meox1	1.26344266	0.337360188	0.32877952	1

Prkci	0.9519195	-0.071088525	0.32879787	1
Csrnp1	0.83374589	-0.262320355	0.32888955	1
Mbd2	0.95042926	-0.073348845	0.32908204	1
Cdc6	0.75816648	-0.399413422	0.32909806	1
Mdh1	1.04712159	0.06642897	0.32920313	1
Psmd10	0.89305303	-0.163182247	0.32923475	1
Tead1	0.87029696	-0.200420337	0.32939286	1
RGD1309821	0.9314716	-0.102416308	0.32939446	1
Rnf167	1.05754692	0.080721679	0.32967096	1
Nsmce2	0.91011573	-0.135878078	0.32975963	1
Kcnk12	1.13615042	0.184153851	0.32993919	1
Spred3	0.84071563	-0.250310203	0.32994528	1
Hemk1	1.15061596	0.20240639	0.33005136	1
Taf11	1.08409214	0.116487385	0.33006237	1
C5ar2	1.25344949	0.325903862	0.33016869	1
Plcxd3	0.93661631	-0.09446994	0.33023734	1
Msl3	1.06485937	0.09066291	0.3302438	1
Zfp18	1.12139782	0.165298172	0.33025165	1
Aldh3b1	0.86988411	-0.201104885	0.33067193	1
Pygl	1.07055543	0.098359502	0.33073059	1
Tom1l2	0.95286979	-0.069649013	0.33090558	1
Hspb11	1.13933004	0.188185727	0.33100052	1
Cox8a	1.04679514	0.065979129	0.33106983	1
Cntn3	0.91048049	-0.135299985	0.33117787	1
Tm9sf2	0.95611398	-0.064745473	0.33127813	1
Evi2b	1.20409022	0.267943494	0.33136223	1
Ppil1	1.09140486	0.126186377	0.33139975	1
Dnajc27	0.95380318	-0.068236509	0.33145452	1
Slc35e3	0.94783354	-0.077294386	0.33152751	1
Atp5sl	0.93693337	-0.093981646	0.33155682	1
Scx	1.12523453	0.170225733	0.3315963	1
Nupl2	1.12272196	0.167000692	0.33176821	1
Sipa1l3	0.89420545	-0.161321757	0.33177944	1
Ldhd	1.11449772	0.156393671	0.33184727	1
Mrpl27	0.93526709	-0.096549668	0.33201659	1
Manf	0.91630633	-0.126098116	0.3320603	1
Cyb5d2	1.13641307	0.184487326	0.33212511	1
Zfp167	0.84764641	-0.238465521	0.33214056	1
Anxa2	1.0476708	0.067185467	0.33231893	1
LOC683897	1.08354579	0.115760116	0.33255135	1

Hmox2	1.04897995	0.068987109	0.33259894	1
Ppp4r4	1.0649854	0.090833649	0.33277368	1
Edc4	1.06088824	0.085272678	0.33279916	1
Rhd	1.21943187	0.286209151	0.33286718	1
Rfxap	0.91819356	-0.123129776	0.33290118	1
Mterf2	1.12705153	0.172553477	0.33302162	1
Ncmap	0.93504108	-0.09689835	0.33306304	1
Tap1	1.10375874	0.142424861	0.33340997	1
Scaf11	1.05223214	0.073453021	0.3334321	1
Tp53i11	0.92530789	-0.111994605	0.33370791	1
Mrpl55	0.92188071	-0.117348019	0.33373046	1
Adam23	1.04526893	0.063874169	0.33377986	1
Tiam1	0.92514372	-0.112250598	0.33381959	1
Slc44a2	0.9558297	-0.0651745	0.33395028	1
Ube2m	0.95272459	-0.069868874	0.33414828	1
Cox5b	1.04792667	0.067537763	0.33427967	1
RGD1565033	1.11392718	0.155654924	0.33432621	1
Sh3bgrl3	0.95628148	-0.064492766	0.33437715	1
Rnf166	1.09596796	0.132205617	0.33438438	1
Setmar	1.12238486	0.166567456	0.33453133	1
Tiprl	1.06146923	0.086062549	0.33458826	1
Clpx	1.07139989	0.099497057	0.33465581	1
Npepl1	0.92197651	-0.117198105	0.33474176	1
Tmem248	1.0652905	0.091246902	0.33477944	1
Stat1	1.04851635	0.068349353	0.33479749	1
Kansl3	0.95050619	-0.073232068	0.33480945	1
Ap5b1	0.90714775	-0.140590551	0.33486668	1
Slc1a4	0.93286048	-0.100266771	0.33498434	1
Fgf7	1.16397882	0.219064804	0.33501069	1
Lars	1.04614442	0.065082031	0.33521961	1
Ldlrap1	1.13480613	0.182445854	0.33525364	1
Ppm1h	0.94532464	-0.081118229	0.33525776	1
Rnf25	1.07748888	0.107672975	0.33526205	1
Ofd1	0.91613764	-0.126363724	0.33530675	1
Kcnq5	1.05898594	0.082683437	0.3354076	1
Clstn1	0.95616394	-0.064670098	0.33542526	1
Cdca7	1.24635099	0.317710404	0.33549153	1
Eri1	1.08683468	0.12013251	0.33553513	1
Ccdc94	1.08069058	0.111953512	0.33555351	1
Heca	0.94049522	-0.088507485	0.33568874	1

Nek2	0.78013234	-0.358209217	0.3357782	1
Itgb1bp1	1.05673521	0.079613918	0.33580731	1
Tctn2	0.88189031	-0.181328877	0.33584671	1
Tpt1	1.04654288	0.065631418	0.33601436	1
Gnpda1	1.08352811	0.115736584	0.33615195	1
Disp1	1.07834108	0.108813579	0.33616372	1
Nr1d2	0.9553692	-0.065869726	0.33622971	1
Dnaaf3	1.21789485	0.284389584	0.33623358	1
Plxnb3	0.94756467	-0.077703687	0.33625634	1
Fas	0.86762561	-0.204855456	0.33632653	1
Rnf125	0.91446822	-0.128995067	0.33646226	1
Psmd5	1.05919464	0.082967732	0.33658027	1
Ndufs7	1.05972486	0.083689744	0.33658615	1
Cnbd2	1.1963459	0.258634581	0.33676477	1
Sos2	0.9411221	-0.087546186	0.33677739	1
Bag6	0.95337121	-0.068890033	0.33678873	1
Slc43a3	1.08745277	0.120952748	0.33679505	1
Cryl1	1.14235788	0.192014695	0.33692623	1
Cyld	0.93810871	-0.092172986	0.33697878	1
Mtrf1l	0.89138795	-0.165874629	0.33699786	1
LOC100911576	1.0477761	0.067330459	0.33713684	1
Ergic3	0.94692237	-0.07868194	0.33714063	1
Rpl23a	1.04498296	0.063479424	0.33717246	1
Snx17	0.9376755	-0.09283936	0.33719179	1
Eif3c	1.0450816	0.063615589	0.33725402	1
Polr3h	1.0891755	0.12323643	0.33728041	1
Pgap1	0.90326707	-0.146775486	0.33736208	1
Kpna4	0.94325402	-0.084281756	0.33743501	1
Dctn4	1.05737611	0.080488636	0.33752043	1
Rac1	0.95487448	-0.066616996	0.33768327	1
Gbp4	1.19021451	0.251221606	0.33770995	1
Fam81a	0.90796582	-0.139290102	0.33776738	1
Dpm2	1.0824345	0.114279729	0.3378797	1
Bzw2	0.92071479	-0.11917378	0.33794796	1
Psma2	1.04684952	0.066054077	0.33812314	1
Nln	1.07620703	0.105955639	0.33812454	1
Dnaaf2	1.10260671	0.140918289	0.33813046	1
Pm20d1	0.81618296	-0.293035504	0.33815455	1
Gnai2	0.9570962	-0.063264147	0.33839294	1
Rab2b	1.06206108	0.086866741	0.33840715	1

Sdhb	0.95431317	-0.067465316	0.33840795	1
Tmem196	1.11923898	0.162518117	0.33854769	1
Bmpr1b	1.1891768	0.249963226	0.33864154	1
Ndufa2	1.06497516	0.090819783	0.33870274	1
RGD1564036	1.19970139	0.262675358	0.33871409	1
Lrg1	1.12834667	0.174210379	0.3387348	1
Ltbr	0.92936447	-0.105683602	0.33883264	1
Adh5	1.05254287	0.073878994	0.33889452	1
Tbc1d9	0.95577378	-0.065258909	0.33898159	1
Pdgfd	1.11311383	0.154601139	0.3390681	1
Igf2r	0.95492181	-0.066545479	0.33907385	1
Clec11a	1.137014	0.185250021	0.33926745	1
Palm2	0.88732793	-0.17246072	0.3392949	1
Paqr7	0.9212103	-0.118397554	0.33930176	1
Trpc5	1.20840996	0.273109982	0.33935781	1
Sestd1	0.94006992	-0.089160023	0.33945421	1
Sox13	0.91952102	-0.121045537	0.33949654	1
Atxn2l	0.95397802	-0.067972067	0.33953494	1
Rps6	1.04501195	0.063519439	0.33960189	1
Zfp397	0.86351908	-0.211700044	0.33974322	1
Kdm1b	0.91901527	-0.121839258	0.33975613	1
Rab13	0.87680927	-0.18966505	0.33981554	1
Pskh1	1.17431428	0.231818572	0.33995071	1
Ulk3	0.86387813	-0.211100294	0.34011901	1
Cep131	0.92487003	-0.112677451	0.34023427	1
Slamf8	1.29854978	0.376901326	0.34034685	1
Ints4	0.94462917	-0.082180015	0.34038672	1
Dbndd2	0.94519074	-0.081322598	0.34040852	1
Nfx1	1.05976438	0.083743548	0.34047372	1
Ccdc64	0.91993001	-0.120403994	0.34054036	1
Trpm3	0.95128836	-0.072045366	0.34058838	1
Rcor1	0.93816783	-0.092082067	0.34059868	1
RGD1308147	1.06112315	0.085592106	0.34063287	1
Zcchc17	1.0561489	0.078813241	0.34070674	1
Gypc	1.11028915	0.150935448	0.34073926	1
Cmtm7	0.88544297	-0.175528703	0.34076852	1
Zbtb33	0.91614042	-0.12635935	0.34082922	1
Ppp1r35	1.17576239	0.233596536	0.34094325	1
Grik2	1.08669307	0.11994452	0.34095987	1
Cir1	1.07621804	0.105970398	0.3410585	1

Wtap	1.04755719	0.067029011	0.34117048	1
Zfp295	0.91304379	-0.131244045	0.34119849	1
Mgat3	0.94627499	-0.0796686	0.34126018	1
Hmgn2	0.94729752	-0.078110488	0.34128767	1
Pabpc4	0.93631071	-0.094940735	0.34161376	1
Xpo6	0.9522112	-0.070646492	0.34181298	1
Fzd4	0.9287541	-0.106631422	0.34200253	1
Dtx3l	1.08179584	0.113428254	0.34209669	1
Scube1	1.08317243	0.11526293	0.34237567	1
Rragd	0.89483185	-0.16031148	0.34243078	1
Atp2b2	0.95672082	-0.0638301	0.34248105	1
Klhl31	0.66372726	-0.591337562	0.34257736	1
Sertad4	0.91825872	-0.123027405	0.34258097	1
Slc7a11	0.90562603	-0.143012663	0.34259693	1
Rnf135	1.19664231	0.258991974	0.34272542	1
Crlf2	0.87614802	-0.190753471	0.34272726	1
Cox16	1.09898263	0.13616858	0.34290896	1
Nelfe	0.92055251	-0.119428082	0.34306173	1
Gan	1.1102893	0.150935634	0.34306621	1
Nsa2	1.04946537	0.069654566	0.34314337	1
Hsd17b11	0.95368686	-0.068412458	0.3433375	1
Slc38a3	1.18579888	0.245859336	0.34364551	1
LOC691921	1.19507354	0.257099393	0.34380258	1
Sik3	0.93922169	-0.090462375	0.34408033	1
Lrwd1	0.89680119	-0.157139898	0.34426125	1
Ldb1	0.94201643	-0.086175872	0.34426389	1
Dpysl3	0.95637466	-0.064352188	0.34443853	1
Zpbp2	1.27352766	0.348830298	0.34459796	1
Jade2	0.92759352	-0.108435346	0.34459981	1
Hr	0.93096802	-0.103196479	0.34494767	1
Rere	0.95125921	-0.072089581	0.34501167	1
Slc22a6	1.18792718	0.248446402	0.34506814	1
Kcnn4	1.11748151	0.160250962	0.34508082	1
Cntnap1	0.95777221	-0.062245514	0.34511733	1
Zfp2	1.19680217	0.25918469	0.34522926	1
Ankrd10	0.92910157	-0.10609178	0.34527856	1
Anxa7	1.04615027	0.065090093	0.34563131	1
Spi1	1.17493901	0.232585873	0.34572654	1
Apoc1	1.24272125	0.313502729	0.3458979	1
Yeats4	1.07398506	0.102973929	0.3459363	1

Cdh5	1.06687711	0.093394004	0.3459488	1
Pdcd4	1.05531583	0.077674822	0.34610198	1
Zfp653	0.88563628	-0.175213777	0.34614709	1
Irak1	0.9465892	-0.079189639	0.34623919	1
Ptpn1	1.10867893	0.148841629	0.34628831	1
MAST1	0.95742845	-0.062763423	0.34631299	1
Cpa3	1.23411731	0.303479534	0.34637896	1
Rpl29	1.04423374	0.062444677	0.34641275	1
H2afz	0.94466434	-0.082126296	0.34644929	1
Nt5dc3	0.91550381	-0.127362211	0.34645348	1
Samd10	1.13749693	0.185862646	0.34646437	1
Prkab1	1.09625461	0.132582904	0.34656919	1
Slc38a9	1.10669134	0.146252904	0.34658003	1
Tmem129	0.92720793	-0.10903519	0.346619	1
Cdh15	0.94271664	-0.085103902	0.3468679	1
Mlc1	1.20002711	0.263067	0.34691193	1
Atl2	1.05819592	0.081606765	0.34695443	1
Tspan4	0.93203468	-0.101544459	0.34698049	1
Ranbp10	0.94558177	-0.080725869	0.34718715	1
Hsd17b8	1.12651073	0.171861056	0.34721671	1
Ptpn9	1.05895109	0.082635963	0.34723479	1
Pcdhb4	0.83429299	-0.261373979	0.34724233	1
Mvp	0.94538869	-0.081020486	0.34725822	1
Plac9	0.89924388	-0.153215657	0.34740608	1
Mgst1	0.94107682	-0.087615593	0.34771896	1
Sgpl1	1.06314828	0.088342823	0.34771908	1
Arhgap9	1.16780308	0.223797026	0.34786112	1
Rarg	0.93316053	-0.099802809	0.34789925	1
Prmt5	0.94519474	-0.081316488	0.34791183	1
Mpv17l2	1.13223928	0.179178881	0.34791799	1
Ppp1r12a	0.94556214	-0.080755826	0.34800571	1
Clpb	0.93428439	-0.098066328	0.34808102	1
Elovl1	0.95375601	-0.06830785	0.34808248	1
Orc3	1.07847702	0.108995432	0.34816425	1
Trip11	0.94936435	-0.074966222	0.34817529	1
G3bp2	1.04514619	0.063704751	0.34840813	1
Prtfdc1	1.0739248	0.102892973	0.34848363	1
Tmem101	1.08435029	0.116830877	0.34849197	1
Hrh3	0.85890924	-0.219422398	0.34856027	1
Gata1	1.45193604	0.537977906	0.34858163	1

Srf	1.08038919	0.111551106	0.34863068	1
Mchr1	1.24335993	0.314243992	0.34890155	1
Tmem14c	1.05293776	0.074420154	0.34893739	1
Epha3	0.83076622	-0.267485533	0.3489518	1
LOC687399	0.81439108	-0.29620634	0.34929397	1
Bax	1.08646555	0.119642429	0.3493071	1
Gpr176	1.08206323	0.113784801	0.3494145	1
Ppp1r11	0.95364673	-0.068473159	0.34941563	1
S1pr4	0.80280776	-0.316873534	0.34957677	1
Rsl1d1	1.06772228	0.094536448	0.3496378	1
Il13ra2	1.20886333	0.273651151	0.34974984	1
Cacnb4	1.05575587	0.078276263	0.34993165	1
Gpsm3	1.17173372	0.228644746	0.34994162	1
Lsr	1.1371134	0.18537614	0.34997084	1
Lrrc16a	0.93782993	-0.092601767	0.34998954	1
Atp6v1g1	1.04413711	0.062311172	0.3500125	1
Dnm1l	1.0438427	0.061904325	0.35012348	1
Zfyve19	1.10137502	0.139305789	0.35020938	1
C2cd3	0.92524317	-0.112095516	0.35022059	1
Lims2	0.94636639	-0.079529262	0.35036882	1
Dusp22	1.08920984	0.123281925	0.35039843	1
Slc16a1	1.04741386	0.066831606	0.35043795	1
Adipor1	1.04408658	0.062241349	0.3505019	1
Ccdc163	1.14068185	0.189896463	0.35074747	1
Jrk	1.15944685	0.213436685	0.35079208	1
Stim1	0.95187998	-0.071148414	0.35095008	1
Pdgfa	0.92467787	-0.112977238	0.35114135	1
Slc35e2b	0.91290525	-0.131462969	0.35122303	1
Vps26b	0.9505326	-0.07319199	0.35130984	1
Med10	1.07127759	0.099332356	0.35131871	1
Xpo4	0.94258691	-0.08530245	0.35151219	1
Vrk1	1.09013156	0.124502252	0.35155327	1
Nthl1	1.14428606	0.194447753	0.35162505	1
Lrrc46	0.85377244	-0.22807651	0.3516574	1
Shoc2	0.95035733	-0.073458035	0.35165877	1
Fam168a	0.95596998	-0.064962774	0.3516892	1
Wdr60	0.917478	-0.124254527	0.35172252	1
Gpr182	1.23564967	0.305269772	0.35186527	1
Zfp867	1.22611489	0.29409417	0.35196467	1
Naa35	1.05902053	0.082730552	0.3519838	1

Arhgap39	0.94812896	-0.076844794	0.35200521	1
Gosr2	1.05325319	0.07485228	0.35202525	1
RGD1307704	0.87918161	-0.185766891	0.35210538	1
Ephb1	0.92513005	-0.112271916	0.35214934	1
Psma3	0.94182568	-0.086468039	0.35215116	1
Hmgcs2	0.94425816	-0.082746744	0.3524781	1
Rrp7a	1.05170749	0.072733507	0.3524856	1
Arrb2	1.06600145	0.092209398	0.35248722	1
Zfp597	1.12311679	0.167507953	0.35250588	1
Gorab	1.09773731	0.134532863	0.35267682	1
Rph3a	0.95828041	-0.061480218	0.35284485	1
Cx3cl1	0.87351359	-0.195097948	0.35297746	1
Tmco1	1.05363643	0.075377135	0.35301129	1
Ebf3	0.94405482	-0.083057456	0.35316566	1
Steap2	0.90219087	-0.148495408	0.35317229	1
Angptl1	1.15163294	0.203680956	0.35318246	1
Atp23	1.16816494	0.224243989	0.35329155	1
Samd8	1.07812538	0.108524968	0.35331786	1
Ror2	0.84967303	-0.235020324	0.35344372	1
Phlpp2	0.93144104	-0.10246365	0.35361664	1
Serpinb10	1.2345615	0.303998702	0.3537971	1
Hba-a1	0.92862858	-0.106826405	0.35380854	1
Zfp57	0.85746507	-0.221850188	0.3538162	1
Ptger2	0.80599267	-0.311161375	0.35401438	1
Ogfod2	1.13086905	0.17743188	0.3542824	1
Rcor2	0.90560809	-0.143041249	0.35428469	1
Elp4	0.88461539	-0.176877748	0.3543091	1
Cabin1	0.94349342	-0.083915634	0.35434476	1
Slc39a10	0.95459309	-0.067042208	0.35439745	1
Adap1	0.95539697	-0.065827793	0.35441563	1
Bcs1l	1.09907844	0.136294348	0.35446874	1
Ythdf1	1.04972309	0.070008806	0.35458479	1
Klra1	1.18201679	0.241250529	0.35459341	1
Nepro	0.8718995	-0.197766237	0.35482125	1
Smarcal1	1.08489701	0.11755809	0.35489669	1
Ccdc127	0.93410755	-0.098339426	0.35493825	1
Taf9	0.94945597	-0.074826999	0.35497886	1
Chrna5	1.13104202	0.177652532	0.35498337	1
Plekhm1	0.92518128	-0.112192014	0.3550414	1
Otub1	0.95392009	-0.068059673	0.35507324	1

Thumpd2	1.13501797	0.182715143	0.35511429	1
Arl2	1.0669781	0.093530571	0.35517393	1
Kcnn3	0.89661654	-0.157436989	0.35521892	1
Ccdc71	1.08437224	0.116860092	0.35532777	1
Acp2	1.05377784	0.075570752	0.3555734	1
Rp2	1.11198412	0.153136182	0.35565392	1
Gpr173	0.81078008	-0.302617452	0.35573795	1
Sf3b4	0.9285437	-0.106958288	0.35588054	1
Snx9	0.93385793	-0.098725005	0.3560187	1
Eif4b	1.04367476	0.0616722	0.3560216	1
Eif3d	0.94640499	-0.079470418	0.35605925	1
Lactb2	1.06051521	0.084765308	0.35613509	1
Gopc	0.95266082	-0.069965433	0.35624317	1
Zfp592	0.94229536	-0.085748755	0.35625957	1
Gpx4	1.04328027	0.061126774	0.35628135	1
Mpg	1.15224402	0.204446274	0.35628296	1
Retnlg	0.75703571	-0.401566735	0.35629305	1
Alms1	1.06288777	0.087989269	0.35631396	1
Cyyr1	1.07422401	0.103294868	0.35641432	1
Atp5s	0.91770252	-0.123901529	0.35654563	1
Wdr20	0.92888559	-0.106427176	0.35660283	1
Arel1	1.04452011	0.062840264	0.3566467	1
Arrdc1	0.93967079	-0.089772693	0.35673261	1
Sgcg	1.25451705	0.32713208	0.35680514	1
Cnot7	1.05019695	0.070659916	0.35700042	1
Prr5	1.09099236	0.125641005	0.35711592	1
Rplp2	1.06549238	0.091520272	0.35722506	1
Xbp1	0.9539727	-0.067980118	0.35729521	1
Dffb	0.84378474	-0.245053104	0.35730827	1
Sh3bgr	1.11955002	0.162918989	0.35748701	1
Bhlhb9	1.05641652	0.07917877	0.35760185	1
Usp10	0.93938495	-0.09021161	0.35768664	1
Ubxn7	1.21387343	0.279618004	0.35784385	1
Myo1e	0.95696693	-0.063459023	0.35786592	1
Lrrc45	1.06328118	0.088523167	0.3578981	1
Smc2	0.93416375	-0.098252637	0.35790414	1
Rap2b	0.80739974	-0.30864497	0.35791862	1
Wdr45b	1.06941067	0.096815982	0.35795	1
Ttc9	0.9576673	-0.062403554	0.35820768	1
Neurl1	0.87718632	-0.189044776	0.35836806	1

Kcnn1	0.90135821	-0.149827527	0.35841674	1
Nae1	1.06038856	0.084593007	0.35851576	1
Fxyd7	1.0437023	0.061710262	0.35868604	1
Polk	1.09558149	0.131696793	0.35871808	1
Hpgds	1.33483028	0.416656319	0.35889629	1
Blcap	1.0568872	0.079821405	0.35891896	1
Eif2s2	1.04657969	0.065682164	0.35897824	1
Tmtc2	0.93423612	-0.098140865	0.35913684	1
Gga3	0.94516258	-0.081365575	0.35914909	1
Spata5	0.90660976	-0.141446396	0.35927498	1
Ier5	1.12714526	0.172673457	0.35949474	1
Araf	1.04751327	0.066968525	0.35954773	1
Ipmk	0.93196368	-0.101654364	0.35977745	1
Entpd4	1.04317887	0.060986546	0.3597801	1
Tmem229b	0.95894426	-0.06048113	0.36006619	1
Smc6	0.9515288	-0.071680778	0.36020785	1
Clgn	0.95621568	-0.064592028	0.36025576	1
Col9a1	0.79511332	-0.330767608	0.36028098	1
Aars	1.04315671	0.060955899	0.36029973	1
Wash1	0.93992466	-0.089382971	0.36034999	1
Atg7	1.10600428	0.145356975	0.36036463	1
Calm3	1.04318175	0.060990538	0.36038016	1
Cpsf6	0.95465965	-0.066941607	0.36039252	1
Aldh4a1	1.07939735	0.110226052	0.36049215	1
Reep1	0.95771125	-0.062337351	0.36049399	1
Dazap1	1.07539716	0.104869571	0.36050134	1
Sh3kbp1	1.048849	0.068806996	0.36072787	1
Pus1	0.91818381	-0.123145098	0.36075053	1
Mypop	0.92280244	-0.115906281	0.36082999	1
Med26	0.86505649	-0.20913375	0.36102069	1
Rap2a	1.22143382	0.2885757	0.3610261	1
Tmem127	0.95838051	-0.061329523	0.36115923	1
Cnpy3	0.94295554	-0.08473834	0.36120961	1
Fam196a	0.87237754	-0.196975465	0.36121449	1
RGD1310553	0.94016009	-0.089021657	0.36124532	1
Stoml2	1.06063454	0.084927641	0.36125526	1
Osbpl7	1.07910519	0.109835498	0.36125621	1
Tm6sf1	1.04489198	0.063353806	0.36128807	1
Zswim8	0.95040453	-0.073386376	0.36136319	1
Galns	1.1691511	0.225461393	0.36138967	1

Mars	1.04866777	0.068557685	0.36142688	1
RGD1311756	1.10619634	0.145607471	0.3614777	1
Lage3	1.05325415	0.074853606	0.361517	1
Echdc2	1.20320884	0.266887072	0.36164299	1
Cyp27a1	1.08007602	0.11113286	0.36169727	1
Lsm8	1.08581909	0.118783759	0.36199998	1
Pdxp	0.92526576	-0.112060294	0.36206183	1
Tmem161a	1.09012026	0.124487301	0.36209009	1
Pbxip1	1.04841083	0.068204164	0.36219343	1
Zbtb38	0.94199551	-0.086207907	0.36243449	1
Syn3	1.11175829	0.152843158	0.36247512	1
Sema4b	0.93213614	-0.101387411	0.36259043	1
Jmjd1c	1.04756566	0.067040673	0.36259963	1
Trmt112	1.06297206	0.088103678	0.36280731	1
Pomgnt1	0.94760077	-0.077648725	0.3629435	1
Ezh1	1.0442177	0.062422524	0.3630443	1
Itga7	0.95918113	-0.060124825	0.36372357	1
M6pr	1.04339283	0.061282427	0.36373958	1
Hoxa7	1.14643904	0.197159639	0.36374439	1
Arl8a	0.95916665	-0.060146597	0.36389475	1
Jmjd6	0.91163765	-0.133467583	0.36394333	1
Dhrs4	1.1007961	0.13854727	0.36394386	1
Ckap2	0.80733027	-0.308769107	0.36395137	1
Slc39a14	0.93585986	-0.095635578	0.36397619	1
Tnks	0.944074	-0.083028142	0.36398607	1
Zbed4	0.91501416	-0.128134021	0.36404266	1
Tbrg4	0.93654938	-0.094573026	0.36404762	1
Mfsd8	0.89078774	-0.166846392	0.36405325	1
Eif3b	0.95585486	-0.06513652	0.3642587	1
Cenpl	1.12136197	0.165252047	0.36427708	1
Arhgap30	0.87654614	-0.190098061	0.36432036	1
Gdap1	1.04246523	0.059999272	0.36444391	1
Rnf152	1.13051963	0.176986043	0.36468398	1
Mrps33	1.06872363	0.095888821	0.36487826	1
Tspan1	1.19466907	0.256611041	0.36493934	1
Fn3krp	1.0756818	0.105251373	0.36506707	1
LOC688765	0.86210426	-0.214065737	0.36508745	1
Paf1	1.06331357	0.088567112	0.36510995	1
Trpc3	0.92668282	-0.109852469	0.36524931	1
Ccdc174	1.0925993	0.127764407	0.36528426	1

Tmem120b	0.91476747	-0.128523025	0.36533511	1
Jakmip3	0.93019745	-0.104391106	0.36534298	1
Pagr1	1.06119728	0.085692877	0.36536348	1
Mgll	0.94895017	-0.075595756	0.36540325	1
Klhl28	0.91568031	-0.127084093	0.36555106	1
Fars2	1.09128704	0.12603062	0.36558721	1
Pianp	0.92930115	-0.105781903	0.36568389	1
Ik	1.04436279	0.062622961	0.36575053	1
Znhit1	0.93391694	-0.098633852	0.36585736	1
Tmem135	0.94207505	-0.086086105	0.36601054	1
Atp7a	0.92871486	-0.106692376	0.36622001	1
Mettl4	0.8237644	-0.279696308	0.36632404	1
Stmn2	1.04548249	0.064168902	0.36635621	1
Pde12	0.89381017	-0.161959638	0.36637187	1
Nme6	1.17948852	0.238161377	0.36655668	1
Chrdl2	0.85045978	-0.233685082	0.36658212	1
Kcnf1	0.9145989	-0.128788918	0.36659607	1
Sf3a1	0.94655611	-0.079240062	0.36667307	1
Atoh8	1.22163997	0.288819173	0.36667462	1
Actr10	1.04377327	0.061808366	0.36675459	1
Pstpip1	0.87533387	-0.192094693	0.36678456	1
Aqp11	0.90455053	-0.144727004	0.3668035	1
Mx1	1.09112249	0.125813069	0.36684617	1
Rhobtb1	1.1339036	0.181297994	0.36685156	1
Timm13	1.05651575	0.079314268	0.36691021	1
Fap	1.10223071	0.140426226	0.36697358	1
Cyp2j10	0.91448697	-0.128965474	0.3670922	1
Chmp6	0.93970475	-0.089720549	0.36720302	1
Mef2d	0.92611987	-0.110729159	0.36721726	1
St8sia1	1.08187176	0.113529497	0.36725873	1
Vps53	1.04706091	0.066345367	0.36739786	1
Sulf1	1.06780676	0.094650582	0.36754844	1
Ddn	0.90239571	-0.148167893	0.36761123	1
Wwp2	0.93340996	-0.099417231	0.36773105	1
Ilf3	1.06272862	0.087773231	0.36774938	1
Fgfr1	0.95795767	-0.061966187	0.36786881	1
Mrm1	0.88636273	-0.174030868	0.36789883	1
Pik3cd	0.9445361	-0.08232216	0.36792123	1
Mepce	0.93111824	-0.102963716	0.3679942	1
RGD1560212	1.04501246	0.063520145	0.36799715	1

Tle1	1.0612903	0.085819335	0.36807695	1
Cpz	1.12551038	0.170579367	0.36813657	1
Max	0.90846555	-0.138496288	0.36824241	1
Scrt1	0.94081642	-0.088014859	0.36827571	1
Sox12	0.88894421	-0.169835213	0.36829719	1
Dse	1.06425293	0.089841061	0.36837787	1
Ubxn6	0.94472927	-0.082027134	0.3684737	1
Slc17a5	1.06860064	0.095722782	0.36849137	1
Ccl6	1.12928684	0.175411984	0.36852025	1
Ash1l	0.95822875	-0.061557989	0.36869089	1
Fam46b	1.23888791	0.309045661	0.36880821	1
Praf2	1.04362386	0.061601836	0.36884954	1
Ech1	1.04833308	0.068097169	0.36899422	1
Flot1	1.04319958	0.061015197	0.36905294	1
LOC306079	1.14062012	0.189818382	0.36914015	1
Mafg	0.93827841	-0.091912025	0.36917018	1
Peli3	1.11752539	0.160307612	0.36932108	1
Stap2	0.85042931	-0.233736769	0.36947222	1
Mical3	0.94159562	-0.086820485	0.36955213	1
Dnase1l1	1.08891439	0.12289053	0.36958625	1
Lfng	0.8965884	-0.157482263	0.36993424	1
Pygm	1.09365353	0.129155763	0.37006688	1
Snw1	1.05607637	0.078714163	0.37009088	1
Hdac11	0.94494773	-0.081693567	0.37013539	1
Tceb3	1.06231454	0.087210997	0.37016667	1
C2cd2l	0.9497472	-0.074384544	0.37038436	1
Smn1	0.92051008	-0.119494568	0.37041973	1
Actr3	0.95965824	-0.059407381	0.37063023	1
Dcn	1.04435825	0.062616683	0.3708452	1
Cep70	1.10554446	0.14475705	0.37089085	1
Pdzd2	0.94364056	-0.083690663	0.37094629	1
Fgfbp3	1.12894595	0.174976421	0.37107898	1
Fbxl19	0.92874527	-0.106645144	0.37113199	1
H2afv	1.09125218	0.125984534	0.37125028	1
Cux2	0.89663553	-0.157406428	0.3712898	1
Fam120b	0.95412105	-0.067755774	0.37129247	1
Mroh7	1.15884695	0.212690046	0.37144128	1
Sf3b2	0.95845194	-0.061221996	0.37152404	1
Prima1	0.9255101	-0.111679366	0.37154898	1
Ilvbl	1.05411667	0.076034549	0.3715518	1

Ndufa6	1.04983683	0.070165117	0.37158089	1
Alkbh5	0.92239585	-0.116542075	0.37177226	1
Nfat5	0.95855076	-0.061073268	0.37194662	1
Dnaja3	1.05307655	0.074610318	0.37203041	1
Unc5a	0.85758686	-0.221645291	0.37231945	1
Tspan33	1.16839764	0.224531351	0.37235425	1
Wdr12	1.06640622	0.092757099	0.3724309	1
Plcd1	1.07239792	0.100840332	0.37259152	1
Slc22a8	1.11319573	0.154707278	0.37286238	1
LOC100909675	0.78192055	-0.354906076	0.37291652	1
Gng12	1.10798978	0.147944571	0.37302301	1
Slc5a6	0.95402277	-0.06790439	0.37302569	1
Apol3	1.13296723	0.180106136	0.3730403	1
Nlk	0.94756019	-0.077710511	0.37313074	1
Suclg2	1.05793752	0.081254421	0.37333772	1
Atf7	0.92624686	-0.110531356	0.3734463	1
Pip4k2c	1.06896334	0.096212373	0.37358189	1
Tor1b	0.92927602	-0.10582091	0.373672	1
Xab2	0.94062305	-0.088311415	0.37373922	1
Fam135a	1.05620274	0.078886784	0.37380546	1
Dnajc25	0.87564144	-0.191587866	0.3738311	1
Dap3	0.93940181	-0.09018572	0.37388831	1
Zbtb41	0.91800775	-0.123421766	0.37404153	1
Rarres1	1.0468755	0.066089877	0.37415371	1
Zcchc7	0.93501235	-0.096942667	0.37418259	1
Card6	1.08341663	0.115588143	0.3742284	1
Ssu72	1.04304356	0.060799405	0.37428414	1
Gale	1.0730928	0.101774842	0.37428575	1
Glb1l2	1.10780831	0.147708267	0.37452146	1
Rpl24	1.04201152	0.05937123	0.37456997	1
Abcc10	0.89831163	-0.154712077	0.37467799	1
Hsd17b4	1.04420442	0.06240417	0.37470554	1
Mtpn	0.95736689	-0.062856184	0.37487108	1
Mrpl2	0.92748488	-0.108604336	0.3750177	1
Dab1	0.8764257	-0.190296305	0.37502353	1
Faf2	1.05899598	0.082697117	0.37506838	1
Zfp598	0.93927645	-0.090378257	0.37509258	1
Cyb5a	1.04765196	0.067159524	0.37521107	1
Prmt7	1.07423526	0.103309983	0.37532887	1
Pemt	1.1671695	0.223014091	0.37541716	1

Commd2		1.07791698	0.108246062	0.375419	1
Nucb2		1.05277326	0.074194756	0.37558353	1
RT1-CE2		1.10972438	0.150201397	0.37627919	1
Abra		1.25484598	0.327510295	0.37629163	1
Mipep		0.92889438	-0.106413531	0.37632154	1
Rarb		1.09843033	0.135443366	0.37636999	1
Man2c1		1.06044345	0.084667694	0.37644202	1
Slc2a13		0.94100122	-0.087731501	0.37651907	1
Tnnc1		1.21976756	0.286606254	0.37653399	1
Prdx5		1.04262207	0.060216308	0.37663619	1
LOC297756		1.04275332	0.060397912	0.37671275	1
Ppm1a		1.04306867	0.060834142	0.37680417	1
Zfp668		0.88407051	-0.177766653	0.37694233	1
Scn3a		1.09289506	0.128154879	0.37695712	1
Lemd2		0.94163308	-0.086763086	0.37696236	1
Bmx		1.28349288	0.360075288	0.37703074	1
Prpsap1		0.94413892	-0.08292895	0.37707732	1
Chd1		1.06299621	0.088136447	0.37717532	1
Homer2		0.90480125	-0.144327173	0.37721121	1
Ushbp1		1.15890148	0.21275793	0.3773263	1
Scp2		0.96022739	-0.058552013	0.37740767	1
Spsb1		0.91783195	-0.123698071	0.37749625	1
Stmn3		1.04256532	0.060137776	0.37773418	1
Phactr1		0.92910086	-0.10609287	0.3779125	1
Ercc3		1.04864474	0.068525998	0.3781993	1
Pdlim7		0.94451384	-0.082356159	0.37835997	1
RGD1312005		1.06327555	0.088515529	0.37837527	1
Dhrs1		1.07832028	0.108785743	0.37840832	1
Thoc6		1.0950056	0.130938251	0.37852808	1
Cers2		0.95624278	-0.064551149	0.3786434	1
Asun		1.07663839	0.106533769	0.37882728	1
Terf2ip		1.04979805	0.070111826	0.37890484	1
	5-Sep	0.96035808	-0.058355669	0.37922485	1
Mnt		1.06509139	0.09097723	0.37928702	1
Pi4k2a		0.94864834	-0.076054715	0.37950926	1
Gabarapl2		1.04122287	0.058278901	0.37964941	1
Slc6a15		1.04115475	0.058184518	0.37984395	1
Sord		1.04872897	0.068641881	0.37988166	1
Camkk2		0.89706033	-0.156723079	0.37991505	1
Zcchc11		1.08761224	0.121164291	0.37998203	1

Dpm1	1.05577427	0.078301406	0.38022822	1
Zbtb40	1.10852999	0.1486478	0.3802466	1
Ccnl1	1.05901839	0.082727638	0.38040515	1
Mknk1	1.08031445	0.111451297	0.38040837	1
Rad9b	1.20348014	0.267212332	0.38049387	1
Scly	0.90294509	-0.147289832	0.38071982	1
Calr	1.04184467	0.0591402	0.38072791	1
Ascc1	0.93258225	-0.100697118	0.38108805	1
Sp4	1.10061444	0.138309166	0.3810922	1
Baiap3	1.2897967	0.367143679	0.38109585	1
Atp13a4	1.15237459	0.204609754	0.38113241	1
RGD1559786	1.06019142	0.084324766	0.38116245	1
Fmo2	0.94455979	-0.082285975	0.38118181	1
Cry1	0.90806493	-0.139132631	0.38150589	1
LOC310926	1.1670561	0.222873916	0.38158357	1
Papolg	1.0740614	0.103076465	0.38158968	1
Cxxc4	1.07830042	0.108759182	0.38167994	1
Cwc15	0.94835842	-0.07649568	0.38171879	1
Sft2d1	0.93376125	-0.098874368	0.38174194	1
Pgp	0.9504691	-0.073288364	0.38182441	1
Syp	0.96019895	-0.058594742	0.38186027	1
Clec1a	1.11445744	0.156341527	0.38186389	1
Trmt2a	1.07833819	0.108809706	0.38190997	1
Atp6v0e2	1.04304341	0.060799198	0.38213168	1
Sfmbt1	1.08065285	0.111903151	0.38213648	1
Spryd7	1.05482486	0.077003471	0.38216381	1
Wasl	0.95762655	-0.06246495	0.38230598	1
Zdhhc2	1.04114563	0.058171874	0.38245887	1
Nmt1	1.04540059	0.064055878	0.38251767	1
Ankrd40	0.96046356	-0.058197223	0.38264559	1
RGD1563986	1.12994718	0.176255337	0.38272137	1
Ammecr1l	0.94290007	-0.084823214	0.38283293	1
Irs1	1.13648678	0.184580904	0.3828441	1
Pgam2	0.75623704	-0.403089585	0.38285283	1
Ap3m1	1.04611656	0.065043614	0.38297328	1
Uba3	1.04243307	0.059954755	0.38297682	1
Ttll3	0.83859316	-0.253957035	0.38307517	1
Cd69	0.78806906	-0.343606032	0.38312493	1
Kcnh8	1.15293564	0.205311978	0.38321703	1
Rqcd1	1.06698553	0.093540615	0.38334181	1

Hira	0.93696401	-0.093934461	0.3833605	1
Tgfbr1	1.05927767	0.083080819	0.38348907	1
Atp1b2	0.95706239	-0.063315126	0.3835886	1
Edem1	0.95434558	-0.067416319	0.38369043	1
mrpl24	1.06155357	0.086177183	0.38389824	1
Gltscr2	1.05041175	0.070954955	0.38391382	1
Zdhhc15	0.942618	-0.085254858	0.38400783	1
Rnpepl1	0.93095188	-0.103221492	0.38423727	1
Dlg3	0.88825325	-0.170957029	0.38441938	1
Srgn	1.07842982	0.10893229	0.38444077	1
Wasf2	1.09642396	0.132805761	0.3847504	1
Dek	1.05667162	0.079527103	0.38480956	1
Wdr81	0.93387292	-0.098701854	0.38495016	1
Bckdhb	1.06887234	0.09608955	0.38498444	1
Cbx2	0.87949393	-0.185254477	0.38505645	1
Shank2	0.93830891	-0.091865134	0.38516155	1
Nedd9	1.06918398	0.096510131	0.38517867	1
Nde1	1.09965722	0.137053888	0.38543762	1
Foxs1	1.12208301	0.166179411	0.38560357	1
Tmsbl1	1.06599335	0.092198443	0.3856362	1
Sntb1	0.92882215	-0.106525723	0.38570148	1
Plekhm2	0.94972097	-0.07442438	0.38575761	1
Dpp4	0.93504674	-0.096889605	0.38576944	1
Fam173a	0.91880981	-0.122161832	0.38586001	1
Fgf1	1.04136795	0.058479911	0.38586244	1
Ccdc28a	1.05494662	0.077169994	0.38601518	1
Kif20a	1.20524237	0.269323295	0.38604126	1
Nup210l	1.29652741	0.374652704	0.3860427	1
Eif3f	0.95518996	-0.066140426	0.38609392	1
Mapk8ip1	0.96046709	-0.058191921	0.38630885	1
Elp5	1.0776953	0.107949343	0.38636764	1
Carm1	0.94850434	-0.076273721	0.38655468	1
Flrt2	1.11074103	0.15152249	0.38674353	1
Cenpq	1.168504	0.224662676	0.38692535	1
Bcas1	1.05600849	0.078621428	0.38717186	1
Mov10	1.13582316	0.183738239	0.38731103	1
Cul5	0.94523211	-0.081259459	0.38741065	1
Ociad2	1.12000346	0.163503188	0.38748886	1
Tubgcp3	1.05795409	0.081277026	0.38751617	1
Rbfa	0.92493587	-0.112574755	0.38752799	1

Nsmce1	0.92494643	-0.11255829	0.38771591	1
Pla2g12a	0.91993488	-0.120396349	0.38780601	1
LOC294154	0.96048556	-0.058164171	0.38787608	1
Ccnf	0.85164474	-0.231676349	0.38787933	1
Rhoc	0.95000772	-0.073988856	0.38794591	1
Card19	0.93741479	-0.093240539	0.38795734	1
Id3	1.04948577	0.069682604	0.38804537	1
Prkcdbp	0.95192088	-0.071086421	0.38816089	1
Ptprm	1.0518008	0.072861501	0.38819392	1
Uqcr11	1.04498352	0.06348019	0.38822633	1
Xylb	0.93832329	-0.091843021	0.38842018	1
Cyp4f18	1.39526372	0.480537834	0.38849396	1
Mrgprf	0.9156189	-0.127180844	0.38854408	1
Foxm1	1.17775344	0.236037541	0.38887363	1
Hgh1	0.92759345	-0.108435457	0.38899494	1
Nat9	0.92802245	-0.107768387	0.38910395	1
RGD1304694	0.95286394	-0.069657869	0.38936132	1
Rpp21	0.89983871	-0.152261658	0.38937922	1
Prkd3	1.05884436	0.082490543	0.38942214	1
Map4k2	1.06013669	0.084250296	0.3894263	1
Hspa12b	1.11121571	0.152138905	0.38952072	1
Triqk	1.06118899	0.085681608	0.38966093	1
Gba	1.04199252	0.059344925	0.3896766	1
Mitf	1.09008976	0.124446928	0.39001863	1
Hsf2	1.05236793	0.073639194	0.39004535	1
Camk4	0.91572522	-0.127013337	0.39008915	1
Hey1	0.88286231	-0.179739646	0.39016737	1
Zfp777	0.92802475	-0.107764811	0.39022916	1
Npl	0.92078443	-0.119064663	0.39028301	1
Myo9a	0.95978989	-0.059209483	0.39029497	1
Alg3	1.08095489	0.112306323	0.39052932	1
Ccdc115	1.08178359	0.113411921	0.39053302	1
Usp12	0.94097732	-0.087768142	0.39053925	1
Btbd6	0.95494029	-0.066517568	0.39057512	1
Sdk2	0.89310944	-0.163091122	0.39071379	1
Sv2b	1.04075814	0.057634836	0.39071939	1
Usp36	0.92762561	-0.108385443	0.39076734	1
Zfp580	1.11198708	0.153140028	0.39078055	1
Plp2	0.93778343	-0.092673307	0.39083663	1
Cmtm3	1.1067838	0.146373432	0.39092114	1

Ado	0.88571903	-0.175078984	0.39111264	1
Trem2	1.14788448	0.198977466	0.39114625	1
Usf2	0.94488679	-0.081786611	0.39118742	1
Sf3b6	1.07619172	0.105935115	0.39136251	1
Ppfibp2	1.06666382	0.093105555	0.39153803	1
RGD1307554	0.9119166	-0.133026211	0.39155686	1
Pcdhb9	1.1146792	0.156628568	0.39166345	1
Nab2	0.91176142	-0.133271734	0.39176295	1
Plaa	0.95514173	-0.066213269	0.39189322	1
Sstr2	0.81270999	-0.29918747	0.39190619	1
Csf2ra	1.14424497	0.194395953	0.39196003	1
Fmo1	0.94647151	-0.079369018	0.39196422	1
Ifngr2	1.06441091	0.090055209	0.39207615	1
Slc39a9	0.91211547	-0.132711613	0.39210644	1
Pex10	1.13031571	0.176725784	0.39218195	1
Snapc2	0.94423166	-0.082787236	0.39226204	1
Lrrtm3	1.12720611	0.172751341	0.39229791	1
Atp6v1g2	1.04025611	0.056938758	0.39245832	1
Myo1c	0.95220453	-0.070656604	0.39252652	1
Iba57	1.12234547	0.166516821	0.39260806	1
Ghitm	1.04011724	0.056746149	0.39260834	1
Mmp16	0.90505631	-0.143920538	0.39277961	1
Gcdh	1.05499654	0.077238265	0.39281353	1
Atp5l	1.04035244	0.057072356	0.39282661	1
Lmo4	0.95109356	-0.072340822	0.39283479	1
Zfp521	1.05882038	0.082457871	0.3928865	1
RGD1307315	0.88921944	-0.169388604	0.39301731	1
Sesn1	1.05426731	0.076240715	0.39317711	1
Naaa	1.07472313	0.103965042	0.39327725	1
Pop4	1.06331676	0.088571433	0.39329784	1
Tmem88b	0.84818227	-0.237553769	0.39335287	1
LOC100910973	1.06951526	0.09695707	0.39336513	1
Ddr1	0.95141443	-0.071854184	0.39358563	1
Prr16	0.83158768	-0.266059706	0.39368474	1
Rida	1.06751354	0.094254365	0.39384419	1
Lbhd1	1.08635606	0.119497035	0.3938967	1
Nrg1	1.04201219	0.059372158	0.39391984	1
Tpr	1.04058487	0.057394633	0.3940253	1
Clec7a	1.13814964	0.18669025	0.39415082	1
A2m	0.95547447	-0.065710765	0.39415672	1

Gls2	0.94616223	-0.079840524	0.39453109	1
N4bp2l2	1.06244183	0.087383856	0.39468029	1
Zfp428	1.09715572	0.133768309	0.39476144	1
Rab10	0.96178025	-0.056220786	0.39491052	1
Diexf	1.06608495	0.092322405	0.39493711	1
Usp21	0.92199897	-0.117162962	0.39495289	1
Wsb1	1.04648359	0.065549687	0.39495615	1
Smcr8	0.93510488	-0.096799904	0.39503843	1
Tex30	1.11105282	0.151927399	0.39506615	1
Nacad	0.96181459	-0.056169278	0.39539581	1
Rangrf	0.89112048	-0.1663076	0.39543738	1
Skiv2l	0.94458642	-0.082245302	0.3954876	1
Clec4a	1.2677886	0.342314195	0.39549705	1
Cst6	1.12461014	0.169424965	0.39556462	1
Sema3d	1.05606205	0.078694603	0.39557165	1
C1qtnf6	0.87360076	-0.194953977	0.39573331	1
Slc8a1	1.06516417	0.09107581	0.3957868	1
Zbtb39	0.91814138	-0.123211772	0.39582441	1
Polr3k	1.07511834	0.104495473	0.39584623	1
Fbxo31	0.95367978	-0.06842317	0.39586252	1
Srp19	1.0548917	0.077094896	0.39590152	1
Cldn19	0.95257484	-0.070095646	0.39607639	1
Fbxo21	1.04575706	0.064547745	0.39614781	1
Fez2	1.05803329	0.081385016	0.39624019	1
Tomm40l	1.08145581	0.112974721	0.39634464	1
Trub1	0.8960174	-0.158401349	0.39641739	1
Gstk1	1.06729518	0.093959236	0.39646489	1
Rpusd4	1.08582258	0.118788391	0.39648947	1
Sncg	1.04217167	0.059592944	0.39649292	1
Gcc2	1.04389051	0.061970406	0.39656628	1
Mcat	0.91351092	-0.130506124	0.39673757	1
Zic2	1.07910995	0.109841872	0.396793	1
Taf1	0.9605396	-0.058083006	0.39687236	1
Etaa1	1.07427015	0.103356842	0.39691469	1
Parp2	1.08538612	0.118208368	0.39701573	1
Sec13	1.05365846	0.075407296	0.39702164	1
Kif5a	0.96055496	-0.058059934	0.39706996	1
Sp110	1.0801665	0.111253705	0.3972094	1
Mettl2b	0.90060374	-0.151035625	0.39721733	1
Clvs2	1.11978288	0.163219033	0.3973814	1

Shq1	0.84164497	-0.248716296	0.39760681	1
Birc6	0.96194006	-0.05598109	0.39761537	1
Zfp622	1.06181777	0.086536197	0.39787767	1
Zfand4	1.12744431	0.173056177	0.39789769	1
Kank3	0.90973493	-0.136481844	0.39790042	1
Adk	1.04115362	0.058182953	0.39799756	1
Zfyve21	0.89731393	-0.15631529	0.39800929	1
A4galt	0.93955963	-0.089943367	0.39808749	1
Depdc7	1.13261607	0.179658899	0.39814607	1
Acvrl1	1.08202812	0.113737992	0.3982217	1
Elmsan1	1.1068972	0.146521246	0.39824503	1
Polr1d	0.93771293	-0.092781773	0.39832207	1
Efnb2	1.09506392	0.131015083	0.39834659	1
Szrd1	0.94712309	-0.078376156	0.39837527	1
Vps16	1.05399584	0.075869175	0.39858126	1
Enpp1	0.92517964	-0.112194583	0.39867467	1
Fjx1	0.84918601	-0.23584749	0.39870531	1
Srpx	1.07057338	0.098383683	0.39875719	1
Tysnd1	0.9154553	-0.127438649	0.39882942	1
Poldip3	1.04626879	0.065253533	0.39884441	1
Brca1	1.12539327	0.170429241	0.39887627	1
Grhpr	1.11626665	0.158681697	0.39898883	1
Tbrg1	1.06010586	0.084208336	0.39910847	1
Aatf	1.0802277	0.111335447	0.39913848	1
Kif13a	0.94686835	-0.078764246	0.3991965	1
Fgd2	0.87212445	-0.197394069	0.3992966	1
Taf3	0.92362229	-0.114625104	0.39932528	1
Mad2l2	0.89193183	-0.164994639	0.39945788	1
RGD1566320	1.07479215	0.104057691	0.39953802	1
Arsk	1.09315222	0.128494304	0.39967401	1
Serhl2	0.8419419	-0.248207417	0.39976075	1
Uso1	0.96132965	-0.056896857	0.39981189	1
Ppp2r5a	0.93850241	-0.09156765	0.39991576	1
Limk1	0.96110373	-0.05723595	0.40002237	1
Ecel1	0.79543633	-0.33018163	0.40003812	1
Asl	0.93639287	-0.094814145	0.40009002	1
Nell2	1.06087632	0.085256467	0.40009744	1
Spg11	1.06058593	0.084861514	0.40012088	1
Ankrd29	1.0489577	0.068956507	0.40044286	1
Pex1	1.06201211	0.086800214	0.4004901	1

Lzts1	0.94835119	-0.076506686	0.40051212	1
Bcdin3d	0.87623573	-0.190609047	0.40064967	1
Fyco1	0.94193626	-0.086298654	0.40070905	1
Fli1	1.10467162	0.14361757	0.40079887	1
Agfg2	0.9347211	-0.097392128	0.40081012	1
Arid4b	1.05218799	0.073392492	0.4008251	1
Kif1b	0.96059732	-0.057996313	0.40084773	1
Grpel2	1.06997089	0.097571541	0.40085245	1
Tgs1	1.07228581	0.100689497	0.40100798	1
Lysmd1	1.07984772	0.110827884	0.40103266	1
Alyref	0.93487294	-0.097157801	0.40106969	1
Atg14	1.07639812	0.10621178	0.40108045	1
Grid1	0.90993131	-0.136170451	0.40111006	1
Grem1	1.13359047	0.180899536	0.40115744	1
Rev1	1.06368051	0.089064888	0.4013769	1
Msln	0.86291151	-0.212715476	0.40152703	1
Naprt	1.10726057	0.146994774	0.40153376	1
Dhx35	1.07690304	0.106888357	0.40158176	1
Pvalb	1.04087709	0.057799715	0.40166762	1
Tnni3k	1.22180354	0.28901233	0.40174178	1
Slc35a4	1.04743448	0.066860005	0.40178182	1
Kdm3a	1.05345488	0.075128519	0.40219386	1
Prkag2	0.95887491	-0.060585468	0.40235519	1
Slc2a9	1.13003648	0.176369345	0.40256796	1
Mpp3	0.88769204	-0.171868842	0.40258038	1
Brsk1	0.96176733	-0.056240168	0.40265122	1
Foxc1	1.07326559	0.102007124	0.4026541	1
Mkrn2	1.05391556	0.075759288	0.4026995	1
Ifi30	0.9286654	-0.106769214	0.40275278	1
Ypel2	1.06997186	0.097572849	0.40289079	1
Kcne4	0.84572725	-0.241735633	0.40309822	1
Csnk2a1	1.04278937	0.060447781	0.40310672	1
Mgat2	1.05804923	0.081406751	0.40325733	1
Bdh2	0.86784302	-0.204493994	0.40326906	1
Rfc2	1.10207848	0.140226965	0.40328891	1
Lrrc17	1.12688939	0.172345909	0.40332873	1
Hhatl	1.05518122	0.077490797	0.40339706	1
Gdf11	0.94384812	-0.083373374	0.40339865	1
Smyd2	1.04372661	0.061743864	0.4034632	1
Slc40a1	1.07894377	0.10961968	0.40354256	1

Ddx23	0.95703459	-0.063357031	0.40359352	1
Wdr34	0.89514731	-0.159802976	0.40379933	1
Prkcq	0.93175348	-0.101979798	0.40380219	1
Rangap1	0.96113702	-0.057185975	0.40381259	1
Mrpl1	1.05981742	0.083815743	0.40386211	1
Adpgk	1.07110887	0.09910512	0.40429858	1
Clic4	0.94039954	-0.088654263	0.40442372	1
Bccip	1.06777495	0.094607611	0.4046133	1
Sema4a	1.10437753	0.143233441	0.40464194	1
Rara	0.90102054	-0.150368098	0.40472021	1
Amt	0.92490736	-0.112619221	0.40472746	1
Vav2	0.95137058	-0.071920676	0.40473179	1
Cmpk2	1.06396497	0.089450651	0.40475128	1
Zfp260	1.0537803	0.075574109	0.4047823	1
Gnl3l	0.95878956	-0.060713895	0.40484156	1
Supv3l1	1.06197835	0.086754358	0.40493495	1
Dnajb11	0.94862421	-0.076091406	0.40518337	1
Ydjc	1.12791676	0.173660603	0.40527525	1
Epha4	0.91983069	-0.120559758	0.40530781	1
Tmem54	1.07825423	0.108697374	0.40539069	1
Stk38	0.94078617	-0.088061243	0.40550362	1
Cd99l2	1.03885889	0.054999702	0.40556755	1
Bid	1.06682964	0.093329814	0.40562493	1
Slc6a2	1.25375513	0.326255609	0.40576605	1
Gabarapl1	1.04045423	0.057213508	0.40590802	1
Gpatch2l	1.08670039	0.119954233	0.40605728	1
Sfr1	1.04335007	0.061223301	0.40625817	1
Smad7	0.89370228	-0.162133797	0.40628958	1
Atxn1	1.10910194	0.149391978	0.40630217	1
Cpsf2	0.95550627	-0.065662756	0.40634109	1
Pir	1.06610769	0.09235318	0.40637452	1
Mrpl19	1.06866707	0.095812464	0.40640468	1
Camk2a	0.9344755	-0.097771258	0.40642362	1
Hsf4	0.94003	-0.089221296	0.40689629	1
Eif2a	1.05152761	0.072486729	0.40703027	1
Pbx3	0.93828076	-0.091908408	0.40713064	1
Hexa	0.956149	-0.064692639	0.40717317	1
Rcc1	0.87385843	-0.194528528	0.40718532	1
Nans	1.04554128	0.064250029	0.40728058	1
Abcb6	1.05776978	0.081025669	0.40737384	1

Mta1	0.94499145	-0.081626823	0.40742106	1
Gipc1	0.95557865	-0.065553471	0.40772277	1
Ppfia3	0.95345537	-0.068762681	0.40778815	1
Tnr	1.08283605	0.114814818	0.40786847	1
Sh3rf2	1.33028661	0.411737102	0.4078993	1
Rfng	1.06885768	0.096069762	0.40798291	1
Sp2	1.11059636	0.151334576	0.40807177	1
Nudt22	0.91152208	-0.133650485	0.40815725	1
Polr1e	1.09709327	0.133686176	0.40818293	1
Dhfr	1.10915757	0.149464331	0.40819571	1
Rev3l	0.94744102	-0.077891959	0.40824194	1
Cyb5r4	1.06090013	0.085288858	0.40825786	1
Spint2	1.07001683	0.097633494	0.40828738	1
Abi2	0.95950034	-0.059644774	0.40860994	1
Fhl2	1.10778282	0.147675074	0.40868259	1
Med24	1.04950074	0.069703185	0.40869682	1
Calcb	0.96055376	-0.058061738	0.40886564	1
Sardh	0.91244298	-0.132193692	0.40898284	1
Pdcl	0.94522413	-0.08127164	0.4089868	1
Smim11	0.88862218	-0.170357938	0.40900774	1
Yars2	1.11770954	0.160545321	0.40912454	1
Swap70	0.95309338	-0.069310529	0.40916212	1
Rrm2	1.12292134	0.167256871	0.40939369	1
Lrrc61	1.0648535	0.09065496	0.40949773	1
Fbxl21	1.20900036	0.27381467	0.40952435	1
Metrn	0.94585486	-0.080309273	0.40953553	1
Mfap2	0.89481111	-0.16034493	0.40954847	1
Plppr4	0.92863189	-0.106821274	0.40965332	1
Rgs2	1.0838269	0.116134354	0.40969041	1
Srfbp1	0.92350481	-0.114808616	0.40972626	1
Serinc3	1.03926948	0.055569783	0.40976909	1
Lpgat1	0.95360027	-0.068543456	0.40988306	1
Galnt11	1.04820246	0.067917403	0.40994741	1
Tmeff2	1.05178977	0.072846373	0.41016437	1
Vash2	1.18765478	0.248115543	0.41023039	1
Tbxa2r	1.18391484	0.243565313	0.4104185	1
Fam134a	0.95791729	-0.062027006	0.41046678	1
Dnajc7	1.03965283	0.056101845	0.41052954	1
Limd2	0.93159693	-0.102222211	0.41060727	1
Ap5m1	0.93175514	-0.101977229	0.41068044	1

N4bp2l1	1.15918422	0.213109867	0.41088775	1
Lsp1	1.07816273	0.108574941	0.41089807	1
Mbtps1	1.04130503	0.058392741	0.41090435	1
Coa7	0.89826894	-0.154780647	0.41098309	1
Kifc2	1.05578588	0.078317275	0.41119099	1
Efs	1.0942611	0.129957017	0.41125291	1
Dyrk2	0.92782701	-0.108072244	0.41138142	1
Igfbp4	1.04204493	0.05941749	0.41145965	1
Cep78	0.89996719	-0.152055682	0.41146973	1
Lrp3	0.9540897	-0.067803185	0.41172752	1
Fig4	1.05211204	0.073288347	0.41186451	1
Chmp2a	0.95290503	-0.069595664	0.41190435	1
Osbp	0.95776941	-0.062249739	0.41192856	1
Maf1	1.04838102	0.06816314	0.41198336	1
Alg13	1.10193832	0.140043478	0.41201604	1
Nos3	0.89269411	-0.16376219	0.41206371	1
Pcsk7	0.94358024	-0.083782883	0.41208159	1
Osbp2	1.05993797	0.083979836	0.41209669	1
Mtif2	1.07096759	0.098914817	0.41214599	1
Eif4a3	0.95343648	-0.068791265	0.4121783	1
Sars2	0.90496699	-0.14406293	0.41218048	1
Usb1	1.09197107	0.126934632	0.41234623	1
Hey2	0.94532436	-0.081118668	0.41243683	1
Cipc	0.9568679	-0.06360833	0.41245148	1
Asb6	1.06742359	0.094132798	0.41256718	1
Dpf3	1.22953316	0.298110644	0.41258921	1
Gfap	0.93186118	-0.101813047	0.41260346	1
Nrxn1	0.96139157	-0.056803936	0.41265787	1
Atg16l1	0.95360948	-0.068529516	0.41273637	1
Dhx57	0.96084327	-0.057626967	0.41332062	1
Ncl	1.03851338	0.054519798	0.41342481	1
Slc35a5	1.05698318	0.079952417	0.41355207	1
Bnip2	1.0690956	0.096390861	0.41358318	1
Rsbn1l	1.07183713	0.100085695	0.41363756	1
Ttc39a	0.92123374	-0.118360841	0.41367482	1
Snx18	1.04858285	0.068440856	0.41367797	1
Pard6g	0.89385869	-0.16188132	0.41373914	1
Paqr5	1.07171522	0.099921593	0.41383036	1
Krt75	1.18503838	0.244933781	0.4138874	1
Nck1	1.05602417	0.078642849	0.41392728	1

Brms1	0.95116476	-0.07223283	0.41402608	1
Insrr	1.08124155	0.112688862	0.41406946	1
Zfyve9	0.95891579	-0.060523975	0.41411233	1
Begain	1.14047203	0.189631064	0.41419946	1
Plxdc1	0.88734172	-0.172438293	0.41430143	1
Fam98c	0.91211827	-0.132707188	0.41430344	1
Rps19	1.0468613	0.066070308	0.41463777	1
Gnaq	0.95202249	-0.070932439	0.41468488	1
LOC102550394	0.848582	-0.236874018	0.41472536	1
Sp100	1.11802026	0.160946337	0.41473652	1
Ttc5	1.05498235	0.077218864	0.41496092	1
Pdap1	0.9591117	-0.060229257	0.41497087	1
Cdc73	0.91378024	-0.13008085	0.41506835	1
Osbpl11	0.94053919	-0.088440037	0.41507151	1
Pcdhga12	0.87240481	-0.196930363	0.41512128	1
Fbxl20	0.91057926	-0.135143502	0.41515721	1
Zfp512b	1.05816563	0.081565462	0.41528949	1
Vangl1	0.90317054	-0.146929671	0.41541251	1
Rad18	1.09797511	0.134845347	0.41550659	1
Plekhh2	0.90269985	-0.147681733	0.41557285	1
Aebp2	0.90227191	-0.148365817	0.41558736	1
Kifc3	0.92622212	-0.110569889	0.41560083	1
Drd2	0.93479961	-0.097270958	0.41568841	1
Dcaf15	0.92214963	-0.116927234	0.41570972	1
Plppr2	1.0619204	0.086675623	0.41571566	1
Rap1gap	0.96076552	-0.057743713	0.41583378	1
Lrrc56	1.20397281	0.267802816	0.41593075	1
Tmem100	0.9426909	-0.085143295	0.41596556	1
Tsen2	1.1079558	0.147900335	0.41597551	1
Fam3c	1.04750419	0.066956019	0.41618842	1
Iqgap1	1.04000435	0.056589563	0.41619635	1
Slc6a13	1.08031313	0.111449546	0.41647176	1
Prpf40a	1.04840627	0.068197885	0.41647185	1
Ppp2r2d	1.05480242	0.076972789	0.41652049	1
Hnrnph3	1.05773909	0.080983807	0.41655876	1
Tbc1d2b	0.9492089	-0.075202464	0.41656163	1
Wdpcp	1.10499566	0.144040703	0.41656825	1
Cnnm2	0.9272537	-0.108963981	0.41658867	1
Rhag	0.82625162	-0.275346894	0.41658994	1
Dact3	0.93466639	-0.097476573	0.4166328	1

Arhgap22	0.93516838	-0.096701943	0.41663737	1
Zfp418	0.89547767	-0.159270645	0.41672549	1
Gsta1	0.9595287	-0.059602129	0.41681159	1
Chst12	1.06881018	0.09600566	0.41682052	1
Gen1	0.83901706	-0.253227949	0.41689699	1
Ttyh1	0.96178134	-0.056219158	0.41720426	1
Fscn1	0.93633733	-0.094899718	0.41736286	1
Nav3	0.91766042	-0.123967708	0.41746145	1
Trpm8	0.95726998	-0.063002224	0.41747806	1
Zbtb48	0.89155871	-0.165598297	0.41760563	1
Adrm1	0.95276363	-0.069809748	0.41772034	1
Grin2d	0.78166698	-0.355373993	0.41774988	1
Fntb	1.04860596	0.068472646	0.41776548	1
Tusc3	0.95859466	-0.061007198	0.41784915	1
Aak1	0.96365129	-0.053416908	0.41792046	1
Tmem68	1.05565456	0.078137827	0.41799631	1
Slc35b2	0.93894908	-0.090881172	0.41800379	1
Pik3c2g	1.14345656	0.193401564	0.41802324	1
Ccdc97	1.06014334	0.084259339	0.41821531	1
Cd300a	0.82578796	-0.276156706	0.4182896	1
Agpat2	1.19576038	0.257928314	0.41845705	1
Bcl6b	1.176137	0.234056124	0.4184764	1
Hdac8	1.09736986	0.134049854	0.41848316	1
Fibp	1.05654054	0.079348128	0.41851275	1
Pxmp4	1.09838066	0.135378123	0.41854567	1
Zfp691	1.10086301	0.138634959	0.4186387	1
Lamc2	1.13109811	0.177724077	0.41871558	1
Ptpro	0.93240224	-0.100975632	0.4188255	1
Nbr1	1.0378735	0.053630611	0.4189369	1
Gpt	1.12267996	0.166946719	0.41899503	1
Plcg1	0.96128263	-0.056967423	0.41901477	1
Hmg20a	1.04623342	0.065204765	0.41906476	1
Serinc1	1.03996014	0.056528237	0.41913624	1
Ehbp1l1	1.0532239	0.074812168	0.41926105	1
Qsox1	0.95100446	-0.072475984	0.41946118	1
Arid5a	1.13989445	0.188900242	0.41952815	1
Ank3	0.96355911	-0.05355492	0.41958351	1
Tle3	0.94459651	-0.082229896	0.41961437	1
Dnajb9	0.95787802	-0.062086151	0.41982586	1
Smagp	0.83159244	-0.266051444	0.41991367	1

Irf2bp1	0.93843139	-0.091676821	0.41993843	1
Stx12	1.03771849	0.053415123	0.41998575	1
Slc6a17	0.9634831	-0.053668736	0.42009325	1
Rps11	1.03805833	0.053887509	0.42009402	1
Npepo	1.11718631	0.159869796	0.42035058	1
Trim21	0.89632443	-0.15790708	0.42035807	1
Shc1	0.96203847	-0.055833514	0.42040652	1
Capn1	0.96287139	-0.054584979	0.42041992	1
Abcd4	1.12147621	0.165399021	0.42060552	1
Acot11	1.13084649	0.177403094	0.42064192	1
Snx11	1.05317505	0.074745256	0.42065482	1
Lrrc24	0.93805158	-0.092260841	0.42077736	1
Dok2	0.84494441	-0.243071674	0.42095273	1
Mfsd2a	0.94032458	-0.088769258	0.42120936	1
Appl2	0.94429797	-0.082685922	0.42143313	1
Slc25a45	1.07822005	0.108651643	0.42156985	1
Tac1	0.96385215	-0.053116233	0.42166613	1
Rab12	0.89266424	-0.163810456	0.42179355	1
Rgs19	0.90894121	-0.137741115	0.42184877	1
Psmb6	1.04899541	0.069008363	0.42186982	1
Notch4	1.09634399	0.132700532	0.42194316	1
Sox6	0.86963511	-0.201517905	0.42195653	1
Rnh1	1.04240783	0.059919821	0.42215892	1
Emc4	1.04163879	0.05885508	0.42217519	1
Hs2st1	0.95822927	-0.061557208	0.42217572	1
Celf4	0.9488439	-0.075757337	0.42229184	1
Rb1	0.95448724	-0.067202191	0.42236994	1
Mvk	0.94428361	-0.082707859	0.42238619	1
Hmgb2	1.09279747	0.12802605	0.42243554	1
Magee1	0.96265384	-0.054910987	0.4224858	1
Zfx	1.08420541	0.116638109	0.42286727	1
Eif4a1	1.03740515	0.052979436	0.42287825	1
Omd	1.05337942	0.075025174	0.42292217	1
Nr1h3	1.10048239	0.138136064	0.42300787	1
Taok2	0.95687647	-0.063595411	0.42303969	1
Klhl23	1.08688555	0.120200033	0.42304067	1
Cdc25a	0.92480966	-0.112771626	0.42318484	1
Psmg2	1.06566528	0.09175437	0.42326657	1
Zfp280b	0.90344575	-0.146490118	0.42334851	1
Gabrb2	0.87672222	-0.189808276	0.42336272	1

Pdzrn4	1.20918408	0.274033892	0.42336955	1
RGD1566239	1.07366766	0.102547495	0.42372896	1
Psmc3	1.0377273	0.053427368	0.42384698	1
Nploc4	0.96229219	-0.055453079	0.42393877	1
Phc3	0.93959457	-0.089889722	0.42395794	1
Dcaf17	0.89358376	-0.162325123	0.42396449	1
Alox15	1.08921386	0.123287246	0.42396534	1
Slc39a7	1.04435381	0.06261055	0.42398139	1
Per3	0.94640375	-0.079472311	0.42435015	1
Golgb1	0.96406219	-0.052801877	0.42438279	1
Vps4a	0.96065027	-0.057916791	0.42439497	1
Yipf6	1.04414502	0.062322097	0.42448045	1
Ltbp4	0.95258773	-0.070076132	0.42451846	1
Ostf1	1.03798581	0.053786725	0.4246101	1
Apln	0.83927687	-0.252781276	0.42464825	1
Raver2	0.88974628	-0.168534106	0.42494724	1
Birc2	1.05565353	0.078136415	0.42498029	1
Mcur1	0.90549059	-0.143228445	0.42520536	1
Prss23	1.06452371	0.090208082	0.42521316	1
Ggnbp1	1.27127192	0.346272653	0.42532247	1
Scamp2	1.06569326	0.09179224	0.42532902	1
Zfyve1	0.95248684	-0.070228931	0.42536011	1
Tars2	0.94852111	-0.076248215	0.4255252	1
St6gal1	1.04586768	0.064700337	0.4259335	1
Slc12a3	0.89445998	-0.160911154	0.42597462	1
Dmrtc1a	1.11145559	0.152450301	0.42604828	1
Cdh13	1.03892784	0.055095458	0.42622834	1
Pik3ap1	0.85935567	-0.218672738	0.42627697	1
Ndufa8	1.04484712	0.06329186	0.42636979	1
Zfp426	1.10312509	0.141596399	0.4263751	1
Ypel3	1.0379255	0.053702889	0.42641239	1
RGD1564541	1.06611029	0.092356699	0.42643844	1
Mrps25	0.94105035	-0.087656178	0.42646347	1
Pou6f1	0.89943925	-0.152902257	0.42653837	1
Pip4k2b	0.95088355	-0.072659424	0.42655539	1
Gtf2i	0.96393967	-0.05298524	0.42662772	1
Flt4	0.84452577	-0.243786641	0.42701353	1
Sec16a	0.95929584	-0.05995229	0.42705011	1
Tyms	1.1003102	0.13791031	0.4271064	1
Slc25a37	1.10896524	0.149214142	0.42716055	1

Socs4		1.10211821	0.140278978	0.42720051	1
Uck1		0.9440513	-0.083062843	0.42724575	1
Poglut1		1.04776727	0.067318302	0.42731616	1
Dcun1d5		0.9504356	-0.073339215	0.42747698	1
Atp1b3		0.96310519	-0.054234717	0.4274897	1
Fbxo9		0.95660447	-0.064005557	0.42755241	1
Abcb10		0.93333521	-0.099532769	0.42758579	1
Mettl23		0.88576391	-0.175005873	0.42770426	1
Gmpr		0.9521185	-0.070786952	0.42797816	1
Ythdf3		1.04213442	0.059541379	0.42814208	1
Nhej1		0.85659965	-0.223307003	0.42819313	1
Ndufb4		1.05090216	0.071628365	0.42822146	1
Btbd3		0.95797074	-0.06194651	0.4284204	1
Cwf19l1		1.07189936	0.100169457	0.4285356	1
Soat1		1.06470212	0.090449852	0.42858478	1
Vat1		0.96368672	-0.053363877	0.42859651	1
Krt26		0.8271679	-0.273747901	0.42866999	1
Tcea1		1.04324417	0.061076864	0.42876062	1
Adnp		0.96153179	-0.056593538	0.42887449	1
Pde4a		1.14253325	0.192236156	0.42896558	1
Dusp3		0.96438656	-0.052316544	0.42900604	1
Hoxd10		0.51004017	-0.971317208	0.42921299	1
Mrpl10		1.05370144	0.075466142	0.42931624	1
	2-Mar	1.03883028	0.054959968	0.42951101	1
Opcml		0.93259085	-0.100683817	0.42951214	1
Akap8		1.04417219	0.062359645	0.42951322	1
Exosc3		1.16959714	0.226011691	0.42957613	1
Ahdc1		1.0639146	0.089382347	0.42974329	1
Fibin		1.08527325	0.118058326	0.42980926	1
Dip2c		0.95832974	-0.061405957	0.42984633	1
Alkbh6		1.09344817	0.128884833	0.43003836	1
Zfp414		0.9261499	-0.110682372	0.4302227	1
Tbc1d10b		0.95790325	-0.062048153	0.4303313	1
Cadm4		0.95613761	-0.064709818	0.43041246	1
Pigm		1.05194674	0.073061657	0.43061648	1
Nrd1		0.96268929	-0.054857853	0.43096085	1
Rrm1		1.05502081	0.077271461	0.43100135	1
Ftsj3		0.94574598	-0.080475356	0.43109634	1
Stxbp5l		1.06723775	0.093881604	0.43118037	1
Cenpa		0.73109361	-0.451871945	0.43137772	1

Cdc25b	0.95821616	-0.061576944	0.43151445	1
Gdap1l1	0.95070447	-0.072931147	0.43163313	1
Chst9	1.13753807	0.185914827	0.43207968	1
Ston1	1.06488016	0.090691074	0.43216423	1
Snf8	0.95005406	-0.073918491	0.43221051	1
Fam110b	1.06340867	0.088696132	0.4322426	1
Gramd1c	0.80599636	-0.311154769	0.43227108	1
Slitrk5	1.05816231	0.081560942	0.43230536	1
Fuk	1.08507102	0.117789471	0.43240924	1
Slc12a4	0.95439662	-0.067339164	0.43271959	1
Rab34	0.9434557	-0.08397331	0.43273731	1
RGD1561149	0.9561501	-0.064690974	0.43275291	1
Psd	0.8895452	-0.168860178	0.43276649	1
Acot7	0.96462427	-0.051960981	0.43287164	1
Idnk	0.89972421	-0.152445259	0.43294273	1
Klhl11	0.95459546	-0.067038622	0.43302014	1
LOC317456	0.91434953	-0.129182315	0.4331111	1
Trmt11	1.13026125	0.176656282	0.43323843	1
Bspry	1.20270897	0.266287586	0.43346598	1
Aebp1	1.03992154	0.05647468	0.43381401	1
LOC361990	1.03864377	0.054700935	0.4338535	1
Rgs16	0.81994719	-0.286397093	0.43386438	1
Nefl	1.0404957	0.057270999	0.43402324	1
Rundc3b	0.94652025	-0.07929472	0.43404918	1
Aldh1l2	0.85926951	-0.218817391	0.43452856	1
Ccng1	0.96472298	-0.051813365	0.43454632	1
Syde1	1.07181463	0.100055409	0.43466501	1
Lipa	1.03656546	0.051811233	0.43467502	1
Gbf1	0.96412766	-0.052703906	0.43474872	1
Mfap1a	1.05175267	0.072795485	0.43475685	1
Pam	0.96475165	-0.051770489	0.43476484	1
Tgif2	0.9025772	-0.147877764	0.43478741	1
Vegfc	0.86232101	-0.213703063	0.43499431	1
l7Rn6	1.05947185	0.083345251	0.43506489	1
Nsdhl	0.95995647	-0.05895911	0.43509845	1
Anapc11	1.0558045	0.078342718	0.43517242	1
Ints7	1.06580398	0.091942128	0.43525239	1
Slc26a10	1.18602309	0.246132097	0.43531424	1
Bri3bp	1.17631841	0.23427863	0.43541383	1
Supt7l	1.10548926	0.144685006	0.43543721	1

Myl6l		1.03670131	0.052000297	0.43544563	1
Nipsnap3b		1.05582336	0.078368496	0.43548536	1
Uqcrc1		1.03691749	0.052301095	0.43555254	1
Smim17		0.90779452	-0.13956231	0.43557821	1
Lcmt1		0.94843956	-0.07637225	0.43558302	1
Arhgap21		0.96360211	-0.05349054	0.43558965	1
Mob3b		0.93482043	-0.097238834	0.43560021	1
Psme4		1.0365458	0.051783868	0.43568717	1
Cdt1		0.82846045	-0.271495264	0.43570476	1
Chpf2		0.95606943	-0.064812703	0.4357169	1
Coq10a		1.06892871	0.096165634	0.43582615	1
Skap2		1.06896934	0.096220473	0.43583585	1
Trps1		0.89078291	-0.166854212	0.43610841	1
Clcn1		1.16167383	0.216205051	0.43616409	1
Mrgprx3		0.94226296	-0.085798365	0.43630119	1
Coq10b		0.94681498	-0.078845563	0.43633417	1
Thnsl2		0.92170098	-0.117629318	0.43642577	1
Kdm8		1.07604139	0.10573357	0.43647982	1
Pde4dip		1.03719138	0.052682117	0.43656526	1
Pigx		1.06859887	0.095720402	0.43662536	1
Pik3r6		1.18793062	0.248450583	0.43663991	1
LOC500124		0.83527272	-0.259680775	0.43664481	1
Tmem178b		0.9628316	-0.054644602	0.43664968	1
Dnajb6		1.04521396	0.063798298	0.43667871	1
LOC691141		1.22471677	0.292448151	0.43669591	1
Atl1		1.03655917	0.051802471	0.43672791	1
Coq5		0.94549876	-0.080852533	0.43679518	1
Wdr4		0.90975591	-0.136448582	0.43729417	1
Tmem168		1.05117644	0.07200484	0.43736834	1
Myo18a		0.96473052	-0.051802081	0.43758615	1
Abca13		0.78728337	-0.345045086	0.43770307	1
Chd2		1.04895087	0.068947113	0.43776876	1
Mapre2		0.96477553	-0.051734778	0.43783055	1
	7-Mar	1.04919986	0.069289516	0.4378331	1
Ppp1r1c		0.96489942	-0.051549526	0.43784004	1
Man1a2		0.95912934	-0.06020272	0.43786933	1
RGD1304624		0.86471251	-0.209707541	0.43795481	1
Prr13		1.0427389	0.060377959	0.43819647	1
Entpd3		1.03980402	0.056311631	0.43827767	1
Ppm1e		1.05911386	0.082857695	0.4383453	1

Nr2c2	0.9457069	-0.080534977	0.43835073	1
Plk4	0.91666014	-0.125541152	0.43852727	1
Trak2	0.964892	-0.051560621	0.43864162	1
Eogt	1.09295703	0.128236686	0.43869406	1
Rfk	1.04126602	0.058338685	0.43872109	1
Pla2g16	0.95791382	-0.06203223	0.43882543	1
Iqgap3	0.8883926	-0.170730726	0.43897473	1
Rapgef6	0.95594219	-0.065004727	0.43900401	1
RGD1561778	1.23511808	0.304648968	0.43947685	1
Zfp955a	1.07602067	0.10570579	0.4394934	1
Dnmt1	0.94429382	-0.082692262	0.43949506	1
Ccnh	1.06771463	0.094526102	0.43969287	1
Mapkapk2	1.05486976	0.077064884	0.43986653	1
Comtd1	0.94149788	-0.086970256	0.44000165	1
Clns1a	1.05063258	0.071258221	0.44008179	1
Tmed7	1.03796571	0.053758782	0.4401201	1
Nckap1l	1.07570251	0.105279148	0.44029869	1
Ipp	1.07616947	0.105905281	0.44035727	1
Dhx40	1.0446581	0.063030845	0.4404932	1
Man1b1	0.96399949	-0.052895718	0.44059426	1
Polr2e	1.04852752	0.068364726	0.44063414	1
Aftph	1.03938327	0.05572774	0.44069047	1
Ifi27	1.03600065	0.051024912	0.44072898	1
Adap2	1.16052112	0.214772776	0.440793	1
Tfpi	1.05109053	0.071886932	0.44085676	1
Cpne5	0.86760798	-0.20488477	0.44095423	1
Trim3	0.95605099	-0.064840529	0.44096002	1
Arfgap1	1.05955071	0.083452636	0.44106734	1
Ddx3x	1.03675311	0.052072374	0.44112674	1
Frmd3	0.94843628	-0.076377247	0.44117638	1
Mcm3	0.88125389	-0.18237037	0.44126831	1
Pcm1	0.96468516	-0.051869925	0.44153396	1
RT1-CE4	1.0948072	0.130676829	0.44157489	1
Inpp4a	0.96310676	-0.054232365	0.44159267	1
Fubp1	1.04606101	0.064966993	0.44179328	1
Pcdhb3	0.90268938	-0.147698461	0.44215687	1
Cyb5r3	1.03600177	0.051026474	0.44225484	1
Retsat	0.94996459	-0.074054358	0.44229225	1
Sod2	1.03590558	0.050892511	0.44237852	1
RT1-CE7	0.90648341	-0.141647476	0.44237992	1

Rab3il1	1.09881463	0.135948029	0.44238141	1
Prelid3a	0.90244989	-0.148081276	0.44242099	1
Slc20a1	1.03704267	0.052475253	0.44244732	1
Ctsb	1.03835372	0.054297987	0.44269122	1
Tspan2	0.96249683	-0.055146304	0.44284958	1
Zeb2	0.95978926	-0.05921043	0.44293588	1
Msi1	1.15128005	0.203238817	0.44294218	1
Elovl4	1.04605683	0.064961234	0.44302805	1
Abr	0.96554147	-0.050589869	0.44308073	1
Mccc2	1.06590821	0.0920832	0.44308335	1
Ppp1r2	0.96469016	-0.051862438	0.44327231	1
Def8	0.94875146	-0.075897887	0.44328165	1
Rpl21	1.03740254	0.052975802	0.44329868	1
Dysf	0.96392156	-0.053012339	0.44334476	1
Egfr	1.08393043	0.116272163	0.44337792	1
Cstf3	1.07289868	0.101513845	0.44395776	1
H2afj	0.91149798	-0.133688631	0.44395899	1
Ap2a2	0.96550964	-0.050637431	0.44403192	1
Ccnt2	1.04032417	0.057033149	0.44414896	1
Clip2	0.9649755	-0.051435783	0.44417845	1
Heatr6	1.07889705	0.109557212	0.44434598	1
Pde4b	1.06442516	0.09007452	0.44434906	1
Nos1	1.13663432	0.184768179	0.44463271	1
Bcl2l2	0.96507186	-0.051291723	0.44464203	1
Mtus1	1.03686932	0.052234083	0.44464452	1
Zfp524	0.89270068	-0.163751572	0.44475559	1
Pcdha6	1.07474661	0.103996562	0.44492094	1
Stra6	1.08362943	0.115871486	0.44493663	1
Pcgf3	0.93110268	-0.102987815	0.44518548	1
Ccdc53	1.05751441	0.080677322	0.44523046	1
Med6	1.06872454	0.095890047	0.44544776	1
Jam2	1.03821805	0.054109473	0.44578031	1
Krcc1	0.95284178	-0.069691428	0.44579108	1
Zfp787	0.9095353	-0.136798458	0.44584405	1
Evpl	1.1259655	0.171162623	0.44608747	1
Sppl2b	0.95116052	-0.072239257	0.44614805	1
Rcan2	0.96543567	-0.050747968	0.44618318	1
Rbsn	1.04606761	0.064976102	0.44618932	1
Nasp	1.07347418	0.102287489	0.44629017	1
Wdtc1	0.96125856	-0.057003554	0.44635229	1

Cdkn2c		1.10871158	0.148884115	0.44635731	1
Cad		0.9487116	-0.075958511	0.44637775	1
Pla2g4a		0.91425689	-0.1293285	0.44655737	1
Crabp2		1.05778296	0.081043645	0.44656406	1
F8		0.94207679	-0.086083429	0.44672743	1
Plp1		0.96449917	-0.052148103	0.44673669	1
Copz1		0.96269604	-0.054847742	0.44690238	1
Angel1		0.95329279	-0.069008709	0.44703582	1
Ptn		0.96493042	-0.051503185	0.44708803	1
Lix1		1.10125906	0.13915389	0.44714035	1
Adcy3		0.95115245	-0.072251505	0.44722753	1
Dclre1b		0.92856038	-0.106932365	0.4472802	1
Ddx52		1.07483805	0.104119304	0.44734005	1
Arpc5		0.96531776	-0.050924169	0.44737285	1
Tfb1m		0.94967276	-0.074497618	0.4473899	1
Mrpl30		1.0576684	0.080887389	0.44742221	1
Dnal1		1.11517871	0.157274921	0.44745853	1
Cpeb1		0.96445179	-0.052218965	0.44750703	1
Abcc4		0.94018195	-0.088988106	0.44781582	1
S100a10		1.03657534	0.051824971	0.44789908	1
Tmcc3		0.94935684	-0.074977637	0.44803738	1
Rnaseh2b		1.07003058	0.097652023	0.44811852	1
Lamtor4		1.0476217	0.067117842	0.44813615	1
Rph3al		0.85432018	-0.227151236	0.44815619	1
Amd1		1.03669129	0.051986343	0.44817478	1
Gnl2		1.04508758	0.063623847	0.44830709	1
Rpp14		1.05805268	0.081411464	0.44848367	1
Fam58b		0.91537364	-0.12756734	0.44852515	1
Cadps		0.96591992	-0.050024503	0.44884507	1
Amigo2		0.93930175	-0.0903394	0.44885858	1
Fam103a1		1.05316374	0.074729749	0.44893839	1
Mogat2		0.90615819	-0.142165175	0.44902233	1
Eno1		0.96560547	-0.050494242	0.44915438	1
Dnajc18		1.05173962	0.072777581	0.44930592	1
	9-Mar	1.0922288	0.1272751	0.44932909	1
Zbtb24		1.0891186	0.123161062	0.44933225	1
Kctd9		1.03856707	0.054594389	0.44947935	1
Mcf2l		0.9615008	-0.05664003	0.44968322	1
Tubgcp6		0.94267401	-0.085169147	0.44973306	1
Sash3		0.88389342	-0.178055681	0.44979074	1

Kcnip2	0.96223517	-0.055538564	0.44986458	1
Asf1a	1.06543833	0.091447094	0.44995576	1
Rcan3	0.89122222	-0.166142885	0.45018757	1
RGD1560289	1.15489619	0.207763181	0.45020319	1
Rps6ka2	0.96447805	-0.052179693	0.45021452	1
Zfp282	0.93494329	-0.097049232	0.45025486	1
Paox	1.05654566	0.079355117	0.45028677	1
Asah2	1.04801154	0.067654604	0.45038508	1
Mrpl16	1.05953315	0.083428721	0.45055876	1
Fdxr	1.11462461	0.156557919	0.45056112	1
Slc9a3	0.91328248	-0.13086693	0.45067199	1
Mmadhc	1.05007678	0.070494813	0.4509915	1
Drc1	1.12946432	0.175638697	0.45115236	1
Mertk	0.94107872	-0.087612686	0.45117186	1
Zfp317	1.06816118	0.095129363	0.4512196	1
Nfyc	1.05578022	0.078309548	0.45139882	1
Pmm2	1.04486168	0.063311968	0.45146657	1
Apobec2	0.72671372	-0.460540949	0.45160082	1
Scn2b	0.91490474	-0.128306553	0.451601	1
Gal3st3	0.86087767	-0.216119852	0.4516089	1
Itfg1	1.03552869	0.050367524	0.45184881	1
Slc25a23	0.93613163	-0.095216693	0.45196825	1
Tmem86b	0.83061869	-0.267741759	0.45212471	1
Hspb6	1.07565111	0.105210218	0.45218272	1
Anxa4	1.03648368	0.051697396	0.45222701	1
Il33	0.96470785	-0.051835995	0.45230152	1
Daglb	0.94087144	-0.087930482	0.45243572	1
Hoga1	0.85336534	-0.228764583	0.45252492	1
MGC94199	1.05911371	0.082857487	0.45260054	1
Pgm3	1.06468172	0.090422213	0.45260679	1
Nfkbil1	0.92593807	-0.111012391	0.45274663	1
Cmtm6	0.9044611	-0.144869644	0.45276028	1
Arf6	0.95149298	-0.071735082	0.45276381	1
Ctsz	1.04633747	0.065348237	0.45277855	1
Sp140	0.92266426	-0.116122314	0.45293726	1
Twist1	1.10465496	0.143595809	0.45309937	1
LRRTM1	0.918148	-0.123201373	0.45330186	1
Copg1	1.03515406	0.049845492	0.45332274	1
Zfp532	1.04928338	0.069404357	0.4533332	1
Kalrn	0.95001724	-0.073974394	0.45333344	1

Sidt2		0.96190577	-0.056032529	0.45342356	1
Rexo1		0.94734225	-0.078042366	0.45345112	1
Entpd2		0.95673465	-0.06380925	0.45345887	1
Snapc1		0.93159866	-0.102219525	0.45353421	1
	6-Mar	0.96625609	-0.049522498	0.4535604	1
Nsl1		1.18577204	0.245826682	0.45361195	1
Gnai3		0.96487712	-0.05158287	0.45365073	1
Gpat4		0.96176752	-0.056239891	0.45373467	1
Mzf1		0.91168446	-0.133393516	0.45399678	1
Cbr3		0.89654782	-0.157547559	0.45401442	1
Cplx2		0.94909828	-0.075370614	0.45402357	1
Pcdhb12		0.8515355	-0.231861412	0.45407144	1
Cd101		1.22426615	0.291917226	0.45414382	1
Runx1t1		0.92763256	-0.108374642	0.45421473	1
Tmx1		0.96351991	-0.05361361	0.45432836	1
Zmat2		1.04061341	0.057434207	0.45445078	1
Fbn2		0.87318796	-0.195635858	0.45447783	1
Cacna1g		0.87717386	-0.189065273	0.45453153	1
Tnfaip8		0.91908573	-0.121728651	0.45456556	1
Actr5		1.11736937	0.160106173	0.45470788	1
Serpinb6		1.03539617	0.05018288	0.4548033	1
Synj2bp		0.96560621	-0.050493144	0.45525399	1
Pelp1		0.94897635	-0.075555955	0.45529409	1
Rufy3		1.03508711	0.04975219	0.45529533	1
Cox5a		0.96295868	-0.054454205	0.45533135	1
Pole		0.90063889	-0.150979327	0.45542692	1
Txndc17		1.05645064	0.079225365	0.45543828	1
Higd1a		0.95592432	-0.065031688	0.45547201	1
Tmem120a		0.94768803	-0.077515879	0.4555124	1
Ptprt		0.92905034	-0.106171328	0.45555911	1
Kif5c		1.03531458	0.0500692	0.45567019	1
Abca4		1.17249509	0.229581884	0.45571783	1
Atp11a		1.03612832	0.051202692	0.4557364	1
Snx6		1.0359841	0.051001862	0.45575252	1
Pnrc1		1.0523477	0.073611459	0.45585509	1
Fam122b		0.9204601	-0.119572913	0.45590685	1
Fam133b		1.05681115	0.079717588	0.45600775	1
Map1b		0.96285214	-0.054613832	0.4561776	1
Esyt1		0.96645061	-0.049232094	0.45638198	1
Adprm		0.94300539	-0.084662085	0.4563966	1

Cops4	1.03690579	0.052284817	0.456409	1
Smpdl3a	1.05740729	0.080531182	0.45644495	1
Sar1b	1.04818545	0.067893992	0.45654104	1
Sdf2l1	1.09587759	0.13208666	0.45658107	1
Gadd45g	0.93285804	-0.100270541	0.45707323	1
Wdr7	0.96489741	-0.05155254	0.45707856	1
Dda1	0.95953839	-0.059587565	0.45708129	1
Rasgrp2	0.89902435	-0.153567911	0.45722257	1
LOC689064	0.96639692	-0.049312234	0.45727753	1
Pogz	0.9511611	-0.072238383	0.45728264	1
Rbm34	0.93373467	-0.098915436	0.45749532	1
Denr	1.0500419	0.070446899	0.45750754	1
Myo16	0.86343447	-0.211841408	0.45755937	1
Reep6	1.08850656	0.122350109	0.45758131	1
Aldh9a1	1.04918516	0.06926931	0.45760451	1
Pbk	0.78340993	-0.352160683	0.45762632	1
Degs1	1.03516732	0.049863982	0.45765611	1
Krt25	1.17670489	0.234752545	0.45768341	1
Blmh	1.04557861	0.064301536	0.45775732	1
Tor4a	1.12183172	0.165856283	0.45776554	1
Mcm4	1.06388314	0.089339693	0.45794726	1
Spry3	1.13074893	0.177278629	0.45801081	1
Fam175b	1.04378677	0.061827027	0.45804982	1
Plcb1	0.94548503	-0.080873476	0.45815095	1
Mrgprd	0.93787297	-0.092535571	0.45825555	1
Krt28	1.10303807	0.141482587	0.45830034	1
Pdgfra	1.03980858	0.056317961	0.45842927	1
Nt5c3b	1.0692568	0.096608377	0.4584953	1
Amph	1.03615211	0.051235817	0.45850102	1
Znhit2	0.93702525	-0.093840166	0.45885715	1
Rbpms	1.15240539	0.204648319	0.4590317	1
Zbtb17	1.06915349	0.096468986	0.45919677	1
Zswim3	1.08048833	0.11168349	0.45924181	1
Kdm2b	0.92684709	-0.109596752	0.45929972	1
Dync2li1	1.07212317	0.100470662	0.45930516	1
Dram1	1.11934055	0.162649032	0.45934426	1
Tmem11	1.05795235	0.081274645	0.45945159	1
Fbxw2	0.96091821	-0.057514449	0.4595066	1
Selenbp1	1.07653617	0.106396788	0.45954362	1
Sqstm1	0.96606504	-0.049807774	0.45959814	1

Dgcr14	0.9368055	-0.094178547	0.45967004	1
Htt	0.96512653	-0.051210002	0.45971285	1
Slk	1.04322619	0.061052	0.45977479	1
Gstp1	1.04034376	0.057060317	0.45979748	1
Dlgap4	0.94910308	-0.075363311	0.45980241	1
Tipin	0.90690457	-0.140977351	0.45984915	1
Pick1	0.93763486	-0.092901885	0.45995147	1
Pla2r1	1.17254468	0.229642894	0.45998992	1
Jakmip1	0.95934318	-0.059881096	0.46000966	1
Elavl3	0.95064676	-0.073018728	0.46002098	1
Tpk1	0.92088387	-0.118908854	0.46012791	1
Gapdh	1.03608779	0.051146252	0.4602083	1
Cldn5	1.04966592	0.069930226	0.46024326	1
Capzb	0.96679056	-0.048724709	0.46061949	1
Agtrap	0.92723227	-0.108997311	0.46077705	1
Atat1	0.92407765	-0.113914009	0.46104771	1
Ankmy2	1.04134663	0.058450377	0.46146342	1
3-Mar	1.1319428	0.178801063	0.46169553	1
Vps45	0.95604707	-0.064846441	0.46170448	1
Cryzl1	1.05613942	0.078800298	0.46179736	1
Itpkb	0.95534798	-0.065901775	0.46195944	1
Vps72	1.06626414	0.092564877	0.4620728	1
Tnfsf12	1.06073491	0.085064162	0.46209528	1
Tshz3	1.05652939	0.079332899	0.46230316	1
Ddb1	0.96687933	-0.048592242	0.4623695	1
Fbln1	1.04799501	0.067631843	0.46237704	1
Sidt1	0.84815228	-0.237604781	0.46241598	1
Foxred2	0.85429156	-0.22719956	0.46247256	1
RGD1559909	1.06338591	0.088665262	0.46251229	1
Hmgn5b	0.85949851	-0.218432964	0.46269088	1
Prune	1.051091	0.071887573	0.46270432	1
Uvrag	0.93958762	-0.089900394	0.46271207	1
Acly	0.96633249	-0.049408421	0.46274678	1
Zc3h11a	0.96344674	-0.053723175	0.46290709	1
Epb41l1	0.96651876	-0.049130358	0.46315303	1
Pum3	1.05850584	0.08202923	0.46326704	1
Tollip	0.96535696	-0.050865586	0.46330005	1
Cuta	1.0508525	0.071560181	0.46361609	1
Psmd12	1.03449908	0.048932363	0.46362845	1
Tnfrsf21	0.9668125	-0.048691974	0.46369369	1

Acot2	1.08706688	0.120440704	0.46372133	1
Tmem192	1.06001798	0.084088733	0.46382874	1
Efhd2	0.94682142	-0.078835757	0.46387089	1
Pwp1	1.07033258	0.098059151	0.46388388	1
Prkrip1	0.93786071	-0.092554431	0.46390734	1
Cmtr2	0.92155838	-0.117852539	0.46394464	1
Dync1i1	1.0343194	0.048681766	0.46399938	1
Cntnap5c	0.92128443	-0.11828146	0.46414692	1
Zfp354a	1.13575352	0.18364978	0.46417171	1
Ift88	1.05695729	0.079917082	0.4642129	1
Trmt44	0.91317099	-0.131043067	0.46423355	1
Zfpm1	0.93505465	-0.096877408	0.46433923	1
Clec5a	1.20149117	0.264826047	0.46436336	1
Adcy4	0.90154382	-0.149530479	0.46439656	1
Dctn3	1.04098262	0.057945988	0.46441348	1
Mtmr10	1.05317064	0.07473921	0.4644159	1
Zfand2b	0.94438068	-0.082559565	0.46452806	1
Tcea3	0.85340183	-0.228702885	0.46457698	1
Dctn5	1.03797977	0.053778328	0.46460969	1
Parp3	1.04955442	0.069776971	0.46473449	1
Lonp1	0.96264665	-0.05492175	0.46474183	1
Ogdhl	0.92955198	-0.10539255	0.46482615	1
Them6	1.03482528	0.049387208	0.46483742	1
Arl4d	0.92819957	-0.10749306	0.46493891	1
P3h1	0.93546735	-0.096240793	0.4650775	1
Zfp516	0.93607863	-0.095298368	0.46517379	1
Ptprr	1.04238566	0.059889147	0.46525891	1
Snrnp40	0.94127196	-0.08731648	0.4653054	1
Cggbp1	0.9594534	-0.059715364	0.46551939	1
Tmem126b	1.0619305	0.086689355	0.46563722	1
Wdr44	0.95906099	-0.060305524	0.46588646	1
Glb1l	1.07263295	0.101156475	0.46598619	1
Mr1	1.14844742	0.199684807	0.46606545	1
Kctd21	1.13227985	0.179230573	0.46610121	1
Nr1d1	1.04604899	0.064950415	0.46664488	1
Hpcal1	0.96065501	-0.057909675	0.46665367	1
Hacl1	1.06567613	0.091769053	0.46698427	1
Tmco3	1.04274146	0.060381501	0.46706421	1
Hcn2	0.96345011	-0.053718128	0.46719167	1
Zfp709	0.87397597	-0.194334481	0.46726186	1

Myadml2	0.86504739	-0.20914893	0.46744071	1
Mrpl35	1.05229814	0.073543509	0.46744095	1
Smyd4	1.08304813	0.115097362	0.46744412	1
Arpc3	1.03534649	0.050113666	0.4675823	1
Prkaca	0.96609862	-0.049757626	0.46768517	1
Psmc1	1.03382063	0.047985903	0.46772523	1
Gcfc2	0.90565798	-0.142961771	0.46785518	1
Vps33b	1.05804631	0.081402779	0.46793537	1
Lmcd1	0.88730158	-0.172503563	0.46803731	1
Sncb	0.96543411	-0.050750295	0.46804503	1
Adamts12	1.06577232	0.091899266	0.46844169	1
Mgst3	1.03901332	0.055214154	0.46847224	1
Rtn1	0.9663488	-0.049384072	0.46878269	1
Chi3l1	1.10501343	0.14406391	0.46886075	1
Plpp7	1.09523299	0.131237809	0.46898116	1
Brinp3	1.11708573	0.159739913	0.46899558	1
Myl12b	1.03522745	0.049947783	0.4690659	1
Sart1	0.95334493	-0.068929802	0.4691453	1
Smc4	1.04367597	0.061673872	0.46919079	1
Ccl7	0.85413542	-0.227463277	0.46920473	1
Capg	1.04928414	0.069405409	0.46936464	1
Zfp192	1.0617872	0.086494661	0.46948956	1
Hsh2d	1.30341748	0.382299249	0.46951738	1
Atp5g2	1.04013176	0.056766302	0.46957347	1
Ring1	1.07024052	0.097935063	0.4696374	1
Fat3	0.95200657	-0.070956563	0.46978045	1
Gak	0.96519347	-0.051109935	0.46978214	1
Tmem132b	0.90170912	-0.14926598	0.46981896	1
Sap18	1.05447067	0.076518969	0.46982248	1
Ddhd2	0.96053313	-0.058092715	0.46991919	1
Eef1g	1.03387335	0.048059472	0.47003975	1
Cyp2d4	0.9124661	-0.132157129	0.47021487	1
Lmnb1	1.14250797	0.192204228	0.47028533	1
Brd4	0.96063744	-0.057936061	0.47034997	1
Gtf2a1	0.91054699	-0.135194619	0.47044849	1
Cct5	1.03387256	0.048058362	0.47057217	1
Gatad2b	0.92741528	-0.108712596	0.47066375	1
Syt4	0.96723229	-0.048065686	0.47078467	1
Bloc1s4	1.0829355	0.114947322	0.47094254	1
MGC105567	1.10460654	0.143532573	0.4709929	1

Bub1b	0.8210933	-0.284381933	0.47102575	1
LOC300308	1.09726105	0.1339068	0.47115505	1
Nup153	0.96124388	-0.057025585	0.47132041	1
Tmem71	1.28425824	0.360935335	0.4713526	1
Col18a1	0.95301816	-0.069424393	0.47148855	1
Cyc1	0.9666047	-0.049002086	0.47151106	1
Atg4d	1.05278256	0.074207498	0.47155616	1
Whamm	0.90589114	-0.142590408	0.47166333	1
Aqr	0.96329792	-0.053946039	0.47179171	1
Paip2	1.03350664	0.047547655	0.47180573	1
Ube2s	0.95900616	-0.060388014	0.47181686	1
Isl1	1.05188841	0.072981668	0.47191746	1
LOC689574	0.95037903	-0.07342509	0.47194183	1
Tmub1	0.9341519	-0.098270938	0.4719808	1
Kctd17	0.95452305	-0.067148051	0.47208864	1
Stam	1.03660438	0.051865392	0.47229792	1
Bmi1	1.07062297	0.098450512	0.47252313	1
Axl	1.04488399	0.063342775	0.47254017	1
Nes	0.96672084	-0.048828748	0.47255165	1
Dennd1c	0.88817883	-0.171077914	0.47259031	1
Tst	1.06655207	0.092954403	0.47312369	1
Zswim7	1.16623051	0.221852976	0.47315019	1
Lgi4	1.03476307	0.049300465	0.47333817	1
Rnf185	0.95109979	-0.072331374	0.47336544	1
Aldh7a1	0.95318214	-0.069176176	0.47340291	1
Cabp1	1.03510734	0.049780377	0.47361559	1
Acad9	1.04414261	0.062318775	0.47362287	1
Zdhhc12	0.90236908	-0.14821046	0.47387315	1
Hpse	0.95301583	-0.069427918	0.47387831	1
Ago1	0.9484779	-0.076313935	0.4739063	1
Slc39a11	0.86996191	-0.200975866	0.4739512	1
Csnk1g3	1.03600824	0.051035476	0.47398164	1
Cherp	0.94666435	-0.079075097	0.47409027	1
Elf1	1.07126334	0.099313166	0.47417294	1
Naa10	0.93188972	-0.101768861	0.47423691	1
Def6	0.91587236	-0.126781542	0.47428158	1
Cep290	1.04773758	0.067277421	0.47430569	1
Gtf3c2	1.04452202	0.062842909	0.47449393	1
Usp24	0.96201563	-0.055867758	0.47452824	1
Tmem263	1.09309196	0.128414775	0.47455933	1

Cops6	0.96380588	-0.053185489	0.47456936	1
H2afy	0.95401637	-0.067914074	0.47481112	1
Tmem147	0.94131497	-0.087250558	0.47489549	1
Zbtb49	0.8842134	-0.177533499	0.47498604	1
Dennd6a	1.04199077	0.059342494	0.47506856	1
Pknox1	0.90055036	-0.151121132	0.47510212	1
Tll1	1.05498274	0.077219399	0.4751482	1
Rcl1	0.92445828	-0.113319883	0.47515973	1
Chmp7	0.96096972	-0.057437119	0.47536807	1
Erbb4	0.85323182	-0.228990331	0.47544199	1
Gng3	0.96762177	-0.047484867	0.47559863	1
Cndp1	1.06747531	0.0942027	0.47561904	1
Calcrl	1.06856208	0.095670721	0.47581839	1
Rassf8	0.92297134	-0.11564225	0.47604359	1
Phka1	1.05201695	0.073157946	0.4761438	1
Crebrf	1.03782239	0.053559573	0.47632071	1
Upf2	1.04610184	0.065023302	0.4764745	1
Capza1	0.96717431	-0.048152178	0.47653987	1
Dnajc6	1.03317364	0.04708274	0.47672388	1
Serpina11	1.08138068	0.112874493	0.47681161	1
Slc2a1	1.03740233	0.052975513	0.47707455	1
Vkorc1l1	0.9241862	-0.11374455	0.47722193	1
Atpaf1	1.04570206	0.064471853	0.47723274	1
Tspan12	1.03519988	0.049909357	0.47733159	1
Fcho1	0.94244889	-0.085513717	0.47737749	1
Ube2j2	0.95560115	-0.065519499	0.47746832	1
Snd1	0.96487009	-0.051593391	0.47757208	1
Lmbrd1	1.03361049	0.047692614	0.4775746	1
Dusp11	1.0698868	0.097458156	0.47770106	1
Hsph1	0.96709301	-0.048273449	0.4777312	1
Bbs4	1.0494108	0.069579548	0.47786116	1
Emb	1.04184196	0.059136446	0.47809275	1
Tgfbi	1.03427912	0.048625582	0.4781132	1
Psap	1.03532557	0.050084514	0.47821917	1
Ckmt2	0.88144297	-0.182060871	0.47830903	1
Dgkq	1.04176247	0.059026366	0.47845607	1
Sh3gl2	1.03545825	0.050269388	0.47851006	1
RT1-Ba	1.04742271	0.066843787	0.4785192	1
Ttc1	1.03410647	0.048384733	0.47854828	1
Tnpo2	0.96754596	-0.047597907	0.47861782	1

Rhbdd2	0.94249813	-0.085438346	0.47864411	1
Otud7b	1.04115442	0.058184066	0.47871751	1
Fan1	0.92242318	-0.11649933	0.47877021	1
Traf3ip3	0.85571673	-0.224794795	0.47891088	1
Cbx3	1.03682975	0.052179021	0.47893254	1
RGD1563888	1.14475567	0.195039707	0.47909238	1
Magt1	1.06375226	0.0891622	0.47922799	1
F11r	1.06334165	0.088605212	0.47923467	1
Cdk17	1.05386375	0.075688365	0.479332	1
Inpp4b	1.10104204	0.138869555	0.4794046	1
Slc35d1	0.93431199	-0.098023707	0.47955657	1
Atf6	1.04257449	0.060150468	0.47956902	1
Iffo1	0.96169706	-0.056345593	0.47982915	1
Htra3	1.04960691	0.069849121	0.4800109	1
C1ql3	1.1314104	0.178122343	0.48010992	1
Ankrd12	0.96585383	-0.050123219	0.48019085	1
Smo	0.93238299	-0.101005408	0.48022829	1
Tp53inp1	1.11508869	0.157158467	0.48033096	1
Eml2	1.03653729	0.051772013	0.48075855	1
Fam118a	1.05974026	0.083710702	0.48102534	1
Prkcd	0.96602171	-0.049872483	0.48112869	1
Coq4	1.10760684	0.14744587	0.48116099	1
Zfp9	1.08338941	0.115551899	0.48124773	1
Vav3	1.05811017	0.081489847	0.4812669	1
Anapc2	1.0340653	0.048327286	0.4813072	1
Atxn7l3b	0.96833209	-0.046426182	0.48146326	1
Ilk	0.96746187	-0.047723286	0.48149758	1
Mrps21	1.05333529	0.074964741	0.48174175	1
Tgfbr2	0.96238147	-0.055319232	0.48184964	1
Prkcg	0.86139665	-0.215250389	0.48200426	1
Rnf5	0.95601131	-0.064900407	0.48211973	1
Vps13b	0.96593229	-0.050006028	0.48229868	1
Tnnc2	0.85200883	-0.231059715	0.48232855	1
Elk3	1.06093008	0.085329586	0.48238003	1
Fcgr1a	1.10198618	0.140106135	0.48250782	1
Vps28	0.9615969	-0.056495852	0.48259522	1
Kcns3	1.03450472	0.048940229	0.48267425	1
Trmt5	1.0584075	0.081895186	0.48315313	1
RGD1307752	0.95754267	-0.062591322	0.48316066	1
Grin3b	0.83635119	-0.257819227	0.48320197	1

Rassf9	1.14618036	0.196834085	0.48322268	1
Kcns1	1.04198901	0.059340062	0.48329343	1
Farsa	0.95285485	-0.069671639	0.4833548	1
Gpatch4	0.89127872	-0.166051435	0.48336286	1
Herc2	0.9683263	-0.046434821	0.48339532	1
Rell1	1.09075199	0.125323106	0.48353875	1
Cep85	1.05152977	0.072489693	0.48363977	1
Fbxo7	1.04134923	0.058453972	0.48373541	1
Ppfia1	0.96058132	-0.058020337	0.48378674	1
Lrguk	1.11348834	0.155086457	0.48378942	1
LOC501110	1.07346469	0.102274734	0.48384138	1
Rab43	1.16043868	0.214670285	0.48390214	1
Cdk16	0.9679784	-0.046953242	0.48391587	1
Dtnbp1	1.0786692	0.10925249	0.48392361	1
Cdc45	0.8242111	-0.278914205	0.48413745	1
Dhx32	0.95343991	-0.06878608	0.48423125	1
Hsbp1l1	0.84705545	-0.239471674	0.48426728	1
Nova1	0.92145427	-0.118015523	0.48427459	1
Gpc4	1.07075751	0.098631792	0.48437714	1
Dnah1	0.88618984	-0.174312314	0.48446685	1
Gpat3	1.09944129	0.13677057	0.48447584	1
Tut1	0.93983032	-0.089527791	0.48455789	1
Ccnd1	1.04058375	0.05739308	0.48477956	1
Lancl2	0.95594135	-0.065005984	0.48514704	1
Inha	0.90841046	-0.138583775	0.48522711	1
Ppp4r3b	1.03850801	0.054512342	0.48538811	1
Tfe3	0.95124994	-0.072103633	0.48564417	1
Fam134b	0.96819345	-0.046632767	0.48573217	1
Thap11	0.93537004	-0.096390872	0.48577467	1
Slc16a12	1.07345744	0.102264993	0.4858596	1
Tor1aip2	0.96400483	-0.052887713	0.48587052	1
Tatdn2	0.95727538	-0.062994091	0.48593682	1
Rtbdn	0.88353676	-0.17863793	0.48603467	1
Clcn2	1.05038953	0.07092444	0.48607517	1
Eaf1	0.96220759	-0.055579921	0.48624957	1
Rmdn3	1.04659719	0.065706288	0.48635504	1
Trappc2l	1.05281286	0.074249019	0.48641008	1
Gorasp2	0.96627531	-0.049493795	0.4864967	1
Serpinb6b	0.86374022	-0.211330625	0.48654541	1
Smg6	1.05533431	0.077700095	0.48674249	1

Lima1	1.05730102	0.080386184	0.48688757	1
Arrb1	0.9517496	-0.071346032	0.48691775	1
Khdrbs1	0.96601746	-0.049878834	0.48705293	1
Ppip5k1	1.04610095	0.06502208	0.48709207	1
Ddi2	0.95837543	-0.061337175	0.4871869	1
Dtd2	0.90401198	-0.145586204	0.4872496	1
LOC498154	0.92132281	-0.118221359	0.48732356	1
Ppia	1.03414079	0.048432617	0.4875208	1
Slc27a3	1.07509063	0.104458278	0.48760639	1
Rps18	1.03620973	0.051316036	0.4882122	1
Ppp2r3a	1.04784232	0.06742164	0.48827976	1
Sepw1	1.03222498	0.045757455	0.48845627	1
Arl8b	0.9684333	-0.046275402	0.48850999	1
Tmem175	1.04726618	0.06662818	0.48876586	1
Mospd2	0.94996924	-0.07404729	0.48876972	1
Ralgps2	1.06186283	0.086597411	0.48883485	1
Dhx34	0.93101242	-0.103127682	0.48884681	1
Csnk1g1	0.88700334	-0.172988562	0.48890956	1
Dnajc2	1.04287463	0.060565729	0.48896308	1
Vps54	1.04612777	0.065059062	0.48898293	1
Acap3	0.9556106	-0.065505243	0.4890543	1
Dnpep	1.03641316	0.051599244	0.48930479	1
Invs	1.06638274	0.092725335	0.48934135	1
Nop2	1.05676387	0.079653051	0.48936538	1
Hapln2	1.09919459	0.136446805	0.48939706	1
Sigmar1	0.9661991	-0.049607588	0.48942852	1
Gimap6	1.13904476	0.187824443	0.48948542	1
Sptlc3	0.94506565	-0.081513546	0.48957699	1
Caly	1.07715333	0.107223634	0.48962515	1
Lpar3	1.06770393	0.094511653	0.48962832	1
Tardbp	1.03320141	0.047121523	0.48968201	1
Arntl2	1.15188196	0.203992879	0.48972172	1
Sytl1	0.83459164	-0.260857634	0.4897516	1
Cpsf7	0.95863874	-0.060940855	0.48980037	1
Serpinf1	0.96750984	-0.047651753	0.48982913	1
Fam167a	1.08966598	0.123885962	0.48988229	1
RGD1309104	0.95811659	-0.061726866	0.48989462	1
H3f3b	1.03223502	0.045771488	0.48995345	1
Prepl	1.03213191	0.04562737	0.49002906	1
Sptb	0.9670329	-0.048363116	0.49012315	1

Slc30a4	0.95454364	-0.067116933	0.49013814	1
Rbbp7	1.03252871	0.046181893	0.49018573	1
Ndufaf1	0.94642713	-0.079436662	0.49031193	1
Lynx1	0.96833118	-0.046427538	0.49042691	1
Slc19a1	0.92308307	-0.115467607	0.49056802	1
Entpd6	0.95559308	-0.065531696	0.49064812	1
Rnf44	0.94261035	-0.085266575	0.49084124	1
RGD1311747	0.95405208	-0.067860068	0.49096079	1
Setd6	1.12030595	0.163892777	0.49100786	1
Snrnp35	0.94249992	-0.085435603	0.49106334	1
Pik3c2b	1.07137144	0.099458739	0.49110712	1
Sdccag8	0.92359095	-0.114674052	0.49119089	1
Suox	1.05712257	0.080142665	0.49119099	1
Zfp644	0.96331058	-0.05392709	0.49120137	1
Serpinb9	1.03473254	0.049257912	0.49120307	1
Rragc	0.9673298	-0.04792025	0.491349	1
Med12	0.95922783	-0.060054579	0.491385	1
Cdk20	0.91685009	-0.125242229	0.49178317	1
Nsfl1c	1.03346968	0.04749607	0.49180449	1
Hk1	0.96900644	-0.045421843	0.49182917	1
Rxra	0.91782983	-0.123701393	0.49204749	1
Cacna2d3	0.96564109	-0.050441027	0.49205469	1
Fam117a	0.93839036	-0.091739907	0.49228733	1
Chrnb3	0.96078395	-0.057716047	0.49234127	1
Wdr37	0.96378946	-0.053210069	0.49242879	1
Arid1a	0.96340039	-0.053792594	0.49256732	1
Renbp	1.09130756	0.126057749	0.49262305	1
Srsf2	0.9683659	-0.046375811	0.49264351	1
Rab11fip1	1.10161791	0.139623926	0.49269204	1
Nmt2	1.04555579	0.064270048	0.49279874	1
Mrvi1	1.06487113	0.090678847	0.49281952	1
Tspan6	0.95428884	-0.067502099	0.49284255	1
Cmbl	0.94097253	-0.087775492	0.49287062	1
Gpr52	1.17176783	0.228686743	0.49287934	1
Chst14	0.89377121	-0.162022513	0.49290546	1
Tpi1	1.03230829	0.045873886	0.49306476	1
Olfm1	0.96893473	-0.045528607	0.49331493	1
Fbll1	0.93924275	-0.090430025	0.49342698	1
Usp14	1.03229897	0.045860858	0.49346086	1
Rab29	1.06621134	0.092493432	0.49347368	1

Cplx1	1.03322063	0.04714836	0.49352375	1
Asic3	1.03465919	0.049155634	0.49361221	1
Eif6	1.04127338	0.058348894	0.49361382	1
Tceb1	1.03321508	0.047140609	0.49373079	1
Magi1	0.96391602	-0.053020637	0.49378187	1
Wdr82	1.04285798	0.060542694	0.49382647	1
Cacna1a	0.9651814	-0.051127976	0.49387329	1
RGD1566099	0.86940414	-0.201901121	0.49390523	1
Ndufs8	0.95977344	-0.059234204	0.49397934	1
Wipf2	1.07746289	0.107638187	0.49402231	1
Cd320	1.05780151	0.081068941	0.49402689	1
Nmi	1.08136663	0.112855738	0.49408479	1
Myo7a	1.16601656	0.221588284	0.49426868	1
Camkk1	1.03828166	0.05419786	0.49429422	1
Plcl2	1.07052828	0.098322913	0.49431099	1
Sgms1	0.95193687	-0.07106219	0.49438583	1
Ano4	0.96638704	-0.049326981	0.49442166	1
Micu1	1.03265674	0.046360772	0.49456623	1
Lgals1	1.03262992	0.046323311	0.4946064	1
Fam104a	1.04092862	0.057871137	0.49469424	1
Odf2l	1.08007942	0.111137402	0.49487306	1
Fchsd1	0.94848841	-0.076297951	0.49488511	1
Ubac1	0.96370068	-0.053342965	0.49489743	1
Glcci1	0.94486961	-0.081812836	0.4948975	1
Osgepl1	1.05465541	0.076771705	0.49490144	1
Rad51ap1	0.88557373	-0.175315665	0.49491665	1
Ncstn	0.96594277	-0.049990378	0.49501523	1
LOC100912071	0.84862934	-0.236793543	0.49505682	1
Fam101b	1.07474104	0.103989084	0.49507544	1
Zfp286a	1.11457618	0.156495232	0.49508967	1
Fam234b	0.96568125	-0.050381022	0.49512763	1
Scrn3	0.94181244	-0.086488316	0.49518198	1
Scarb1	0.951627	-0.071531887	0.49519848	1
Zdhhc16	1.06894085	0.096182027	0.49533948	1
Dnajc14	0.96263861	-0.054933806	0.49534174	1
Adrbk2	0.92008721	-0.120157484	0.49537316	1
Rpl5	1.03168782	0.045006486	0.49554637	1
Fam120a	0.96863304	-0.045977883	0.49564631	1
Rbbp8	1.04993075	0.070294178	0.49586824	1
Sema6c	1.06817952	0.095154123	0.49593006	1

Ncbp1	0.96168146	-0.056368984	0.495932	1
Mthfd1l	0.95863724	-0.06094311	0.49594349	1
Apbb3	1.03826386	0.054173136	0.49606973	1
Asnsd1	1.03292456	0.046734896	0.4960915	1
Syngr3	0.9664411	-0.049246286	0.49617337	1
Bag2	1.08309677	0.115162148	0.49621895	1
Zfp496	1.07038234	0.098126223	0.49623991	1
Siah2	0.91250722	-0.132092127	0.49628175	1
Abi3	0.86187162	-0.214455103	0.49630301	1
Aldh3a1	1.1214551	0.165371864	0.49633722	1
Arl4c	0.95769743	-0.06235816	0.49657103	1
Ube2e2	1.05886735	0.08252186	0.49671252	1
Brf1	0.94343668	-0.084002406	0.49672591	1
Sgol2	0.86330873	-0.212051515	0.49679629	1
Golt1b	0.94758443	-0.077673603	0.49687724	1
Acadsb	0.95788255	-0.06207932	0.49702429	1
Tasp1	0.9311376	-0.102933716	0.49712662	1
Phb2	0.96309186	-0.054254683	0.4971405	1
Lonrf3	0.87650881	-0.190159504	0.49725602	1
Anapc15	1.06616213	0.092426837	0.49742009	1
Rpl41	0.96891246	-0.045561773	0.49742972	1
Fstl1	1.0334416	0.047456858	0.49748604	1
Lcp1	1.03211653	0.045605871	0.49749613	1
Slc35e4	1.05326973	0.074874937	0.49759602	1
Tmf1	0.96694171	-0.048499169	0.49767306	1
Map3k10	0.95838351	-0.061325013	0.49776057	1
Tyro3	0.96106497	-0.057294133	0.49788822	1
Prokr2	0.89323516	-0.162888059	0.49801645	1
Itgb4	1.03239823	0.045999572	0.49802876	1
Klhl6	0.87616273	-0.190729252	0.49814664	1
Celf3	1.12945561	0.175627572	0.49817711	1
Abcg2	1.0471446	0.066460677	0.49823975	1
Naa40	0.90587413	-0.142617499	0.49830494	1
Pnisr	1.0325171	0.046165672	0.49833356	1
Pdhb	1.03174713	0.045089432	0.49843793	1
Wbp4	0.95372906	-0.068348624	0.49855012	1
Tada1	1.06278943	0.087855787	0.49855148	1
Pphln1	1.04450467	0.062818946	0.49857693	1
Mphosph6	1.06346187	0.08876831	0.49860704	1
Zc3h3	0.93393284	-0.098609282	0.49873608	1

Arfgef2	0.96759344	-0.047527102	0.49880488	1
Gzf1	1.05614022	0.078801385	0.49881542	1
Adat1	0.89203357	-0.164830087	0.49882457	1
Sh3gl3	0.92532569	-0.111966846	0.49906552	1
Hist1h4b	1.05537418	0.077754588	0.49915463	1
Atp5c1	1.03172559	0.0450593	0.49916936	1
Zc3h12a	0.87872747	-0.186512296	0.49917147	1
Cnep1r1	1.04837431	0.068153901	0.49924968	1
Gtpbp3	0.91662602	-0.125594851	0.49927845	1
Srr	0.93671893	-0.094311881	0.49930837	1
Ncoa1	0.96279964	-0.054692486	0.4993372	1
Btrc	0.95998804	-0.058911659	0.49935895	1
Tbcc	0.94665488	-0.079089532	0.49937525	1
Rrp1	0.95672997	-0.063816301	0.49948723	1
Rtn2	0.96623888	-0.049548185	0.49950004	1
Vwa9	1.04398813	0.062105311	0.49981542	1
Cr1l	1.04001007	0.056597498	0.49994974	1
Lpar1	0.96745183	-0.047738259	0.49999775	1
Yy1	0.94003918	-0.089207211	0.50004196	1
Aldh1l1	1.12002615	0.163532421	0.50011921	1
Zfp251	1.09214375	0.127162762	0.50014893	1
Ppil4	1.05770088	0.08093169	0.50020245	1
Smarce1	0.96306543	-0.054294274	0.5002036	1
Trim47	0.91881945	-0.122146703	0.50024938	1
Lilra5	1.16163644	0.216158618	0.50025804	1
Hfe	1.12877653	0.174759902	0.50028362	1
Eml5	0.93031857	-0.104203268	0.50034136	1
Ralbp1	0.96756701	-0.047566521	0.50050819	1
Mllt3	0.93516431	-0.096708229	0.50095561	1
Snapc5	0.94605135	-0.080009606	0.50102761	1
Polr2d	1.07411021	0.103142027	0.50124264	1
Met	1.1172446	0.159945072	0.50130571	1
Pld2	1.05015107	0.070596876	0.50133293	1
Slc4a7	0.91699617	-0.12501238	0.5016252	1
Slc25a39	0.96588275	-0.050080028	0.50163823	1
Traf3ip1	0.92005269	-0.120211617	0.50166682	1
Ctdsp1	0.96343761	-0.053736845	0.50169399	1
B4galnt4	0.94565823	-0.080609217	0.50173862	1
Prodh	1.05392722	0.075775239	0.50175375	1
Zbtb11os1	0.89116375	-0.166237539	0.50177411	1

Cyp2t1	0.9005598	-0.151106011	0.50186157	1
Psmb7	1.03131373	0.044483279	0.50195028	1
Dyx1c1	1.16946377	0.225847164	0.50200354	1
Wwc2	0.95596704	-0.064967223	0.50207422	1
Jkamp	1.03658452	0.051837751	0.50230468	1
Pcdh7	1.0429721	0.060700565	0.50235699	1
Fam131b	0.96855608	-0.046092507	0.50251416	1
Faxc	1.05763719	0.080844808	0.50260878	1
Gsap	1.09983549	0.137287741	0.50281885	1
Oasl2	1.10113436	0.138990516	0.5028489	1
Fam181b	0.90432623	-0.145084782	0.50292593	1
Iqcb1	1.0542115	0.076164336	0.50296664	1
Plvap	1.07368234	0.102567215	0.50315261	1
Sh3bp4	1.04643014	0.065476002	0.50322389	1
Calca	0.96853621	-0.046122108	0.50326666	1
Prkar1a	1.03128258	0.044439694	0.50326896	1
Puf60	1.03303891	0.046894589	0.50334723	1
Reep5	0.96810271	-0.046767983	0.50341245	1
Camta2	0.96770368	-0.047362749	0.5034234	1
Itga4	0.90960067	-0.13669477	0.50347006	1
Celf1	0.95034343	-0.073479128	0.50347813	1
Zscan21	0.93900923	-0.090788763	0.50352772	1
Ctxn1	0.95033151	-0.073497232	0.50353129	1
Mcm3ap	0.96475204	-0.051769903	0.50353983	1
Snca	1.03496886	0.049587356	0.50358377	1
Trim45	1.07161005	0.099780022	0.50373616	1
Clk4	0.94958636	-0.074628879	0.50382229	1
Pex11g	1.20695736	0.271374703	0.50395522	1
RGD1563348	1.06596768	0.092163699	0.50398037	1
Zfp91	0.96874019	-0.045818295	0.50411013	1
Lbr	1.08659792	0.119818185	0.50430195	1
Slco5a1	0.95200509	-0.070958806	0.5044286	1
Galk2	1.05786398	0.081154141	0.50443335	1
Dus2	0.92949347	-0.105483362	0.50450669	1
Afap1l1	0.94392787	-0.083251478	0.50452987	1
Wdr75	1.04813858	0.067829477	0.5045574	1
Afg3l2	1.03585461	0.050821524	0.50456244	1
Napepld	0.896765	-0.157198116	0.50472192	1
Rpf2	1.05731394	0.080403808	0.50475007	1
Med18	1.08300115	0.115034773	0.5049884	1

Ubiad1	0.9170321	-0.124955857	0.50506569	1
Apc	0.96987083	-0.044135471	0.50506793	1
Gmppb	0.93933408	-0.090289744	0.50506886	1
Ercc2	1.05880808	0.082441108	0.50513048	1
Hk3	1.31096001	0.390623678	0.50515531	1
Habp4	0.96470634	-0.051838252	0.50518069	1
Pycrl	1.06155024	0.086172652	0.50528626	1
Zmat4	0.9130712	-0.13120073	0.50530908	1
Dok4	0.96935385	-0.0449047	0.50531892	1
Gas2	1.04918221	0.069265244	0.50539701	1
Fnip2	1.07333477	0.102100123	0.50549372	1
Mrps36	1.04140801	0.058535402	0.50550335	1
Ssfa2	1.03128101	0.044437496	0.50567627	1
Etf1	0.96677169	-0.048752866	0.50568298	1
Slc16a14	0.90741397	-0.140167225	0.50570742	1
Patz1	0.94601958	-0.080058057	0.50575105	1
Ubxn2b	0.91719382	-0.124701464	0.50575441	1
Slc35a1	0.94700276	-0.07855946	0.50576196	1
Rmnd5b	0.94307483	-0.084555852	0.50578664	1
Ezh2	1.09597903	0.132220189	0.5058581	1
Atp13a1	0.9583536	-0.061370041	0.50589055	1
Pllp	0.95929014	-0.059960865	0.50599278	1
Mtch1	0.9699528	-0.044013548	0.50606076	1
Bbof1	1.10597082	0.145313326	0.50607169	1
Pacs1	0.95420562	-0.067627915	0.50607604	1
Polr3gl	0.90320046	-0.146881875	0.50616347	1
Calcoco1	1.03901501	0.055216493	0.50636453	1
Atn1	0.96082997	-0.057646948	0.50657663	1
Ddx58	1.06490531	0.09072515	0.50666417	1
P2ry12	1.11689233	0.159490111	0.50671804	1
Mif4gd	1.08926798	0.12335893	0.50690489	1
Cd248	1.06627767	0.092583187	0.50696889	1
Mavs	1.05309889	0.074640915	0.50718914	1
Asgr1	1.16836979	0.22449696	0.50728867	1
Msl3l2	0.89838708	-0.154590921	0.50735763	1
Apoo	0.95693642	-0.063505023	0.50755964	1
Rab8a	0.95900777	-0.060385596	0.50765732	1
Dhrs11	1.08912095	0.123164175	0.50778656	1
Cln8	1.06011524	0.084221102	0.50781378	1
C1qc	1.04294407	0.060661793	0.50784692	1

Prkcb	0.96775277	-0.047289557	0.50786702	1
Hjurp	1.17760571	0.235856578	0.50787498	1
Sft2d3	1.09614074	0.132433047	0.50800847	1
Sf3a3	1.04350486	0.061437318	0.50806999	1
Cd164l2	1.12628	0.171565535	0.50809663	1
Mrpl32	1.0522912	0.073534	0.5081424	1
Rasgrp3	1.08072911	0.11200495	0.50829287	1
Mlec	1.04217625	0.059599285	0.50835175	1
Susd2	0.96937783	-0.044869012	0.5083941	1
Cstf2t	0.95134344	-0.071961833	0.50868662	1
Nap1l2	1.03132352	0.044496967	0.50877509	1
RGD1306954	1.04834177	0.068109129	0.50877944	1
Myd88	1.07399533	0.102987725	0.5088	1
Snx8	0.93333913	-0.099526711	0.50883219	1
Gpaa1	0.95501854	-0.066399356	0.50888739	1
Stx11	0.89697397	-0.15686197	0.50891675	1
Sprtn	0.90650703	-0.141609887	0.50901791	1
Trpt1	1.08182706	0.113469888	0.50921278	1
Slc24a3	0.96035871	-0.058354711	0.50934224	1
Aurka	0.92107231	-0.118613667	0.5093507	1
Btbd10	0.96025914	-0.058504299	0.50945797	1
Pfas	1.04775219	0.067297532	0.50949337	1
Tpm4	0.9658556	-0.050120586	0.50959083	1
Prpf8	0.97013761	-0.043738696	0.50959515	1
Fat4	0.93841214	-0.091706415	0.50959642	1
Commd7	1.05814428	0.081536361	0.5096938	1
Nebl	1.0557884	0.07832072	0.5098689	1
Tbc1d10a	0.94374141	-0.083536489	0.51011008	1
Nol10	1.04954892	0.069769417	0.51020212	1
Hspa12a	0.96902133	-0.045399673	0.51025444	1
Cib1	0.93666813	-0.094390117	0.51033246	1
Sccpdh	1.03092166	0.043934711	0.51034623	1
Plxna2	0.9698415	-0.044179101	0.51034726	1
Lrrn1	1.03090307	0.043908693	0.51034848	1
Brms1l	0.96312791	-0.05420068	0.51040754	1
Fam69b	1.03715714	0.0526345	0.51042164	1
Aass	1.08604718	0.119086773	0.51060833	1
Trpm4	1.06141609	0.085990328	0.51063218	1
Rgs5	0.93878007	-0.091140882	0.51063612	1
Sub1	0.9678365	-0.047164751	0.51079984	1

Mcmbp	0.94921693	-0.075190268	0.51085366	1
Ggta1	1.05414743	0.076076657	0.51099087	1
Cep57	0.94434618	-0.082612269	0.51099272	1
Foxj2	1.04923231	0.069334142	0.5111024	1
Snai1	0.87915482	-0.185810848	0.51111638	1
Ap3s2	1.03499357	0.0496218	0.51113336	1
Pak2	1.04238978	0.059894844	0.51126744	1
Rap2c	0.96686085	-0.048619818	0.51132987	1
Arfgap3	1.03775613	0.053467448	0.51139005	1
Drosha	0.96531686	-0.050925519	0.51142357	1
5-Mar	0.961612	-0.056473197	0.51144585	1
Tp53i3	0.93058358	-0.103792359	0.51144932	1
Nipa1	1.03755535	0.0531883	0.5117824	1
Topors	1.04730177	0.066677205	0.51187211	1
Ppard	0.94368066	-0.08362936	0.51190477	1
Psmd1	1.03075686	0.043704059	0.51193706	1
Kcnk1	1.03492465	0.049525732	0.51212324	1
Golph3	1.03228188	0.045836969	0.51214537	1
Mmachc	1.07094963	0.098890626	0.51214927	1
Ints5	1.0603088	0.084484488	0.51218638	1
Ppef1	1.03138101	0.044577383	0.51221108	1
Gabrb3	1.04475898	0.063170157	0.51226853	1
Rasa2	1.05876008	0.082375703	0.51236848	1
Katnbl1	1.05211266	0.073289202	0.51244566	1
Rbl1	0.87285917	-0.196179185	0.51246826	1
Sgsm3	1.04528418	0.063895224	0.51258892	1
Thap1	0.90686312	-0.141043284	0.51260172	1
Ap1m1	1.03428093	0.048628098	0.51265395	1
Cbfb	1.03052349	0.043377387	0.51265407	1
Igf1r	0.93406588	-0.098403793	0.51265801	1
Ndufb10	1.03231123	0.045877999	0.51267466	1
Kctd5	0.96192659	-0.056001291	0.51267689	1
Khk	0.94397948	-0.083172595	0.51270753	1
Cfi	1.07234089	0.100763608	0.51271898	1
Clptm1l	0.96523008	-0.051055221	0.51274881	1
RGD735065	0.95932591	-0.059907068	0.51275588	1
Tarsl2	1.05516695	0.077471277	0.51294466	1
RGD1307621	0.90175616	-0.149190724	0.51300417	1
Ddx47	1.04180331	0.05908293	0.51317768	1
Fbxw8	0.95934615	-0.059876641	0.51332217	1

Col4a4	0.85668546	-0.223162486	0.51332702	1
RGD1305089	1.05850283	0.082025132	0.51353884	1
Ifitm2	0.9602582	-0.058505715	0.5136108	1
Chchd6	1.04556636	0.064284636	0.513615	1
Zmiz2	0.96499507	-0.051406519	0.51363063	1
Ift43	0.92055402	-0.119425709	0.51369105	1
Kcnt2	0.93579203	-0.095740151	0.51371283	1
Kifc1	1.15843735	0.212180029	0.51372808	1
Capns1	1.03076091	0.043709727	0.51378367	1
Usp25	0.96722291	-0.048079682	0.51383679	1
Ift27	1.06497968	0.0908259	0.51383778	1
Nars	1.03075558	0.043702266	0.51405428	1
Prpf19	1.03034817	0.043131922	0.51420439	1
Pcnp	1.04355327	0.061504252	0.51439038	1
Fam8a1	0.96993565	-0.044039053	0.51442197	1
Cygb	0.94867692	-0.076011251	0.51448154	1
Ephx4	0.87849856	-0.186888181	0.51449652	1
St5	1.04493536	0.063413694	0.51480228	1
Gdi1	1.03119743	0.044320571	0.5148067	1
Tusc5	0.96989442	-0.04410038	0.51500409	1
Esco1	1.0467806	0.065959094	0.51506583	1
Alg11	1.04258411	0.060163771	0.51508241	1
Eif2ak1	1.03534055	0.050105386	0.51530302	1
Rita1	0.95625731	-0.064529218	0.51545082	1
Spast	0.9622952	-0.055448565	0.51568961	1
Slpi	1.19832235	0.26101605	0.51572154	1
Kcne3	1.13822532	0.186786183	0.51599136	1
Kit	0.95895803	-0.060460423	0.51601743	1
Agtr1a	0.91666912	-0.125527021	0.51623417	1
Ccar1	0.96665532	-0.048926533	0.51623826	1
Camk2n1	1.07687528	0.106851166	0.5164498	1
Impdh1	0.97056736	-0.043099748	0.51647198	1
Nat8l	0.97067812	-0.042935125	0.51661376	1
Arhgap15	1.11372622	0.155394629	0.51663706	1
Otc	1.19784544	0.260441762	0.51663963	1
Surf4	1.03175537	0.045100948	0.51664345	1
Snx3	0.97073906	-0.042844545	0.51664996	1
Mre11a	0.93636433	-0.094858112	0.51666873	1
Mybl2	0.81499279	-0.295140801	0.5166927	1
RT1-DMb	1.07393008	0.102900067	0.51671321	1

Rb1cc1	1.03049647	0.043339565	0.51693147	1
Arpp19	1.04081691	0.057716303	0.51710827	1
Prrt1	1.05578918	0.07832179	0.51711923	1
Acat2	0.96411043	-0.052729691	0.51722363	1
Nubpl	0.91866178	-0.122394279	0.51734191	1
Large	0.97055488	-0.043118309	0.51746953	1
Rabac1	0.9695587	-0.044599847	0.51748848	1
Copb2	1.03225871	0.045804593	0.51752251	1
Slc5a3	0.90314847	-0.146964928	0.5175557	1
Pex13	0.95120776	-0.072167616	0.517682	1
Uqcrfs1	1.03227718	0.045830403	0.51777792	1
Kcnmb1	1.21581332	0.281921729	0.51796517	1
Wdr61	1.0417085	0.05895163	0.51798084	1
Rexo2	0.96462425	-0.05196102	0.51799111	1
Edf1	1.03164095	0.044940951	0.5180214	1
Kif2a	0.96785262	-0.047140723	0.51813021	1
Rp9	1.12329991	0.167743167	0.51814069	1
Trim46	1.05042787	0.070977102	0.51821353	1
Zdhhc9	0.96134836	-0.056868789	0.51829329	1
Zmym3	0.96634817	-0.04938501	0.51831318	1
Gtpbp6	0.92574695	-0.1113102	0.51835503	1
RGD1565784	0.95061303	-0.073069913	0.51837028	1
Cys1	0.93771769	-0.092774444	0.51860631	1
Pdgfb	1.102076	0.140223712	0.51864246	1
Gde1	1.03232354	0.045895193	0.51867053	1
Fam101a	1.13430742	0.18181169	0.51884049	1
Cct6a	0.97042251	-0.043315072	0.51889571	1
Csnk1e	0.95120058	-0.072178508	0.51895413	1
Lhfpl2	0.96538814	-0.050818997	0.51898176	1
Cd300le	1.09835831	0.13534877	0.51908751	1
Acsl3	0.96877303	-0.045769388	0.51926472	1
Entpd7	1.11037114	0.15104197	0.51931937	1
Xpo7	1.03251815	0.046167146	0.51933878	1
Lcat	1.05728802	0.080368434	0.51935966	1
Banf1	1.04143059	0.05856669	0.51941396	1
Apbb1	0.97095718	-0.042520427	0.5195312	1
Eif4e	1.03319112	0.047107156	0.51954977	1
Bst1	0.89955726	-0.152712974	0.51958982	1
Jak3	1.08709904	0.120483385	0.51959261	1
Dynlrb1	1.03000215	0.042647351	0.51971167	1

Tmem53		1.09149934	0.126311261	0.5197172	1
	3-Sep	1.05306532	0.074594933	0.51995294	1
Med1		1.044221	0.062427084	0.51995895	1
Cops7a		1.03272595	0.04645746	0.52003581	1
Gls		0.97065022	-0.042976596	0.52003981	1
Stxbp3		1.04269768	0.060320917	0.52008736	1
Caskin2		0.96240562	-0.055283022	0.52015735	1
Aars2		1.08127408	0.112732255	0.52020926	1
Mapk3		0.97048703	-0.043219159	0.52034624	1
Efemp1		1.03148243	0.044719244	0.52036991	1
Stmn1		0.97103205	-0.042409183	0.52045563	1
Srrm3		0.94999068	-0.074014739	0.52046867	1
Lrrc4		1.06387032	0.089322308	0.52056773	1
Laptm5		1.04864487	0.068526178	0.52067958	1
Rnf2		0.94734855	-0.078032773	0.52068883	1
Zfp672		1.05876946	0.082388486	0.52100209	1
Adra1b		1.14649476	0.197229761	0.52129614	1
Ash2l		1.04004911	0.056651654	0.52138124	1
Ptgr2		0.95283336	-0.069704172	0.52140132	1
Mier1		1.03749677	0.053106845	0.52153405	1
Cand1		0.97110137	-0.04230619	0.52162352	1
Ehhadh		1.09814232	0.135065045	0.52168926	1
Dcaf5		0.96563344	-0.050452457	0.52179635	1
Pak7		1.11493981	0.156965831	0.52179856	1
Ece2		1.03732192	0.052863687	0.52185382	1
Il31ra		1.04549758	0.064189728	0.52203155	1
Myl6b		1.03555125	0.050398955	0.52212555	1
Slirp		1.05043108	0.070981504	0.52215229	1
Hbs1l		1.03019578	0.042918535	0.52229311	1
Tspan15		0.95762168	-0.062472287	0.52266048	1
Cacna1i		0.90001453	-0.151979806	0.52266702	1
Ubac2		0.95566411	-0.065424456	0.52280098	1
Ttc30a		1.13754165	0.18591937	0.52287396	1
Pcdhga5		0.9056365	-0.14299599	0.52287638	1
Mien1		1.03844497	0.054424766	0.52290481	1
Arl6ip1		1.02998886	0.042628731	0.52293582	1
Kcnj14		0.90905449	-0.13756132	0.5230007	1
Nup98		0.96516533	-0.051152007	0.52320101	1
Pop5		1.08055113	0.111767341	0.52320993	1
Ptpn7		1.19443562	0.256329091	0.52321371	1

Arhgap5	0.96531426	-0.050929406	0.52324398	1
Plce1	0.96237463	-0.055329486	0.52333703	1
Rnf6	0.96725792	-0.048027458	0.52335691	1
Fer	1.06804568	0.094973347	0.52339569	1
Cdyl2	0.91884921	-0.122099964	0.52339926	1
Mfsd13a	0.936994	-0.093888283	0.52340099	1
Dnaja2	1.03012624	0.04282115	0.52340596	1
Zfp280d	1.0411158	0.058130543	0.52343392	1
Alkbh4	0.88785912	-0.171597325	0.52344962	1
Slc7a5	0.96051085	-0.05812619	0.52374362	1
Kirrel	0.93815957	-0.092094764	0.52380381	1
Sema7a	0.97038553	-0.043370059	0.52397127	1
Acot3	0.92578423	-0.111252104	0.52408964	1
Adamts10	0.95934009	-0.059885751	0.52411034	1
Lgi3	1.02989388	0.042495688	0.52415172	1
Gpr135	0.86686498	-0.206120794	0.52415331	1
Kctd15	1.0522568	0.07348684	0.52427355	1
Acacb	1.07328979	0.102039659	0.52443632	1
Apeh	1.03702223	0.052446827	0.52450048	1
Mmp17	0.9329587	-0.100114877	0.52461598	1
Cmpk1	1.03027465	0.043028982	0.52471347	1
RGD1563349	0.97003605	-0.043889734	0.52472364	1
Klhl26	0.94614255	-0.079870538	0.52476226	1
S1pr3	1.03105444	0.044120511	0.52480915	1
Ap3b1	0.964676	-0.051883614	0.52482073	1
Itpr1	1.04868362	0.06857949	0.5248557	1
Hps5	1.04472537	0.063123751	0.52497176	1
Slc39a3	0.93357529	-0.099161714	0.52506035	1
Epsti1	1.12669664	0.172099127	0.52515907	1
Cept1	0.96603556	-0.049851801	0.52522394	1
Stard13	0.96168885	-0.056357909	0.52557961	1
Pigc	1.04715999	0.06648188	0.52561752	1
Vstm2b	1.0620167	0.086806447	0.52575469	1
Phf2	0.95199356	-0.070976279	0.52576605	1
Pdik1l	0.9378748	-0.092532746	0.52585568	1
Tmed4	0.96400429	-0.05288853	0.52587132	1
LOC100910827	0.97084998	-0.042679718	0.52602928	1
Lias	1.05196195	0.073082516	0.52613679	1
Dhx37	0.94011158	-0.089096101	0.52633834	1
Cd46	1.12964899	0.175874558	0.52646463	1

Ppp1r8	1.04764753	0.067153419	0.52650165	1
Gli1	1.05258836	0.073941349	0.52668455	1
Slc35a2	0.95428934	-0.06750134	0.52674456	1
Bfsp1	0.89210146	-0.164720293	0.52677649	1
Pttg1ip	1.03091704	0.04392824	0.52679854	1
Lgalsl	0.96889546	-0.045587083	0.52687899	1
Lysmd2	1.03885811	0.054998623	0.52688672	1
Tbc1d14	1.03269152	0.046409366	0.52696115	1
Psma4	1.03113224	0.044229373	0.52705918	1
Kif13b	1.03914161	0.055392266	0.52710409	1
Wdr54	1.04765738	0.06716698	0.52717692	1
Nuak2	1.1348997	0.1825648	0.52747084	1
Ctsk	0.9582714	-0.061493781	0.52770935	1
Zfp868	1.07474127	0.103989392	0.52773357	1
Tmem63c	0.97001265	-0.043924526	0.52774138	1
RGD1310429	1.04278619	0.06044338	0.52794216	1
Arfip1	1.05011716	0.070550294	0.52799365	1
Lmtk2	0.96131964	-0.056911886	0.52845597	1
Ptprc	0.95507312	-0.066316898	0.52852551	1
Gng5	0.95419807	-0.067639325	0.52853841	1
Yeats2	1.05311946	0.074669092	0.52857157	1
Pcdha13	1.06622198	0.092507834	0.5285854	1
Egfem1	1.11338883	0.154957509	0.52865661	1
Palmd	1.0412493	0.058315531	0.52878509	1
Htr2c	1.11322559	0.154745982	0.52887078	1
Sbsn	1.11660379	0.159117356	0.52898137	1
Cdk6	1.11314951	0.154647381	0.5290617	1
Armc1	1.03569324	0.050596753	0.52907161	1
Txnrd2	1.0714298	0.099537323	0.52911152	1
Slc33a1	0.95613739	-0.064710162	0.52920411	1
Sugp2	0.96683267	-0.048661875	0.52922415	1
Slc37a2	1.11195389	0.153096959	0.52924821	1
Wisp2	1.05219109	0.073396734	0.52931185	1
Apmap	0.96123315	-0.057041691	0.52931839	1
Carhsp1	0.96475372	-0.051767389	0.52947871	1
Aldh1b1	0.89691301	-0.156960023	0.52954778	1
Nmrk1	0.92901899	-0.10622001	0.52957026	1
Adnp2	0.95081145	-0.072768821	0.52957878	1
Col4a3	0.89966425	-0.152541405	0.52961076	1
Ttc3	1.03050964	0.043357998	0.52963295	1

Tex10	1.04576237	0.06455506	0.5296628	1
Lrfn5	0.96403896	-0.052836649	0.52972039	1
Pfdn1	0.96898537	-0.045453215	0.52985637	1
Tcta	1.03722171	0.052724302	0.52990541	1
Dgcr2	0.96057879	-0.058024136	0.53017403	1
Trib2	0.9678134	-0.047199184	0.53025302	1
Sec11a	0.95923757	-0.060039931	0.53026537	1
Cdkn2aipnl	1.04227792	0.059740017	0.53031333	1
Irf1	1.06434346	0.089963785	0.5303145	1
RGD1306063	0.95811202	-0.061733758	0.53035436	1
Suclg1	1.03001362	0.042663416	0.53042484	1
Oser1	1.04664433	0.065771269	0.53043195	1
Vezf1	1.03893366	0.05510354	0.53050703	1
Vgll4	0.95274404	-0.069839413	0.53067268	1
Ntsr2	1.11092381	0.151759882	0.53067943	1
Ehd3	0.97174525	-0.041349947	0.531166	1
Fgfrl1	0.8973886	-0.156195246	0.53126185	1
Pcdha3	0.93833561	-0.091824083	0.53132125	1
Rab3ip	0.95770183	-0.062351535	0.53134	1
Ide	0.96589136	-0.050067163	0.5316389	1
Trip13	0.84642492	-0.240545995	0.531688	1
Tbce	1.03310217	0.046982941	0.53176336	1
Kiaa0895l	0.96136755	-0.056839982	0.53182758	1
Gtf2h1	0.96142823	-0.056748925	0.53203545	1
Ppp4r2	0.96710572	-0.048254492	0.5320518	1
Ucp2	0.97077565	-0.042790175	0.53213508	1
Kcnab1	1.0290396	0.041298509	0.53224379	1
Trmt12	1.07516989	0.104564646	0.53232417	1
B3galt4	0.8865167	-0.173780292	0.53240346	1
Ccdc102a	0.94409677	-0.082993344	0.53250192	1
Sec14l5	0.88287789	-0.179714189	0.53260718	1
Tmem67	1.05194886	0.073064572	0.53277027	1
Bach1	0.9488609	-0.075731483	0.53282429	1
B3gat2	0.93892323	-0.090920899	0.53289796	1
Lrrk2	1.03268652	0.046402383	0.53295864	1
Mpc2	1.04895026	0.068946273	0.5331193	1
Tsga10	0.92468393	-0.112967776	0.5332205	1
Klhl36	1.0995123	0.136863746	0.53341003	1
Ogfod3	1.0870261	0.120386576	0.53343425	1
LOC654482	1.09952207	0.136876568	0.53372074	1

Cma1	1.201132	0.264394711	0.53374993	1
Snurf	0.97181843	-0.041241309	0.53397077	1
Serinc2	0.94191847	-0.086325912	0.53397341	1
Cxxc5	0.96056475	-0.058045221	0.53397475	1
Vps4b	1.03000341	0.042649113	0.53398881	1
Sspn	1.04445246	0.062746823	0.53400721	1
Kbtbd3	1.03778654	0.053509724	0.53415541	1
Gstt3	0.9548846	-0.066601711	0.534318	1
G4	0.93025408	-0.104303275	0.53436642	1
Cacul1	0.96852062	-0.046145331	0.53440315	1
Rbm42	0.96009874	-0.058745312	0.53457881	1
Dpagt1	1.03546658	0.050280997	0.53501259	1
Pias4	0.94940077	-0.074910868	0.53507903	1
Smg9	0.93967217	-0.08977057	0.53511579	1
Prkar2b	1.02995914	0.042587107	0.53512426	1
Fbxo25	0.9589801	-0.060427212	0.53544877	1
Cask	0.96239376	-0.055300809	0.53568002	1
Fxr2	0.97116485	-0.042211892	0.53583353	1
Trove2	1.04397322	0.062084705	0.53583649	1
Rpl22	1.04222213	0.059662788	0.53587506	1
Ankrd50	1.03836014	0.054306902	0.53591347	1
Slc9a8	0.95110276	-0.072326874	0.53593094	1
Cln3	1.0610131	0.085442467	0.53595637	1
Mcm8	1.08317974	0.115272662	0.53602061	1
Grk4	0.89438069	-0.16103906	0.53619432	1
Nudt19	0.94292747	-0.08478129	0.5362095	1
Ankrd13a	0.96704096	-0.048351103	0.53632954	1
Pcyt2	0.96264824	-0.054919369	0.53632978	1
Elfn1	0.94679329	-0.078878611	0.53640911	1
Pofut1	0.91511395	-0.127976697	0.53647365	1
Slc1a5	1.09867277	0.135761754	0.53648375	1
Tmem18	0.93936188	-0.090247048	0.5366439	1
Rraga	1.02966499	0.042175022	0.53677778	1
Llgl2	1.13337984	0.180631448	0.53689705	1
Trhde	1.11050433	0.151215014	0.53704843	1
Hcfc2	0.94470605	-0.082062604	0.53705709	1
Prkch	1.07261148	0.101127602	0.53719175	1
Msra	0.93452769	-0.097690684	0.53735671	1
Creld1	0.96911367	-0.045262198	0.53737124	1
Spns1	0.95923556	-0.060042949	0.5374973	1

Dhrs7b	1.04858201	0.068439695	0.53770421	1
Cxcl16	1.05364809	0.075393101	0.53778199	1
Ets1	1.04054136	0.057334307	0.53782348	1
Chid1	0.95444923	-0.067259643	0.53786024	1
Ccdc12	0.94139316	-0.087130732	0.53790722	1
Tp53rk	0.91258605	-0.131967489	0.53793489	1
Src	0.95089586	-0.072640744	0.53795122	1
Dmd	0.96586709	-0.050103421	0.5379848	1
Tmem63a	1.08314004	0.115219781	0.53805635	1
Snap29	1.03395505	0.048173473	0.53814281	1
Kdm5b	0.96239648	-0.05529673	0.53820504	1
Zic5	1.2280799	0.296404422	0.53822479	1
Pafah1b3	1.05686203	0.079787056	0.53825348	1
Cep41	1.09116425	0.125868279	0.53829686	1
Cd2ap	1.0335646	0.047628557	0.5385387	1
Srebf2	0.9710285	-0.042414456	0.53888841	1
Slc20a2	1.04271489	0.060344738	0.5389638	1
Acot9	1.03416715	0.048469382	0.53901887	1
Arhgdia	0.97168356	-0.04144153	0.53924025	1
Sh3tc1	1.12279315	0.167092173	0.53927068	1
Rlim	1.04282614	0.060498657	0.53938929	1
Ebna1bp2	1.04134526	0.058448474	0.53945897	1
Ttc13	0.95724347	-0.063042187	0.53957391	1
Lmo2	1.06905261	0.096332853	0.54002701	1
Tmod2	0.96997553	-0.043979739	0.54005777	1
Ddx3y	1.08372978	0.116005079	0.54007935	1
RGD1560010	1.07030388	0.098020467	0.54014896	1
Zfhx4	0.94207855	-0.086080736	0.54015496	1
Extl1	1.14496266	0.195300554	0.5402699	1
Cecr2	1.09615366	0.132450051	0.54028374	1
Gfm1	0.96269375	-0.054851171	0.54042227	1
Osr2	1.13310312	0.180279161	0.54050835	1
Spag7	0.95955313	-0.059565412	0.54056365	1
Zfp386	1.05991322	0.083946154	0.54085922	1
Lats1	1.03941238	0.055768148	0.54092662	1
Tle4	0.96428378	-0.052470314	0.54093453	1
Hbb	1.02999875	0.042642593	0.54101326	1
Ctif	0.96188666	-0.056061186	0.54111354	1
Hacd1	0.88353256	-0.178644796	0.54113878	1
Magee2	0.90152177	-0.149565766	0.54117536	1

Sesn2	0.92860662	-0.10686053	0.54118348	1
Epdr1	1.02847355	0.040504687	0.54119608	1
Cyth1	1.05598785	0.078593232	0.54124371	1
Mycn	1.1241974	0.168895388	0.54127695	1
Zfp319	0.92455479	-0.113169274	0.54130211	1
Pdcd5	1.03683738	0.05218964	0.54146779	1
Tnrc18	1.03959711	0.056024529	0.54149616	1
Hdgfrp3	1.03702358	0.052448697	0.54150684	1
Cdc123	1.03515902	0.049852404	0.54158443	1
Prcc	0.95950179	-0.059642604	0.54160339	1
Zadh2	1.05060459	0.071219794	0.54167839	1
Tm4sf4	1.17526942	0.232991522	0.54185573	1
Erap1	0.9659115	-0.050037091	0.54189355	1
Kif3a	1.02841073	0.040416563	0.54212035	1
Bbs2	0.96047453	-0.058180744	0.5421305	1
Mms22l	1.15979443	0.21386911	0.54215011	1
Ggct	1.08202044	0.113727755	0.54249196	1
Vamp7	1.04710974	0.066412645	0.5425024	1
Nrarp	1.04865173	0.068535622	0.54253674	1
Lrba	0.95737283	-0.062847224	0.5425518	1
Znrd1	1.07696289	0.106968543	0.5425596	1
Ppp2r2c	0.96771927	-0.04733951	0.54257266	1
LOC100233176	0.95543965	-0.06576334	0.54259001	1
Tmed2	0.97162707	-0.041525409	0.54269979	1
Pnpla2	0.96172997	-0.056296219	0.54280418	1
App	0.97162026	-0.041535524	0.5428386	1
Hip1r	0.96421571	-0.052572161	0.54289699	1
Prpf40b	0.96071815	-0.05781485	0.54299531	1
Morc2	0.95855716	-0.061063636	0.54301793	1
Stub1	1.02962116	0.042113608	0.54305131	1
Hmgb1	0.96799457	-0.046929142	0.54306877	1
Sumf1	0.96383053	-0.053148593	0.54311304	1
Gpld1	0.92777915	-0.108146664	0.54325236	1
Plxnd1	1.03249786	0.04613879	0.54338005	1
Ssh3	1.04796963	0.067596902	0.54347489	1
Idh3g	1.02952101	0.041973271	0.54353169	1
Hnrnpa2b1	1.02872721	0.040860468	0.54364909	1
Coq7	0.93130942	-0.102667531	0.54365548	1
Ppp1r16b	0.93108985	-0.103007706	0.54381374	1
Ksr1	0.94691943	-0.078686417	0.54400534	1

Stx3	0.9039386	-0.145703322	0.54411002	1
Ophn1	1.0395545	0.055965394	0.5441196	1
Gxylt1	0.96153867	-0.056583214	0.5442544	1
Fgf10	1.13107642	0.177696406	0.54440451	1
Dock8	1.0644444	0.0901006	0.54441535	1
Kcnh7	0.96540659	-0.050791416	0.54452612	1
Pnpo	1.04324274	0.061074882	0.54456489	1
Atp2b1	0.97264534	-0.040014253	0.54457052	1
Clcnkb	1.13340096	0.180658333	0.54465454	1
Rbm28	0.9526868	-0.069926095	0.54470631	1
Adprh	0.95789391	-0.062062212	0.54479916	1
Bin1	0.97134043	-0.04195108	0.54483427	1
Cast	1.02820105	0.04012239	0.54484164	1
Ewsr1	0.96966283	-0.044444906	0.54497462	1
Wnk4	1.05298653	0.074486983	0.54511829	1
Rhpn1	0.96216726	-0.055640385	0.54519482	1
Tomm40	0.95823015	-0.06155589	0.54533727	1
Hint1	1.02893261	0.041148491	0.5454341	1
Rheb	1.02911035	0.041397682	0.54552689	1
Irs3	1.11764074	0.160456516	0.54559708	1
Usp46	0.96966694	-0.044438792	0.54561579	1
Kpna1	0.96275306	-0.054762299	0.54566214	1
Slc25a16	0.9621329	-0.055691909	0.54566614	1
Spcs2	0.96916403	-0.045187234	0.54572098	1
Trim26	0.95943266	-0.059746546	0.54578819	1
Tbc1d10c	0.87265606	-0.19651494	0.54588483	1
Trpc6	0.92194581	-0.117246142	0.54592174	1
Ybx1	0.96196372	-0.055945604	0.54603135	1
Mob2	0.95956207	-0.059551967	0.54616714	1
Casr	0.94630441	-0.079623746	0.54617138	1
Crim1	0.94959235	-0.074619783	0.54642081	1
Taz	0.95293673	-0.069547668	0.54643157	1
Mical2	0.95801925	-0.061873446	0.54652922	1
Akirin2	1.03676435	0.052088018	0.54658182	1
Fancb	1.11276298	0.154146326	0.54664035	1
Pdrg1	1.0355695	0.050424376	0.54670077	1
Nos1ap	0.923757	-0.114414696	0.54672498	1
Uhrf2	0.96711306	-0.048243541	0.54678732	1
Cep112	0.90670578	-0.141293621	0.54681732	1
Nt5c	1.14868134	0.199978634	0.54710572	1

Slc34a2	0.88446394	-0.177124766	0.54720781	1
Scgn	1.24900507	0.320779332	0.54744834	1
Sqle	0.97249785	-0.040233038	0.54759966	1
Dpp3	0.96864732	-0.045956614	0.54766698	1
Zmynd11	1.03096114	0.04398996	0.54770154	1
Cacna1d	1.05911584	0.08286039	0.54779586	1
RGD1304567	1.05968236	0.083631881	0.54785082	1
Lrrfip2	0.96087133	-0.057584843	0.5479115	1
Tti1	1.04584499	0.064669042	0.5479359	1
Ptp4a1	0.97010612	-0.043785526	0.5479472	1
Zfp275	0.95069481	-0.072945814	0.54822405	1
Tmem17	1.06844915	0.095518252	0.54827005	1
Ppp1r3e	0.88972548	-0.168567833	0.54831383	1
Spire2	0.96462984	-0.051952661	0.54838612	1
Fundc2	1.04597345	0.064846233	0.54843932	1
S100a3	1.11592767	0.15824352	0.54844703	1
Ptbp2	1.03352934	0.047579343	0.5485348	1
Psmd6	1.02783804	0.039612953	0.54869015	1
Kansl2	0.96852785	-0.046134568	0.54909709	1
Rrm2b	0.94814233	-0.076824444	0.54914796	1
Fdft1	0.97298101	-0.039516443	0.54941083	1
Mettl6	1.06454898	0.090242328	0.549445	1
Pln	1.11192718	0.153062314	0.54947571	1
Spdya	1.13552195	0.183355596	0.54950637	1
Fam160b2	0.96468665	-0.0518677	0.54968948	1
Polr3e	0.9554139	-0.065802226	0.54978234	1
Rabif	1.04827667	0.068019531	0.55007151	1
Tmem200c	0.93846304	-0.091628166	0.55026068	1
Fiz1	1.05543855	0.077842584	0.55030157	1
Clspn	0.82025649	-0.285852985	0.55030497	1
Mtch2	1.02849352	0.040532697	0.55034703	1
Mast2	1.03195848	0.045384928	0.55035149	1
Knop1	0.92750594	-0.108571574	0.55053271	1
Tnni2	0.86481591	-0.209535038	0.55057871	1
Hspbap1	1.08760819	0.121158923	0.55059719	1
Atp6v0a1	0.97292518	-0.039599226	0.55063727	1
Diras2	1.06934346	0.096725299	0.55065171	1
Stk25	1.02903599	0.04129344	0.55077896	1
Tmbim4	1.0427444	0.060385559	0.55080586	1
Chrnb4	0.94338649	-0.084079156	0.55084632	1

Esam	0.94186346	-0.086410159	0.55094328	1
Hspa4l	0.97266836	-0.039980103	0.5509821	1
Itgb8	1.03946349	0.055839083	0.55100593	1
B3galt6	0.93317002	-0.09978813	0.55102106	1
Nr2c2ap	0.9500402	-0.073939541	0.55115186	1
Spidr	1.09024017	0.124645986	0.55122336	1
Ap2s1	1.02981822	0.042389696	0.5513996	1
Hagh	1.03140873	0.044616167	0.55145629	1
Slc35f2	0.94107289	-0.087621627	0.55169575	1
Chrm2	1.05451171	0.076575118	0.55174453	1
P4ha2	0.95439579	-0.067340413	0.55175312	1
Il13ra1	1.03638577	0.051561111	0.55176533	1
Pgrmc2	1.03389087	0.048083915	0.55177564	1
Gtf2e2	1.04589855	0.064742917	0.55179477	1
Cpne8	0.96264133	-0.054929729	0.55193886	1
Tubg1	0.96601894	-0.049876619	0.55203006	1
Xylt2	0.9640792	-0.05277643	0.55203637	1
Taf6	0.9556631	-0.065425987	0.55218468	1
Irf4	0.87456479	-0.193362828	0.55225599	1
Tmem222	1.0452324	0.063823747	0.55230855	1
Phrf1	0.96400242	-0.052891331	0.5524825	1
Ndufaf5	1.04616092	0.065104787	0.55254	1
Ppp1r16a	0.9518316	-0.071221742	0.55254763	1
Ltv1	1.04599635	0.064877811	0.55271017	1
Pigu	0.95582648	-0.065179364	0.55294367	1
RGD1311188	1.0915598	0.126391164	0.55304097	1
Stk36	0.92357787	-0.114694486	0.55307217	1
Gtpbp2	0.96050185	-0.058139695	0.55320205	1
Plscr4	1.04729833	0.066672457	0.55320943	1
Dscam	0.93611292	-0.095245523	0.55325155	1
Fam172a	1.03566961	0.050563842	0.55346345	1
Card9	0.88073586	-0.183218693	0.55360273	1
Oxsr1	0.97104545	-0.042389271	0.55361983	1
Kbtbd7	0.95577197	-0.06526163	0.55373598	1
Rbmxrtl	0.96084809	-0.057619737	0.55381213	1
Arnt	0.95028046	-0.07357473	0.55382942	1
Tmem37	1.172217	0.229239661	0.55384033	1
Erlin2	1.04421903	0.06242435	0.55406577	1
Spon1	0.96116242	-0.057147853	0.55408072	1
LOC688869	1.02819177	0.040109375	0.55415771	1

Adck2	1.05353008	0.075231511	0.55425712	1
Casc5	1.0770898	0.10713854	0.5546215	1
Etv6	0.89753658	-0.15595735	0.55474441	1
Csnk1a1	0.97088955	-0.042620915	0.55502678	1
Ftl1	1.02811588	0.040002888	0.55504873	1
Six4	1.03099229	0.044033542	0.5553315	1
Myh1	0.91974743	-0.120690352	0.5553493	1
Romo1	1.04507928	0.063612396	0.55539971	1
Zfp444	0.93552489	-0.096152063	0.55542515	1
Depdc1	1.19630221	0.258581889	0.55545678	1
Sobp	0.94500719	-0.081602795	0.55573628	1
Trmt1	0.95892755	-0.060506271	0.55584322	1
Aar2	0.9512687	-0.072075182	0.55596985	1
Kdelc1	1.05780504	0.081073758	0.55598243	1
Prmt1	0.96114164	-0.057179036	0.55621901	1
Cdk12	0.95961427	-0.059473485	0.55629665	1
Rai1	0.9622479	-0.055519475	0.55634776	1
Dcbld2	1.04504287	0.063562122	0.55639389	1
Smpdl3b	1.12254556	0.166773999	0.55654541	1
Tmem199	0.96593882	-0.049996281	0.55665403	1
Zfp281	0.94217263	-0.085936677	0.55666312	1
Supt6h	0.97316248	-0.03924739	0.55694837	1
Shisa4	0.95709331	-0.063268514	0.55716695	1
Duox2	1.11449588	0.156391284	0.55717396	1
Bbx	0.9393724	-0.090230896	0.55723702	1
Zbtb9	0.942353	-0.085660506	0.55723706	1
Txn1	1.02850554	0.040549563	0.55726983	1
Arhgef2	0.96247749	-0.055175302	0.55728242	1
Rft1	1.07305333	0.101721772	0.55747695	1
Slc12a9	0.93164311	-0.102150692	0.55749655	1
Loxl3	1.06969572	0.097200477	0.55752526	1
Abhd12	0.97353238	-0.038699135	0.55756433	1
LOC102549726	1.04900162	0.069016903	0.55770945	1
Crebbp	1.04238606	0.059889695	0.55779772	1
Pxk	0.96205456	-0.055809385	0.55780935	1
Foxp1	0.92504975	-0.112397131	0.55785798	1
Phykpl	0.88624404	-0.174224078	0.55792537	1
Med21	0.93885662	-0.091023242	0.55813477	1
Fam195b	1.03966347	0.056116618	0.55828281	1
Tmem14a	1.03181772	0.045188134	0.55848995	1

Kpna3	0.96224129	-0.055529392	0.55876129	1
Cdkn1b	1.03030122	0.043066184	0.55876609	1
Cdkn3	0.83909241	-0.253098387	0.55883099	1
Tra2a	0.96822812	-0.046581096	0.55892965	1
Psma1	1.02852692	0.040579548	0.55904061	1
mrpl11	0.95198382	-0.070991048	0.55906699	1
Armcx3	0.97338304	-0.038920459	0.55912319	1
Gnas	0.97238937	-0.040393971	0.55917899	1
Iffo2	1.1070909	0.146773679	0.55923281	1
Pcdha12	1.07960819	0.11050782	0.55930426	1
Cpped1	1.05310093	0.074643712	0.55948174	1
Zcchc10	1.09786497	0.134700628	0.55957123	1
Fsd1	0.94769362	-0.077507373	0.55967232	1
Snrpd1	1.04632919	0.065336815	0.55968435	1
Tagln2	1.02710077	0.038577739	0.55971351	1
Eps8l2	0.9070152	-0.140801371	0.55978514	1
Cbx8	0.91242386	-0.132223928	0.56005731	1
Stat3	0.97364254	-0.038535895	0.56007224	1
Slc4a10	0.87960266	-0.18507613	0.56011773	1
Pqlc2	1.10845215	0.148546494	0.56013175	1
Prkrir	1.03768485	0.05336835	0.56021117	1
Zfp112	0.91283055	-0.131581013	0.56028077	1
Vps37c	0.95968409	-0.059368525	0.56028144	1
Hps1	1.07127235	0.09932531	0.56049953	1
Sdad1	0.94212157	-0.086014861	0.56051469	1
Tmem25	1.03215691	0.045662312	0.56051653	1
Dclre1a	1.05564593	0.078126024	0.56052167	1
Desi2	0.93758238	-0.092982641	0.56078239	1
Mcoln1	0.96359738	-0.053497617	0.56106161	1
RGD1560394	0.91917132	-0.121594315	0.56107904	1
Zfp629	0.94517526	-0.081346224	0.56109023	1
Pter	1.10014838	0.137698118	0.56111327	1
Scyl2	1.02946658	0.041896997	0.5612228	1
Spen	0.96277825	-0.05472455	0.56128294	1
Stk19	1.06413804	0.089685315	0.56131326	1
Slc12a6	0.97073854	-0.042845328	0.56135031	1
Ccr1	1.11072002	0.1514952	0.5614178	1
Gabarap	0.97350594	-0.03873832	0.56142808	1
Pik3ip1	0.96746704	-0.047715582	0.56159442	1
Terf1	1.06048747	0.084727577	0.56161267	1

Rgs4	0.9724341	-0.040327607	0.56170074	1
Nprl3	0.95237278	-0.070401706	0.56176293	1
Ppm1f	0.96038829	-0.058310275	0.56185343	1
Uchl5	1.04303773	0.060791346	0.56187319	1
Dedd	1.04981585	0.070136291	0.56199962	1
Fgf18	1.08585585	0.118832589	0.56208744	1
Ubc	0.97261234	-0.040063204	0.56218382	1
Tyrobp	0.92710988	-0.109187763	0.56218797	1
L3mbtl2	0.96669809	-0.048862705	0.56232823	1
Mrpl43	1.04205606	0.059432893	0.5624038	1
Vdac2	1.02703941	0.038491541	0.56245938	1
Laptm4a	1.02719094	0.038704387	0.5626444	1
Nrp1	0.9668155	-0.048687495	0.56272153	1
Gsr	0.96765469	-0.047435785	0.56273158	1
Arse	1.07192801	0.100208021	0.56294146	1
Fbxo30	0.96209009	-0.055756106	0.56297864	1
Pbx1	1.05105499	0.071838149	0.5631638	1
Meis1	0.91388708	-0.129912182	0.56318861	1
Rab6b	0.97260905	-0.040068084	0.56364921	1
Mospd1	1.03624851	0.051370022	0.5636899	1
Ppp1r3a	1.30959532	0.389121075	0.56373619	1
Cdc16	1.03662275	0.051890956	0.5637897	1
Ptk2b	0.95785078	-0.062127177	0.56388204	1
Srsf6	0.97287685	-0.039670899	0.56388858	1
Esrra	0.96208921	-0.055757421	0.56395281	1
Atcay	1.02764969	0.039348551	0.56403495	1
Mns1	1.10571932	0.144985208	0.56403994	1
Tor2a	1.05857245	0.082120016	0.56423518	1
Fbxl3	1.03026573	0.043016492	0.5644462	1
Pde1b	0.90220459	-0.148473469	0.56466608	1
Mon2	0.97071312	-0.042883106	0.56473352	1
Lrrn2	0.96582373	-0.050168179	0.56488347	1
Mfsd14b	0.96676733	-0.048759367	0.56493266	1
Ns5atp4	1.12207698	0.166171658	0.56497485	1
Gpr165	1.13784803	0.186307887	0.56502826	1
Acot13	1.0638602	0.089308584	0.56507976	1
Snx30	1.06900371	0.096266856	0.56531838	1
Slc10a3	0.9487551	-0.075892361	0.56541517	1
Ywhae	0.97313521	-0.039287825	0.56545179	1
Erbb3	0.96949568	-0.044693621	0.56548795	1

Dstn	1.02696188	0.038382627	0.56549149	1
Fgd3	0.88460314	-0.176897728	0.56550586	1
Fnbp1	0.97176143	-0.041325921	0.56555692	1
Fip1l1	0.96078959	-0.057707581	0.56560418	1
Map2k1	0.97307838	-0.039372078	0.56573305	1
Tkt	0.97403459	-0.037955086	0.565765	1
Letm2	1.05546557	0.07787952	0.56580357	1
Morf4l2	1.02690063	0.038296582	0.56586215	1
Nbl1	0.9740929	-0.037868719	0.56596976	1
Pkmyt1	0.88014752	-0.184182746	0.56618053	1
Kdm5a	1.03125983	0.044407876	0.56620271	1
Sdccag3	0.96897859	-0.045463305	0.56634204	1
LOC690871	0.9318692	-0.101800629	0.56635744	1
RGD1304587	1.06165721	0.086318024	0.56639069	1
Exoc2	0.97314192	-0.039277881	0.56639786	1
Fbxl6	1.07911451	0.109847961	0.56643586	1
Rps24	1.02737037	0.038956369	0.56646396	1
Ppp1r9a	1.04767979	0.067197848	0.56655291	1
Acox3	0.96145305	-0.056711679	0.56656213	1
Ccnj	0.93663167	-0.09444628	0.56659585	1
Zfp949	0.92065067	-0.119274247	0.56663232	1
Gria3	0.95277024	-0.069799741	0.56663238	1
Fanca	1.08804765	0.121741744	0.56669447	1
Klf2	0.9310897	-0.10300794	0.56670708	1
Dopey2	0.97082213	-0.042721103	0.56677139	1
Plpp6	0.95318256	-0.069175538	0.56679456	1
Hist3h2a	1.04217316	0.059595003	0.566838	1
Senp3	1.03562862	0.050506737	0.56701856	1
Chkb	0.9597647	-0.05924734	0.56708623	1
Usp19	0.97215405	-0.04074315	0.5671609	1
Ppip5k2	1.04268478	0.060303074	0.5671745	1
Jph3	0.97270696	-0.039922849	0.56718145	1
Ccdc17	1.10256057	0.14085791	0.56727546	1
Gcs1	1.04823818	0.067966556	0.56729742	1
Zbtb46	0.92345548	-0.11488568	0.56730869	1
Ticam2	1.15210876	0.204276917	0.56733399	1
Zc3h12c	0.92302049	-0.115565414	0.56742992	1
Il18bp	1.10109642	0.138940812	0.56745574	1
Sbk1	0.96643692	-0.04925253	0.56748151	1
Spon2	1.07345826	0.1022661	0.56755467	1

Snx24	0.95732204	-0.062923773	0.56765165	1
St8sia4	0.92113275	-0.118519013	0.56781413	1
Mrps27	1.04892823	0.068915974	0.56785921	1
Acp1	1.02713215	0.038621812	0.56791172	1
Bcl3	1.10580436	0.14509616	0.56804045	1
Sec61a2	1.03118218	0.044299236	0.56806853	1
Rpp38	1.06943807	0.09685294	0.56809639	1
Usp1	1.04758285	0.067064352	0.56812325	1
RGD1309594	0.96440676	-0.05228633	0.56812885	1
Tbc1d22b	0.95523887	-0.066066555	0.56814731	1
LOC103689992	1.02864553	0.040745918	0.56829061	1
Rps9	1.02864553	0.040745918	0.56829061	1
Lime1	0.94019768	-0.088963973	0.56839917	1
Sh3glb1	0.96823532	-0.046570374	0.56850691	1
Tmem45b	1.03971111	0.056182716	0.56852834	1
Nckipsd	0.96532426	-0.050914465	0.56854512	1
Il1b	1.15160898	0.203650945	0.56855471	1
Gch1	1.14145188	0.190870036	0.56885696	1
Dock7	0.97188036	-0.041149368	0.56905009	1
Sec24c	0.97166944	-0.041462504	0.56907204	1
Apba2	0.9687458	-0.045809952	0.56908981	1
Rnf111	1.03285278	0.046634626	0.56915663	1
Dhodh	0.92014648	-0.120064543	0.56916246	1
Eral1	1.05064883	0.071280546	0.56925073	1
Acsl1	1.0281374	0.040033084	0.56944443	1
Mtfmt	1.06090994	0.08530219	0.56963326	1
Acadl	1.0278445	0.039622022	0.5696678	1
Ppp6r2	0.9699424	-0.044029016	0.56977881	1
Rundc3a	0.97362875	-0.038556321	0.56991113	1
Thbs2	0.95783282	-0.062154221	0.5699125	1
Dmtn	0.97398796	-0.038024161	0.56994962	1
Cct8	1.02655182	0.037806458	0.57000082	1
Mok	1.09706963	0.133655091	0.57001477	1
Ankrd46	1.02639353	0.037583982	0.57001992	1
Dck	1.06915244	0.096467572	0.57004017	1
Gar1	0.91826204	-0.123022183	0.57020206	1
Mturn	0.97336101	-0.038953108	0.57024367	1
Golga2	0.973078	-0.039372637	0.57027336	1
Mast3	0.96419034	-0.052610121	0.57052529	1
Fgfr1op	0.95231295	-0.070492348	0.57064761	1

Spats2	1.03582073	0.05077434	0.57083121	1
Usp32	0.97427342	-0.037601381	0.57086673	1
Psmd11	0.97209957	-0.040824	0.57091212	1
Aoc3	1.03492522	0.049526529	0.57095501	1
Myo10	1.03879727	0.054914122	0.57098888	1
Serpinb1a	0.9742585	-0.037623477	0.57105811	1
Sgta	0.96584845	-0.050131262	0.57106231	1
Ccm2	0.96266124	-0.054899897	0.57117676	1
Arfgef1	0.97436863	-0.037460405	0.57124211	1
Alg2	0.9737207	-0.038420079	0.57124913	1
Dusp7	0.95229042	-0.070526477	0.57126319	1
Mtbp	1.11853356	0.161608541	0.57126551	1
Nek4	1.05662967	0.079469823	0.57136999	1
Hoxd3	0.90726178	-0.140409213	0.57149077	1
Cd9	0.9740847	-0.037880867	0.57157328	1
Mau2	0.96637852	-0.049339711	0.57178828	1
Phf3	1.03254395	0.046203187	0.57183267	1
Stim2	0.97081031	-0.04273866	0.57186333	1
Arhgef40	0.97161959	-0.041536524	0.57189657	1
Rxrg	0.95110635	-0.072321423	0.5720005	1
Tmem138	0.91370595	-0.130198138	0.57203322	1
Eepd1	0.97265784	-0.039995715	0.57211826	1
Pdlim1	0.96704339	-0.048347472	0.57222004	1
B3galnt2	0.95799776	-0.061905812	0.57222203	1
Cdk2ap1	0.97213253	-0.04077508	0.57224362	1
Smu1	1.03612888	0.051203463	0.57232076	1
Cebpd	1.06217465	0.087021003	0.5723365	1
Tmem39a	1.05806304	0.08142559	0.57235775	1
Nr3c2	1.03691726	0.052300781	0.57235885	1
Apoe	1.02849168	0.04053012	0.57249422	1
Gal3st4	1.09397199	0.129575802	0.57253094	1
Tmem123	0.96043423	-0.058241277	0.57273658	1
Sprn	0.90808439	-0.139101721	0.57288885	1
Itch	1.03228778	0.045845227	0.57300011	1
Cdc42bpa	0.974013	-0.037987074	0.57302582	1
Mapk10	1.02613091	0.037214799	0.57306421	1
MGC95208	0.94155175	-0.086887701	0.57319175	1
Msl1	0.96950915	-0.044673581	0.57340775	1
Pkd2	0.96593365	-0.050003999	0.57343965	1
Slc8a2	1.06734973	0.094032971	0.57345655	1

Rpl31	1.02913931	0.041438287	0.57352084	1
Stox2	0.95139533	-0.071883151	0.5736721	1
Leng1	1.06080063	0.085153539	0.57374797	1
Ero1a	0.96413698	-0.052689958	0.5738475	1
Trip6	0.9393807	-0.090218146	0.57402967	1
Rps12	1.03562896	0.05050722	0.57403179	1
Mt3	1.02743618	0.039048782	0.57412001	1
Mcm5	0.88601291	-0.174600376	0.57420669	1
F8a1	1.04963297	0.069884947	0.57437055	1
Stk11	1.03831253	0.054240751	0.57447557	1
Ddx17	0.97368967	-0.038466062	0.57448489	1
Eef1d	0.97129833	-0.042013608	0.57454113	1
Mmp28	1.08154811	0.113097836	0.57461264	1
Swt1	0.95583488	-0.065166686	0.57462543	1
Btd	0.95859981	-0.060999446	0.5746564	1
Plrg1	1.03657628	0.051826281	0.57466097	1
Sfi1	1.04228036	0.059743398	0.57467986	1
Alg1	0.94653803	-0.079267616	0.57480295	1
Tomm70	1.02906464	0.041333607	0.57481144	1
Zfp180	1.02620093	0.03731324	0.57482822	1
Hps6	0.95115953	-0.072240765	0.57485809	1
Cpox	1.05090369	0.071630464	0.57500412	1
Sertad2	0.93327132	-0.099631539	0.57501339	1
Mff	1.02605005	0.037101104	0.57506825	1
Cstf1	0.95464519	-0.066963472	0.57510363	1
Gstm5	1.05081389	0.071507173	0.57510608	1
Abcf1	0.97076121	-0.042811638	0.57514611	1
Itgae	1.08371078	0.115979789	0.57517535	1
Usp42	0.95230195	-0.070509001	0.57526345	1
Rhbdf2	0.93503407	-0.096909168	0.57538482	1
Rem1	1.09621349	0.132528792	0.57543262	1
Tcerg1	1.02718774	0.038699889	0.57544063	1
Col5a1	1.02954242	0.042003275	0.57559147	1
Lrrc58	0.96369433	-0.053352475	0.57561102	1
Gna14	1.02714128	0.038634628	0.57565733	1
Rpl18	1.02785462	0.039636222	0.57570193	1
Hspa13	0.9610994	-0.057242442	0.57581417	1
Tgfb1	1.07247853	0.100948761	0.57603032	1
Ogfrl1	0.96791244	-0.047051555	0.57606524	1
Scamp4	0.96096896	-0.057438263	0.57628167	1

St3gal6	0.95983238	-0.059145611	0.57635325	1
Trappc6b	1.03081943	0.043791639	0.57642334	1
Csnk1d	0.97473957	-0.036911281	0.57646051	1
Rpn1	1.02591509	0.036911329	0.57664388	1
Endog	0.91444423	-0.129032906	0.57666613	1
Socs6	1.05459289	0.07668618	0.57670698	1
Mllt11	0.97465875	-0.037030903	0.57671103	1
Itga5	0.94183328	-0.086456387	0.5768276	1
Abca17	0.87508794	-0.192500098	0.57688556	1
Stx2	0.95766818	-0.062402226	0.5768949	1
Tmem97	0.95540727	-0.065812245	0.57694681	1
Rfesd	0.94915652	-0.075282085	0.57695608	1
Mmp11	0.9096015	-0.136693455	0.57699787	1
Thtpa	0.96886323	-0.045635069	0.57706628	1
Spry1	0.91142389	-0.133805902	0.57708249	1
Med9	1.04240826	0.059920415	0.57709534	1
Ddah2	1.0333787	0.047369053	0.57747823	1
RGD1311899	0.9727628	-0.039840036	0.57748286	1
Slc25a20	1.0340226	0.048267715	0.57754782	1
Prosc	1.04678794	0.06596921	0.57764483	1
Pacsin3	1.04849309	0.068317355	0.57769455	1
Gart	0.96698844	-0.048429456	0.57773157	1
Foxj1	0.9001266	-0.151800162	0.57789445	1
Ctps1	0.97168463	-0.041439944	0.57796595	1
Pcmt1	1.02614401	0.03723321	0.57800028	1
Nme2	1.02690555	0.038303495	0.57805907	1
Lilrb3a	1.12961091	0.17582593	0.57815544	1
Lilrb3b	1.12961091	0.17582593	0.57815544	1
Pde10a	0.94161675	-0.086788111	0.57818814	1
Uqcrb	1.03070235	0.043627764	0.57819515	1
Rcbtb1	1.02992294	0.042536395	0.57829925	1
Tnrc6b	1.02877247	0.040923947	0.57830332	1
Fbxl14	0.95714276	-0.063193973	0.57832699	1
Mrps2	0.94953893	-0.074700944	0.57858029	1
RGD1304810	0.92600161	-0.110913395	0.57858157	1
Card10	0.93185239	-0.101826652	0.57863887	1
Cysltr2	0.9415111	-0.08694999	0.57865751	1
Elf2	0.95745962	-0.062716456	0.57873086	1
Daxx	0.9539495	-0.068015196	0.57890985	1
Dr1	1.03562118	0.050496382	0.57930802	1

Samd14	0.96589178	-0.05006654	0.57934834	1
Xpot	0.9693037	-0.04497934	0.57964752	1
Cwc25	0.95281243	-0.069735865	0.57973996	1
Rilpl1	1.04736786	0.066768242	0.57987945	1
RGD1359108	1.02824316	0.04018147	0.57995579	1
Scg3	0.97450535	-0.037257995	0.58013153	1
Gnb5	0.96134995	-0.056866393	0.58026163	1
Emc8	0.94652199	-0.079292072	0.58041822	1
Pnrc2	1.03003889	0.042698814	0.58055441	1
Ctf1	0.90796865	-0.139285616	0.58062895	1
Ppme1	1.02637536	0.037558438	0.58078444	1
Asns	0.97173714	-0.041361979	0.58079044	1
Ostm1	0.96244719	-0.055220708	0.58082736	1
Frem1	1.12703636	0.172534065	0.58085956	1
Ptpn18	1.12212256	0.16623026	0.58111182	1
Kmt5a	0.95877765	-0.060731817	0.58141853	1
Prkab2	0.96043105	-0.058246052	0.58144561	1
Prmt2	0.96720363	-0.048108441	0.58152119	1
Ywhah	0.97345655	-0.038811511	0.58157014	1
Jrkl	1.05611082	0.078761224	0.5816204	1
Tsr3	1.05773234	0.080974595	0.58163722	1
Nphp4	0.94145927	-0.087029419	0.58174206	1
Fam173b	0.94884571	-0.075754587	0.58177576	1
Gtf2h3	0.94529463	-0.081164039	0.5819637	1
Dync2h1	0.96477303	-0.051738517	0.58201738	1
Akr1b1	1.02636359	0.037541898	0.58213207	1
Ptcd3	1.03337806	0.047368154	0.58235097	1
Bcl11a	1.12070502	0.164406595	0.58236599	1
Plekha7	1.0983052	0.135279006	0.58279439	1
Esf1	1.03780666	0.053537701	0.58290595	1
LOC500684	0.93294038	-0.100143206	0.58291623	1
Afg3l1	0.95833104	-0.061403999	0.5829942	1
Vezt	1.02689564	0.038289566	0.58301465	1
Ints12	0.93779273	-0.092659004	0.58304466	1
Rasal2	0.96237356	-0.055331095	0.58350645	1
Gtf2h5	0.96458072	-0.052026114	0.5836131	1
Sfrp1	1.03474486	0.049275078	0.58363799	1
Tmem117	1.030447	0.043270306	0.58376373	1
Gli3	1.11729124	0.160005303	0.5838962	1
Dtx3	1.04239616	0.05990367	0.58399271	1

Cited1	1.13070087	0.177217311	0.5840098	1
Zfp579	0.93043157	-0.104028041	0.58406007	1
Ppm1b	0.97407117	-0.037900911	0.5842504	1
Oprd1	0.88210372	-0.180979787	0.58455747	1
Fam160a1	1.19611938	0.258361386	0.58456304	1
Ccdc93	1.044531	0.062855309	0.58466407	1
Lck	0.90844264	-0.138532678	0.58467424	1
Fnip1	1.03398945	0.048221468	0.58479659	1
Jtb	1.04176647	0.059031913	0.5848986	1
Tmem69	1.04949459	0.069694725	0.58492301	1
Elmod2	1.03356891	0.047634578	0.58495741	1
Crkl	1.03484921	0.049420565	0.58500157	1
Cab39	1.02562556	0.036504118	0.58506398	1
Letm1	0.97093945	-0.042546769	0.58510276	1
Prdx3	1.02678977	0.038140834	0.58517944	1
Uba6	1.0271067	0.038586065	0.5853664	1
Cchcr1	0.95262972	-0.070012536	0.58541144	1
Insr	0.95424633	-0.067566367	0.58547879	1
Vopp1	0.97400812	-0.037994294	0.58549237	1
Ptpru	1.06241109	0.087342112	0.58550136	1
Tnpo3	0.970992	-0.042468693	0.58560489	1
Pls3	1.02566147	0.036554636	0.58561422	1
Atp1a4	0.94837165	-0.07647556	0.58577445	1
Eno2	1.02618111	0.03728538	0.58583677	1
Znrd1as1	1.09242427	0.127533268	0.5858823	1
Stard10	0.96863156	-0.045980082	0.58599749	1
Fcgr3a	0.87625719	-0.190573718	0.58607278	1
Wdr26	0.97395085	-0.038079133	0.58607761	1
Rassf3	0.95248319	-0.07023446	0.58614963	1
Wasf1	0.96782424	-0.047183015	0.58620017	1
Tsc1	1.04233153	0.059814228	0.58622451	1
Neto1	0.90383556	-0.145867783	0.58627421	1
Casp6	1.1054734	0.144664313	0.58639411	1
LOC498276	0.92352547	-0.114776339	0.58642586	1
Gpr174	0.86676746	-0.206283094	0.58651529	1
Ppt1	1.02950082	0.041944984	0.58657042	1
Mest	0.96559314	-0.050512665	0.58659542	1
Ddit3	1.04253715	0.060098798	0.58659716	1
Ccdc8	0.93871656	-0.091238491	0.58666469	1
Cldn22	1.09836405	0.135356305	0.58668465	1

Stk24		0.96528948	-0.050966433	0.58673353	1
Lancl3		1.08423593	0.116678719	0.58674085	1
Pi4ka		1.02524192	0.035964369	0.58681534	1
Timm21		0.95851464	-0.061127627	0.58702756	1
Stt3a		1.02730915	0.038870397	0.58720131	1
Aagab		1.05199103	0.073122408	0.58739106	1
Ccpg1		1.02587494	0.03685487	0.58767371	1
Stard3		0.96278663	-0.054711986	0.58773443	1
Mmaa		0.93348826	-0.099296215	0.58774197	1
Lmx1a		0.87765064	-0.188281317	0.58783198	1
Acsf2		1.04686557	0.066076196	0.58789687	1
Plin2		1.02950182	0.041946374	0.5879514	1
Ubfd1		1.04387688	0.061951568	0.58797523	1
Rfc5		1.07183049	0.100076763	0.58799323	1
Ppp2r2a		0.97202531	-0.040934216	0.58806155	1
Hps4		0.95573775	-0.065313298	0.58815287	1
Cfap20		1.03920537	0.05548079	0.58818837	1
Tnxa-ps1		0.95842496	-0.061262615	0.58820694	1
Psma7		1.02639666	0.037588376	0.58821119	1
	10-Mar	1.13103013	0.17763736	0.58825819	1
Insig2		1.06816786	0.095138387	0.58826126	1
Aco1		0.96696523	-0.048464083	0.5882891	1
Rnf40		1.03866403	0.054729067	0.58838967	1
Dpm3		0.94045164	-0.088574341	0.58839595	1
Nup205		1.03825793	0.054164896	0.5883965	1
Bcl2a1		1.12834029	0.17420223	0.58840991	1
H6pd		1.06154494	0.08616545	0.58855604	1
Brap		0.97430337	-0.037557043	0.58861934	1
Frk		1.09143503	0.126226253	0.58882491	1
Cisd1		1.02832009	0.040289404	0.58909297	1
Kif2c		1.1757297	0.233556425	0.589112	1
Klhl22		1.03764559	0.053313778	0.58911278	1
Mdh2		1.02511688	0.035788412	0.58928269	1
Ergic2		1.03435909	0.048737127	0.58938419	1
Ankrd16		1.08412088	0.116525625	0.58943896	1
Katna1		1.06256949	0.087557199	0.58953688	1
Nfib		0.9448833	-0.081791944	0.58970861	1
Hmgxb4		1.06722926	0.093870131	0.58973408	1
Mpp7		1.0601125	0.084217368	0.58985195	1
Eif5b		1.03081624	0.043787165	0.58997566	1

Akt1s1	0.96703883	-0.048354279	0.59019062	1
Snrpe	1.04717879	0.066507782	0.59022955	1
Trim16	1.04191366	0.059235735	0.59029847	1
Chrna6	1.03915352	0.055408801	0.59036817	1
Cops8	1.02731964	0.038885132	0.59039172	1
Dbndd1	1.05311636	0.074664851	0.59051824	1
RT1-CE5	1.03424401	0.048576597	0.5905535	1
Arl14ep	1.04397596	0.062088497	0.5906172	1
Celf6	0.95373167	-0.068344678	0.59066763	1
Acy3	0.93781282	-0.092628093	0.59069433	1
Bcat2	1.03219079	0.045709663	0.59082406	1
Pms1	1.05528555	0.077633436	0.59084435	1
Smco4	0.87777207	-0.188081726	0.59094974	1
Clasrp	0.96175646	-0.056256473	0.59105153	1
Smg7	0.97075653	-0.042818591	0.59107178	1
Snap25	1.02641959	0.037620612	0.59110377	1
Enoph1	0.96840627	-0.04631568	0.59113036	1
Gca	0.96220834	-0.055578786	0.59124364	1
Metrnl	1.05116359	0.071987212	0.59127512	1
Fen1	1.04918736	0.069272329	0.59133187	1
Gsta3	1.14375507	0.193778137	0.59148768	1
LOC361985	1.02881505	0.040983646	0.59150742	1
Taf7	0.94443203	-0.082481131	0.59170292	1
Pus3	1.05178599	0.072841188	0.59172442	1
Impact	0.97485502	-0.036740411	0.59198431	1
Ift20	1.03653922	0.0517747	0.59204095	1
Gipc2	0.92067943	-0.119229182	0.59219872	1
Dll4	0.91204964	-0.132815754	0.59223883	1
Cox15	1.03245517	0.046079147	0.5923095	1
Gpr139	1.10510083	0.144178012	0.59231432	1
Rbmx	0.9589233	-0.060512676	0.59249335	1
Hoxb4	1.09606468	0.132332935	0.59257516	1
Ptcd1	1.04857064	0.068424061	0.59261055	1
Taco1	0.92974849	-0.105087599	0.59266585	1
Magoh	0.95506055	-0.066335891	0.59266944	1
Spata5l1	0.91213485	-0.13268097	0.59267881	1
Nfkb2	0.94011345	-0.089093225	0.59268005	1
Casq1	0.89070081	-0.166987184	0.59272895	1
Apopt1	0.95756667	-0.062555158	0.59276338	1
Itpr2	1.04301055	0.060753754	0.59293425	1

P3h3	1.03370143	0.047819546	0.59312773	1
Nudt7	1.04237726	0.059877516	0.59314782	1
Slc25a19	1.05658698	0.079411537	0.59315749	1
Rab11fip4	0.96761441	-0.047495836	0.59316109	1
Tspyl1	0.97465521	-0.037036147	0.59323205	1
Fth1	1.02683687	0.038206999	0.59323568	1
Pmpca	1.03029441	0.043056654	0.59324428	1
Anxa11	0.97472356	-0.036934983	0.5932966	1
Zfp846	1.04457193	0.062911837	0.59331219	1
Mcfd2	1.03756457	0.053201127	0.59331923	1
Ccni	0.97577501	-0.03537956	0.59353723	1
Josd2	0.96288868	-0.054559072	0.59355935	1
Cbll1	1.03912061	0.055363116	0.59357238	1
Glrx3	1.02526519	0.035997125	0.59370808	1
Lhfpl1	0.95447185	-0.067225447	0.59375862	1
Bivm	1.0388461	0.054981945	0.59379356	1
Foxn3	0.95634712	-0.064393731	0.59382817	1
Ralgapa1	1.02524677	0.035971195	0.59395367	1
Pak3	1.04651213	0.065589038	0.5939555	1
Zfp523	1.04612008	0.065048463	0.5940012	1
Ppp1r14b	1.04452635	0.062848892	0.59401834	1
Pros1	0.96539242	-0.050812603	0.59402001	1
Jag1	0.96891179	-0.045562771	0.59410919	1
Padi4	1.22640712	0.294437976	0.59411112	1
Rcn2	0.97313739	-0.039284591	0.59415436	1
Psmd9	1.03817349	0.054047559	0.59423583	1
Srsf10	0.95713314	-0.063208477	0.5943058	1
Krtcap2	0.96638948	-0.049323344	0.59430924	1
Rela	1.03909585	0.055328746	0.59431385	1
Cdh7	1.0645388	0.09022853	0.59452686	1
Cltb	1.02516872	0.035861372	0.59455434	1
Gabra3	1.05038655	0.070920345	0.59461906	1
Hdgf	0.96937557	-0.044872376	0.59465019	1
Fkbp8	0.97572434	-0.035454477	0.59469542	1
Akna	1.0566344	0.079476284	0.5947048	1
Hectd2	1.04784789	0.06742931	0.59475149	1
Ccnt1	1.07549028	0.104994481	0.59479088	1
Map10	1.11306925	0.154543359	0.59502703	1
Slc25a11	0.96858548	-0.046048724	0.59507122	1
Cdc42	1.02482803	0.035381843	0.59512466	1

Fbxw11	0.97536298	-0.035988876	0.59515878	1
Faim	1.06144473	0.086029253	0.59523727	1
Myo1g	1.09167169	0.12653905	0.59528177	1
Bcar3	0.96216645	-0.055641599	0.59539934	1
Fzd9	1.13032087	0.17673238	0.59547486	1
Prkcsh	0.97445324	-0.037335131	0.59552312	1
Nudt9	0.96573562	-0.050299803	0.59575284	1
Abcc12	1.08248665	0.114349234	0.59588282	1
Ei24	0.97325528	-0.039109834	0.59588403	1
Polr2k	1.03398499	0.048215249	0.595928	1
Dph3	1.04313769	0.060929604	0.59595936	1
Tmtc3	0.96597926	-0.049935883	0.59605656	1
Yipf4	1.03005792	0.04272546	0.59605742	1
Pex16	1.04888875	0.068861664	0.59611912	1
LOC100294508	0.97281175	-0.039767447	0.59612855	1
Rhoq	1.02569582	0.03660295	0.59617793	1
Itgb5	1.02775353	0.039494329	0.59637257	1
Riok2	0.95747847	-0.062688042	0.59648609	1
Xrcc4	1.06513697	0.091038969	0.59650787	1
Slc35g1	0.89024139	-0.167731518	0.59664983	1
Mecr	0.93834254	-0.091813427	0.59672348	1
Snrpc	1.0503818	0.07091382	0.59673492	1
Zdhhc4	0.94412254	-0.082953968	0.59681519	1
Vnn1	0.86655232	-0.206641234	0.59691147	1
Scube3	0.90295751	-0.147270002	0.59718133	1
Nsun6	1.07737814	0.107524702	0.59720874	1
Timp1	0.96712769	-0.048221719	0.59721561	1
Rspo2	0.95663994	-0.063952065	0.59735552	1
Stard6	1.09651676	0.132927867	0.59737358	1
Calu	1.02449506	0.034913025	0.59763799	1
Oxct1	0.97615789	-0.034813573	0.59779363	1
Lgi1	1.02805131	0.039912273	0.59783039	1
Col2a1	0.94941539	-0.07488866	0.59808482	1
Zfp207	1.02743021	0.039040395	0.59825464	1
Ube2j1	0.97286053	-0.039695099	0.59832556	1
Specc1l	1.02785427	0.039635735	0.59845994	1
Cpeb4	0.96545404	-0.050720516	0.59859201	1
Usp8	1.02464947	0.035130458	0.59866812	1
Rtn4r	0.94103348	-0.087682041	0.59868707	1
Cntn6	1.07118275	0.099204627	0.59882548	1

Tmem143	0.94569517	-0.08055286	0.59885957	1
Plekha4	0.97580347	-0.035337487	0.5988925	1
Abcb9	0.9687504	-0.045803093	0.59896771	1
Paqr6	0.96042917	-0.05824887	0.59901112	1
Tmtc4	0.97081382	-0.042733449	0.59914276	1
Mtif3	0.94657693	-0.079208339	0.59924749	1
Scaf8	0.9683506	-0.046398606	0.59939635	1
Moap1	0.94970793	-0.074444192	0.59941182	1
Atp6v1b2	1.02506227	0.035711558	0.59941294	1
E4f1	1.05083737	0.071539409	0.59949214	1
Phactr3	0.95052481	-0.073203804	0.59953706	1
Tmem30b	0.94356494	-0.083806279	0.59957249	1
Lrrtm2	0.95174523	-0.071352663	0.59970501	1
Pdk1	1.05650486	0.07929941	0.59979691	1
Ccbl1	0.94780457	-0.077338479	0.59980101	1
Sfxn3	1.02542227	0.036218133	0.60015114	1
RGD1307461	0.96079978	-0.057692282	0.60019328	1
Kdm4d	1.10770088	0.147568349	0.60026047	1
Slc13a3	0.9618639	-0.056095323	0.60034802	1
Dynll2	0.97526625	-0.036131967	0.60047445	1
Cactin	1.04796953	0.067596773	0.60052796	1
Rasef	0.90818734	-0.138938169	0.60074659	1
Akr1e2	1.04412864	0.062299466	0.60092213	1
Grik4	1.04352392	0.061463674	0.60101672	1
Tceal1	1.03817939	0.054055753	0.60103539	1
Slc16a2	0.92498818	-0.112493167	0.6010419	1
Tnfaip6	1.12076719	0.164486623	0.60110175	1
Abhd13	1.04155413	0.05873782	0.60114943	1
Ubap1	1.03112454	0.04421859	0.6012357	1
Cnbp	1.02429979	0.034638021	0.60146026	1
Mxd1	0.94634044	-0.07956882	0.60146407	1
Kcnh2	0.97425053	-0.037635282	0.60149036	1
Rad23b	1.02426963	0.034595541	0.60163341	1
Taok3	0.95808288	-0.061777631	0.60165558	1
Cd84	1.09081881	0.125411485	0.60180013	1
Cdc14a	0.95866428	-0.06090241	0.60185559	1
Vegfa	0.97343751	-0.038839724	0.60197254	1
Rnmt	1.03467444	0.049176893	0.60201638	1
Papd4	1.04934351	0.069487038	0.60202141	1
Lmo7	0.96391572	-0.053021086	0.6020625	1

Cdk1	1.12265958	0.166920531	0.60216422	1
Faf1	1.03243247	0.046047424	0.60227571	1
Chordc1	0.97485274	-0.036743785	0.60228924	1
Ggnbp2	1.02574716	0.036675158	0.60231547	1
Baz2b	1.03443104	0.048837474	0.60241934	1
Vsig10	0.94874703	-0.075904626	0.60243485	1
Sox11	0.8853646	-0.175656407	0.60243704	1
Rsad1	0.90056707	-0.151094366	0.6024561	1
Tram1	1.02823343	0.040167824	0.60247194	1
Lin7a	0.92320924	-0.115270435	0.60261322	1
Hspd1	1.02421109	0.03451308	0.60271342	1
Spata24	1.10863272	0.148781489	0.60279148	1
Fbxo16	1.07227239	0.100671441	0.60281091	1
Nol3	0.96584121	-0.050142073	0.60284352	1
Tox	0.93942727	-0.090146617	0.60286078	1
Efemp2	0.95760664	-0.062494936	0.60287149	1
Trappc5	0.95397953	-0.067969781	0.60292982	1
Rpl28	0.97529285	-0.03609262	0.60293412	1
Elac2	0.96436419	-0.052350022	0.60297327	1
RGD1564450	1.04106727	0.058063298	0.60314708	1
Napg	1.02427549	0.03460379	0.60314979	1
Farp2	1.03458342	0.049049974	0.60319137	1
Mapk8	1.02604159	0.037089218	0.60332931	1
Cct4	1.024075	0.034321376	0.60336362	1
Morn1	1.08020402	0.111303819	0.60348527	1
Vapa	1.02407671	0.034323793	0.60353484	1
Tex264	0.96963156	-0.04449144	0.60372895	1
Tcea2	1.03193989	0.045358932	0.60380969	1
Aip	0.96865522	-0.04594484	0.60381146	1
Pgm2l1	0.97600668	-0.035037067	0.60418727	1
Tspyl4	1.02424065	0.03455472	0.60422879	1
Shfm1	0.96753228	-0.047618297	0.60426863	1
Tmem35	0.97570597	-0.03548164	0.60430973	1
St13	1.02402372	0.034249138	0.60432045	1
Rnaseh1	1.05644167	0.079213118	0.60453018	1
Zfp110	1.03229092	0.045849613	0.60455211	1
Smc3	1.02789452	0.039692222	0.6046224	1
Ubqln4	0.97064311	-0.042987165	0.60463716	1
Tspan17	0.97652605	-0.034269559	0.60464887	1
Slc39a1	1.03184064	0.045220171	0.60465171	1

Cnnm3	1.04082025	0.057720937	0.60469742	1
Skiv2l2	0.97221892	-0.040646882	0.60483014	1
Ncapd3	1.05055825	0.071156151	0.6050181	1
Acbd6	1.04544534	0.064117628	0.60504117	1
Socs3	0.90027885	-0.151556162	0.60505693	1
Arhgef11	0.97416097	-0.037767914	0.60507675	1
Lrrc71	1.08715299	0.120554982	0.60513759	1
Chn1	0.97454336	-0.037201711	0.60527559	1
Slitrk3	1.02819579	0.040115009	0.60527853	1
Lrrc42	1.04297085	0.060698834	0.60531834	1
Ift22	1.05006837	0.070483267	0.60532879	1
Elac1	1.06298566	0.088122136	0.60535803	1
Grxcr2	0.92082358	-0.119003317	0.6053709	1
Lmnb2	0.93248548	-0.100846837	0.60544317	1
Hdac1	1.03843584	0.054412077	0.60559394	1
Ufd1l	1.02777075	0.039518496	0.60563542	1
Thap7	0.95538458	-0.065846509	0.60569696	1
Sars	1.02391146	0.034090968	0.60570514	1
Fkbp10	0.95822037	-0.061570606	0.60584808	1
Armc9	1.03434744	0.048720879	0.60591036	1
Otud5	0.97307183	-0.039381795	0.60598917	1
Ica1	1.03102324	0.044076849	0.60604023	1
Zfp36l2	1.03010123	0.042786115	0.6060626	1
Cckar	1.08000145	0.111033245	0.60620417	1
Trrap	0.97550126	-0.035784356	0.60626732	1
Man2a2	0.96834582	-0.046405728	0.60628036	1
Pin1	1.02477185	0.035302756	0.60647412	1
Akap6	0.97603884	-0.03498953	0.60648262	1
Arf3	0.97652321	-0.034273764	0.60659099	1
Dlg1	0.96273483	-0.054789611	0.60670433	1
Tcf19	0.92398749	-0.11405477	0.60684784	1
Grem2	0.97038481	-0.043371125	0.60685327	1
Atg3	0.97147529	-0.041750791	0.60697567	1
Cant1	1.03436685	0.048747939	0.60714096	1
Mrps26	0.96248662	-0.055161604	0.60731769	1
Egfl8	0.97468739	-0.036988511	0.60734964	1
Pde4d	0.95435504	-0.067402011	0.60735621	1
Prkar2a	0.96512035	-0.051219235	0.60735648	1
Llph	1.04491154	0.063380818	0.60744526	1
Car8	0.94515363	-0.08137925	0.60747531	1

Tm7sf3	0.96504982	-0.051324669	0.60756371	1
Utp3	1.03369145	0.047805613	0.60759445	1
Tmem214	0.96759931	-0.047518348	0.60774589	1
Smarca5	0.97465877	-0.037030882	0.60775381	1
Alk	0.95969346	-0.059354438	0.6078475	1
Tmem198b	1.0676914	0.094494717	0.60790332	1
Timm10b	1.0566787	0.07953677	0.60793222	1
LOC361635	1.02806994	0.039938421	0.60793761	1
Jdp2	1.07599591	0.105672599	0.60795598	1
Dnaja1	0.97628423	-0.034626863	0.60812462	1
Etv3	1.06227505	0.087157361	0.60831872	1
Gpr27	0.89355819	-0.16236641	0.60844607	1
Fra10ac1	1.04371426	0.061726789	0.60863712	1
Lrrc36	1.10552149	0.144727066	0.60882374	1
Xkr7	0.92977617	-0.105044644	0.60887363	1
Pet100	1.04451544	0.062833814	0.60888949	1
Bcl2l1	0.97061438	-0.043029859	0.60893845	1
Lrrc49	1.02374588	0.033857645	0.60901458	1
Adgrb3	0.96725675	-0.048029207	0.60901625	1
Itgbl1	1.02920507	0.04153047	0.60917034	1
Tomm34	1.02490206	0.035486046	0.60919674	1
Wiz	0.96510133	-0.051247664	0.60925244	1
Dok6	0.93836149	-0.091784294	0.60931666	1
Rps3	1.02396385	0.034164782	0.60935811	1
Fgd1	0.94130221	-0.087270118	0.6094888	1
Slc36a1	1.03154612	0.044808331	0.60956069	1
Zfp566	1.11034047	0.151002126	0.60959565	1
Rps16	1.02368344	0.033769658	0.60966037	1
Cpne3	0.97091767	-0.042579127	0.6097217	1
Glmn	1.04322133	0.061045276	0.60973044	1
Wbp1l	0.97154005	-0.041654628	0.60975205	1
Itga1	1.05132194	0.072204521	0.60982336	1
Ust	0.9587986	-0.060700291	0.60983899	1
N6amt1	1.07521319	0.104622743	0.60986569	1
Otud4	1.03756871	0.053206875	0.60991836	1
Ptch2	1.06092726	0.085325743	0.60996976	1
Parm1	1.02471856	0.03522773	0.61018534	1
Cops2	1.02369697	0.033788716	0.61031164	1
Lrig2	0.96691442	-0.048539885	0.6103918	1
Cdc42ep5	1.10013554	0.137681275	0.61042906	1

Rab21	0.97015024	-0.043719908	0.61052466	1
Fam214b	0.96301297	-0.054372862	0.61057406	1
Med8	0.91575067	-0.126973241	0.61062707	1
Ror1	0.92857591	-0.106908246	0.61110655	1
Nrsn2	1.08883907	0.122790735	0.61113297	1
Hmces	1.04119259	0.058236954	0.61119018	1
Car2	1.02512711	0.035802802	0.6112248	1
Flnc	0.95425415	-0.06755454	0.61124606	1
Ahctf1	0.97135763	-0.041925541	0.61132268	1
LOC100910996	1.0434022	0.061295384	0.61134499	1
Iqub	0.91564866	-0.127133956	0.61140514	1
Ndst3	1.06123835	0.085748714	0.61141026	1
Mrps34	1.04348867	0.061414936	0.61163289	1
Ddx3	1.02621434	0.037332091	0.61165324	1
Pigh	0.94586679	-0.080291073	0.61193064	1
Dohh	1.03711275	0.052572752	0.61194638	1
Trnau1ap	1.05618752	0.078866001	0.61194659	1
Leprotl1	1.03588968	0.050870374	0.61205583	1
Igfbp7	1.02353742	0.033563849	0.61213888	1
Cdk8	0.96497267	-0.051440005	0.6121496	1
Spata6	0.96016718	-0.058642468	0.61221417	1
Rpl36	0.96872631	-0.045838965	0.61226554	1
Med7	1.04793668	0.067551553	0.61227535	1
Cd81	1.02437258	0.034740542	0.61228229	1
Ift52	1.03155716	0.044823764	0.61232821	1
Arg2	1.04303938	0.060793632	0.61234609	1
Nrn1	1.02371326	0.033811677	0.61256244	1
Slc9a9	1.0234894	0.033496161	0.61258048	1
Pttg1	1.14608344	0.196712085	0.61275866	1
Gstm1	0.97185088	-0.04119313	0.61284528	1
Rala	1.03453293	0.048979569	0.61284931	1
Brca2	0.94414645	-0.082917438	0.61290834	1
Fzd3	1.05547402	0.077891072	0.61300897	1
Mpdu1	1.02922169	0.041553769	0.61316777	1
Nlrp1a	1.07415833	0.10320666	0.61341525	1
Tspan5	0.96831751	-0.046447916	0.61342546	1
Htr3a	0.97602897	-0.035004121	0.61349922	1
Hnrnpf	0.97667485	-0.034049742	0.61351448	1
Cars	0.96562435	-0.050466042	0.61363744	1
Man2b2	0.96456402	-0.052051105	0.61373581	1

Tmem204	1.05761037	0.080808221	0.61374103	1
Eif2b4	1.03644139	0.051638538	0.61379518	1
Phldb3	1.1254353	0.170483115	0.61392007	1
Klhl5	0.96760074	-0.047516229	0.61395728	1
Nek9	1.02395231	0.034148531	0.61423161	1
Cinp	0.96218406	-0.055615193	0.61454393	1
Rpl39	0.9761294	-0.034855687	0.61463633	1
Pex6	0.95894291	-0.060483171	0.61464009	1
Eif2s1	0.97218958	-0.040690416	0.61469954	1
Ankrd44	0.95906275	-0.060302881	0.61471603	1
Pnn	1.02689026	0.038282018	0.61477551	1
RGD1311164	1.04484509	0.063289059	0.61478183	1
Pdzd4	1.0379798	0.053778368	0.614812	1
Arhgef3	1.03206579	0.045534946	0.6149096	1
Nudt6	0.90709121	-0.140680464	0.61493582	1
Armc8	0.97163016	-0.041520828	0.61494309	1
Wdr6	0.97600852	-0.035034357	0.61502342	1
Grb10	0.9709724	-0.042497811	0.61510568	1
Slc10a7	1.06141806	0.085993007	0.61522888	1
Cdk9	0.9708875	-0.042623958	0.61569674	1
Atl3	1.03178432	0.045141421	0.61570908	1
Rxrb	0.96590313	-0.050049584	0.61570937	1
Gstz1	1.06185259	0.086583506	0.61584797	1
Bcl2	0.92217489	-0.116887709	0.61591447	1
Rad54l	1.14320689	0.193086518	0.61593503	1
Aoc2-ps1	0.90968129	-0.136566916	0.61596059	1
Rps6kb2	1.0474171	0.06683607	0.61601719	1
Abcg3	1.07674273	0.106673582	0.61606438	1
Syndig1l	0.94794791	-0.077120313	0.6161252	1
Stpg1	0.91120575	-0.134151249	0.61614862	1
Ubl7	0.97044659	-0.043279285	0.61626893	1
Phkg2	1.02965487	0.042160848	0.61629073	1
Srsf3	1.02414305	0.034417237	0.61640214	1
Slc26a6	1.06027418	0.084437382	0.61644207	1
Sec24d	0.96375937	-0.053255112	0.61646825	1
Camk1	1.03777373	0.053491925	0.61646988	1
Pop7	1.04461519	0.062971589	0.61647524	1
Fam109b	1.10900096	0.149260618	0.61651067	1
Ccdc92	0.97720408	-0.033268209	0.61670621	1
Lars2	1.04380253	0.061848805	0.61671076	1

Ndufa5	0.97327854	-0.039075351	0.61674926	1
Bmp7	1.06185168	0.086582259	0.61691777	1
Ppib	0.97600443	-0.035040402	0.61698705	1
Atp2b4	0.96352109	-0.053611848	0.6172613	1
Rbp2	0.88427701	-0.17742972	0.61728375	1
Slc19a2	1.03524998	0.049979171	0.61738876	1
MGC94335	0.95553728	-0.065615929	0.61742587	1
Sphk2	0.97337984	-0.038925194	0.61793739	1
Fancd2	1.08079528	0.11209328	0.61802396	1
Qdpr	1.02391418	0.034094806	0.61802437	1
Sumo2	1.02409699	0.034352353	0.6180472	1
Grwd1	0.95028858	-0.07356241	0.6180814	1
Rab3d	1.02886365	0.041051808	0.61819205	1
Spopl	0.95314856	-0.069226996	0.61820966	1
G6pc3	0.97312341	-0.039305319	0.6183665	1
Yipf2	1.03829014	0.054209645	0.61841593	1
Opn4	1.1114066	0.152386707	0.61848455	1
Gcnt2	1.02652979	0.037775489	0.61854842	1
Surf1	1.04118466	0.058225957	0.61876203	1
Thbs3	1.05577296	0.078299627	0.61883549	1
Plekhh3	0.94854616	-0.076210108	0.61894165	1
Ap1b1	1.02371978	0.03382087	0.61907604	1
Tfap2a	1.11661131	0.15912707	0.61915255	1
Zfp710	1.06449496	0.090169124	0.61915419	1
Hccs	1.03556785	0.050422081	0.6192113	1
Hcn4	0.94545559	-0.080918402	0.61929181	1
Stap1	1.14552532	0.196009352	0.61931361	1
Sass6	1.10130787	0.13921783	0.61935643	1
Rlbp1	0.89254719	-0.163999641	0.61942445	1
Efr3a	1.02312161	0.032977632	0.61948909	1
Catsper2	1.07422511	0.103296355	0.61959065	1
Ciao1	1.03037159	0.043164721	0.61968535	1
Dmtf1	0.97465291	-0.037039557	0.6197187	1
Srek1	1.04011957	0.056749385	0.61972366	1
Cnksr2	1.05127162	0.072135471	0.61972579	1
Zc3h14	0.97388	-0.038184085	0.61977649	1
Ly86	1.08660893	0.119832813	0.61979152	1
Akirin1	0.97652167	-0.034276038	0.61980801	1
Ralgapb	0.97753204	-0.032784099	0.61992148	1
Ntsr1	0.93313405	-0.09984375	0.62021224	1

Adcy2	1.0399662	0.056536642	0.62022699	1
Naa60	0.97176681	-0.04131793	0.62023641	1
Ehd2	0.96710592	-0.048254191	0.62033251	1
Rhoh	0.88935835	-0.169163247	0.6204978	1
Gas7	0.97755955	-0.032743505	0.6205109	1
Ptcd2	1.04764945	0.067156068	0.62051154	1
Cd8a	0.89697111	-0.156866584	0.62053685	1
Ppp1r14a	1.0884172	0.122231666	0.62059313	1
Rassf1	0.95561003	-0.065506101	0.62064907	1
Lat2	0.85206816	-0.230959245	0.62067034	1
Slc7a7	0.97218254	-0.040700864	0.62068524	1
Tnrc6a	1.02490617	0.035491839	0.62068716	1
Ikbip	1.04973847	0.070029938	0.62072577	1
Chodl	0.93768395	-0.092826358	0.62076179	1
B4galt7	0.96154367	-0.056575713	0.62077226	1
Fcho2	0.96256926	-0.055037737	0.62091481	1
LOC100363521	1.03484504	0.049414751	0.62095944	1
LOC500475	1.08630472	0.119428851	0.62099265	1
Tmx2	1.02294861	0.032733677	0.62102525	1
Insc	0.95329554	-0.069004546	0.62119397	1
Prkaa1	1.02366956	0.033750094	0.62128799	1
Arhgap4	0.94279919	-0.084977577	0.6213329	1
Hoxb5	0.93264238	-0.100604108	0.62134045	1
Cul4a	1.02744493	0.039061066	0.6213544	1
Itga10	1.12262991	0.1668824	0.62136727	1
Fermt2	0.97763838	-0.032627174	0.62144792	1
Lhfpl4	0.96769185	-0.047380386	0.62146348	1
Eif4ebp3	1.07776765	0.108046183	0.62146378	1
Rnf103	0.97331444	-0.039022134	0.62151306	1
Srxn1	0.97046683	-0.043249197	0.62152552	1
Kazald1	0.91777551	-0.123786789	0.6216722	1
Abcd1	1.05764151	0.08085071	0.62169511	1
LOC100365289	1.04615945	0.065102762	0.62169887	1
Sart3	1.03846172	0.054448041	0.62178695	1
Scarf1	0.91615167	-0.126341636	0.62184723	1
Gatm	0.97266121	-0.039990718	0.62186576	1
Tmbim6	0.97514051	-0.036317976	0.62189848	1
Faap24	1.0971427	0.133751188	0.62193269	1
Psmg4	0.94033426	-0.088754408	0.62195817	1
Kcnj10	0.96590806	-0.050042221	0.62198873	1

Fkbp11	1.09095163	0.125587144	0.6220094	1
LOC497899	0.91832153	-0.122928722	0.62202747	1
Phpt1	1.03578992	0.050731417	0.62215559	1
Grk6	0.96840707	-0.046314488	0.6221924	1
Eed	1.04072154	0.057584108	0.62223999	1
Tmem266	1.11139661	0.15237374	0.62235599	1
Hspa14	0.95930322	-0.059941192	0.62254622	1
Anks3	1.04418508	0.062377452	0.62256674	1
Calhm2	0.94524036	-0.081246869	0.62262109	1
Ptgis	1.06498856	0.09083793	0.62271796	1
Necab2	0.92249174	-0.116392097	0.62296314	1
Hexb	1.03135993	0.044547901	0.62312816	1
Asmtl	0.95781085	-0.062187316	0.6232443	1
Parp6	1.02344414	0.033432359	0.6233598	1
Slc25a29	1.06011861	0.084225686	0.62349972	1
Aasdhppt	0.96817766	-0.046656286	0.62379668	1
Snupn	0.95599082	-0.064931333	0.6238354	1
Ubxn4	1.02399205	0.03420451	0.62400114	1
Ankrd23	1.15394635	0.206576154	0.62404893	1
Cog6	1.03795654	0.053746042	0.62412602	1
Uri1	0.96490243	-0.051545029	0.62425762	1
Zfp143	1.04656833	0.065666503	0.62435942	1
Zfp36	1.03329937	0.047258294	0.62446517	1
Dbt	1.03145269	0.044677656	0.62447233	1
Nr3c1	0.97605135	-0.034971042	0.62448601	1
Dnmt3a	1.05288993	0.074354625	0.62458482	1
Irf6	1.03375094	0.047888646	0.62460765	1
Stx6	0.97040355	-0.04334326	0.62464881	1
Mmp9	0.90160978	-0.149424933	0.62466579	1
Fcf1	1.03612417	0.051196902	0.6247406	1
Guf1	1.03310887	0.046992295	0.62477423	1
Mtap	1.0620673	0.086875182	0.62517324	1
Ino80	0.96715592	-0.048179604	0.62521223	1
Tmem243	1.06116485	0.085648797	0.6253248	1
Mef2a	0.9730086	-0.039475541	0.62538066	1
Mfn2	0.97791144	-0.03222427	0.62545905	1
Wfs1	0.97479089	-0.036835326	0.62551094	1
Bmp15	0.87242602	-0.196895291	0.62563603	1
Utp14a	0.96092078	-0.057510601	0.62580293	1
Bop1	1.03680238	0.052140938	0.62625673	1

Trim5	1.0394439	0.055811897	0.6264176	1
Epm2a	0.92819043	-0.107507265	0.62642547	1
Rps6ka6	0.93366951	-0.099016122	0.62651099	1
Lrp2	0.92987111	-0.104897333	0.62651597	1
Polr3g	0.97737379	-0.033017681	0.62679357	1
Matk	0.95450112	-0.067181197	0.62683615	1
Cfap52	0.88155397	-0.181879198	0.62709109	1
Hist2h2aa3	0.95154267	-0.071659739	0.62711107	1
Bag1	1.02486608	0.035435401	0.62716681	1
Fam50a	1.03973246	0.056212348	0.62718429	1
Gprc5c	1.04016837	0.056817069	0.62719048	1
Camp	1.09039646	0.124852785	0.62719969	1
RGD1565616	1.02783491	0.03960856	0.62721243	1
Sel1l	0.97685495	-0.033783734	0.62725327	1
Taf5l	0.95246797	-0.070257512	0.62727158	1
Fpr1	1.32438647	0.405324179	0.62728069	1
Ceacam4	1.14350608	0.19346404	0.62737873	1
Cops7b	1.04230417	0.059776356	0.62741842	1
Grb14	0.96550745	-0.050640706	0.62746632	1
Mrrf	1.02969108	0.042211582	0.62757498	1
Lsm6	1.04633623	0.065346526	0.62763217	1
Galc	0.94935556	-0.074979574	0.627726	1
Rfxank	1.06666052	0.09310109	0.62784277	1
Dxo	0.93088509	-0.103325002	0.62791671	1
Rabgap1	0.97682734	-0.033824509	0.62792767	1
Cd37	1.06537641	0.091363245	0.62796982	1
Trappc10	0.97645607	-0.034372958	0.62798865	1
Zfp503	0.95400741	-0.067927623	0.6280408	1
Bsg	1.02293051	0.032708149	0.62832761	1
Cers5	1.02908045	0.041355769	0.62847501	1
Parg	1.03214529	0.045646069	0.62852652	1
Ddx49	0.96001349	-0.058873415	0.62858828	1
Med14	1.02849785	0.040538783	0.62864246	1
RGD1309730	1.03880457	0.054924269	0.62867762	1
Twist2	0.8843007	-0.177391065	0.6288096	1
S100a4	0.97375341	-0.038371626	0.62897387	1
Fbxw9	1.06340142	0.088686292	0.62903869	1
Vps37d	0.91302604	-0.131272081	0.6291364	1
Smurf2	1.03004519	0.042707634	0.62928746	1
Amz2	0.97529667	-0.036086971	0.629344	1

Pusl1	0.95484737	-0.066657956	0.62943809	1
Gstt1	1.07967233	0.110593531	0.62962716	1
Trpv3	1.08680828	0.120097462	0.62963847	1
Dsel	0.96549676	-0.05065668	0.62969418	1
Tinf2	1.04284801	0.06052891	0.62974724	1
Agpat5	0.96899519	-0.04543859	0.62975797	1
Wdfy1	0.96421383	-0.052574977	0.62981027	1
Blvra	1.03485988	0.049435434	0.62988017	1
Limk2	1.0363747	0.051545706	0.62993076	1
Nrxn3	1.03439742	0.048790582	0.6300834	1
Lamtor5	1.03415828	0.048457006	0.6301171	1
Pdia3	0.97786964	-0.032285944	0.6301903	1
Idh3a	0.97808283	-0.031971455	0.63023974	1
Nat10	1.04192722	0.059254513	0.63037505	1
Wbscr17	0.96608858	-0.049772618	0.63041171	1
Wdr91	1.04011539	0.056743588	0.63043137	1
Pgpep1	1.04654056	0.065628221	0.63043363	1
Rbpj	1.0294174	0.041828072	0.63045348	1
Lsm3	0.95859197	-0.061011248	0.63046429	1
Il2rb	0.87547289	-0.191865598	0.63056391	1
Inpp5k	1.03185869	0.045245413	0.6310364	1
Wipi1	0.96332159	-0.053910588	0.63107072	1
Sox9	1.10688116	0.146500337	0.63130583	1
Ngb	0.92260288	-0.1162183	0.63135171	1
Tmem115	1.03393792	0.048149569	0.63137865	1
Agbl5	1.07822349	0.108656242	0.63142823	1
Nfkb1	0.97048372	-0.043224077	0.63165832	1
Dusp4	0.91007189	-0.135947584	0.6316625	1
Inhbb	0.956826	-0.063671505	0.6317806	1
Sat2	0.95522614	-0.066085774	0.63186767	1
RGD1562114	1.06876633	0.095946457	0.63193366	1
Mmp14	1.03015005	0.04285449	0.63199889	1
Scn4b	0.97773593	-0.032483227	0.63208085	1
Cdkl3	1.07860318	0.109164192	0.63210772	1
Agtpbp1	1.02271194	0.032399852	0.63211229	1
Nadk	1.02943401	0.041851354	0.63218403	1
Rae1	0.96172277	-0.056307017	0.63228642	1
Cacfd1	1.06103872	0.085477307	0.63251064	1
Gkap1	1.03811446	0.053965515	0.63254826	1
Nup62	1.0364589	0.051662904	0.63272776	1

Ptges2		1.03735927	0.052915628	0.63285509	1
Mfsd11		0.9693344	-0.044933637	0.63299858	1
Clmp		0.97695712	-0.033632856	0.63301203	1
Ran		1.02208804	0.031519466	0.63301697	1
G6pd		1.02354152	0.033569621	0.63304398	1
Plgrkt		1.04235053	0.059840523	0.63316215	1
Ripk1		1.04143305	0.058570099	0.63351699	1
Uchl3		1.04237763	0.059878022	0.63358479	1
Macf1		0.97828473	-0.031673668	0.63370336	1
Eef1a2		0.97791208	-0.032223331	0.63374877	1
Cdc14b		1.05798604	0.081320589	0.63377979	1
Eef2		0.97727853	-0.033158291	0.63392054	1
Metap1d		0.93030584	-0.104223006	0.63395152	1
Ablim3		0.96623293	-0.049557068	0.63411631	1
Psme2		0.95382622	-0.068201659	0.6341711	1
Dad1		1.02494466	0.035546009	0.63442337	1
Rps15a		0.9723298	-0.040482359	0.63453167	1
Sptbn2		0.97824173	-0.031737081	0.63460573	1
Nabp1		0.9361129	-0.095245562	0.63463675	1
Inip		0.95930047	-0.059945324	0.63464072	1
Shh		1.03678218	0.052112824	0.6347992	1
Sgcd		0.9373956	-0.093270071	0.6349035	1
Tfeb		1.0570156	0.079996662	0.63495199	1
Gprc5a		0.86798705	-0.204254581	0.63503743	1
	10-Sep	1.03504287	0.049690522	0.63525176	1
Tmem56		1.03752253	0.053142663	0.63528287	1
Sec23b		1.0364644	0.051670571	0.63534824	1
Chga		1.02236337	0.031908046	0.63539334	1
Dnm3		0.97829079	-0.031664737	0.63540263	1
Tdrd3		0.96239977	-0.055291797	0.63547799	1
LOC688090		1.04910538	0.069159603	0.63553464	1
Ptpn13		0.97427495	-0.03759912	0.63566103	1
Clk3		0.96988074	-0.044120738	0.63569138	1
Msto1		1.03853572	0.054550841	0.63574558	1
Glul		0.97824769	-0.031728299	0.63586281	1
Abhd17c		0.95660088	-0.064010978	0.6359819	1
Oscp1		1.04518129	0.063753205	0.63629349	1
Mcemp1		1.15311796	0.205540105	0.63649548	1
Zfp24		0.96355282	-0.053564343	0.63655314	1
Hspb8		1.02191214	0.031271167	0.63682865	1

Cd151	1.02201465	0.03141587	0.63698576	1
Igf2bp2	0.90313852	-0.146980817	0.63701996	1
Zmym6nb	0.90771486	-0.139688915	0.63703709	1
Matr3	1.02197903	0.03136559	0.63711562	1
Tenm4	0.95568955	-0.065386051	0.63714217	1
Gid8	1.03212003	0.045610758	0.63716104	1
Arhgap8	0.92958643	-0.105339091	0.63728585	1
Jagn1	0.9698506	-0.044165565	0.63731622	1
Ndfip2	0.97863818	-0.031152526	0.63733387	1
Veph1	1.115195	0.157295999	0.63735983	1
Cd48	0.97868761	-0.031079658	0.63748267	1
Kat8	1.0345457	0.048997381	0.63750363	1
Arhgap11a	0.93808007	-0.09221703	0.63753374	1
Fdx1l	1.03776165	0.053475125	0.63753868	1
Ssx2ip	0.97309532	-0.039346967	0.63756379	1
Fh	1.02478413	0.035320041	0.63772205	1
Klhl25	0.94413411	-0.082936293	0.6378093	1
Tspan13	0.97863744	-0.031153613	0.63827364	1
Ubl5	1.03073073	0.04366749	0.63831604	1
Adh1	0.89100547	-0.166493806	0.6383603	1
Mllt1	1.0393681	0.055706685	0.63872486	1
Manba	1.04647979	0.065544449	0.63881276	1
Psmb1	1.02232312	0.031851255	0.63883587	1
Ints10	1.0323914	0.045990029	0.63904387	1
Ube2l6	1.03983702	0.056357425	0.63905656	1
Pag1	0.91867898	-0.122367272	0.63909063	1
Cdk5	0.97617365	-0.034790289	0.63919073	1
Lcp2	1.05508203	0.077355176	0.6392581	1
Wdr24	1.03578392	0.050723067	0.63932492	1
Gfm2	0.96342694	-0.053752828	0.63933517	1
Mylpf	0.90628054	-0.141970388	0.63954987	1
Gbas	1.03159058	0.044870503	0.63970887	1
Lyar	1.04654912	0.065640028	0.63976292	1
RGD1308134	1.05198899	0.073119607	0.63982965	1
Zfp706	0.97089498	-0.042612841	0.63988808	1
Cox7a2	0.97584502	-0.035276047	0.63996789	1
Fam212a	1.08621728	0.119312722	0.64000171	1
Spata33	1.07244339	0.100901494	0.64005821	1
Pfdn5	0.97423975	-0.037651244	0.6400864	1
Eif2ak3	0.97107104	-0.042351252	0.64010247	1

Vhl	0.97050407	-0.043193831	0.64012446	1
Eya3	0.97290033	-0.039636085	0.6401565	1
Slc2a8	1.03309343	0.046970738	0.64017622	1
Zfp507	1.04054305	0.057336649	0.64031912	1
Foxk1	0.92973252	-0.10511237	0.64043313	1
Clec10a	1.0416584	0.058882234	0.64045664	1
Prrg1	1.04357072	0.061528367	0.64053544	1
Mboat7	0.96137846	-0.056823616	0.6406149	1
Mboat7l1	0.96137846	-0.056823616	0.6406149	1
Wdr5b	1.07998397	0.111009894	0.64076302	1
Clstn2	0.94965069	-0.074531155	0.64096136	1
Trim41	0.96947105	-0.044730282	0.64108075	1
Trappc2	1.02193167	0.031298731	0.64120536	1
Tbx2	0.95291605	-0.069578976	0.64130251	1
Rab26	1.09284098	0.128083484	0.64137207	1
Cyp4f5	1.0636098	0.088968973	0.64160041	1
Rock1	0.97312443	-0.039303807	0.64164349	1
Lpin3	0.93906474	-0.090703473	0.64170269	1
Nenf	1.03600337	0.0510287	0.64194273	1
Ap5s1	0.92264061	-0.116159295	0.64200225	1
Rap1gds1	0.97832446	-0.031615081	0.64213458	1
Bsn	0.97385606	-0.038219549	0.64215533	1
Ppfia4	0.96956773	-0.044586412	0.64215582	1
Tnfsf13	0.9440409	-0.083078724	0.64221761	1
Ciita	0.91939405	-0.121244758	0.64232536	1
Wipi2	0.96687126	-0.048604285	0.64234273	1
Cth	1.08328538	0.115413356	0.64239149	1
Slc15a2	1.04034072	0.057056099	0.64239484	1
Tstd2	1.04736328	0.066761931	0.64242084	1
Fbxo32	0.90375845	-0.145990863	0.64265228	1
Tmem179	1.02238624	0.031940322	0.64266279	1
Gal3st1	1.05611588	0.078768141	0.64268963	1
Twistnb	1.04316239	0.060963757	0.6426916	1
Fam83h	0.95386226	-0.068147149	0.64279642	1
Tsc22d4	0.97079192	-0.042765991	0.64287473	1
Crlf3	0.9689228	-0.045546371	0.64292058	1
Pepd	0.97365119	-0.038523071	0.64333145	1
Pafah1b2	1.02184021	0.031169613	0.64345467	1
Bad	1.03820567	0.054092275	0.64358287	1
Wnt2b	1.07372028	0.102618201	0.64366343	1

11-Sep	1.02882106	0.040992081	0.64383357	1
Jmjd8	0.96763697	-0.047462209	0.64400051	1
Notch2	0.97387327	-0.038194055	0.64400994	1
Kcnj2	1.11036971	0.151040124	0.64412246	1
Map3k14	1.06483096	0.090624419	0.64415675	1
Tnfaip1	1.02332992	0.033271339	0.64418361	1
Tars	0.97453669	-0.037211588	0.64419183	1
Ercc6	0.96342938	-0.053749179	0.64425957	1
Rbck1	1.03003538	0.042693894	0.64428836	1
Wdr1	0.97909781	-0.030475107	0.64430448	1
Slbp	1.02898976	0.041228625	0.64431171	1
Apaf1	1.04512461	0.063674959	0.64435935	1
Sipa1l1	0.97356615	-0.038649086	0.6444044	1
Dennd6b	0.95279763	-0.069758273	0.64441132	1
Slc25a4	1.02221899	0.031704304	0.64451301	1
Tep1	0.97300832	-0.039475946	0.644536	1
Ahi1	0.96750435	-0.047659949	0.64476878	1
Pf4	1.09610783	0.132389732	0.64479605	1
Bmp3	1.12166995	0.165648228	0.64486391	1
Lpcat2	1.04496351	0.063452563	0.64488091	1
Nradd	0.90121511	-0.150056592	0.64493752	1
Kars	1.02300519	0.032813468	0.64506757	1
Tmem98	1.05854336	0.082080366	0.6450894	1
Slc39a8	1.03854112	0.054558344	0.64546228	1
Ercc6l	0.88327872	-0.179059334	0.64547055	1
Nrbp1	1.02286865	0.032620895	0.64551007	1
Sigirr	1.10049977	0.138158847	0.6455353	1
Spred1	1.04202165	0.059385253	0.64579532	1
Gimap5	0.924129	-0.113833841	0.64579836	1
Thsd7b	1.04069541	0.057547878	0.64599014	1
Sppl2a	0.95877771	-0.060731723	0.64612889	1
Actr2	0.97912715	-0.030431872	0.64615088	1
Rnf187	0.9790017	-0.030616726	0.64618773	1
Nemp1	1.09831989	0.135298313	0.64623328	1
Pcmtd1	0.97833478	-0.031599863	0.64627199	1
Epb41l3	1.02234209	0.031878018	0.64630391	1
Atp6v0d1	1.02146445	0.030638991	0.64637118	1
Ikbkb	1.03421829	0.048540718	0.64637653	1
Pea15	0.97840063	-0.031502758	0.64639556	1
Sfxn4	1.04526439	0.063867905	0.64669861	1

Tprkb	0.95872783	-0.060806786	0.64676004	1
Abca1	0.97609168	-0.034911434	0.64679185	1
Atg10	1.0696604	0.09715284	0.64682109	1
Ocrl	1.02912998	0.041425207	0.64692027	1
Lancl1	0.97784375	-0.032324138	0.64699502	1
Trit1	0.96138626	-0.056811913	0.64707863	1
Ldah	0.96919201	-0.045145585	0.64708694	1
Negr1	1.05795671	0.081280591	0.64723511	1
LOC100909539	0.93492763	-0.097073393	0.64740938	1
Cyp2u1	0.9485324	-0.076231045	0.64754239	1
Hcn3	0.95003759	-0.0739435	0.64759411	1
Popdc2	0.90797364	-0.139277679	0.64759899	1
Srp9	1.02671235	0.038032049	0.64766876	1
Imp3	1.03860396	0.054645635	0.64772185	1
Tom1l1	0.96517429	-0.051138604	0.64773629	1
Gpalpp1	0.95849875	-0.061151548	0.6477818	1
Nup37	1.05394854	0.075804431	0.64798308	1
Bach2	1.06467007	0.090406427	0.64799494	1
Dtx2	1.05016205	0.070611964	0.64804009	1
Dnajb3	0.92993506	-0.10479812	0.64811675	1
RGD1562608	0.94156282	-0.086870743	0.64812354	1
Ppp2r2b	1.02158697	0.030812031	0.64816149	1
Mdm4	0.96402423	-0.05285868	0.64816735	1
Abcc9	1.05003437	0.070436546	0.64821334	1
Cd44	0.97870524	-0.031053672	0.64823126	1
Grid2ip	0.93226001	-0.10119572	0.64829873	1
Tmem177	0.93629495	-0.094965019	0.64834653	1
Tubb3	1.02273446	0.032431609	0.64838898	1
Kcnb2	1.03611878	0.051189404	0.64846939	1
Bard1	0.89061059	-0.167133323	0.64857118	1
Glrx2	1.03395401	0.048172018	0.64857328	1
Sec23ip	1.0298054	0.042371734	0.64858451	1
Scml4	0.9649973	-0.051403184	0.64871001	1
Fam229b	1.06714598	0.093757539	0.64885891	1
Tdrp	0.97384196	-0.038240424	0.64895925	1
Ntng1	1.0267973	0.038151409	0.64895936	1
Zmpste24	1.02813376	0.04002797	0.64904462	1
Bcat1	0.97825587	-0.031716229	0.64916206	1
Parp9	1.03480592	0.049360219	0.64940341	1
Dcaf8	1.02421378	0.034516868	0.64945453	1

Oma1	1.07625913	0.106025473	0.64965169	1
Ric3	1.02660675	0.037883648	0.64981454	1
Osbpl5	0.97331157	-0.039026397	0.64997346	1
Srrm1	0.9764585	-0.034369368	0.64997561	1
Hdac2	0.97711469	-0.033400189	0.65002117	1
Grin1	0.97793757	-0.03218573	0.65002223	1
Foxj3	0.9735514	-0.038670938	0.65002745	1
Ing5	1.05683349	0.079748086	0.65011274	1
Fxyd2	1.02785215	0.039632762	0.65013511	1
Hecw1	1.0228779	0.032633945	0.65014822	1
Tob2	0.95682194	-0.063677628	0.65015575	1
Tefm	1.06442255	0.090070975	0.65017878	1
Zbtb5	0.95701266	-0.063390082	0.65023043	1
Ttc12	0.93230172	-0.101131167	0.65030445	1
Hnrnph1	1.02104798	0.030050668	0.65032284	1
Rps8	0.97868721	-0.031080254	0.65036975	1
Phldb1	0.97659547	-0.03416701	0.65037204	1
Keap1	0.97301471	-0.039466479	0.6503848	1
Wnt11	0.90449404	-0.144817099	0.65047678	1
Aldh5a1	1.0323285	0.045902121	0.65047911	1
Vom2r44	1.10082957	0.138591127	0.65049254	1
Fam53a	0.92433539	-0.113511677	0.65068693	1
Nrxn2	0.97796356	-0.032147383	0.65072712	1
Pias1	0.96701272	-0.048393228	0.65100357	1
Ago2	0.9550526	-0.066347901	0.65109397	1
Alg9	1.03435397	0.048729978	0.65112331	1
Thumpd1	0.97275158	-0.039856683	0.65117964	1
Lrrc20	1.04570533	0.064476377	0.65118388	1
Uchl1	1.02239304	0.031949925	0.65120485	1
Gys1	0.97530476	-0.036075001	0.65127591	1
Mier3	1.03588887	0.050869243	0.65143508	1
Lingo4	0.94566801	-0.080594306	0.65148047	1
Vps50	0.97904472	-0.03055334	0.65162787	1
Siae	0.96682309	-0.048676169	0.65168305	1
Reep3	0.97102611	-0.042418005	0.65172006	1
Clybl	1.04239226	0.059898282	0.652108	1
Pecr	1.04513663	0.063691552	0.6521422	1
Cep83os	1.05150263	0.072452463	0.65218456	1
Pcyt1b	0.97316197	-0.039248156	0.65241598	1
Oas1b	1.09425039	0.129942902	0.6524567	1

Spice1	1.06024979	0.084404196	0.65253567	1
Extl3	0.97393301	-0.03810555	0.65257001	1
Rilp	1.09197097	0.126934498	0.6525748	1
Ola1	1.02112518	0.030159739	0.65258587	1
Arv1	1.04531795	0.06394183	0.65259105	1
Ypel4	1.03713609	0.052605214	0.65267506	1
Ankrd49	1.03913475	0.055382752	0.65285469	1
Hspa4	0.97939025	-0.030044261	0.65298857	1
Ncam1	0.97773451	-0.032485322	0.65299864	1
Bap1	0.97894077	-0.030706516	0.65320124	1
Actl6b	0.96022851	-0.058550324	0.65323912	1
Uvssa	0.92526882	-0.112055516	0.6534199	1
Dnajc5	0.97297573	-0.039524283	0.65343132	1
Pyroxd2	0.95146648	-0.071775265	0.65353906	1
Zfp217	1.06762243	0.094401519	0.65361126	1
Acss2	1.02240867	0.031971972	0.65376738	1
Slc7a6	1.03002574	0.042680389	0.65380295	1
Il17rd	1.08452544	0.117063893	0.65381288	1
Il10rb	1.04047107	0.057236852	0.65389343	1
Tmem206	0.95572332	-0.065335068	0.65390753	1
Patl1	0.96816662	-0.046672734	0.65407541	1
Tsfm	1.04065624	0.057493585	0.65411573	1
Maff	0.93855842	-0.091481541	0.65412999	1
Sh2b1	0.96956847	-0.044585318	0.65413962	1
Clmn	0.95993993	-0.058983971	0.65420862	1
Dapk2	0.93395288	-0.098578324	0.65421625	1
Jmjd7	0.90801479	-0.139212303	0.654345	1
Eml4	0.97536235	-0.035989818	0.65446245	1
Cbln2	0.97656421	-0.034213186	0.65446736	1
RGD1310110	0.97515239	-0.036300407	0.65464843	1
Mlh1	0.95634441	-0.064397819	0.65465684	1
Smim7	1.02560447	0.036474449	0.65475646	1
Trnt1	0.97168801	-0.041434923	0.65506395	1
Alg8	0.94970263	-0.074452247	0.65506727	1
Nfatc2	1.08133303	0.112810909	0.65522312	1
Aplnr	1.07175753	0.099978558	0.65530139	1
Polr2j	1.03571426	0.050626042	0.65534445	1
Slc29a3	1.05478784	0.076952848	0.6554038	1
RGD1308106	0.94605316	-0.080006848	0.65551921	1
Mid1ip1	0.97950622	-0.029873437	0.65552086	1

Capn6	0.94141921	-0.087090801	0.65579764	1
Isy1	0.95724969	-0.063032814	0.65589431	1
Enpp2	1.03285015	0.04663096	0.65591577	1
Slx1b	1.0371438	0.052615943	0.65610186	1
Musk	1.10948703	0.149892807	0.65610562	1
Dock11	0.97970842	-0.029575661	0.65622704	1
Vwc2l	1.04514498	0.063703079	0.65624122	1
Thap6	1.06768989	0.094492684	0.6562675	1
Fxyd3	1.08813174	0.121853228	0.65644296	1
Hdhd3	0.93712987	-0.093679104	0.65655623	1
Cyp3a9	0.92814532	-0.107577393	0.65661985	1
Mrps9	0.96965786	-0.044452306	0.65675473	1
Fxyd6	1.02090946	0.029854921	0.65687594	1
Ctsd	1.02134259	0.030466867	0.65689035	1
Rpl36a	0.96462591	-0.051958536	0.65689865	1
Prrg4	0.94813667	-0.076833064	0.656921	1
Tgfb2	0.9620982	-0.055743942	0.65696616	1
Clip4	0.96911574	-0.045259127	0.65715432	1
Prdm11	1.09486364	0.130751206	0.65733458	1
Pddc1	0.96292822	-0.054499831	0.65735604	1
Ggact	1.04468507	0.063068098	0.65748361	1
RGD1565002	1.02739578	0.03899205	0.65775747	1
Gfpt2	1.03301599	0.046862581	0.65786195	1
Epn3	0.97703618	-0.033516115	0.65787322	1
Fam227a	1.0711915	0.099216423	0.65789988	1
Ywhab	0.97902305	-0.030585273	0.65801293	1
Pkig	1.03151781	0.04476873	0.65806599	1
Fam91a1	0.97891385	-0.03074619	0.65808238	1
Ssc5d	1.03792746	0.053705617	0.65812546	1
Brd2	0.97912103	-0.030440885	0.65817259	1
Lmbr1	0.9653998	-0.050801563	0.65818328	1
Ubn2	1.03812689	0.053982801	0.65819298	1
Fam96b	1.03253518	0.046190937	0.65820146	1
Tmco4	0.94221196	-0.085876456	0.65832067	1
Exoc6	0.97219286	-0.040685557	0.65841647	1
Gatc	0.96903146	-0.045384593	0.65843547	1
Gpr150	1.1112221	0.152147197	0.65849128	1
Acap1	0.936127	-0.095223831	0.6587299	1
Nfs1	1.02537631	0.036153477	0.65873613	1
Paip1	0.97671254	-0.033994072	0.65879835	1

B4galnt3	0.90729909	-0.140349881	0.65882894	1
Cdh20	1.07043932	0.098203013	0.65897039	1
Kidins220	0.97959472	-0.029743104	0.6590471	1
Pcdhb20	1.07265362	0.101184281	0.65907999	1
Dcp2	1.04998111	0.07036337	0.65937148	1
Tgfb3	0.97083081	-0.042708196	0.6594194	1
Snrpf	1.05158594	0.072566762	0.65963609	1
Cdc34	0.97237293	-0.040418359	0.65976128	1
Eef1akmt1	0.96038467	-0.058315725	0.65981971	1
Aco2	1.02081056	0.02971516	0.65981972	1
Smurf1	0.97444603	-0.037345806	0.65996338	1
Tubb2a	1.02129927	0.030405682	0.66002456	1
Taf1d	1.05535561	0.077729214	0.6601221	1
Rpl7	0.97998467	-0.029168909	0.66017809	1
Manbal	1.02774118	0.039476988	0.66024391	1
Emd	0.96090188	-0.057538971	0.66034371	1
Arih2	0.97464812	-0.037046646	0.66045504	1
Col13a1	0.93076273	-0.103514646	0.66052507	1
Luzp1	0.97976111	-0.029498072	0.66067217	1
U2af1l4	1.03626794	0.051397074	0.66067635	1
Armt1	1.0317583	0.045105046	0.6607212	1
Sipa1	0.96638707	-0.049326939	0.66075376	1
Ephb3	0.93761092	-0.092938728	0.66078261	1
Ppcdc	1.04526463	0.063868232	0.66084172	1
Mrps12	1.03857995	0.05461228	0.66089373	1
Lrp1b	1.04192718	0.059254446	0.66091808	1
Faap20	1.0553409	0.077709095	0.66099824	1
Slc25a34	1.11228	0.153520007	0.66109278	1
Exoc3	0.97730667	-0.033116758	0.66112675	1
Zfand2a	0.97284524	-0.039717777	0.66115909	1
Mgme1	1.07999827	0.111029	0.66123391	1
Ftx	0.93327837	-0.099620637	0.66123901	1
Mea1	1.02653707	0.037785724	0.66128376	1
Zfp638	0.97928415	-0.030200558	0.66130496	1
Sulf2	1.02263809	0.03229567	0.66136807	1
Pcca	0.96490804	-0.051536645	0.66141527	1
Txn2	0.97416532	-0.037761471	0.66147393	1
Zhx1	1.02123768	0.030318674	0.66149205	1
Wdr35	0.9689662	-0.045481752	0.66155713	1
Cntrob	1.07490198	0.104205112	0.66161244	1

Trim24	1.0428501	0.060531801	0.66161616	1
Dnttip1	1.03933558	0.055661541	0.66164272	1
Acot8	1.05009769	0.070523548	0.6616468	1
Atp5f1	1.0204227	0.0291669	0.66176002	1
Pde3b	1.03702074	0.052444748	0.6618283	1
Cd200	0.97951033	-0.029867384	0.6619444	1
Gpr63	0.91821912	-0.123089615	0.66197415	1
Zranb2	1.02074224	0.029618604	0.6620049	1
Zbtb8a	1.07337259	0.102150948	0.66218236	1
Acaca	0.97913331	-0.030422799	0.66218459	1
Ptpn21	1.0418947	0.059209474	0.66219853	1
Lrrc73	0.94464781	-0.082151533	0.66241959	1
Amdhd2	1.05012886	0.070566367	0.66246011	1
Gcsh	1.02813559	0.040030536	0.66263082	1
Mapk1ip1	0.96247309	-0.055181888	0.66265531	1
Rab9a	0.96493352	-0.051498542	0.66277412	1
Rps6kb1	0.97360525	-0.03859115	0.66278649	1
Impad1	0.97959194	-0.029747184	0.66278922	1
Exoc1	1.02431864	0.034664574	0.66279435	1
Nop56	1.02355568	0.03358959	0.66304363	1
Cep76	1.04845374	0.068263214	0.66306121	1
Fam193b	1.030382	0.043179291	0.66310496	1
Churc1	1.03712013	0.052583015	0.6631084	1
Rrp8	0.95055803	-0.073153396	0.66311053	1
Mettl14	1.02926559	0.041615299	0.66334615	1
Dkc1	0.96724773	-0.048042663	0.66335508	1
Ube3b	1.02052689	0.029314195	0.66341858	1
Otub2	0.94471414	-0.08205025	0.66355526	1
Ttc8	0.94436655	-0.082581148	0.66369394	1
Zfp513	1.03869453	0.054771431	0.66372856	1
Ifi35	1.0560981	0.078743849	0.66376262	1
Daam1	0.97588838	-0.03521195	0.66382916	1
Fmod	1.03348611	0.047519001	0.6638634	1
Rabgef1	1.02996343	0.042593112	0.66389257	1
Rpusd1	1.04448155	0.062787004	0.664013	1
Spns2	1.03625727	0.051382217	0.66441822	1
Fnbp1l	1.05772	0.080957765	0.6645605	1
Zfp148	0.9727658	-0.039835591	0.66499954	1
Ccdc51	0.95416498	-0.06768935	0.6650431	1
Alas2	0.97168899	-0.041433478	0.66505741	1

Fam216a	1.02932223	0.041694687	0.66506108	1
RGD1307155	0.92182452	-0.117435953	0.66513993	1
Ctdp1	0.96466641	-0.051897967	0.66515533	1
Fam134c	0.97065278	-0.042972787	0.66521165	1
Mapre3	0.97967184	-0.029629524	0.66527359	1
Erh	1.02660618	0.037882857	0.66572286	1
Phyhd1	0.93071796	-0.103584053	0.66572468	1
St3gal3	1.06028392	0.084450636	0.66601767	1
Catsperg1	1.06055658	0.084821588	0.66616928	1
Uqcrq	1.02404912	0.034284918	0.66626467	1
Atp6v1a	1.02038513	0.029113774	0.66628699	1
Golga3	0.97671598	-0.033989001	0.66635925	1
Ttc7a	1.04085423	0.057768038	0.66636511	1
Osgep	1.05661186	0.07944551	0.66648937	1
Nhlrc1	1.0747058	0.103941772	0.66653747	1
MGC105649	1.09769559	0.13447802	0.66656994	1
Calb1	1.0556592	0.078144164	0.66659558	1
Tdp1	0.94412113	-0.082956127	0.66668645	1
Nek1	0.9763908	-0.034469392	0.66669657	1
Zfp131	1.02949462	0.041936294	0.66672003	1
Ube2i	0.97802156	-0.032061824	0.66682425	1
Slc25a2	0.92147721	-0.117979616	0.66685853	1
Pard3b	0.93911828	-0.090621225	0.66700307	1
Gli2	0.93820107	-0.092030942	0.66701943	1
Srgap1	0.96574471	-0.050286225	0.66713911	1
Med31	0.95980056	-0.059193444	0.66714044	1
Kcnmb4	1.0613142	0.085851823	0.66725508	1
Pogk	0.97684445	-0.033799246	0.66728865	1
Slc6a12	0.90772115	-0.139678924	0.66735103	1
Scn5a	0.93965824	-0.089791962	0.66751849	1
Tbc1d5	1.02950522	0.041951146	0.66757645	1
Enho	1.03760478	0.053257037	0.66770748	1
Blk	0.87490973	-0.192793914	0.66773174	1
Camsap1	1.02232055	0.031847627	0.66775656	1
Zdhhc20	1.03281948	0.046588116	0.66788201	1
Flad1	1.02869512	0.040815469	0.66788957	1
Bex4	0.95459124	-0.067044999	0.66790089	1
Dis3	1.03744557	0.053035646	0.66801545	1
Phb	1.02177703	0.031080416	0.66804211	1
Ermn	1.08738255	0.120859582	0.66813103	1

S100a6	1.01997125	0.028528488	0.66816839	1
Dnajc8	1.02165643	0.030910118	0.66828117	1
Abcg3l2	0.92008954	-0.120153822	0.6682997	1
Slc25a40	1.04305392	0.060813745	0.66833729	1
Zfat	0.9614547	-0.056709203	0.66837864	1
Gabrg1	1.02430249	0.034641818	0.66842835	1
Rpl13	1.0197946	0.0282786	0.66846466	1
Fhit	1.05911408	0.082857999	0.66858797	1
Dpy19l3	1.0351877	0.049892384	0.66864367	1
Ier3	1.04417974	0.062370078	0.66867811	1
Dlc1	0.9737169	-0.038425718	0.66877592	1
Smim20	1.03342143	0.047428708	0.66906919	1
Cntnap5b	0.91620793	-0.126253038	0.669092	1
Gdap2	1.0277531	0.039493719	0.66909897	1
Lrrc28	0.97246207	-0.040286118	0.6693117	1
Adgrl2	1.02649031	0.037720005	0.66931888	1
Slc38a6	1.08623632	0.11933801	0.66949483	1
Fau	1.02024114	0.028910185	0.66952288	1
Zfp26	0.9482061	-0.076727421	0.66963272	1
Pank4	1.03083916	0.043819251	0.66968429	1
Trib1	1.05389361	0.075729235	0.66969032	1
RGD1564804	1.05094905	0.071692723	0.66973023	1
Pfkfb3	1.03065194	0.043557204	0.66975912	1
Tincr	0.95500456	-0.066420479	0.66989182	1
Fam185a	1.04176479	0.059029585	0.66990385	1
Fn1	1.01993218	0.02847322	0.67002349	1
Casp8ap2	1.03171685	0.04504709	0.67004633	1
Dld	1.01978267	0.028261727	0.6700524	1
Tmem119	0.92860349	-0.106865392	0.67024754	1
Brcc3	1.03483271	0.049397569	0.67033748	1
Hic2	0.9123598	-0.132325214	0.67039746	1
Egr2	0.97047917	-0.043230849	0.6703978	1
Pi4k2b	1.04527224	0.063878736	0.67042696	1
Aifm2	0.96134094	-0.056879928	0.67042797	1
LOC498368	1.05465024	0.076764627	0.67044115	1
Cobl	0.97542387	-0.035898824	0.67053827	1
Osbpl6	0.97364923	-0.038525981	0.67064665	1
Shkbp1	1.05280293	0.074235408	0.67114711	1
Ctdnep1	1.0292725	0.041624985	0.67117159	1
Ttc33	1.03074707	0.043690354	0.67133333	1

Rbm41	1.06465508	0.090386117	0.67134784	1
Gng11	1.03516603	0.049862179	0.67141061	1
Bambi	1.06709028	0.093682236	0.67141809	1
Kcna5	0.94881215	-0.075805606	0.67143157	1
Mrc2	1.03204766	0.045509598	0.67144325	1
Tmem173	0.92738043	-0.108766806	0.67165956	1
Lrfn2	0.93599301	-0.095430345	0.67170298	1
Ptrf	0.97994818	-0.029222639	0.67176409	1
Rab1b	0.98038276	-0.028582977	0.6718548	1
Epm2aip1	1.01973201	0.028190049	0.6719596	1
Fut4	0.94134007	-0.08721209	0.6720738	1
Ist1	1.02116426	0.030214957	0.67234154	1
Rpl18a	1.01962952	0.028045053	0.67238109	1
Clec4d	0.84505523	-0.242882465	0.6726699	1
Rnpep	1.03520073	0.049910542	0.67270898	1
Peg3	0.98022177	-0.028819905	0.67275124	1
Prkd2	1.06111356	0.085579066	0.6727638	1
Mapk7	1.03969592	0.056161645	0.67280962	1
Samd5	0.91677952	-0.125353274	0.67281174	1
Rims3	0.96551121	-0.050635092	0.67281935	1
Stk39	1.01955264	0.027936259	0.67282302	1
Orc6	0.9377659	-0.092700278	0.67305604	1
Adck4	1.0377197	0.053416805	0.6731155	1
Yaf2	0.97833521	-0.03159923	0.67315538	1
Ppcs	0.96609154	-0.049768205	0.67319971	1
Igsf21	1.06076197	0.085100962	0.67322687	1
Slc25a32	0.96632354	-0.049421795	0.67330214	1
Gon4l	0.97174483	-0.041350571	0.67330895	1
Nudt16	0.96570463	-0.050346102	0.67341722	1
Psme3	0.97649125	-0.03432098	0.67343218	1
Abhd14b	0.9667511	-0.048783597	0.67345686	1
Rdh13	0.95770823	-0.062341891	0.6735333	1
Vimp	0.9747581	-0.036883863	0.67356948	1
Hsf1	0.97069582	-0.042908814	0.67374965	1
Map1lc3a	0.9805803	-0.028292312	0.67376775	1
Chuk	0.97021215	-0.04362785	0.6738281	1
Fam43a	1.04488337	0.06334192	0.67386426	1
Trappc8	1.02074052	0.029616162	0.67392399	1
Taok1	0.97575503	-0.035409095	0.67397956	1
Gtpbp8	0.94308786	-0.084535917	0.67429206	1

Adamts4	0.92131159	-0.118238927	0.67435252	1
Opa3	0.96677329	-0.048750473	0.67437432	1
Hnrnpa3	1.01950856	0.027873884	0.67438079	1
Dync1i2	0.98040015	-0.028557386	0.674463	1
Ost4	1.02338382	0.033347335	0.67454891	1
Mab21l2	0.97228233	-0.040552793	0.67464521	1
Nudt1	1.0800303	0.111071789	0.67471167	1
Pcdh18	1.03436261	0.048742031	0.67471986	1
Clint1	1.02940132	0.041805533	0.67492464	1
Ddx46	0.97862192	-0.031176496	0.67511062	1
C1qtnf7	0.93919925	-0.090496839	0.67540646	1
Map2k4	1.02237609	0.031925997	0.67541357	1
Tax1bp1	1.01965798	0.028085322	0.67541799	1
Rhobtb3	1.02704181	0.038494909	0.67552904	1
Snrpb	1.02918425	0.041501279	0.67553817	1
Cers4	1.02447215	0.034880766	0.67559054	1
Flnb	1.02023729	0.028904738	0.6756222	1
Tmem242	1.03609074	0.051150364	0.67569079	1
Mrps15	1.02493459	0.035531838	0.67576473	1
Klhl15	1.07872296	0.109324396	0.67576688	1
Amigo1	0.97256668	-0.040130935	0.67576803	1
Arhgap24	1.03672637	0.052035171	0.67581878	1
Slc25a30	1.04028456	0.056978216	0.67588904	1
Chchd5	1.06277305	0.087833553	0.67595858	1
Slc9a7	1.0433379	0.061206474	0.67611583	1
Kif1c	0.97595521	-0.035113158	0.67619705	1
Trim59	1.05003866	0.07044245	0.67621322	1
Cds2	1.02133388	0.030454566	0.67622789	1
Pax5	0.7954435	-0.33016863	0.67623224	1
Ppm1l	0.97989927	-0.029294649	0.67627071	1
Pkdcc	1.04042538	0.057173497	0.67627403	1
Ak1	1.02185605	0.031191972	0.67628607	1
Arntl	1.05055114	0.071146394	0.67636212	1
Ddx20	1.03641944	0.05160798	0.67642796	1
Pigs	0.97570672	-0.035480523	0.67652735	1
Bles03	1.02747568	0.039104251	0.6765869	1
Zfp362	0.95051679	-0.073215976	0.67666238	1
Lgals8	1.01930222	0.027581869	0.67671014	1
L2hgdh	0.92749356	-0.10859083	0.67683213	1
Rbm18	1.02245803	0.032041627	0.67691235	1

Clcn7	0.97049919	-0.043201092	0.67691535	1
Calr3	0.92861803	-0.106842805	0.67709535	1
Hivep3	0.96321978	-0.054063084	0.67722859	1
Fktn	0.97455983	-0.037177333	0.67723705	1
Wdr53	1.05059395	0.071205187	0.6773186	1
Wnt4	1.10121851	0.139100767	0.67753595	1
C1qtnf5	1.03970752	0.056177735	0.67764978	1
Rnf19a	0.9810699	-0.027572161	0.67770181	1
Brd7	0.97261419	-0.040060456	0.67770783	1
Rdh10	0.94685121	-0.078790364	0.67781638	1
Bmpr1a	1.02413026	0.034399221	0.6779334	1
Nop14	1.02966804	0.042179296	0.67821237	1
Ccdc85a	0.96605851	-0.049817524	0.67852248	1
Zmiz1	0.97865745	-0.031124125	0.67853034	1
Gemin2	1.04859724	0.068460649	0.67855171	1
Rplp1	1.02134341	0.030468024	0.6785691	1
Fis1	1.02112245	0.030155887	0.6785951	1
Spata13	0.96825593	-0.046539659	0.67879987	1
Capn7	0.97601469	-0.035025234	0.67882624	1
Tmed3	0.97315326	-0.039261057	0.67902682	1
Pkhd1l1	1.04499932	0.063502009	0.67907721	1
Zfp637	0.9667709	-0.048754043	0.67914438	1
Dnajc15	1.0338968	0.048092194	0.67917133	1
Npr2	1.02022133	0.028882169	0.67929262	1
Rffl	0.95424818	-0.06756356	0.67956201	1
Nsf	1.01957037	0.027961355	0.6795683	1
Tcf20	0.97541269	-0.035915356	0.67970556	1
Hibch	1.03365828	0.047759313	0.67973436	1
Lin7b	0.95391142	-0.068072785	0.67987985	1
Scap	1.02276059	0.032468479	0.6799121	1
Edem3	1.02698242	0.03841149	0.68005463	1
Cd82	0.97993404	-0.029243453	0.68009152	1
Maged1	1.01917371	0.027399965	0.68050171	1
Vps29	0.97992514	-0.029256555	0.68050907	1
Gars	1.01929796	0.027575837	0.68056498	1
Nxpe1	1.07428234	0.103373212	0.68057616	1
Fkbp2	1.02720594	0.038725446	0.68058542	1
Dync1li2	0.98103619	-0.027621744	0.68071875	1
Sf3b5	1.02564136	0.036526351	0.68077358	1
Chp1	0.98120396	-0.027375037	0.68083996	1

Rnf128	0.94025613	-0.088874283	0.68084575	1
Ccz1b	1.02650174	0.037736071	0.68092253	1
Ptp4a3	0.96436779	-0.052344626	0.68093357	1
Tcf7l2	1.05033905	0.070855109	0.68107388	1
Zfp772	0.96198661	-0.055911278	0.68110027	1
Spata2L	0.93118916	-0.102853835	0.68118206	1
Mboat1	0.96263749	-0.05493549	0.68123109	1
Fam83f	1.04483725	0.063278234	0.68124176	1
Slc30a9	0.98084893	-0.027897149	0.68126027	1
Enthd2	0.96672049	-0.048829281	0.6814355	1
RGD1565222	1.05985099	0.083861441	0.68144008	1
Kctd11	0.95509438	-0.066284795	0.68144938	1
Rpl22l1	1.02544254	0.036246659	0.6814865	1
Eif2b5	1.02256961	0.032199054	0.68149383	1
Impa2	0.95880551	-0.060689888	0.68150604	1
Ccdc146	1.08116044	0.112580634	0.68153896	1
Riok1	1.03161079	0.044898773	0.68163013	1
Fam214a	0.97151936	-0.041685355	0.68164247	1
Sgcz	0.92042209	-0.119632487	0.68169942	1
Psat1	0.96722107	-0.048082424	0.68193054	1
Itga11	1.04017217	0.056822344	0.68194887	1
RGD1562079	0.97021232	-0.04362759	0.68200785	1
Gpcpd1	1.02045699	0.02921537	0.68204177	1
Nf1	0.98035683	-0.028621138	0.68212737	1
RT1-N1	1.1073565	0.147119759	0.68216123	1
Arf2	1.03071692	0.043648156	0.68226461	1
Asb2	0.91751778	-0.124191973	0.68228858	1
Cdca2	1.10181101	0.139876783	0.68242433	1
Fgf9	1.03348348	0.047515326	0.68246707	1
Arf4	1.01935263	0.027653218	0.68252486	1
Hdac10	1.04228239	0.05974621	0.68266742	1
Acvr1	0.95964518	-0.059427014	0.68273963	1
Tdrd7	1.0212202	0.030293978	0.68278676	1
Vars2	0.95915771	-0.060160042	0.68284803	1
Satb1	0.97102807	-0.042415092	0.68286045	1
Tfb2m	1.0266769	0.037982229	0.6829625	1
Ctnnbl1	1.02810803	0.03999187	0.68297661	1
Vprbp	0.9737045	-0.038444079	0.68300645	1
Qrich1	1.0243143	0.034658456	0.6833391	1
Purg	1.05666011	0.079511393	0.68337606	1

Ccdc181	1.03069525	0.043617824	0.68340764	1
Gemin8	1.04198626	0.059336253	0.68362245	1
Cpn1	0.91556413	-0.127267151	0.68366075	1
B9d1	0.94853958	-0.076220117	0.68378413	1
Timm8b	1.02214927	0.031605902	0.68383539	1
Tle2	0.94167958	-0.086691849	0.68388007	1
Phf7	0.95273999	-0.069845542	0.68393246	1
LOC681325	0.84579148	-0.241626065	0.68395696	1
Llgl1	0.97239186	-0.040390277	0.68400769	1
Eya4	0.90012873	-0.151796757	0.68400936	1
Etnk2	1.09684788	0.133363456	0.68401968	1
Slf2	0.97392951	-0.038110738	0.6840234	1
Gne	1.03336843	0.047354708	0.68417346	1
Abcc2	1.09149347	0.126303504	0.68421659	1
Ptpmt1	0.96877943	-0.045759859	0.6842752	1
Tub	0.91700942	-0.124991544	0.68429154	1
Ttc14	1.0295124	0.041961205	0.68445865	1
Sec14l2	0.97580952	-0.035328539	0.68454789	1
Jam3	1.02382726	0.033972325	0.6845823	1
Osbpl9	1.01885442	0.02694792	0.68460908	1
Nup58	1.02947514	0.041908992	0.68466933	1
Eef1e1	0.97086638	-0.042655339	0.68479798	1
Crispld2	0.9797294	-0.02954476	0.68482134	1
Zfand6	1.02232467	0.031853447	0.68483892	1
Get4	1.02600518	0.03703801	0.68506934	1
Abtb1	1.02950171	0.041946231	0.68507145	1
Papss1	0.98093094	-0.027776519	0.6851068	1
Cfl2	0.98162743	-0.026752525	0.68522669	1
Tpcn1	1.02017284	0.028813592	0.68525489	1
Rhno1	1.05125748	0.072116069	0.68529557	1
Vwa1	1.0194405	0.027777578	0.68544495	1
Ilf2	0.9727446	-0.039867031	0.68545729	1
Sod3	1.02048243	0.029251343	0.68551747	1
Kmt5c	0.9650202	-0.051368957	0.68561124	1
Tkfc	0.94654888	-0.079251089	0.68563208	1
Sytl3	0.96567202	-0.050394815	0.6857304	1
Rpl30	0.97944176	-0.02996839	0.68591605	1
Usp6nl	0.97714055	-0.033362003	0.68599102	1
Lrrc1	0.94960043	-0.0746075	0.68627251	1
Pink1	1.02315846	0.033029592	0.68637787	1

Gtf2h4	1.04553473	0.064240982	0.68639365	1
Hmbs	1.03108132	0.044158127	0.68641246	1
Zdhhc6	1.0283544	0.04033755	0.68646174	1
Pdlim4	0.96928855	-0.045001887	0.68648885	1
Gtdc1	0.96731253	-0.047946013	0.68663703	1
Ythdc1	1.0237077	0.033803834	0.68664621	1
Efcc1	1.08217052	0.113927851	0.68683353	1
Rpp30	0.9710595	-0.042368391	0.68692904	1
Ntn1	1.03672311	0.05203063	0.6869404	1
Jun	0.98088724	-0.027840803	0.68712892	1
Mettl25	0.9479771	-0.077075881	0.6871953	1
Ngrn	1.01910196	0.027298403	0.68725758	1
Parp16	1.06286082	0.087952684	0.68735471	1
Pak1ip1	1.05315092	0.074712199	0.68738969	1
Adamts15	0.95951278	-0.059626072	0.68744305	1
Tbc1d17	0.97199588	-0.04097789	0.68763224	1
Dusp16	0.9413808	-0.08714966	0.68764409	1
Rtn3	1.01998298	0.028545079	0.68770611	1
Filip1	0.97237042	-0.040422081	0.68771544	1
R3hdm2	0.97869259	-0.031072313	0.68778417	1
Usp13	0.97840129	-0.031501793	0.68778966	1
Pcgf1	0.96329713	-0.053947223	0.6878096	1
Stat5a	1.03802714	0.053844158	0.68783312	1
Recql4	0.91757397	-0.124103627	0.68785442	1
Gstcd	0.93372532	-0.098929887	0.68797984	1
Rai2	1.05675052	0.079634816	0.68808174	1
Sarnp	1.02642126	0.037622952	0.68809961	1
Trappc2b	0.97345811	-0.038809197	0.68813293	1
Lamc3	0.94961588	-0.074584036	0.68822025	1
Myoz1	1.13121202	0.177869351	0.6882913	1
Gtf2f2	1.04527433	0.063881619	0.68830818	1
Golga7b	0.96485451	-0.051616685	0.68835484	1
Mafk	1.03416114	0.048460997	0.68835886	1
Pdpk1	0.97547735	-0.035819718	0.68842842	1
Klhl9	0.98178404	-0.026522385	0.68851983	1
Ccnl2	0.97174241	-0.04135416	0.68861995	1
Robo2	0.9817909	-0.026512301	0.68868083	1
Tcn2	1.02337361	0.03333293	0.68869545	1
Epb42	0.91422296	-0.129382051	0.68872971	1
Ccdc59	1.04207742	0.059462463	0.68873202	1

15-Sep	1.01851693	0.026469963	0.68877107	1
Magi3	1.01933635	0.027630179	0.68880692	1
Scfd1	0.97731323	-0.033107077	0.68883624	1
Oas1k	1.20944238	0.274342034	0.68897799	1
Mthfd2	1.03819578	0.05407853	0.68915288	1
RGD621098	1.03266819	0.046376773	0.68926101	1
Fam207a	1.03212098	0.04561209	0.68957798	1
Bcas2	1.02883068	0.04100557	0.68959387	1
Rhbdl1	1.06267573	0.087701437	0.68961058	1
Pmm1	1.01977745	0.028254346	0.68961373	1
Smarcc1	1.02442327	0.034811924	0.68969337	1
Rasgrp4	1.10905313	0.149328481	0.68975541	1
Myg1	0.96892203	-0.045547524	0.6898394	1
Flvcr2	1.04969486	0.069970003	0.68995074	1
Tmem38b	0.95317744	-0.069183289	0.6900423	1
Nr4a2	1.05138557	0.072291846	0.69009777	1
Dpy30	1.02765403	0.039354651	0.69014122	1
Gna11	1.04626148	0.065243458	0.69015609	1
Cnot6l	1.04170809	0.058951063	0.69037305	1
Xrcc1	0.96959122	-0.044551466	0.69040857	1
Sertad3	1.06490245	0.090721281	0.69055307	1
Thy1	1.01945144	0.027793059	0.69055578	1
Hyal1	0.9455797	-0.080729027	0.6905692	1
Sephs1	1.02756557	0.039230455	0.69058873	1
Dhps	1.02770839	0.039430957	0.69059542	1
Gapt	1.20800515	0.272626611	0.69073472	1
Pex11a	0.9565526	-0.06408379	0.6907372	1
Rpp40	0.9654444	-0.050734919	0.69080901	1
Atp1b1	1.01949675	0.027857183	0.69085027	1
Psmd2	1.01852063	0.026475206	0.69092172	1
Mynn	0.96292244	-0.054508491	0.69094131	1
Slc39a6	0.97808544	-0.031967591	0.69098968	1
Arhgap18	1.03739075	0.052959415	0.69109978	1
Hddc3	1.04255352	0.060121452	0.69123674	1
Ift80	1.02696242	0.038383385	0.69124537	1
Pdcd2l	0.94913451	-0.075315537	0.69132061	1
Taf8	1.03506847	0.049726209	0.69168263	1
Aaas	0.96223803	-0.05553428	0.69171978	1
Rpl10	1.0184327	0.026350651	0.69175462	1
Bbs10	0.96251213	-0.055123377	0.69193773	1

Cuedc1	0.96374864	-0.053271173	0.69202079	1
Bex2	1.01929569	0.027572627	0.69209085	1
Znrf2	1.0244071	0.034789164	0.69213281	1
Diaph1	1.0290173	0.04126724	0.69223111	1
Haus3	0.96484158	-0.051636014	0.69228156	1
Rbbp4	1.02494499	0.035546481	0.69233808	1
Sgcb	1.01922367	0.027470689	0.69244478	1
Rapsn	0.93348129	-0.099306991	0.69252897	1
Ppt2	0.96506026	-0.051309066	0.69256805	1
Ripk3	0.92908785	-0.106113071	0.69271756	1
Tgfbrap1	0.97031335	-0.043477369	0.69282494	1
Ncbp2	0.97894915	-0.030694166	0.6928808	1
Atp5g1	1.02246306	0.032048726	0.69290673	1
Deaf1	0.97234869	-0.040454324	0.69290803	1
Cd177	0.91630345	-0.126102641	0.69294597	1
Mplkip	1.04105681	0.058048798	0.69313837	1
Bcl2l11	0.94511471	-0.081438659	0.69314639	1
Zfp865	0.94374153	-0.0835363	0.69324272	1
Nphp3	0.9458448	-0.080324616	0.69325983	1
Abcc3	1.05040254	0.070942313	0.69335991	1
Rps3a	0.98207944	-0.026088364	0.69351042	1
Ctnnd1	0.98140109	-0.027085218	0.69394587	1
Schip1	0.97947157	-0.029924473	0.69400365	1
Med4	1.0342882	0.048638237	0.69420795	1
Akap9	0.98166874	-0.026691815	0.69425717	1
Kat5	1.02431859	0.034664504	0.69441775	1
Rps6ka3	1.0200326	0.028615262	0.69449857	1
Rpgrip1l	1.0295353	0.041993298	0.69449878	1
Vps35	0.98199457	-0.026213049	0.69467239	1
Eif5a	1.01816363	0.025969444	0.69473534	1
Sh2d4a	1.06624196	0.092534856	0.69488261	1
Fgf13	1.01851742	0.026470649	0.69493782	1
Wrb	1.01975758	0.02822623	0.69494565	1
Depdc5	1.01984322	0.028347387	0.69496144	1
Ttc9b	0.96599249	-0.04991612	0.69498955	1
Rimbp2	0.97050532	-0.043191972	0.69510588	1
Pih1d2	1.07372167	0.102620064	0.6951305	1
Arpc4	1.01843363	0.026351962	0.69516322	1
Rps21	0.94332634	-0.084171151	0.69527513	1
Ppwd1	1.03919005	0.055459517	0.69542441	1

Rab20	0.93761815	-0.092927595	0.69547305	1
Nrbf2	1.02914663	0.041448543	0.69576921	1
Zfp511	1.04661876	0.065736023	0.69580688	1
Gtf2h2	1.04233387	0.059817462	0.69590297	1
Creb1	0.949546	-0.074690201	0.69591865	1
Smpd1	0.98152297	-0.026906069	0.69594538	1
Mxi1	0.97991338	-0.029273868	0.69601922	1
Adrb2	0.92765572	-0.108338612	0.69613109	1
Frmpd4	1.02311798	0.032972517	0.6962533	1
Adcy8	0.9618889	-0.056057833	0.69633073	1
Rgl1	0.97965244	-0.029658096	0.69635075	1
Pla2g6	1.02345778	0.033451589	0.69637092	1
Tgfa	1.03251441	0.046161917	0.69658993	1
Lcmt2	1.0537454	0.075526336	0.69673782	1
Pnkp	1.04366016	0.061652014	0.69676722	1
Usp40	1.0283001	0.040261369	0.69680971	1
Phf11	1.08024356	0.111356625	0.69684311	1
Zufsp	0.96539754	-0.050804947	0.69687595	1
Btf3	1.01874191	0.026788599	0.69690883	1
Cdk10	1.02596137	0.036976408	0.6969176	1
Mrps23	1.02551817	0.03635305	0.69697223	1
Mtfp1	0.9261545	-0.110675212	0.69699687	1
Irs2	0.97858253	-0.031234566	0.69701283	1
Vwa5b2	0.97720858	-0.033261564	0.6970598	1
Alkbh1	1.04099512	0.057963311	0.69712858	1
Maml3	0.93034921	-0.104155756	0.69722554	1
Rad51	0.94065597	-0.088260921	0.69722765	1
Dpp7	1.0229587	0.032747895	0.69726033	1
Rchy1	1.02667675	0.037982024	0.69727504	1
Nup85	1.03510895	0.049782631	0.6972842	1
Nhp2	1.03263156	0.046325598	0.69731968	1
Rnf20	1.02010176	0.028713069	0.69733294	1
Gemin4	1.03309991	0.046979784	0.69735129	1
Snx16	1.02329304	0.033219342	0.69751432	1
Snrnp70	1.02027932	0.028964176	0.69753507	1
Rtkn	0.9815839	-0.026816515	0.69768166	1
RGD1308706	1.0312501	0.044394259	0.69771213	1
Tab2	0.97736061	-0.033037126	0.69789153	1
Ptdss1	0.9805843	-0.028286436	0.69803662	1
Prpf3	1.02992064	0.042533169	0.69813349	1

Kdelr2	1.02255156	0.032173588	0.69848558	1
Acads	1.0267603	0.038099418	0.69878842	1
Atrip	0.96039691	-0.058297326	0.69885005	1
Phkb	0.97251677	-0.040204973	0.69899923	1
Zfand5	0.98247931	-0.025501073	0.69922177	1
Fgr	0.9370022	-0.09387566	0.69930946	1
Uba1	0.98211122	-0.026041688	0.69936791	1
Sdc4	1.02555222	0.03640095	0.69946175	1
Nfic	0.97743121	-0.032932922	0.69952618	1
Ten1	0.9512207	-0.072147991	0.69972375	1
Arl2bp	1.01902468	0.027188987	0.69979527	1
Gria4	1.03195795	0.045384187	0.69995281	1
Mark1	1.02262523	0.03227752	0.70002299	1
Helq	0.96303354	-0.05434205	0.70002802	1
Pi4kb	0.97891537	-0.030743949	0.7000693	1
Vegfb	1.03857578	0.054606485	0.70016792	1
Col16a1	1.02073853	0.029613362	0.70020777	1
Ispd	0.96966905	-0.044435658	0.70035999	1
Atp6v0c	0.98222199	-0.025878967	0.70047066	1
Ssna1	0.97303925	-0.03943009	0.70053168	1
Patj	1.01797953	0.025708556	0.70060067	1
Pm20d2	0.95513464	-0.066223971	0.70070185	1
Rtcd1	1.02069009	0.029544891	0.70074807	1
Bcan	1.02306889	0.032903294	0.70079291	1
Gmfg	0.9517204	-0.071390295	0.70104237	1
Myef2	1.01865853	0.026670522	0.70115436	1
Bcorl1	0.96114188	-0.057178677	0.70122119	1
Cox6a1	1.01807205	0.025839664	0.70125238	1
Cdk11b	1.02143806	0.030601715	0.70125602	1
Fam107b	0.97409849	-0.037860439	0.70128096	1
Etv1	0.98250845	-0.025458277	0.70141248	1
Defa11	0.79850983	-0.324617927	0.70148128	1
Fam49b	0.97763062	-0.032638619	0.70158824	1
Nfia	0.96591095	-0.050037899	0.70171589	1
Rbm20	0.9385659	-0.091470051	0.70172403	1
Metap1	1.023465	0.033461767	0.70190221	1
Smap2	1.0195014	0.027863751	0.70196536	1
Dpysl2	1.01832542	0.026198666	0.70198588	1
C2cd5	0.97919546	-0.030331225	0.70205473	1
Cahm	1.09421528	0.129896605	0.70206265	1

Ube2k	1.01865111	0.026660005	0.70223688	1
Slmap	0.97979344	-0.029450466	0.70227065	1
Timm44	0.97459167	-0.037130199	0.70237866	1
Wdr33	0.97362459	-0.038562488	0.70238348	1
Cdk5rap1	1.03072779	0.043663381	0.70259411	1
Ppp3cb	0.98216755	-0.025958934	0.70266766	1
Lrrc59	1.01835829	0.02624523	0.70270328	1
Pde8b	1.04788105	0.067474954	0.70278225	1
Dctd	0.91995124	-0.120370695	0.70278965	1
Rapgef3	1.03674704	0.052063932	0.70280717	1
Gyg1	1.01770801	0.025323699	0.70280911	1
Fam89b	1.02000072	0.028570174	0.70280998	1
Csgalnact2	1.0294395	0.041859049	0.70287298	1
Tmem107	1.06915547	0.096471652	0.7029014	1
Nudt5	0.96503525	-0.051346457	0.70303782	1
Gtf3c5	1.0309884	0.044028104	0.70321759	1
Nt5c1a	1.11824059	0.161230623	0.70329895	1
Pcdhgb7	0.96580132	-0.050201661	0.70335205	1
Tfcp2l1	1.05202581	0.073170104	0.70350362	1
Arid4a	1.02823733	0.040173292	0.70369176	1
Ctsl	0.9826101	-0.02530902	0.70374267	1
Dnajc10	1.01772942	0.02535405	0.70382058	1
Ipo4	0.97992898	-0.029250899	0.70385719	1
Rplp0	0.98248803	-0.025488258	0.70393111	1
Strbp	1.0187865	0.026851746	0.70396353	1
Pkp1	1.02953025	0.041986218	0.70401939	1
P4ha1	1.01960251	0.028006831	0.7040523	1
Dgat1	0.96740129	-0.047813636	0.70437368	1
Lyrm2	1.03961129	0.056044203	0.70446247	1
Gpc5	0.97083564	-0.042701023	0.7044761	1
Itpkc	1.04477542	0.063192863	0.70459611	1
Zbtb22	1.03011379	0.042803711	0.70461354	1
Styxl1	0.93721399	-0.093549611	0.70463176	1
Rogdi	0.98067724	-0.028149699	0.70472934	1
Alas1	0.97733726	-0.033071601	0.70475432	1
Nub1	1.01783925	0.025509728	0.70480657	1
PVR	0.95370583	-0.068383761	0.70482707	1
Timm9	0.96756135	-0.047574948	0.70489703	1
Chst15	1.02559805	0.036465429	0.70491119	1
Ube2c	1.11263643	0.153982255	0.70498305	1

Fgfr3	0.92824817	-0.107417533	0.70506093	1
Arpin	0.96288381	-0.054566377	0.70510172	1
Hspa2	1.02157538	0.030795658	0.70515769	1
Eif4enif1	1.02606394	0.037120638	0.70535143	1
Ube2d3	1.01750545	0.025036524	0.70547751	1
Pold4	1.04506623	0.063594374	0.7055233	1
Ing4	1.02601196	0.037047542	0.70566098	1
Spag1	1.04768697	0.067207724	0.70571792	1
Atad1	1.01828811	0.026145808	0.7057557	1
Erich2	0.91543914	-0.127464121	0.70575929	1
Rad50	0.97915442	-0.030391694	0.7058454	1
Echs1	1.02052201	0.029307299	0.70587747	1
Bmpr2	0.98167369	-0.026684549	0.70596757	1
Sin3a	0.97333544	-0.03899101	0.70598065	1
S100a5	0.90545193	-0.14329005	0.70601495	1
Lama5	1.03436189	0.04874103	0.70602085	1
Ppef2	1.07941856	0.1102544	0.70620635	1
Masp1	0.95871739	-0.060822496	0.70642559	1
Naa15	0.98204945	-0.026132419	0.70643324	1
Galnt1	1.0251871	0.035887232	0.70643891	1
Grsf1	1.01772073	0.025341737	0.70647531	1
Nuak1	1.03371808	0.047842777	0.70669855	1
Nicn1	1.02079906	0.029698905	0.70697299	1
Rras2	0.9679802	-0.046950561	0.70716994	1
Kank2	0.97867747	-0.031094608	0.70730572	1
RGD1561157	0.92620405	-0.110598027	0.70739381	1
Nbn	1.02011093	0.028726045	0.70744018	1
Klf10	0.97019445	-0.043654169	0.70753296	1
Zc3h18	0.97117371	-0.042198724	0.70772412	1
Ssr2	1.0208452	0.029764115	0.70775011	1
Kcmf1	0.98011616	-0.028975359	0.70781676	1
Trpv2	0.97850112	-0.031354591	0.7078884	1
RGD1305537	1.05315359	0.074715848	0.70801935	1
Dyrk3	1.06943028	0.096842427	0.70801958	1
Ufm1	1.02147591	0.030655177	0.70815642	1
Evi2a	1.05416928	0.07610655	0.70821256	1
St3gal4	0.97618506	-0.03477342	0.7082757	1
Uap1l1	1.0364717	0.051680727	0.70840011	1
Ablim2	0.97427619	-0.037597284	0.70844144	1
Fam118b	1.0388038	0.054923191	0.70845994	1

Zbtb25	0.96539663	-0.050806309	0.7085233	1
Pdcd6	1.02135181	0.030479901	0.70853728	1
P4ha3	0.92485569	-0.112699819	0.70859393	1
Cbr1	0.97815081	-0.031871177	0.7085963	1
Pbx4	1.07202923	0.100344244	0.7086045	1
Trerf1	0.94473423	-0.082019556	0.70877036	1
Mpc1	0.98254675	-0.025402039	0.70890784	1
Trappc9	0.97581267	-0.03532388	0.70897672	1
Irf2	0.9634462	-0.053723984	0.70907113	1
Eif4g3	0.98276079	-0.025087801	0.70929449	1
Podxl	0.97607717	-0.034932887	0.70934351	1
Lig4	1.02464645	0.035126202	0.70946152	1
Man1a1	1.02877239	0.040923836	0.70952881	1
Cyp4f6	1.04387694	0.061951653	0.70959178	1
Sar1a	0.98254824	-0.025399852	0.70970426	1
Dnm2	0.97810197	-0.031943215	0.70977831	1
Ncaph2	1.02254868	0.032169521	0.71000922	1
Nr2f6	0.96973616	-0.044335807	0.71003789	1
Zfp358	0.96174872	-0.056268085	0.71045849	1
Galnt14	1.02282136	0.032554193	0.7104863	1
Psip1	0.97983848	-0.029384141	0.71075964	1
Fam122a	1.04149054	0.058649729	0.71103067	1
Rnpc3	0.96886106	-0.045638308	0.71105091	1
Lrrc4c	1.02350533	0.033518621	0.71117628	1
Scarb2	0.98138954	-0.027102195	0.71124322	1
Hax1	0.97066544	-0.042953963	0.71124672	1
Adcy6	0.97521785	-0.036203559	0.71139439	1
Slc41a1	0.97509992	-0.036378038	0.71142145	1
Ap1s1	0.98213419	-0.026007935	0.71146034	1
Tmem150a	1.03706757	0.052509902	0.71146998	1
Sssca1	1.02864905	0.040750858	0.71147215	1
Ddost	0.9808823	-0.027848058	0.71156067	1
Adgrl4	0.96787549	-0.047106626	0.71156321	1
Gsto1	0.97838467	-0.031526297	0.7116602	1
Rap1b	0.97746067	-0.032889435	0.71166705	1
Tpra1	1.03215414	0.045658443	0.71168024	1
Smpd2	1.06011943	0.084226798	0.71173894	1
Uxs1	0.98138008	-0.0271161	0.7117552	1
Cspg4	1.02829739	0.040257561	0.7118016	1
Klf3	0.97523407	-0.03617957	0.71188672	1

Atg4a	0.97431585	-0.03753856	0.71189192	1
Tmed10	1.01762726	0.02520922	0.71195007	1
