# Supplementary figures and images for: Neural Inflammation in Thoracic Dorsal Root Ganglia Mediates Cardiopulmonary Spinal Afferent Sensitization in Chronic Heart Failure
Source: bioRxiv. 2025 Oct 23:2025.10.22.683960. Preprint. [Version 1] doi: 10.1101/2025.10.22.683960 (PMC12633454; doi:10.1101/2025.10.22.683960)

## Slide 1
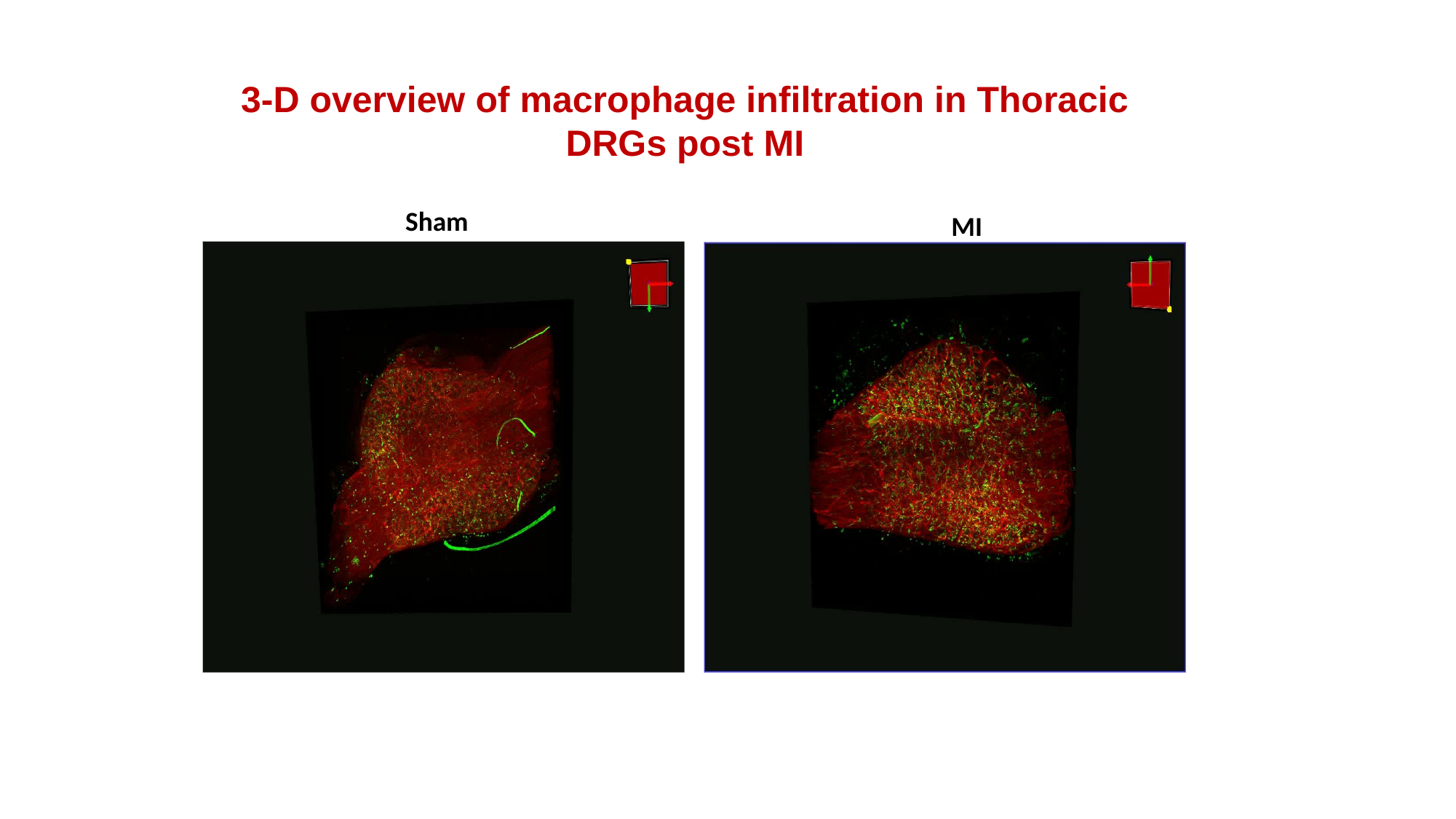

3-D overview of macrophage infiltration in Thoracic DRGs post MI
Sham
MI

Supplement: Supplement 2 [file media-2.pptx]
